# Xiaoyaosan Exerts Antidepressant-Like Effect by Regulating Autophagy Involves the Expression of GLUT4 in the Mice Hypothalamic Neurons

**DOI:** 10.3389/fphar.2022.873646

**Published:** 2022-06-16

**Authors:** Fu-Rong Yang, Xiao-Xu Zhu, Ming-Wang Kong, Xiao-Juan Zou, Qing-Yu Ma, Xiao-Juan Li, Jia-Xu Chen

**Affiliations:** ^1^ School of Basic Medical Science, Hubei University of Chinese Medicine, Wuhan, China; ^2^ Hubei Provincial Key Laboratory of Occurrence and Intervention of Rheumatic Diseases, Hubei Minzu University, Enshi, China; ^3^ Formula-Pattern Research Center, School of Traditional Chinese Medicine, Jinan University, Guangzhou, China; ^4^ School of Traditional Chinese Medicine, Beijing University of Chinese Medicine, Beijing, China

**Keywords:** depression, autophagy, GLUT4, hypothalamus, xiaoyaosan

## Abstract

Many studies have proven that autophagy plays a pivotal role in the development of depression and it also affects the expression of GLUT4 in the hypothalamus. Xiaoyaosan has been shown to exert antidepressant effects in a variety of ways, but its underlying mechanism by which Xiaoyaosan regulates autophagy as well as GLUT4 in the hypothalamus remains unclear. Thus, in this study, we established a mouse model of depression induced by chronic unpredictable mild stress (CUMS), and set up autophagy blockade as a control to explore whether Xiaoyaosan exerts antidepressant effect by affecting autophagy. We examined the effects of Xiaoyaosan on behaviors exhibited during the open field test, tail suspension test and sucrose preference test, and the changes in autophagy in hypothalamic neurons as well as changes in GLUT4 and the related indicators of glucose metabolism in CUMS-induced depressive mouse model. We found that CUMS- and 3-MA-induced mice exhibited depressive-like behavioral changes, with decreased LC3 expression and increased p62 expression, suggesting decreased levels of autophagy in the mouse hypothalamus. The expression of GLUT4 was also decreased, and it was closely related to the level of autophagy through Rab8 and Rab10. Nevertheless, after the intervention of Xiaoyaosan, the above changes were effectively reversed. These results show that Xiaoyaosan can regulate the autophagy in hypothalamic neurons and the expression of GLUT4 in depressed mice.

## Introduction

Depression is a common mental and psychological disorder whose main clinical features are feeling down, anorexia, fatigue, and cognitive impairment. Depression is also accompanied by insomnia, autonomic nervous system and gastrointestinal tract disorders, a strong sense of self-blame and inferiority, and even suicidal tendency. The WHO listed major depressive disorder (MDD) as the third leading cause of the global burden of disease, and it is expected that the disease will rank first by 2030 (WHO, 2008). The economic burden caused by MDD is approximately $2.5 trillion in the US, accounting for 10% of the total global disease burden (Bach et al., 2020). Depression is also a major independent risk factor for other diseases, such as cardiovascular disease, dementia, diabetes, and osteoporosis (Knol et al., 2006). Depression can also severely restrict patients’ social and psychological functions, reduce their quality of life, and bring misfortune upon them (Pan et al., 2019). The high proportion of suicides among patients also has some direct adverse effects on social stability. Due to the diverse and complex causes and mechanisms of the disease, coupled with the high incidence of disease, depression is becoming a serious social, economic and medical problem threatening the world that has not yet been clarified.

Recent studies have shown that autophagy is mainly involved in the biological process of energy metabolism (Yang et al., 2019). Autophagy is a key mechanism for eukaryotes to maintain cell renewal and homeostasis based on the intracellular lysosomal degradation pathway (Luo et al., 2019). LC3 is a reliable marker of autophagosome, and p62 is the most characteristic molecule and degradation product of autophagy, and the expressionls of both can reflect the autophagic activity of cells. LC3 II/LC3 I can reflect the degree of autophagy, the ratio is positively correlated with the degree of autophagy, and p62 is inversely proportional to the degree of autophagy. Many studies have shown that the onset of depression is related to the degree of autophagy. Some studies have shown that enhanced autophagy can alleviate depression-like behaviors to some extent (Shu et al., 2019), while others have found the opposite (Liao et al., 2021). Previous research has found that mice with depression induced by chronic restraint stress exhibit increased fasting and postprandial blood glucose levels, and reduced insulin levels (Pan et al., 2019) and these symptoms of glucose metabolism disorders are related to central neuron autophagy.

Glucose transporter-4 is the main regulatory protein in the body to take up glucose from peripheral tissues, and it plays an important role in maintaining the body’s glucose homeostasis (Govers, 2014; Amira et al., 2019). GLUT4 plays a role in regulating systemic glucose homeostasis in hypothalamic neurons (Hongxia et al., 2015), and autophagy plays an important role in regulating the translocation of GLUT4 (Safa et al., 2018). Rab protein, a member of the Ras GTPase superfamily, is closely related to autophagy in regulating the maturation, transportation and fusion of GLUT4 vesicles (Ao et al., 2014). Rab8 and Rab10 jointly regulate the maturation of autophagy and the transport of GLUT4 vesicles and are an important regulatory mechanism by which autophagy participates in the regulation of GLUT4 translocation (Jaldin-Fincati et al., 2017). We screened and preliminarily verified that GLUT4 is the target molecule of hypothalamic neurons involved in glucose metabolism through high-throughput sequencing of whole-genome DNA methylation (Li, X. J, 2018). However, the involvement of GLUT4 in hypothalamic neurons during the biological process of glucose metabolism and its relationship with autophagy are currently unclear.

Xiaoyaosan was originally described in Taiping Huimin Heji Jufang, a chinese materia medica officially compiled in the Song Dynasty of China (960–1127 AD). The Xiaoyaosan formula contains eight herbs, including Radix Bupleuri: Radix Paeoniae Alba: Angelica Sinensis:Poria Cocos: Rhizoma Atractylodis Macrocephalae: Rhizoma Zingiberis Recens: Radix Glycyrrhizae: Herba Menthae = 5:5:5:5:5:5:4:1. We have been conducting researches on the antidepressant effects of Xiaoyaosan for many years, and we have found that Xiaoyaosan has a modulating effect on depression-like behaviors caused by CUMS (Yan et al., 2018; Ding et al., 2020). However, whether Xiaoyaosan can regulate the autophagy of hypothalamic neurons and further affect the glucose metabolism in a mouse model of depression through GLUT4 is not yet known. CUMS is known to be risk factor for psychiatric disorders, and stress-induced animal models of mood disorders have therefore been widely investigated. Since it effectively induces the pathophysiology of depression, CUMS is considered as an appropriate paradigm for imitating psychiatric-related illnesses in rodents. In view of this, we established a mouse model of depression induced by CUMS for 6 weeks, evaluated the model, and related indicators of autophagy and energy metabolism were detected. After verifying that the level of autophagy decreased in depressed mice, we then injected the autophagy inhibitor 3-Methyladenine (3-MA) into the control mice, and tested the behavioral and related indicators of autophagy and energy metabolism. Hence, we explored the regulatory effect of Xiaoyaosan on the autophagy and GLUT4 mediated by it.

## Materials and Methods

### Animals

One hundred and eight specific pathogen-free (SPF), 6-8-week-old male C57BL/6J mice weighed 18–25 g. The animals were provided by Beijing Sibeifu Biotechnology Co., Ltd.(SYXK (yue)2019–0010) and were housed in an animal room with a barrier system under standard experimental conditions (The permit number of the local Ethics Committee is 20210512–05). The mice were bred in the SPF animal room of Jinan University, at room temperature (21 ± 2)°C, relative humidity of 30%–40%, and light and dark conditions for 12 h (light, 7:00∼∼19:00, dark, 19:00∼7:00). There were two batches of mice and all of them were given free access to distilled water and fed a standard rodent diet.

**FIGURE 1:**
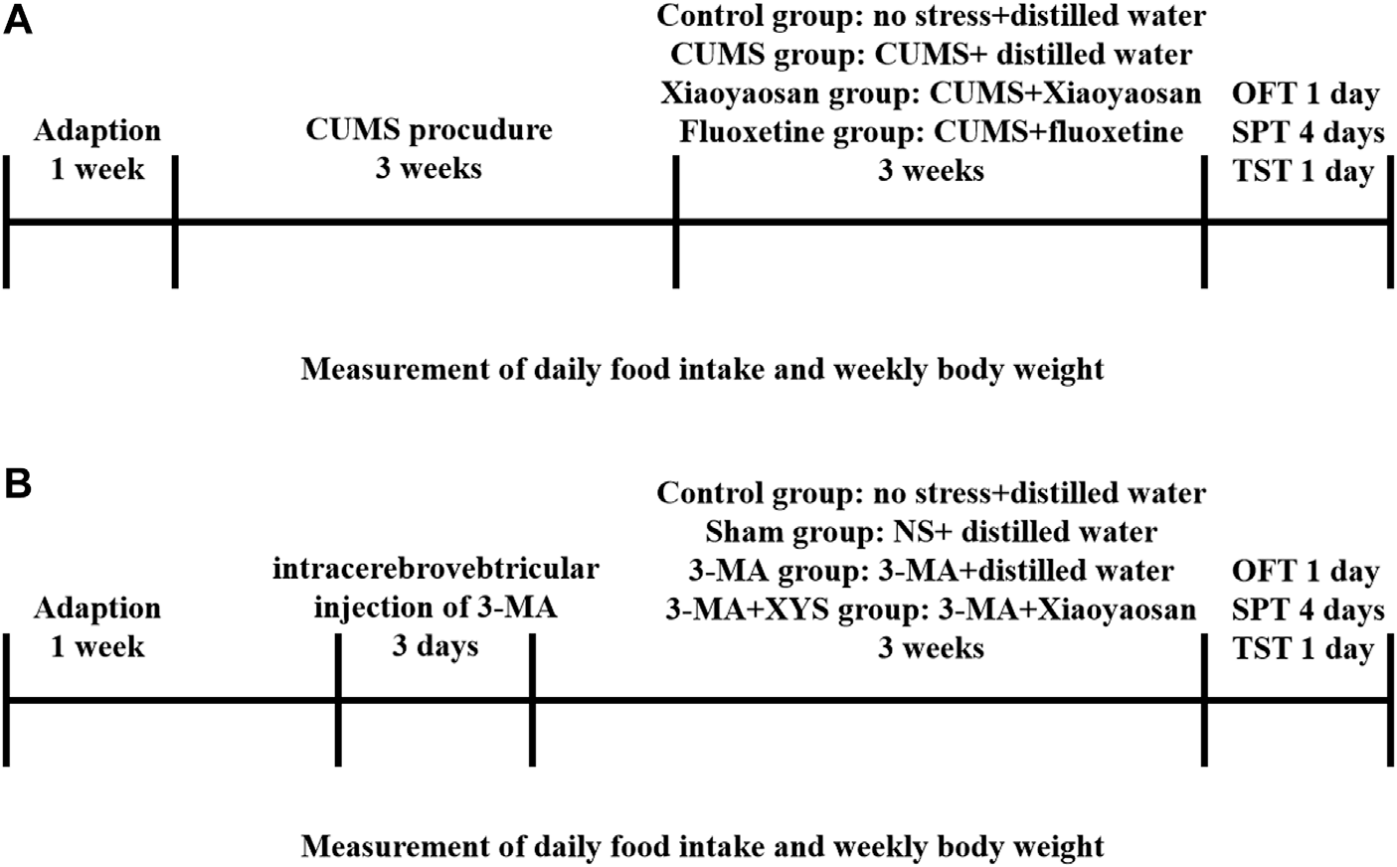
Experimental schedule in this study. **(A)** After 1 week of habituation, except for those in the control group, the first mice in the three other groups were subjected to chronic unpredictable mild stress (CUMS) stress for 3 weeks. The mice in each group received the corresponding treatment for 3 weeks after the stress ended. Daily food intake and weekly body weight were recorded from the first day of stress until the end of treatment. The open field test (OFT), sucrose preference test (SPT) and tail suspension test (TST) were performed. Then, the animals were sacrificed, and tissue was collected and cryopreserved. **(B)** After 1 week of habituation, except for those in the control group, the first mice in the three other groups were injected with NS and 3-MA respectively. The mice in each group received the corresponding treatment for 3 weeks after the injection. Daily food intake and weekly body weight were recorded from the first day of wound recovery until the end of treatment. The open field test (OFT), sucrose preference test (SPT) and tail suspension test (TST) were performed. Then, the animals were sacrificed, and tissue was collected and cryopreserved.

After 7 days of habituation, the first batch of 48 mice were randomly divided into four groups: the control group, CUMS group, Xiaoyaosan group and fluoxetine group, twelve mice in each group, were fed in three cages. The protocols were approved by the Institutional Animal Care and Use Committee of Jinan University and strictly abided by the Beijing Experimental Animal Ethics and Welfare Guidelines (released on 2006-01-01) to minimize animal suffering. Except for the control group, the other three groups of mice received CUMS for three consecutive weeks. The CUMS paradigm consists of various mild stressors, such as food and water deprivation for 24 h, 45° tilted cage for 24 h, inversion of day/night light cycle for 24 h, odor (glacial acetic acid) stimulusfor 24 h, wet bedding for 24 h, 45°C oven heat drying for 5 min, restraint for 2 h, etc. We implemented a stressor on 1 day, and each of these stressors was guaranteed not to be applied on consecutive days (Figure 1A).

As shown in Figure 1B, the second batch of 60 mice were randomly divided into four groups after 7 days of habituation: the control group, Sham group, 3-MA group and 3-MA + XYS group, fifteen mice in each group, were fed in three cages. Except for the mice in control group, the others were deeply anesthetized with sodium pentobarbital and fixed in a stereotaxic apparatus with a pair of ear bars and an incisor bar. A small incision was made to expose the skull, and the bregma was labelled to orient the coordinates. The coordinates of the lateral ventricle were 1.4 mm anteroposterior (AP), 1.1 mm mediolateral (ML), and 4.9 mm dorsoventral (DV) to the bregma. After marking the position, first drill the skull with a suitable size electric drill, then fix the micro syringe, and start the injection procedure in the system (RWD Life Science, Shenzhen, China). We injected normal saline (NS) and 3-MA (Sigma-Aldrich, United States) (dissolved with normal saline) into the hypothalamus of the Sham group and 3-MA group, 3-MA + XYS group, respectively. One-time injection of 3-MA into mouse hypothalamus at a concentration of 12 μg/μl, 30 μg per mouse. The mice were immediately removed from the stereotaxic apparatus and placed in an incubator to maintain their basal body temperature after surgery. Mice were given intramuscular injections of penicillin for three consecutive days after surgery to prevent infection. After the treatment of the two batches of mice was finished, the open field test (OFT), sucrose preference test (SPT) and tail suspension test (TST) were performed. After one of behavioral tests finished, the mice were allowed to rest for a day before proceeding to the next one.

### Preparation of Drugs

The Xiaoyaosan dry extract (provided by Jiuzhitang Co., Ltd. (Changsha, China)) was produced based on the procedure described in the Chinese Pharmacopoeia 2015 Edition (National Pharmacopoeia Commission, 2015). We completed the study on the drug properties of Xiaoyaosan compounds using DrugBank and a gene chip combined with cMap (Yuan et al., 2020). According to the 60-kg/d dosage conversion from human to animal (Ao et al., 2014), the mice in the Xiaoyaosan group were given Xiaoyaosan powder dissolved in distilled water at a dose of 0.658 g/kg/d and 0.1 ml/kg bodyweight via gavage. Fluoxetine hydrochloride tablets (20 mg/tablet) were obtained from Patheon France (packaged by Lilly Suzhou Pharmaceutical Co., Ltd, Suzhou, China), and the fluoxetine group was given fluoxetine dissolved in distilled water at a dose of 2.6 mg/kg/d and 0.1 ml/kg body weight via gavage every day. The control group and CUMS group were given an equal volume of distilled water.

Starting from the fourth week of CUMS, intragastric gavage was administered to mice in each group 1 h after the completion of CUMS treatment every day, and those requiring 24 h for stress were given intragastric gavage at 21:00. The gavage treatment was continued for 3 weeks.

### Body Weight and Food Intake

To evaluate whether CUMS will affect the physical conditions of the mice, the body weight and the food intake of mice in each group were weighed every week from the first day of the experiment. The data were continuously monitored and recorded for 6 weeks. The formula for calculating the daily food intake of mice is daily food intake = total daily food weight (Gram)-daily remaining food weight (Gram).

### Open Field Test

1 h later after finishing CUMS stress and administration, all C56BL/6J mice were subjected to an open field test to evaluate the behavioral characteristics of mice in each group, such as autonomous activities and space exploration. We placed the open field box (50 × 50 × 50 cm) with a central zone (40 × 40 cm) in a quiet operation room, installed a camera directly above the open field box and connected the camera to a computer for real-time observation and recording. Thirty minutes before the start of the test, the mice of each group were put into the quiet operating room for habituation. We gently placed the mouse into the center of the open field box and started timing and video recording. Observer 5.0 and EthoVision XT (Noldus Information Technology, Netherlands) software were used to analyze behavioral parameters such as the total distance traveled and the time spent in the open area of each group of mice within 5 min. After the test, we first cleaned the urine and feces left by the test mouse with a wet towel, and then sprayed 75% ethanol to remove the residual odor. After the smell dissipated, the next mouse was put into the box for testing. The observer remained quiet throughout the experiment.

### Tail Suspension Test

The tail suspension test was used to evaluate the activity and the desperate behavior of mice. Thirty minutes before the start of the test, the mice were put into a quiet operating room to habituate to the environment. A special tail suspension box was placed in the quiet operating room. A camera was installed directly in front of the tail suspension box and connected to a computer for real-time observation and video recording. We hung the mice above the suspension box with tape at a height of approximately 30 cm from the bottom of the box and started timing and video recording. Observer 5.0 and EthoVision XT software were used to analyze the immobility time of mice in each group within 5 min.

### Sucrose Preference Test

After the test was over, we assessed the degree of anhedonia in mice to determine the severity of depressive state by a sucrose preference test. All mice were trained to habituate to the sucrose water for 72 h. First, we put two bottles of 1% sucrose water in each cage at the same time (within the first 24 h). Then, we placed a bottle of distilled water and a bottle of 1% sucrose water in each cage (the second 24 h). Finally, after the mice were fasted for 24 h, we performed SPT. Each mouse was tested in a cage. A bottle of 1% sucrose in water and a bottle of distilled water was simultaneously placed in each cage. The mice were allowed to drink freely for 1 h, and then the two bottles were removed and weighed to calculate the sucrose preference rate of the mice. The preference for sucrose was calculated according to the following formula: sucrose consumption (g)/[water consumption (g) + sucrose consumption (g) ]×100%.

### Intraperitoneal Injection Glucose Tolerance Test (IPGTT)

After finishing the SPT, and 24 h after the mice resumed their diet, they were fasted again for 12 h. We intraperitoneally injected 2 mg/g glucose prepared as 0.2 g/ml glucose solution into each mouse, collected blood from the tail of the mice at different time points at 0, 15, 30, 60, and 120 min after being injected, and then detected and recorded blood glucose levels. We used a fully automatic biochemical detector (Roche, Switzerland) to test blood glucose in accordance with the instructions.

### Enzyme-Linked Immunosorbent Assay

Twenty-four hours after the IPGTT, the mice were deeply anesthetized with sodium pentobarbital, and retro-orbital blood was collected. The supernatant was collected after centrifugation, and the serum insulin concentration was assayed with an ELISA kit (Solarbio Life Science, Beijing, China) according to the instructions. Wash buffer and antibody-HRP conjugate working solutions were prepared after the reagents were warmed, and the standards were serially diluted. Set up blank control wells and add samples and reagents to the corresponding well plates. We measured the OD 450 value immediately after the operation, drew a standard curve, and calculated the sample concentration.

### Transmission Electron Microscopic Analysis

A small portion (∼1 mm^3^) of the hypothalamus from one mouse in each group was sectioned and incubated for 2 h at 4°C in 2.5% glutaraldehyde (Solarbio Life Science, P1126, Beijing, China). The specimens were rinsed with 0.1 M phosphoric acid, postfixed in 1% osmium tetroxide for 2–3 h, and then rinsed with 0.1 M phosphoric acid again for 15 min × 3 times. The specimens were dehydrated with different concentrations of ethanol at 4°C, and put into acetone three times at room temperature, then embedded in epoxy resin. After curing in the oven, the specimen is cut into ultrathin sections of 50–60 nm which were stained with lead citrate and examined by transmission electron microscopy (JEM-1010, Japan). Then we randomly selected any cell in the hypothalamus tissue for observation, with 10 visual fields for each cell and counted the number of autophagosomes in the 10 visual fields of each group by observing the electron microscope pictures.

### Immunofluorescent Staining

Mice brain tissue was fixed in 4% paraformaldehyde (Solarbio Life Science, P1110, Beijing, China) for 48 h, embedded in conventional paraffin and then sliced into 5 μm coronal sections. After numbering the slices, they were placed on a slide warmer for more than 2 h and then they were deparaffinized with xylene and gradient alcohol. Antigen retrieval was performed on the slices: The slices were placed in a container containing sodium citrate buffer, and antigen retrieval was performed in a pressure cooker. After the solution in the pressure cooker boiled for 3 min, the container was taken out and cooled naturally. Do not take out the slices. After cooling to room temperature, the slides were rinsed in PBS (pH 7.4) for 5 min × 3 times. Then they were placed in 0.5% Triton X-100(Solarbio Life Science, P1080, Beijing, China) (prepared in phosphate-buffered saline) for 30 min at room temperature. The slices were blocked in goat serum working solution for 30 min, then the blocking solution was removed, and incubated the slices in GLUT4 primary antibody (1; 500, Bioss Antibodies, bs-0384R, Beijing, China) at 4°C overnight. Then we incubated the slices with a fluorescent secondary antibody (1:200, Alexa Fluor^®^ 594, ab150116) in the dark, stained the nucleus (DAPI: Solarbio, S2110, Beijing, China) and mounted the slices. Finally, the slices were covered with cover glass, observed and photographed under an upright fluorescence microscope (Olympus, BX53/53 M, Japan). The immunofluorescence intensity of GLUT4 in the hypothalamus of mice in each group was analyzed by ImageJ.

### Western Blot Analysis

We observed the expression of LC3 (1:500, Cell Signaling Technology, #2775), p62 (1:500, Cell Signaling Technology, #5114), GLUT4 (1:500, Cell Signaling Technology, #2213), Rab8 (1:500, Cell Signaling Technology, #6975), and Rab10 (1:500, Cell Signaling Technology, #8127) by WB. Protein was extracted from hypothalamic tissue using the Tissue Protein Extraction Kit (Shanghai Beibo Biotechnology Co., Ltd, BB-3101–2, China) and the protein concentration was measured with a BCA Protein Quantitative Kit (Shanghai Biyuntian Biotechnology Co., Ltd, P0012, China). We loaded 20 μg of protein extract per well based on protein concentration and performed gel electrophoresis. Then we transfered the protein to a PVDF membrane (activated by soaking in methanol in advance). The molecular weight of the target protein is the basis for our selection of membrane specifications. For molecular weights less than 20 kDa, we used the 0.22 μm pore size membrane, and for molecular weights greater than 20 kDa, we used the 0.45 μm pore size membrane. After blocking the membrane with 5% nonfat milk in TBST for 30 min, incubate with corresponding specific antibodies against LC3B, p62, GLUT4, Rab8, and Rab10 overnight at 4°C. After incubation with the appropriate HRP-conjugated secondary antibodies. (goat anti-mouse IgG-HRP: 1:2,000, Asbio Technology, As006; goat anti-rabbit IgG-HRP: 1:2,000, Asbio Technology, As006) for 1 h and rinsing with tris buffered saline tween (TBST), The bands were visualized with an enhanced chemiluminescence reagent (Millipore, Billerica, MA) and then scanned and analyzed by an image analyser (Bio–Rad, California, United States). The intensity of the protein bands was normalized to GAPDH (1:1,000, Cell Signaling Technology, #5174).

### Real-Time Fluorescence Quantitative Polymerase Chain Reaction

The mRNA expression of LC3, p62, GLUT4, Rab8 and Rab10 mRNA in the hypothalamus was detected by RT–qPCR. Total RNA in the hypothalamus was extracted with TRIzol reagent (Solarbio Life Science, Beijing, China), and then the concentration was measured with a spectrophotometer (Eppendorf, Germany). First-strand cDNA was synthesized using a RevertAid First Strand cDNA Synthesis Kit (Thermo Fisher Scientific, Waltham, United States) on a C1000 TouchTM Thermal Cycler (Bio-Rad, California, United States) according to the manufacturer’s instructions in a total volume of 20 μl. Primers were designed based on published mRNA sequences using Primer three primer selection software, and then synthesized by a professional biotechnology company (Sangon Biotech Co., Ltd, Shanghai, China), and the sequences are shown in Table 1. PCR was used to amplify the cDNA with a Power SYBR^®^ Green PCR Master Mix kit (Thermo Fisher Scientific, United States) in a total volume of 25 μl on a CFX96 Real-time PCR System (Bio-Rad, California, United States) consisted of the following: template cDNA (2 ng/1 μl, equal to 100 ng of total RNA template), 2 × SYBR Green PCR Premix HS Taq mM dNTPs (12.5 μl), 10 μM forward primer (0.5 μl), 10 μM reverse primer (0.5 μl), complement deionized water to 25 μl. RT-qPCR reaction with the following cycling parameters: 94°C for 3 min; 40 cycles of 94°C for 30 s, 63°C for 30 s and 72°C for 30 s. The amplification reactions were performed in triplicate. In this study, β-actin was used as the reference gene and the results were calculated using the 2^-ΔΔCt^ method (Livak and Schmittgen, 2001).

**TABLE 1:** Primer sequences used in RT-qPCR analysis.

Gene symbol	Forward primer	Reverse primer
LC3	ATC​ATC​GAG​CGC​TAC​AAG​GG	AGC​CGA​AGG​TTT​CTT​GGG​AG
p62	GAC​AAG​AGT​AAC​ACT​CAG​CCA​AGC​A	CTC​CAT​CTG​TTC​CTC​TGG​CTG​TC
GLUT4	GCT​GAA​GGA​TGA​GAA​ACG​GAA​GT	TTC​TAC​TAA​GAG​CAC​CGA​GAC​CAA
Rab8	TGG​CAC​TCG​ACT​ATG​GGA​TCA	AGG​AGA​CTG​CAC​CGG​AAG​AA
Rab10	TCG​GAC​GAT​GCC​TTC​AAT​ACC	TGT​AGT​AGG​AGG​TTG​TGA​TGG​TGT​G
β-actin	GTG​ACG​TTG​ACA​TCC​GTA​AAG​A	GTA​ACA​GTC​CGC​CTA​GAA​GCA​C

### Statistical Analysis

SPSS (Statistic Package for Social Science) 21.0 software (IBM, Chicago, IL, United States) was used for statistical analysis of the data expressed as the mean ± standard error of the mean (SEM). We first performed a normality test on the data. When the data were normally distributed, one-way ANOVA was used for multiple group analysis, and LSD was further used for pairwise comparisons. When the variances were not uniform, a nonparametric test was used, and then a pairwise comparison was performed. When the data did not conform to a normal distribution, Kruskal–Wallis H was used to compare among K independent samples in the nonparametric test. When pairwise comparison was required, two independent samples were used for analysis. The body weight and food intake of mice in each group were analyzed by repeated measures analysis of variance. *p* < 0.05 was defined as significant.

## Results

### Xiaoyaosan Increased the Body Weight and Food Intake of Mice Induced by CUMS

**FIGURE 2:**
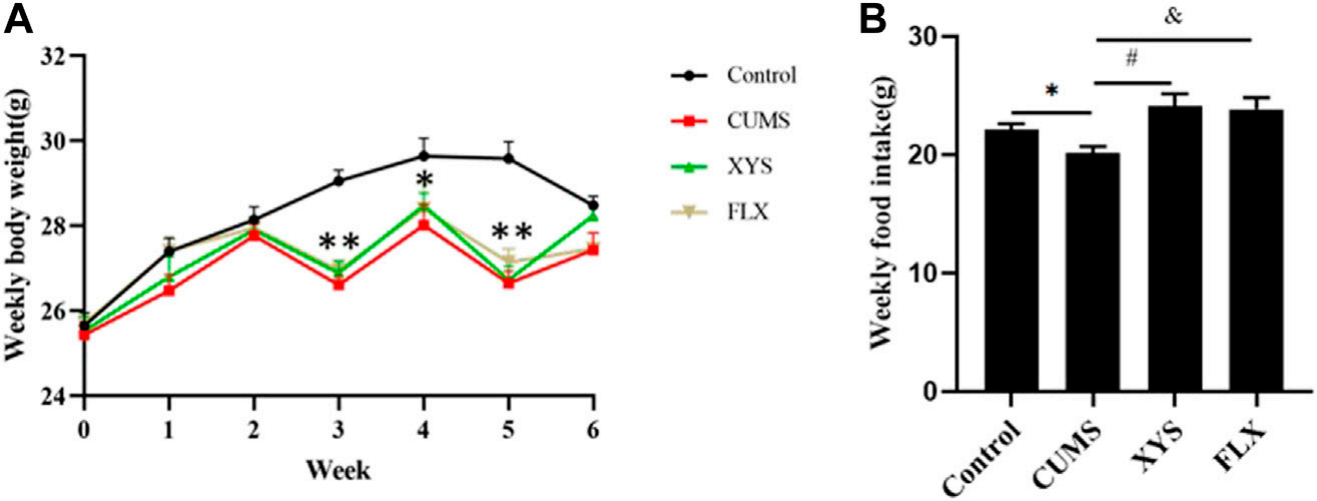
Effects of CUMS, Xiaoyaosan, and fluoxetine on the weekly body weight gain and daily food intake of the mice.**(A)** Weekly body weight (F (3, 44) = 20.310, *p* < 0.0001, n = 12) **(B)** Weekly food intake (F (3, 20) = 7.885, *p* = 0.0092, n = 12). The values shown represent the mean ± SEM. ^∗^ *p* < 0.05 or ^∗∗^ *p* < 0.01 compared with the control group.

During the study, the daily food intake and weekly body weight of the mice were monitored to verify the influence of CUMS on these parameters. As shown in Figure 2A, before the CUMS started, the weight of the mice in each group was the same. During the first and second weeks, the weight of the mice in each group increased gradually, and the increase in the control group was greater than that of the other groups. At the end of the third week, due to the food and water restriction of the mice on the day before the weighing, the body weight of the mice in the CUMS group, fluoxetine group and Xiaoyaosan group decreased (*p* < 0.01), except for the control group. After administration began, during the fourth week, the weight of the mice in the fluoxetine group and the Xiaoyaosan group was slightly higher than that of the CUMS group. The weight of the mice in all the groups was higher than that at any previous time point, but it was still lower than the control group (*p* < 0.05). At the end of the fifth week, due to the food and water restriction, the weight of the mice the CUMS group, fluoxetine group and Xiaoyaosan group decreased and was significantly lower than that of the control group (*p* < 0.01). By the end of the sixth week, the body weight of mice in the CUMS group, fluoxetine group and Xiaoyaosan group increased again, while the weight of the mice in the control group decreased due to external interference factors, and there was no significant difference compared with the other three groups (*p* > 0.05).

Due to the stress of food and water restriction, the food intake on that day was not weighed. The food intake results of the mice in each group at different time points are shown in Figure 2B. The weekly food intake of the control group was higher than that of the CUMS group (*p* < 0.05). After intervention with Xiaoyaosan and fluoxetine, the weekly food intake of mice increased correspondingly. (*p* < 0.05).

### Xiaoyaosan Ameliorates CUMS-Induced Depression-Like Behaviors in Mice

**FIGURE 3:**
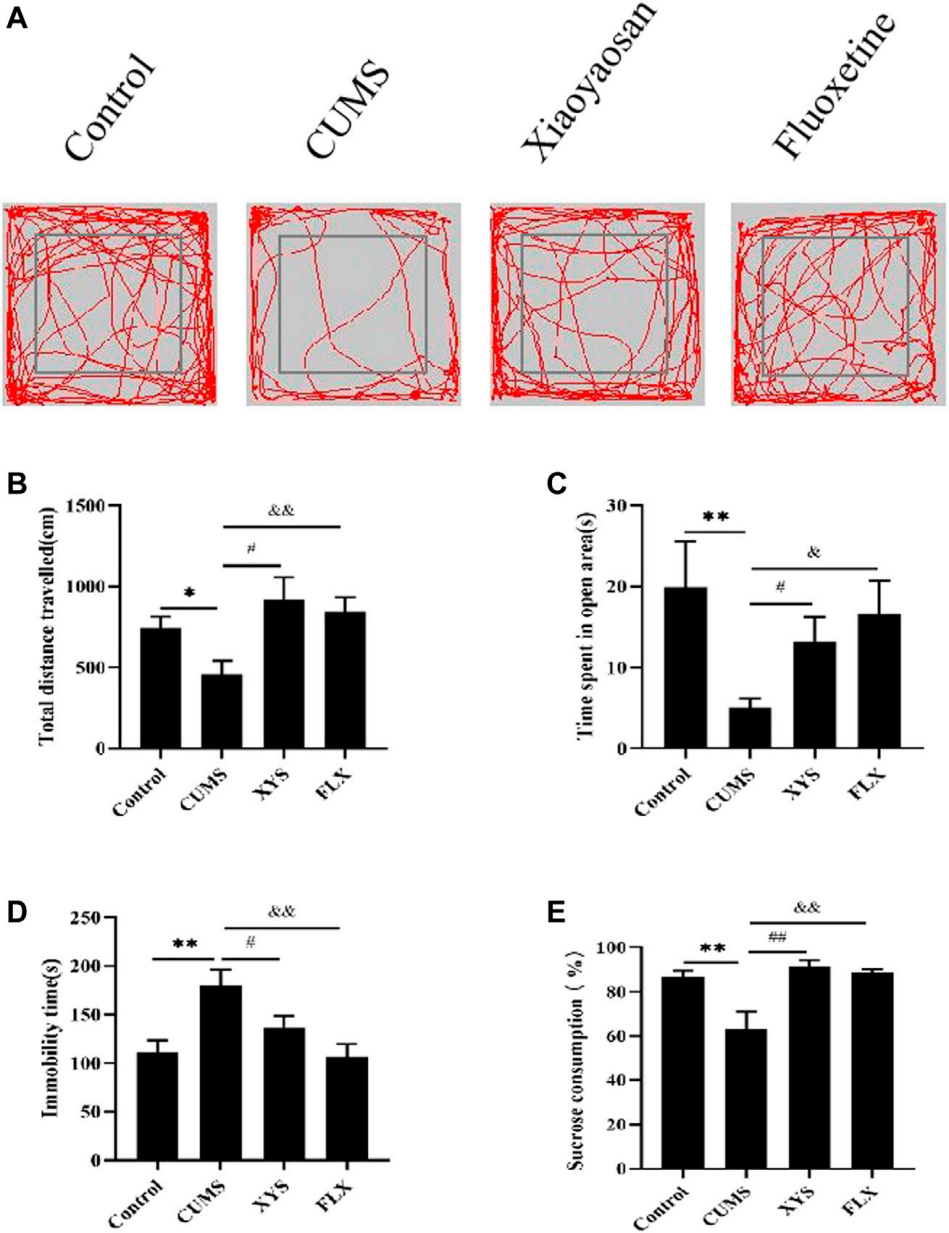
Xiaoyaosan ameliorate depression-like behaviors in mice induced by CUMS.**(A)** Map of the trajectory taken by mice in each group in the OFT assessed by video tracking software. **(B)** Total distance traveled in the OFT (F (3, 44) = 4.017, *p* = 0.0130, n = 12). **(C)** Time spent in the open area in the OFT (F (3, 44) = 2.723, *p* = 0.0556, n = 12). **(D)** Immobility time spent in the TST (F (3, 44) = 6.056, *p* = 0.0015, n = 12). **(E)** Sucrose consumption in the SPT (F (3, 44) = 8.602, *p* = 0.0001, n = 12). The values shown represent the mean ± SEM. The values represent the mean ± SEM.^∗^ *p* < 0.05 or ^∗∗^ *p* < 0.01 compared with the control group. #*p* < 0.05, ##*p* < 0.01 compared with the CUMS group. &*p* < 0.05, &*p* < 0.01 compared with the CUMS group.

During OFT, the total distance traveled by the mice in OFT of each group is shown in Figures 3A,B. Compared with the control group, the total distance traveled in the open field by the mice in the CUMS group is significantly reduced (*p* < 0.05). The mice in the Xiaoyaosan group and fluoxetine group improved their mobility to varying degrees after treatment, and the data in the Xiaoyaosan group were statistically different from those of the CUMS group (*p* < 0.05). The total distance traveled in the fluoxetine group was significantly greater than that of the CUMS group (*p* < 0.01). The results of the time spent in the open area in the OFT are shown in Figure 3C. The time spent in the open area in the CUMS group was largely less than that in the control group (*p* < 0.01). Compared with the CUMS group, the mice in the Xiaoyaosan group and fluoxetine group spent significantly more time in the open area (*p* < 0.05). These indicated that exposure to the CUMS procedure decreased the exploratory behavior of mice, but Xiaoyaosan can change the behavior of mice.

The immobility time in the TST is shown in Figure 3D. The immobility time of mice in the CUMS group was significantly longer than that of the control group (*p* < 0.01). The immobility time of mice in the Xiaoyaosan group was less than that in the CUMS group (*p* < 0.05). Compared with the CUMS group, the immobility time of the mice in the fluoxetine group was also significantly reduced (*p* < 0.01).

As shown in Figure 3E, CUMS reduced the sucrose consumption of mice in the CUMS group compared with the control group (*p* < 0.01). The sucrose consumption of the two treatment groups was significantly higher than that of the CUMS group (*p* < 0.01), indicating that both Xiaoyaosan and fluoxetine can effectively ameliorate the depression-like state in mice.

### Xiaoyaosan Promotes Hypothalamic Autophagy in CUMS-Induced Mice

**FIGURE 4:**
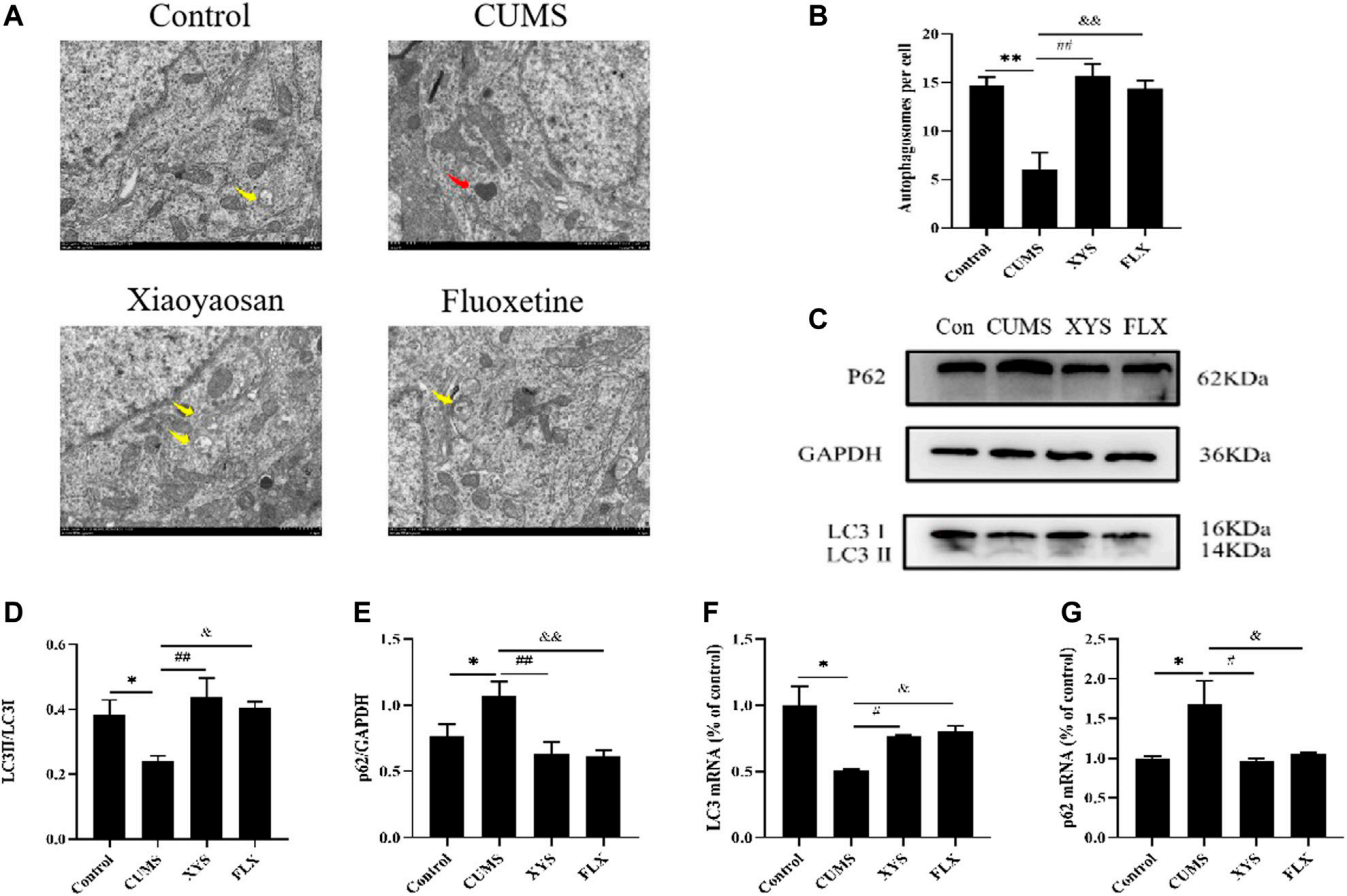
Xiaoyaosan promote hypothalamic autophagy in CUMS-induced mice. **(A)** Transmission electron microscope showed the situation of autophagosomes and mitochondria in the hypothalamus of the mice (scale bar = 1 µm). Mitochondria were disrupted in CMS group (the red arrow). Autophagosomes were observed in control, Xiaoyaosan and fluoxetine group (yellow arrows). **(B)** The number of autophagosomes in the hypothalamus of the mice (F (3, 8) = 13.383, *p* = 0.0017, n = 1). **(C)** Representative western blotting of LC3 and p62 expressions. **(D)** Quantitative analysis of protein levels of LC3(F (3, 16) = 4.904,*p* = 0.0133, n = 5). **(E)** Quantitative analysis of protein levels of p62(F (3, 16) = 5.931, *p* = 0.0064, n = 5). **(F)** The expression of LC3 mRNA (F (3, 12) = 7.326, *p* = 0.0047, n = 4). **(G)** The expression of p62 mRNA (F (3, 12) = 5.160, *p* = 0.0161, n = 4). The values represent the mean ± SEM.^∗^ *p* < 0.05 or ^∗∗^ *p* < 0.01 compared with the control group. #*p* < 0.05, ##*p* < 0.01 compared with the CUMS group. &*p* < 0.05, &*p* < 0.01 compared with the CUMS group.

We monitored autophagy in the hypothalamus of each group of mice by TEM, and the results are shown in Figure 4A and Figure 4B (the yellow arrow in the figure shows the autophagosome, and the red arrow shows the damaged mitochondaria). We found that the hypothalamic mitochondrial morphology of the CUMS group was damaged and that the number of autophagosomes was significantly reduced compared with that of the control group (*p* < 0.01). The Xiaoyaosan and fluoxetine groups had more complete mitochondrial morphology and more autophagosomes (*p* < 0.01).

As shown in Figure 4C and Figure 4D, the protein levels of LC3 especially LC3II in the hypothalamus of the CUMS group were lower than those of the control group, and the LC3II/LC3I ratio was also decreased (*p* < 0.05). Regarding the protein expression of p62 in the hypothalamus, the expression in the mice of CUMS group was higher than that of the control group (*p* < 0.05) (Figure 4E). Xiaoyaosan and fluoxetine reduced the expression of this protein in the hypothalamus of these two groups (*p* < 0.01 or *p* < 0.05). Changes in mRNA expression, shown in Figures 4F,G, exhibited the same trend (*p* < 0.05).

### Effect of Xiaoyaosan on Glucose Metabolism in the Hypothalamus of Mice Induced by CUMS

**FIGURE 5:**
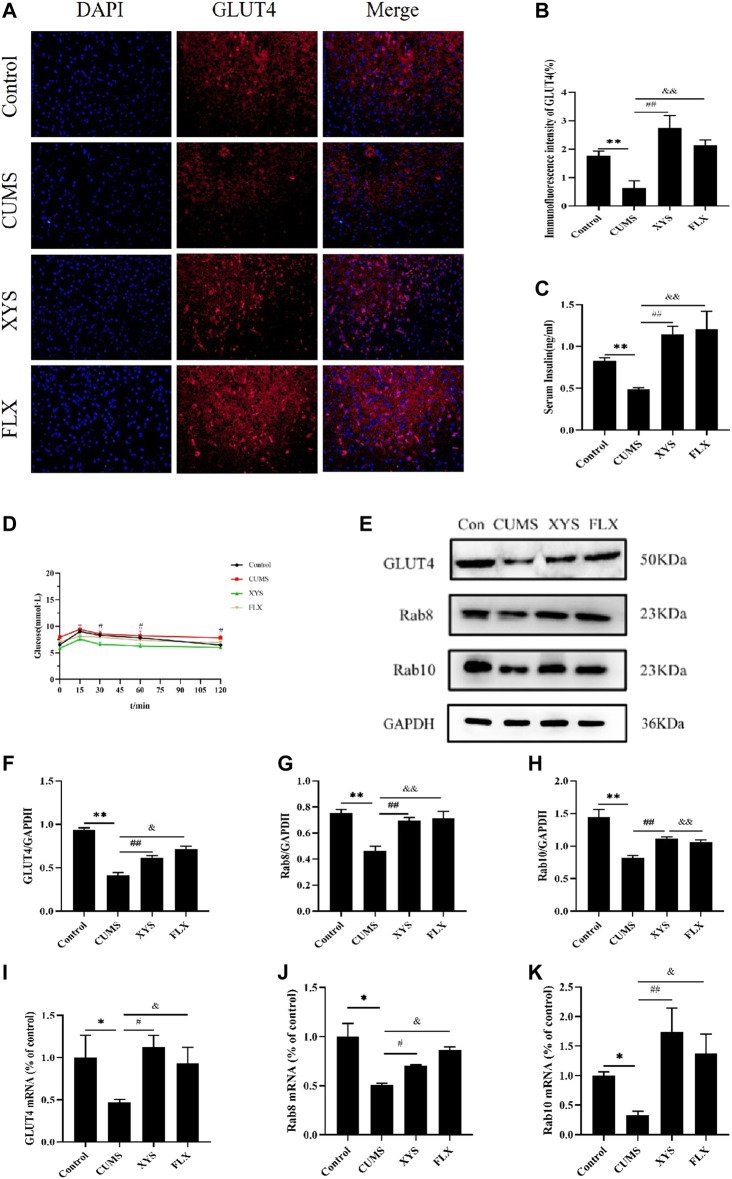
Effect of Xiaoyaosan on glucose metabolism in hypothalamus of CUMS-induced mice. **(A)** Immunofluorescence expression of GLUT4 in hypothalamus of the mice (scale bar = 20 µm). **(B)** Immunofluorescence intensity of GLUT4 (%) (F (3, 8) = 9.938, *p* = 0.0045, n = 3). **(C)** Serum insulin level of the mice (F (3, 16) = 7.973, *p* = 0.0018, n = 5). **(D)** The blood glucose of the mice (F (3, 36) = 2.542, *p* = 0.0716, n = 10). **(E)** Representative western blotting of GLUT4, Rab8 and Rab10 expressions. **(F)** Quantitative analysis of protein levels of GLUT4 (F (3, 16) = 57.780, *p* < 0.0001, n = 5). **(G)** Quantitative analysis of protein levels of Rab8 (F (3, 16) = 13.041, *p* = 0.0001, n = 5). **(H)** Quantitative analysis of protein levels of Rab10 (F (3, 16) = 14.085, *p* < 0.0001, n = 5). **(I)** The expression of GLUT4 mRNA (F (3, 12) = 2.542, *p* = 0.1054, n = 4). **(J)** The expression of Rab8 mRNA (F (3, 12) = 9.332, *p* = 0.0018, n = 4). **(K)** The expression of Rab10 mRNA (F (3, 12) = 5.218, *p* = 0.0155, n = 4). The values represent the mean ± SEM.^∗^ *p* < 0.05 or ^∗∗^ *p* < 0.01 compared with the control group. #*p* < 0.05, ##*p* < 0.01 compared with the CUMS group. &*p* < 0.05, &*p* < 0.01 compared with the CUMS group.

Based on the results of GLUT4 immunofluorescence in each group of mice (Figures 5A,B), the expression of GLUT4 in the dorsal medial nucleus and paraventricular nucleus of the hypothalamus in the mice of CUMS group was significantly lower than that in the control group (*p* < 0.01). Compared with the CUMS group, the expression of GLUT4 in the dorsal medial nucleus and paraventricular nucleus of the hypothalamus in the two treatment groups was drastically increased (*p* < 0.01).

In terms of mouse serum insulin results (Figure 5C), the serum insulin level of mice in the CUMS group was significantly less than that of the control group (*p* < 0.01). After administering Xiaoyaosan and fluoxetine, compared with the CUMS group, the decreased serum insulin levels in the two groups were effectively reversed and improved by a large margin (*p* < 0.01).

By testing the blood glucose of mice at 0, 15, 30, 60, and 120 min, we found that the blood glucose value of each group reached its peak at 15 min. The blood glucose level of the mice in the CUMS group was higher than that of the control group and the two treatment groups at each time point, and was drastically higher than that of the Xiaoyaosan group when at 60 min (*p* < 0.01). At 120 min, the blood glucose of the other three groups was statistically significantly lower than that of the CUMS group (*p* < 0.01 or *p* < 0.05) (Figure 5D).

As shown in Figures 5E–H, the GLUT4 protein expression in the hypothalamus of the CUMS group was significantly lower than that of the control group (*p* < 0.01), but GLUT4 expression increased after administion of Xiaoyaosan and fluoxetine to the mice, and there was a significant difference (*p* < 0.05). Regarding Rab8 and Rab10 protein expression in the hypothalamus, the expression in the CUMS group was lower than that of the control group (*p* < 0.01), and the expression in the Xiaoyaosan group and the fluoxetine group were higher than that in the CUMS group (*p* < 0.01).

The RT-qPCR results demonstrate the GLUT4 mRNA in the hypothalamus of CUMS group was lower than that of the control group (*p* < 0.05). After the mice received Xiaoyaosan and fluoxetine intervention, the GLUT4 mRNA expression increased (*p* < 0.05) (Figure 5I). The mRNA expression levels of Rab8 and Rab10 in the hypothalamus of the CUMS group were lower than those of the control group (*p* < 0.05); the expression levels of these two parameters in the mice of the Xiaoyaosan group and fluoxetine group were higher than those in the CUMS group, with significant differences (*p* < 0.01 or *p* < 0.05) (Figures 5J,K).

### Effects of Xiaoyaosan on the Body Weight and Food Intake of Mice Injected With 3-MA

**FIGURE 6:**
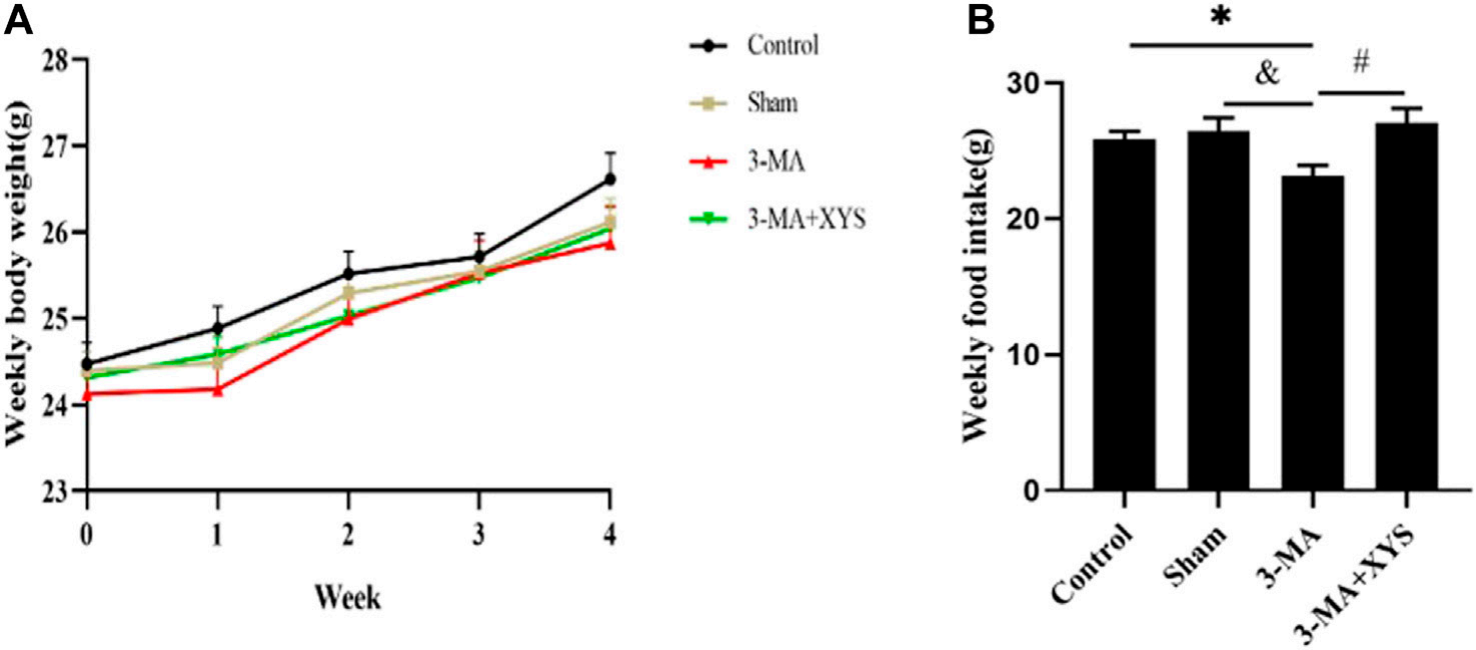
Effects of 3-MA and Xiaoyaosan on the weekly body weight gain and daily food intake of the mice.**(A)** Weekly body weight (F (3, 56) = 1.689, *p* = 0.1797, n = 15). **(B)** Weekly food intake (F (3, 8) = 4.002, *p* = 0.0518, n = 15). The values shown represent the mean ± SEM. ∗*p* < 0.05 compared with the control group. &*p* < 0.05 compared with the 3-MA group. #*p* < 0.05 compared with the 3-MA group.

To explore the role of autophagy in this study, we injected mice with 3-MA, an autophagy inhibitor, to observe the body weight changes of mice in each group (Figure 6A). Before the experiment, the body weight of the mice in each group was the same. While the experiment was in progress, the body weight of the mice was steadily increasing every week, and the weight of the control group increased slightly in the first and fourth weeks compared with the other groups. The body weight of mice in the sham group increased slightly in the second week compared with the other groups and mice in the 3-MA + XYS group showed a steady increasing trend. In the last week, the weight of the mice in the control group was the highest. The mice in the sham group and 3-MA + XYS group had the similar body weights but lower body weights than those in the control group. The weight of the mice in the 3-MA group was slightly lower than that of the other three groups (*p* > 0.05).

The changes in food intake of mice in each group at different time points after 3-MA injection are shown in Figure 6B. The weekly food intake of the control group and sham operation group was greater than that of the 3-MA group (*p* < 0.05). The mice in the 3-MA + XYS group had the largest food intake, while the mice in 3-MA group had the lowest food intake (*p* < 0.05).

### Effect of Xiaoyaosan on the Behavior of Mice Injected With 3-MA

**FIGURE 7:**
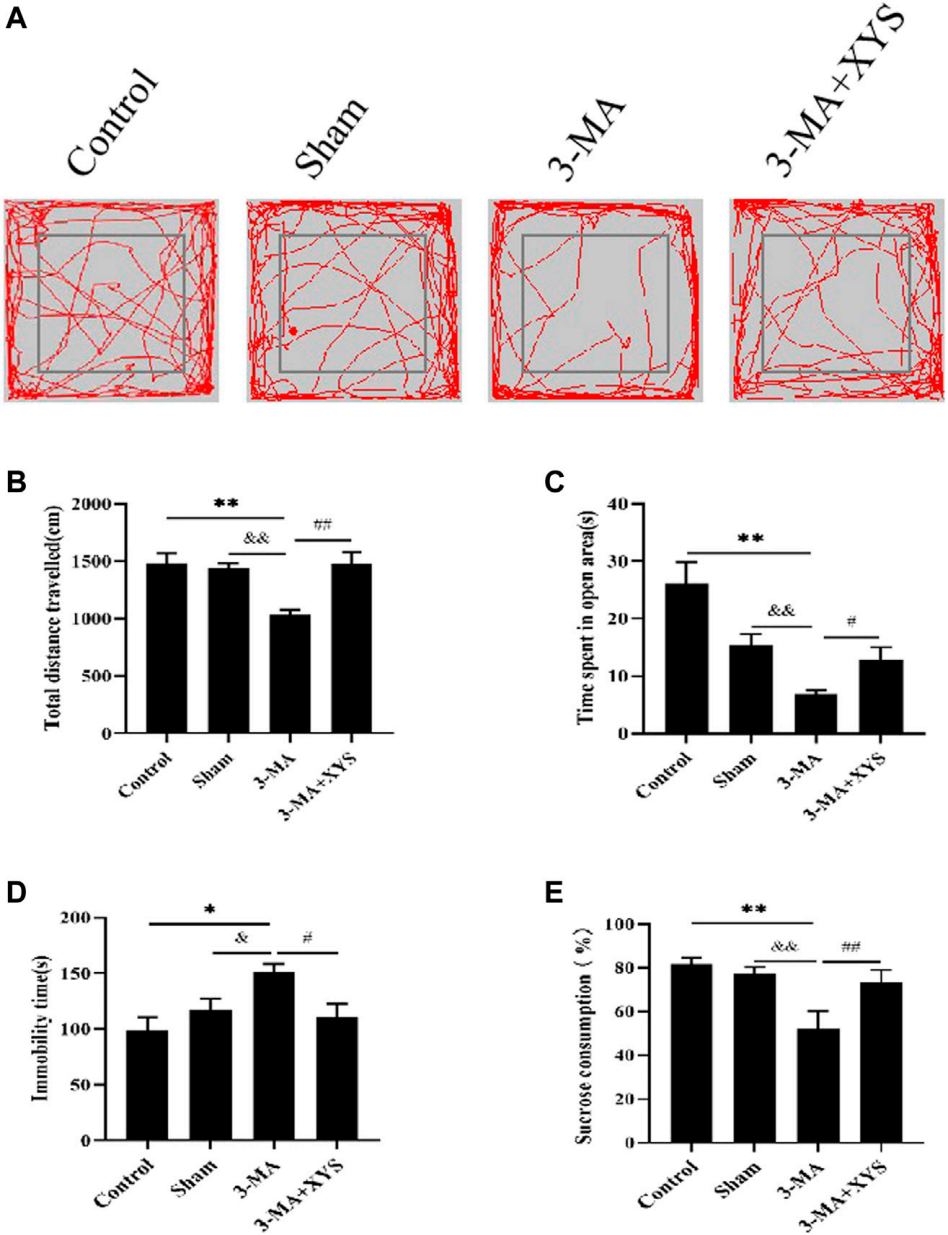
Xiaoyaosan ameliorate depression-like behaviors in mice Injected with 3-MA.**(A)** Map of the trajectory taken by mice in each group in the OFT assessed by video tracking software. **(B)** Total distance traveled in the OFT (F (3, 56) = 8.933, *p* < 0.0001, n = 15). **(C)** Time spent in open area in the OFT (F (3, 56) = 11.767, *p* < 0.0001, n = 15). **(D)** Immobility time spent in the TST (F (3, 56) = 4.534, *p* = 0.0065, n = 15). **(E)** Sucrose consumption in the SPT (F (3, 56) = 29.444, *p* < 0.0001, n = 15). The values shown represent the mean ± SEM. The values represent the mean ± SEM.^∗^ *p* < 0.05 or ^∗∗^ *p* < 0.01 compared with the control group. &*p* < 0.05, &*p* < 0.01 compared with the 3-MA group. #*p* < 0.05, ##*p* < 0.01 compared with the 3-MA group.

We evaluated the behavior of mice after they were injected with 3-MA. Catching sight of the results of OFT, we found that the total distance traveled by mice in the sham group did not change significantly (*p* > 0.05), while that of mice in the 3-MA group was significantly reduced (*p* < 0.01)compared with that of the control group. The total distance traceled of mice in sham group and 3-MA + XYS group was significantly greater than that in the 3-MA group (*p* < 0.01) (Figures 7A,B). Mice in the 3-MA group spent less time in the open area than that of the control group (*p* < 0.01), but there was no significant difference between the sham and the control group (*p* > 0.05). However, compared with the 3-MA group, the time spent in open area of the mice in sham group and 3-MA + XYS group was significantly increased, and the results were statistically different (*p* < 0.01, *p* < 0.05) (Figure 7C).

The immobility time of the mice in the 3-MA group during the TST was significantly longer than that of the control group (*p* < 0.05), while no significant difference existed between the sham group and the control group (*p* > 0.05). Compared with the 3-MA group,the immobility time of the mice in the sham group was significantly shorter (*p* < 0.05), and it was effectively reduced in the 3-MA + XYS group when compared with the 3-MA group (*p* < 0.05 (Figure 7D).

Regarding sucrose consumption, mice injected with 3-MA reduced sucrose consumption compared with the control group (*p* < 0.01). Data of the mice injected with saline were not much different from the control group (*p* > 0.05) but obviously higher than that of 3-MA group (*p* < 0.01), and the same was true for the 3-MA + XYS group (*p* < 0.01) (Figure 7E).

### Xiaoyaosan Increased Autophagy Expression in Mice Injected With 3-MA

**FIGURE 8:**
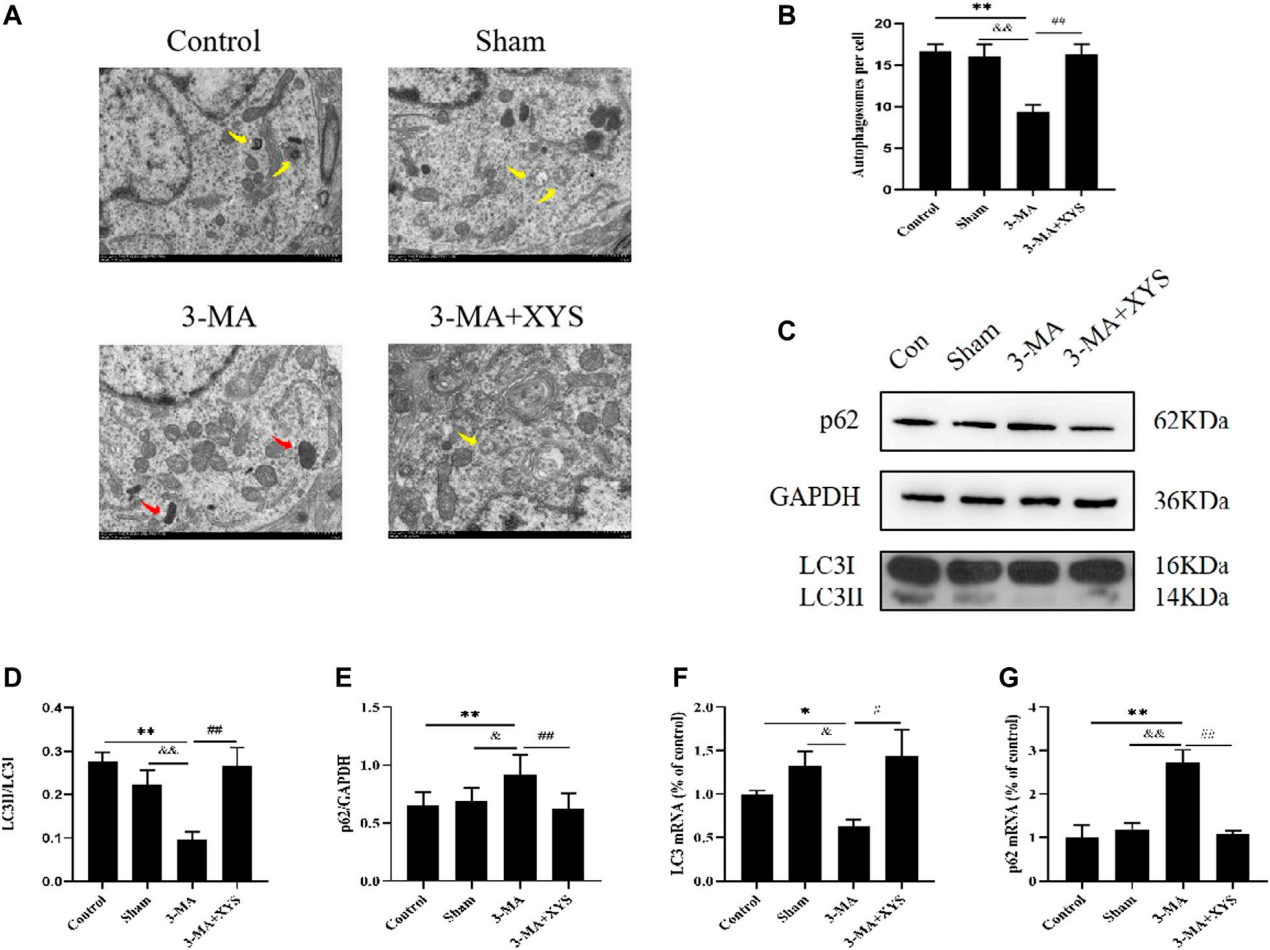
Xiaoyaosan Increased Autophagy Expression in Mice Injected with 3-MA. **(A)** Transmission electron microscope showed the situation of autophagosomes and mitochondria in the hypothalamus of the mice (scale bar = 1 µm). Mitochondria were disrupted in 3-MA group (the red arrow). Autophagosomes were observed in control, Sham and 3-MA + XYS group (yellow arrows). **(B)** The number of autophagosomes in the hypothalamus of the mice (F (3, 8) = 9.243, *p* = 0.0056, n = 1). **(C)** Representative western blotting of LC3 and p62 expressions. **(D)** Quantitative analysis of protein levels of LC3 (F (3, 16 = 7.689, *p* = 0.0021, n = 5). **(E)** Quantitative analysis of protein levels of p62 (F (3, 16 = 4.772, *p* = 0.0146, n = 5). **(F)** The expression of LC3 mRNA (F (3, 12 = 3.998, *p* = 0.0346, n = 4). **(G)** The expression of p62 mRNA (F (3, 12 = 14.176, *p* = 0.0003, n = 4). The values represent the mean ± SEM. ^∗^ *p* < 0.05 or ^∗∗^ *p* < 0.01 compared with the control group. &*p* < 0.05, &*p* < 0.01 compared with the 3-MA group. #*p* < 0.05, ##*p* < 0.01 compared with the 3-MA group.

As shown in Figures 8A,B, by taking a TEM image of the mouse hypothalamus, we found that the mitochondrial morphology of the mice in the 3-MA group was damaged, and the number of autophagosomes was obviously reduced compared with that in the control group (*p* < 0.01). The sham group had more mitochondrial damage than the control group, but there was little difference in the number of autophagosomes (*p* > 0.05). The number of autophagosomes in the hypothalamus of mice in sham group was significantly higher than that in the 3-MA group (*p* < 0.01), whereas after the mice injected with 3-MA were treated with Xiaoyaosan, their mitochondria were more complete, and the number of autophagosomes in the hypothalamus also increased significantly (*p* < 0.01).

Next we observed the expression of LC3 and p62 in the mouse hypothalamus (Figures 8C–E). Compared with the control group and sham group, the expression of LC3II was significantly lower in the mice injected with 3-MA, and the expression of p62 was increased, both of which were significantly different (*p* < 0.01 or *p* < 0.05). These two values between the sham group and the control group showed no differences. After treatment with Xiaoyaosan, the expression of LC3II in mice increased considerably, while the opposite trend was observed for p62 (*p* < 0.01).

LC3 mRNA expression in the hypothalamus was lower in the 3-MA group compared with the control group (*p* < 0.05), and the expression was slightly higher in sham group but not statistically significant (*p* > 0.05). After administration of Xiaoyaosan, the inhibitory effect of 3-MA on LC3 mRNA in the hypothalamus was effectively reversed compared with that of the 3-MA group (*p* < 0.05) (Figure 8F). The expression of p62 mRNA in the hypothalamus was just the opposite. The 3-MA group was significantly higher than the control and sham groups (*p* < 0.01). There was no difference between the sham group and the control group (*p* > 0.05), and p62 mRNA in the 3-MA + XYS group was effectively reduced by comparation with that in the 3-MA group (*p* < 0.01) (Figure 8G).

### Xiaoyaosan Improved Glucose Metabolism in Mice Injected With 3-MA

**FIGURE 9:**
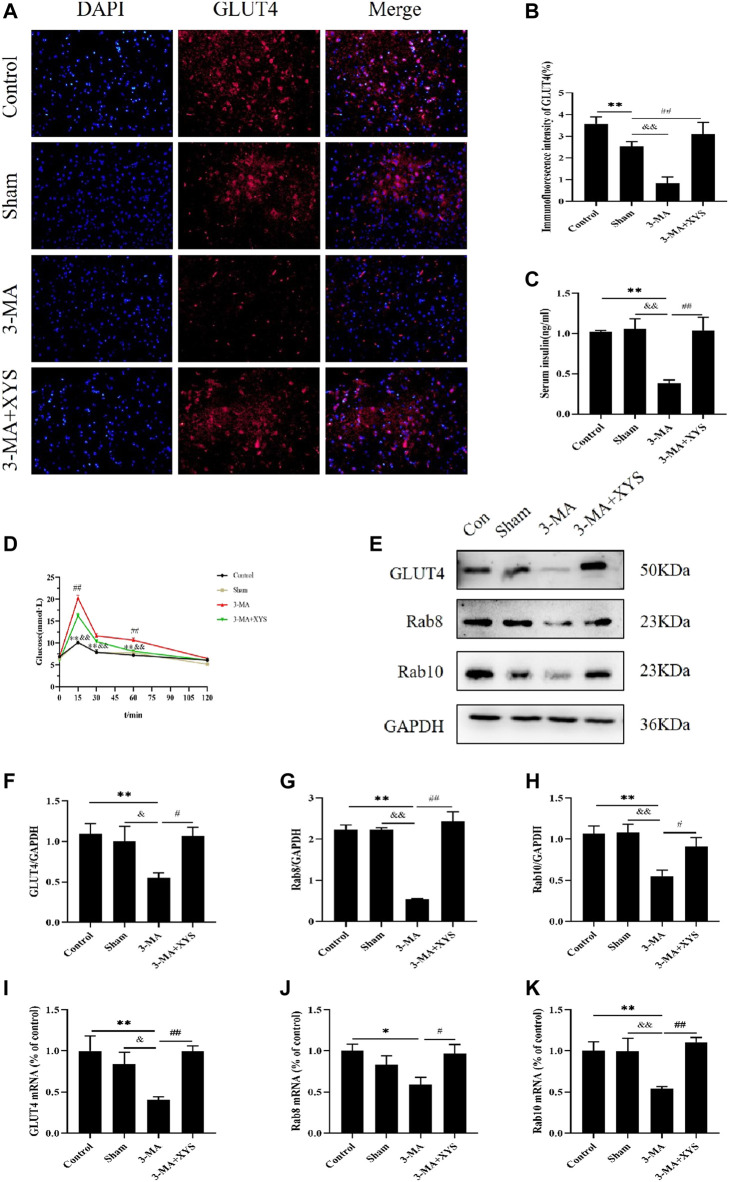
Effect of Xiaoyaosan on glucose metabolism in hypothalamus of mice injected with 3-MA. **(A)** Immunofluorescence expression of GLUT4 in hypothalamus of the mice (scale bar = 20 µm). **(B)** Immunofluorescence intensity of GLUT4 (%) (F (3, 8) = 10.900, *p* = 0.0034, n = 3). **(C)** Serum insulin level of the mice (F (3, 16) = 9.543, *p* = 0.0008, n = 5). **(D)** The blood glucose of the mice (F (3, 56) = 22.165, *p* < 0.0001, n = 15). **(E)** Representative western blotting of GLUT4, Rab8 and Rab10 expressions. **(F)** Quantitative analysis of protein levels of GLUT4 (F (3, 16) = 4.001, *p* = 0.0266, n = 5). **(G)** Quantitative analysis of protein levels of Rab8 (F (3, 16) = 23.664, *p* < 0.0001, n = 5). **(H)** Quantitative analysis of protein levels of Rab10 (F (3, 16) = 6.453, *p* = 0.0045, n = 5). **(I)** The expression of GLUT4 mRNA (F (3, 12) = 5.221, *p* = 0.0155, n = 4). **(J)** The expression of Rab8 mRNA (F (3, 12) = 3.655, *p* = 0.0443, n = 4). **(K)** The expression of Rab10 mRNA (F (3, 12) = 6.112, *p* = 0.0091, n = 4). The values represent the mean ± SEM. ^∗^ *p* < 0.05 or ^∗∗^ *p* < 0.01 compared with the control group. &*p* < 0.05, &*p* < 0.01 compared with the 3-MA group. #*p* < 0.05, ##*p* < 0.01 compared with the 3-MA group.

Figure 9A shows the immunofluorescence results of GLUT4 in mice. The expression of GLUT4 in the dorsal medial nucleus and paraventricular nucleus of the hypothalamus in the control group mice and in the paraventricular nucleus of the hypothalamus in sham group mice were both greater than that in the 3-MA group (*p* < 0.01). The sham group had relatively lower expression in the paraventricular nucleus and there was no significant difference in the dorsal medial nucleus compared with the control group with no significance level (*p* > 0.05). The expression of GLUT4 in the dorsal medial nucleus and paraventricular nucleus of the mice in the 3-MA + XYS group was increased compared with that in the 3-MA group (*p* < 0.01).

The results of serum insulin are shown in Figure 9B. The serum insulin levels of the mice in the 3-MA group were significantly lower than that of the normal group and sham group (*p* < 0.01). No difference was found between the control group and sham group (*p* > 0.05). After administration of Xiaoyaosan in mice injected with 3-MA, the decrease in serum insulin was distinctly improved (*p* < 0.01).

By monitoring the blood glucose at 0, 15, 30, 60, and 120 min, we found that the blood glucose values of mice in each group reached their peak at 15 min, and the 3-MA group exceeded the values of the other three groups at each time point. The blood glucose level of mice in the 3-MA group exceeded that in the control group and sham group at 15, 30, and 60 min (*p* < 0.01). After administration of Xiaoyaosan, the blood glucose level of mice injected with 3-MA decreased significantly at 15 and 60 min (*p* < 0.05). The blood glucose values of mice in all groups basically returned to the same level at 120 min (Figure 9C).

The reduction of GLUT4 protein expression in the hypothalamus of mice in the3-MA group was greater than that in the control and sham groups (*p* < 0.01 or *p* < 0.05). After administering Xiaoyaosan to the mice injected with 3-MA, the GLUT4 protein expression recoveried significantly (*p* < 0.05) (Figures 9D,E). Regarding the protein expression of Rab8 and Rab10 in the hypothalamus, both were significantly lower in the 3-MA group than that in the control group and sham group (*p* < 0.01). The expression of these two proteins in the hypothalamus of the 3-MA + XYS group was in sharp contrast with the 3-MA group (*p* < 0.01 or *p* < 0.05) (Figures 9D,F,G).

GLUT4 mRNA expression remained consistent with that of protein expression (Figure 9H). The levels of Rab8 and Rab10 mRNA in the 3-MA group were significantly lower than those in the control group and sham group (*p* < 0.05 or *p* < 0.01), but the expression of Rab8 was not much different from the latter (*p* > 0.05). There was no significant difference in the expression of the two genes between the control and sham groups (*p* > 0.05). The expression levels of Rab8 and Rab10 in the 3-MA + XYS group both increased sharply (*p* < 0.05 or *p* < 0.01) (Figures 9I,J).

## Discussion

The clinical application of Xiaoyaosan is very extensive and involves internal medicine, surgery, gynecology, pediatrics, etc. Xiaoyaosan has a relatively high probability of application in psychiatric and nervous system diseases, and depressive psychosis is the most common disease (Liu et al., 2020). Our team has also conducted in-depth research on Xiaoyaosan for treatment of depression for many years, and its mechanism involves many aspects (Yan et al., 2018; Ma et al., 2019; Hou et al., 2020). Xiaoyaosan regulates HPA axis dysregulation in depressed rats and inhibits its hyperactivity (Chen et al., 2008a; Song et al., 2020). Xiaoyaosan can be effective by regulating 5-HT metabolism disorder and promoting its synthesis (Jiao et al., 2018), can promote the recovery of synaptic structure and function in depression (Meng et al., 2013), and can reverse the structural damage of mitochondria and neurons, and increase the expression of neurotrophic factors (Chen et al., 2008b; Jiang et al., 2016). Xiaoyaosan can also have an antidepressant effect by improving intestinal microbes and regulating the gut-brain axis (Zhu et al., 2019; Hao et al., 2021). The antidepressant effect of Xiaoyaosan is not achieved through a certain way, but exerts its clinical efficacy through a combination of aspects, dimensions, levels, and targets. In this study, we explored the mechanism of Xiaoyaosan on depression through other ways.

Chronic unpredictable mild stress is known to be risk factor for psychiatric disorders, and it can effectively induces the pathophysiology of depression, so it has been widely used to establish modeling depression in rodents (Svitlana et al., 2019; Ma et al., 2021). Currently, the widely used assays for depression-related behaviours include the OFT, the SPT, the TST and so on. The OFT is based on the nature of rodents who simultaneously fear open spaces and wish to explore novel environments (Ferreira et al., 2018). In this study, the total distance travelled and the time spent in open area of CUMS-induced mice were decreased simultaneously, which means the miice are less eager to explore. The SPT utilizes rodents’ preferences for the taste of sugar. When animals show depressive-like behavior, the pleasure of drinking sweet water partly disappears, hence, the consumption of sucrose is significantly reduced (Wang et al., 2017). That’s exactly what we found in our study. The TST is based on the fact that animals subjected to the short-term, inescapable stress of being suspended by their tail, will develop an immobile posture to estimate the helpless emotion of the mice. In our study, the immobility time of the mice induced by CUMS was longer than the other mice, indicating the depressive state of mice to some extent. The results ot the above three tests all indicate that CUMS-induced mice exhibit depressive-like behaviors.

Many pathogeneses account for the onset of depression. In recent years, the relationship between autophagy and depression has received much attention. Studies have shown that reduced autophagy is found in animal models of depression (Zhao et al., 2017; Gulbins et al., 2018; Huang et al., 2018). LC3 is a reliable marker of autophagosomes (Feng et al., 2016), p62 is a characteristic molecule and degradation product of autophagy (Glick et al., 2010), and the expression levels of both can reflect the autophagy activity of cells. The amount of LC3II in the cell is positively correlated with the number of autophagosomes (Tanida, 2011). Autophagosomes and lysosomes are fused to form autophagolysosomes and then degraded by lysosomal enzymes (Tanida et al., 2008). p62 is a typical autophagy receptor, a multifunctional protein distributed in cells, and is involved in the proteasomal degradation of ubiquitinated proteins. When Atg7 (A protein associated with autophagy) and p62 are knocked out in combination, the accumulation of polyubiquitinated aggregates in cells can be reversed (Komatsu et al., 2007). Overexpression of p62 delays the delivery of proteasome substrates to the proteasome, thereby affecting its degradation. In addition, p62 and the proteasome can regulate the activity of HDAC6 deacetylase, thereby affecting autophagy degradation (Liu et al., 2016). In this study, we learned from the TEM, WB and RT-qPCR results that the number of autophagosomes in mice induced by CUMS was less than that in the control group, and this was changed after treatment. The mRNA and protein levels of the autophagy-related markers LC3 and p62 in the hypothalamus of mice incuded by CUMS were quite different from those in the control group and the two treatment groups: the LC3Ⅱ/LC3Ⅰ ratiodecreased, especially the expression of LC3Ⅱ ratio, which decreased significantly, while the expression of p62 increased. These results suggested that the level of autophagy in the hypothalamus of mice induced by CUMS was significantly reduced, but after Xiaoyaosan intervention of, the levels of LC3 and p62 were effectively reversed, indicating that Xiaoyaosan had a regulatory effect on autophagy in the hypothalamus of those mice.

GLUT4 is the primary regulatory mechanism by which glucose uptake occurs in the periphery and plays an important role in maintaining glucose homeostasis. In recent years, it has been discovered that in the central nervous system, GLUT4 is widely present in neurons in the cortex, olfactory bulb, hippocampus and hypothalamus. Due to the regulation of glucose sensing in the hypothalamus, GLUT4 in the hypothalamus can affect the whole body glucose metabolism. In general, insulin can promote the translocation of GLUT4 from the cell to the cell membrane in the form of vesicles in general. Insulin in the body can affect glucose metabolism by GLUT4 translocation, and can especially make an important contribution in regulating systemic glucose homeostasis in hypothalamic neurons. Autophagy plays an important regulatory role in the translocation of GLUT4 and can be used as a carrier for GLUT4 translocation. By regulating autophagy as a target for GLUT4, it is possible to control the translocation and circulation of GLUT4 to regulate insulin (Elhassan et al., 2018). Many proteins in the Ras GTPase superfamily are involved in the regulation of autophagy (Szatmari et al., 2014). Studies have confirmed that upregulation of Rab8 and Rab10 can increase the expression of GLUT4 in skeletal muscle cell membranes, thereby increasing glucose uptake in skeletal muscle (Samad et al., 2017), and Rab8 and Rab10 can colocalize with GLUT4 and autophagosomes (Zhang et al., 2017). Rab8 and Rab10 jointly participate in the process of regulating the maturation of autophagy and the transport of GLUT4 vesicles. At the same time, autophagy regulates the translocation and expression of GLUT4 through Rab8 and Rab10, which are closely related. Therefore, this study investigated glucose metabolism from the perspective of the effects of autophagy on the expression of GLUT4 mediated by Rab8 and Rab10. In this study, the results of immunofluorescence, WB and RT-qPCR showed that the changes in GLUT4, Rab8 and Rab10 expression in the hypothalamus of mice induced by CUMS were consistent with the changes in autophagy levels. At the same time, the insulin and blood glucose levels of depressed mice showed abnormalities. After Xiaoyaosan and fluoxetine intervention, the protein and mRNA levels of GLUT4, Rab8 and Rab10 were effectively increased, and the insulin and blood glucose levels of the mice in the treatment group were also significantly improved. The above results clarified that Xiaoyaosan regulates the expression of GLUT4 in the hypothalamus of mice induced by CUMS by regulating autophagy and affects the glucose metabolism throughout the body.

We found that the changes in glucose metabolism and autophagy have a causal relationship. Autophagy mediates Rab8 and Rab10 to affect the expression of GLUT4. To confirm this conclusion, we injected 3-MA, an autophagy inhibitor, into the cerebral ventricle of C57BL/6J mice to further study the regulatory effect of 3-MA on autophagy in the mice hypothalamus and observed the changes in Rab8, Rab10 and GLUT4, as well as the regulatory effect of Xiaoyaosan on them. 3-MA is a chemical drug that has been found to inhibit the expression of autophagy and is often used as an autophagy inhibitor in scientific research to study the effects of changes in autophagy levels on subjects. In recent years, as research on autophagy has gradually increased, studies on the relationship between autophagy and depression have also risen. To explore the effect of changes in autophagy on depression, 3-MA has been used as a control in many studies (Chen et al., 2019; Shih et al., 2019; Ali et al., 2020).

We found that after injection of 3-MA into the brain ventricle of mice, the results of OFT, TST and SPT showed that the mice in the 3-MA group exhibited depression with anxiety-like behavior change, indicating from another perspective that reduced levels of autophagy can induce depressive-like behavior. The number of autophagosomes in the hypothalamus decreased, indicating that autophagy was inhibited. The WB and RT-qPCR results were consistent, and both showed a decrease in autophagy levels. After treatment with Xiaoyaosan, the above phenomena were improved, indicating that Xiaoyaosan increased the autophagy level in the 3-MA group. The immunofluorescence results showed that Xiaoyaosan significantly improved the depression with anxiety-lik behavior of mice in the 3-MA group and increased the expression of GLUT4 in the hypothalamus of mice injected with 3-MA. At the same time, the expression of Rab8 and Rab10 showed the same trend as GLUT4. Therefore, the expression levels of Rab8, Rab10 and GLUT4 were consistent with the changes in autophagy, indicating that autophagy plays a nonnegligible role. The serum insulin level of the mice in the 3-MA group decreased, accompanied by an increase in blood glucose. Xiaoyaosan improved the disorder, and the mechanism may be related to the regulation of GLUT4. These results demonstrate that 3-MA effectively reduced the expression of autophagy and glucose metabolism in the mouse hypothalamus, and Xiaoyaosan ameliorated these effects. These results indicate that the mechanism of Xiaoyaosan’s antidepressant effect may be achieved by regulating the level of autophagy.

In addition, this study also selected fluoxetine as a positive control for the study of autophagy because fluoxetine is a commonly used clinical antidepressant with stable efficacy. Second, studies have shown that fluoxetine can promote the formation of autophagosomes in CUMS mice and exert its antidepressant effect by enhancing the level of autophagy (Shu et al., 2019). By comparison with fluoxetine, we can further determine the effect of Xiaoyaosan on depression.

## Conclusion

In general, our work provides new insights for revealing the biological mechanism of Xiaoyaosan’s antidepressant properties. Xiaoyaosan can improve glucose metabolism and its associated indicator GLUT4 in hypothalamus in the CUMS induced depressive behavior in mice, the mechanism of which may involve improving the hypothalamic autophagy. This study helps us to further understand the mechanisms of XYS as a potential antidepressant.

## Data Availability

The original contributions presented in the study are included in the article/Supplementary Materials, further inquiries can be directed to the corresponding authors.
