# Supplementary figures and images for: Xiaoyaosan Exerts Antidepressant-Like Effect by Regulating Autophagy Involves the Expression of GLUT4 in the Mice Hypothalamic Neurons (part 1 of 2)
Source: Front Pharmacol. 2022 Jun 16;13:873646. doi: 10.3389/fphar.2022.873646 (PMC9243304; doi:10.3389/fphar.2022.873646)

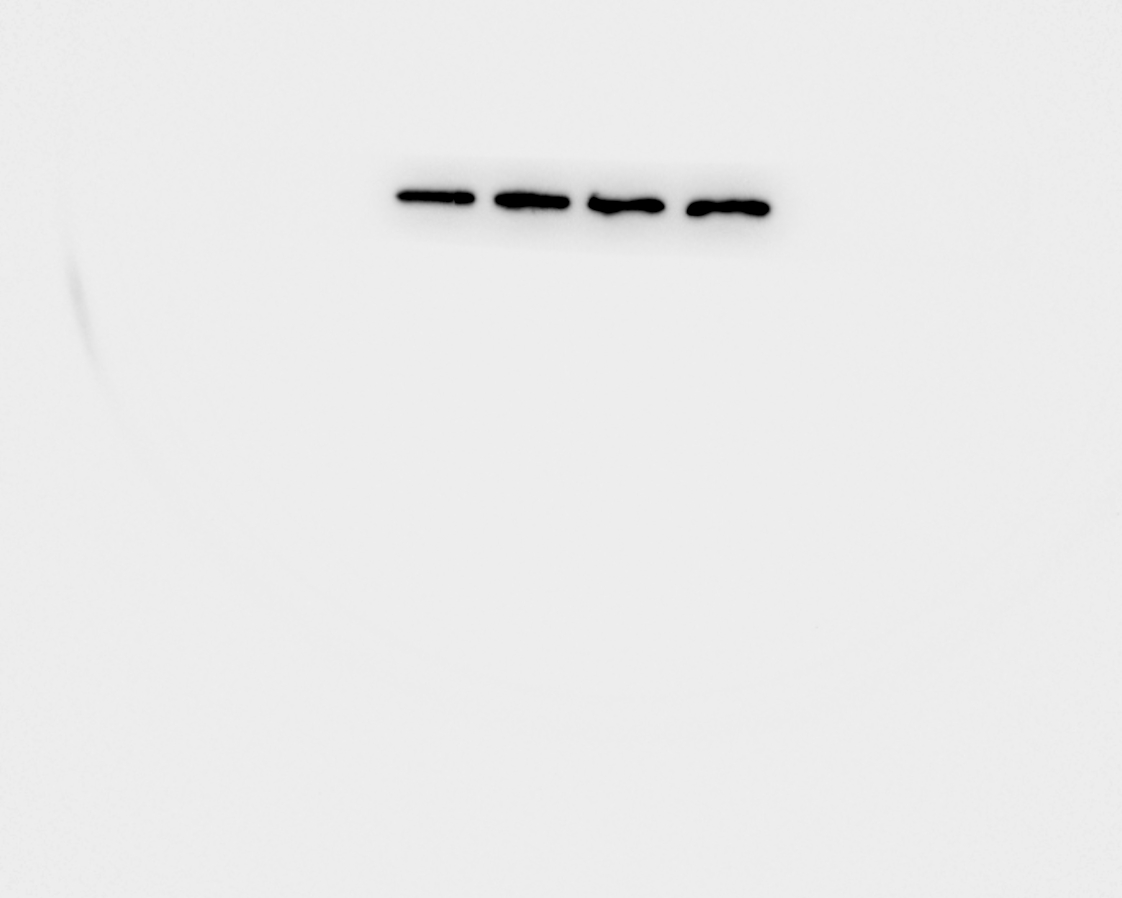

Supplement: Supplementary file 2 [file DataSheet3.ZIP › pictures of WB/2020-08-10 gapdh(Chemiluminescence).jpg]

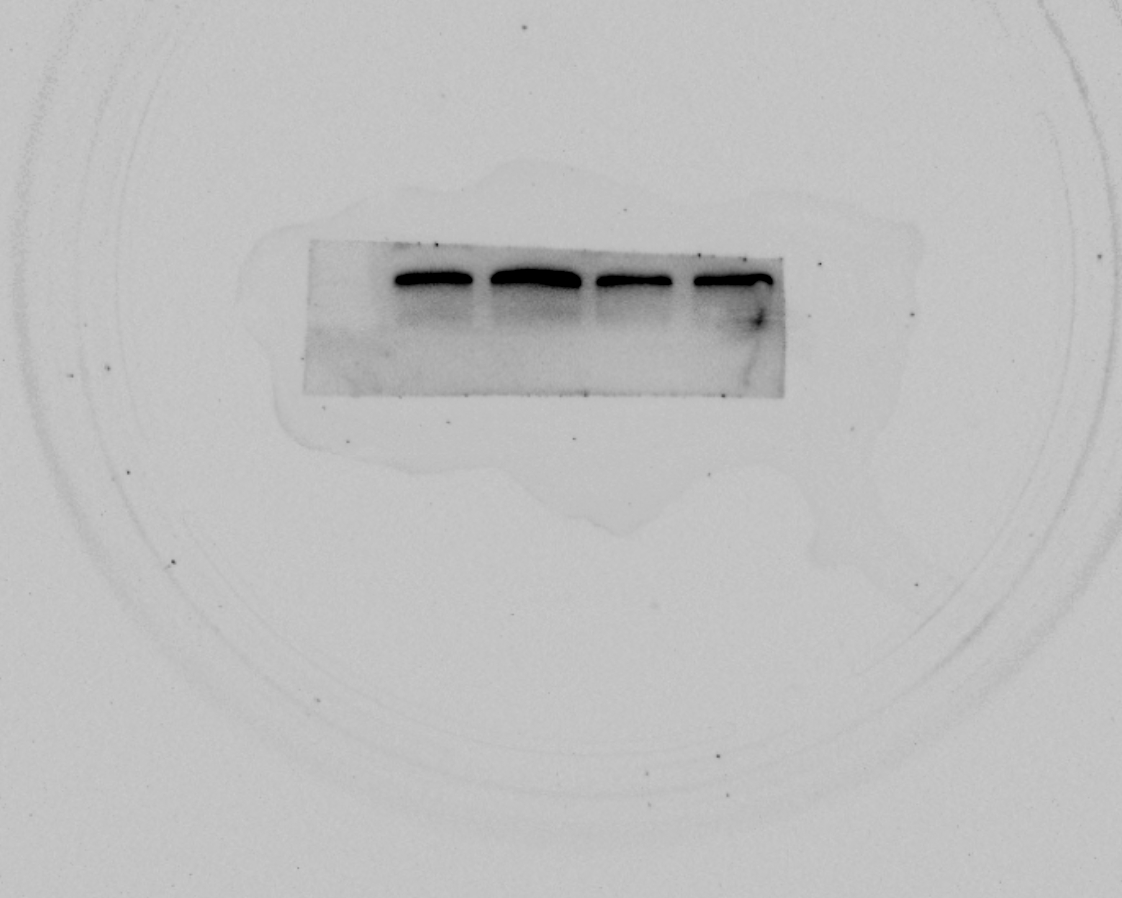

Supplement: Supplementary file 2 [file DataSheet3.ZIP › pictures of WB/2020-08-19 p62(Chemiluminescence).jpg]

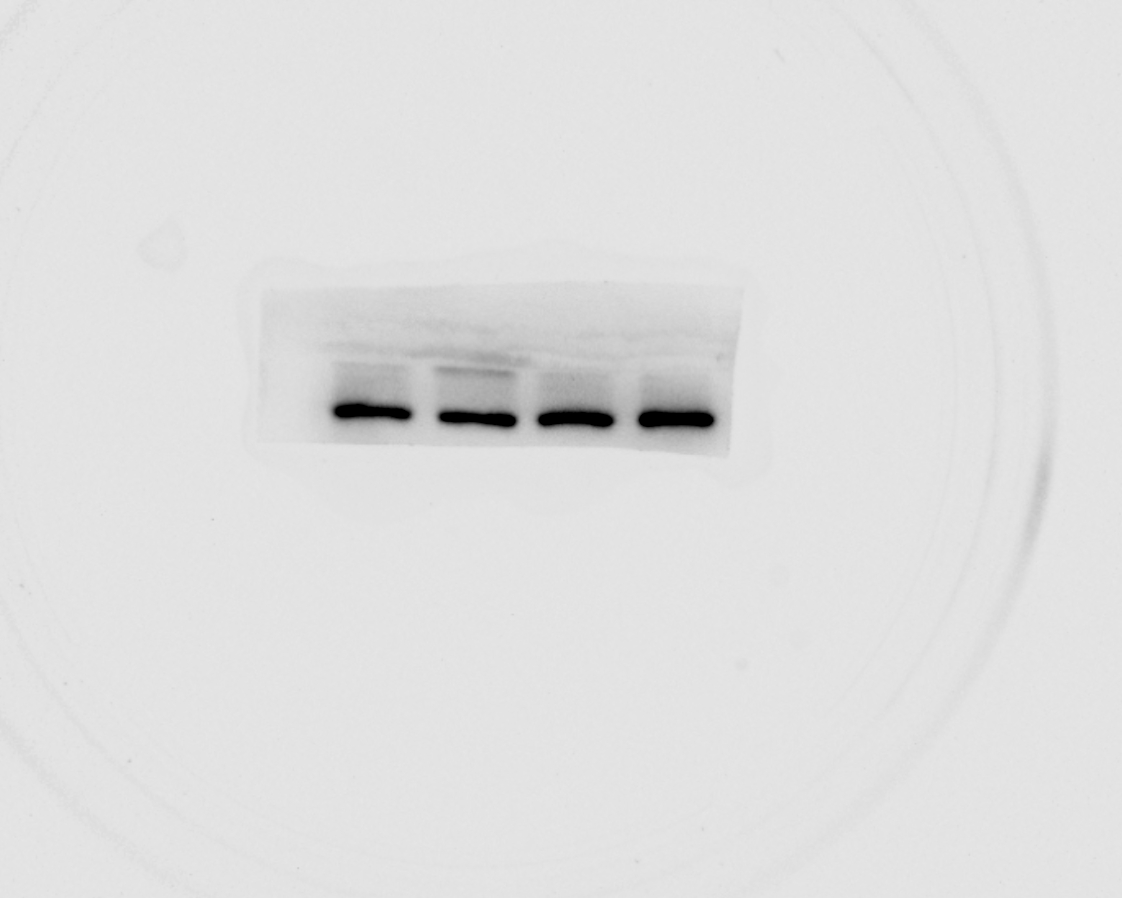

Supplement: Supplementary file 2 [file DataSheet3.ZIP › pictures of WB/2020-08-19 Rab 10(Chemiluminescence).jpg]

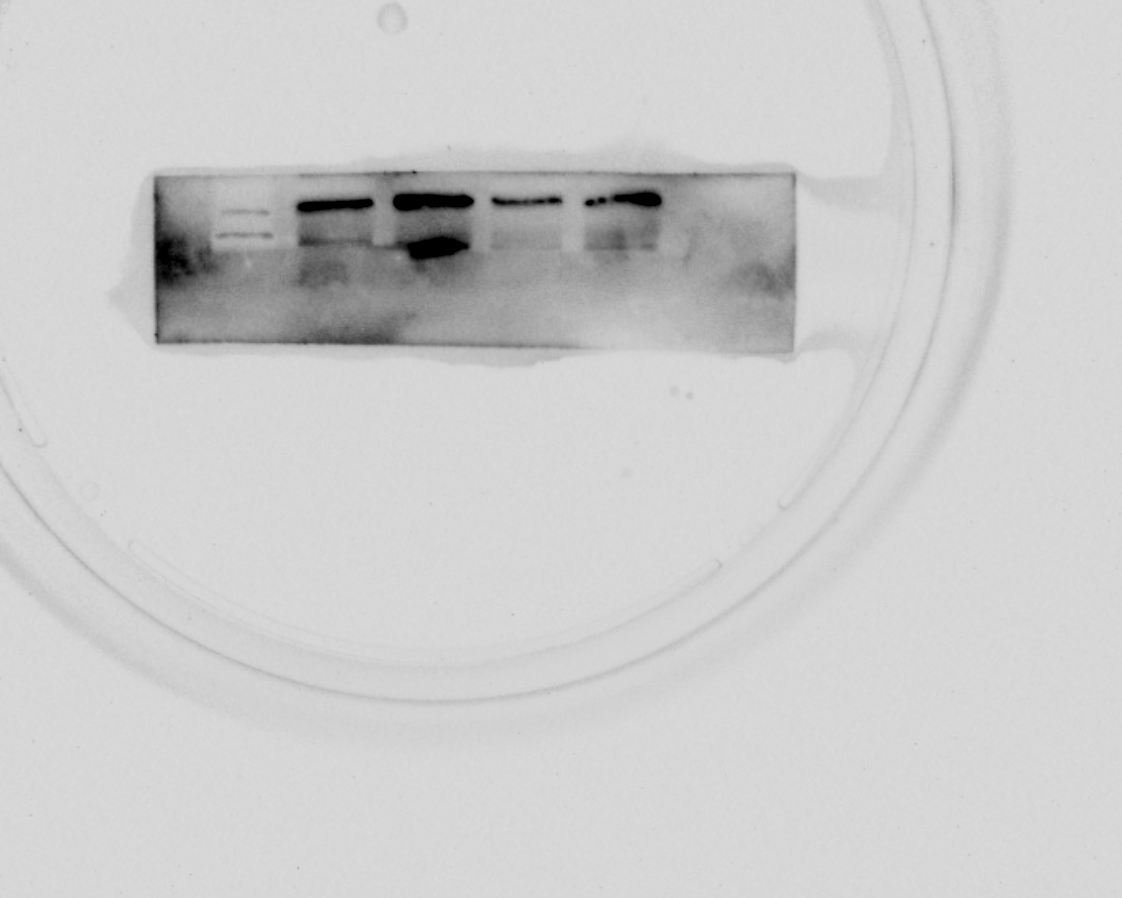

Supplement: Supplementary file 2 [file DataSheet3.ZIP › pictures of WB/2020-08-23 p62(Chemiluminescence).jpg]

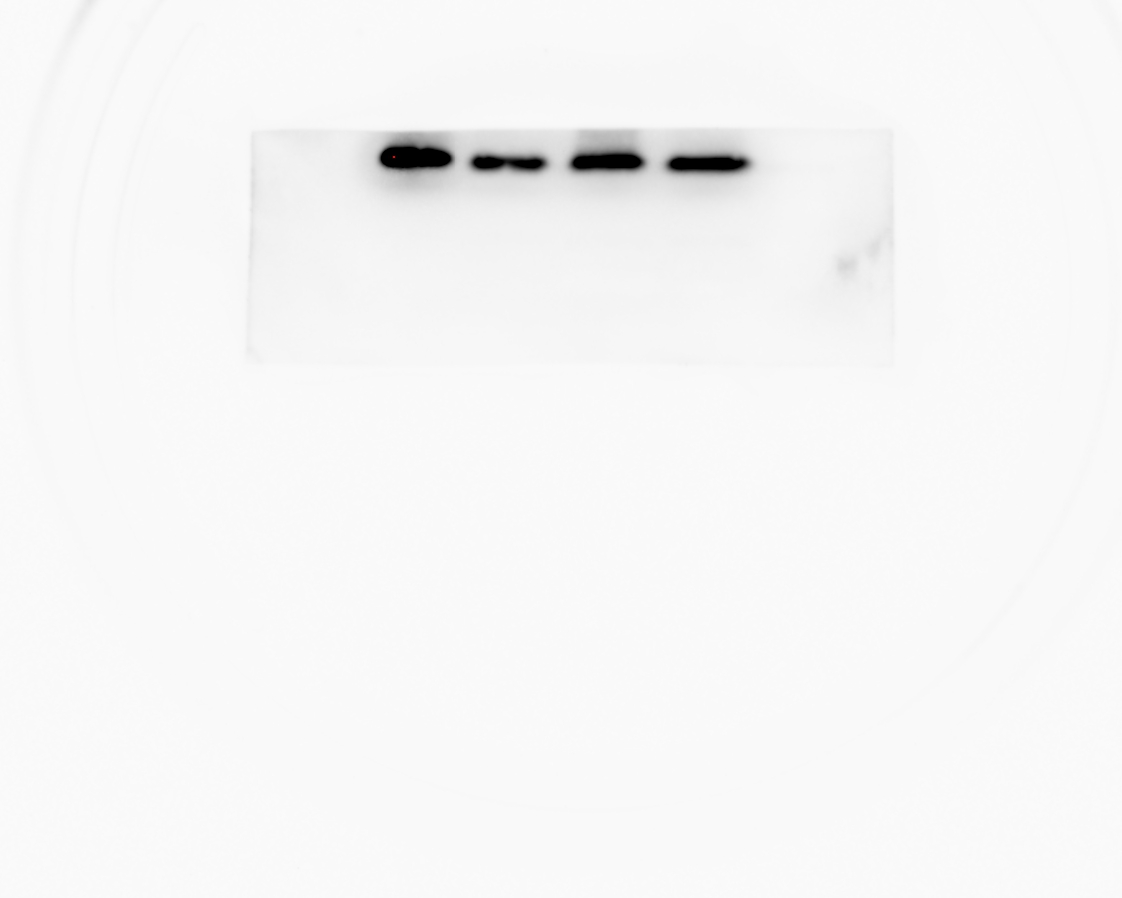

Supplement: Supplementary file 2 [file DataSheet3.ZIP › pictures of WB/2020-08-23 Rab10(Chemiluminescence).jpg]

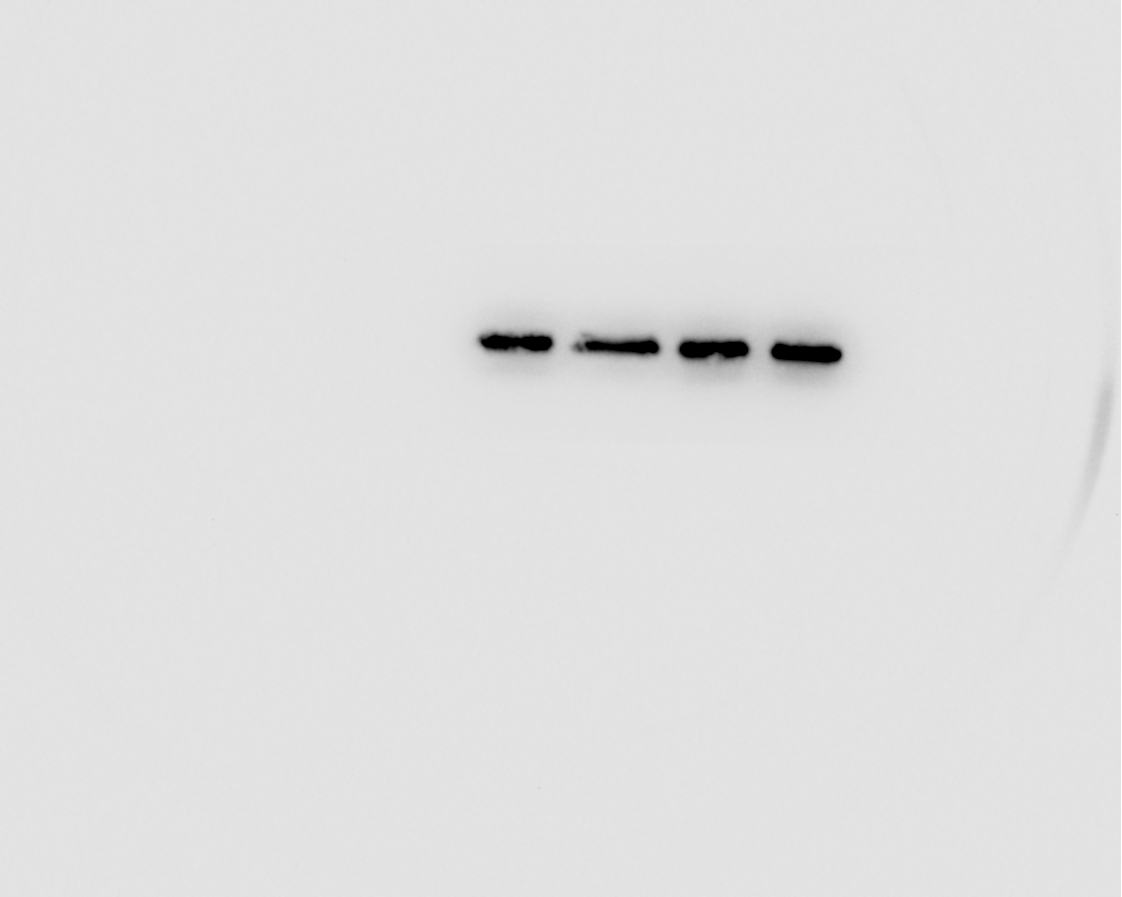

Supplement: Supplementary file 2 [file DataSheet3.ZIP › pictures of WB/2020-08-24 Rab10(Chemiluminescence).jpg]

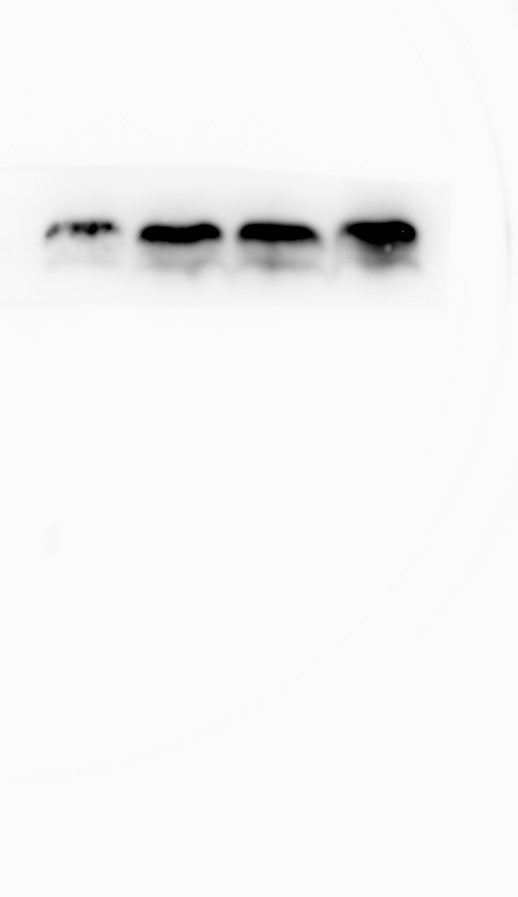

Supplement: Supplementary file 2 [file DataSheet3.ZIP › pictures of WB/2020-08-26 lc3(Chemiluminescence) .jpg]

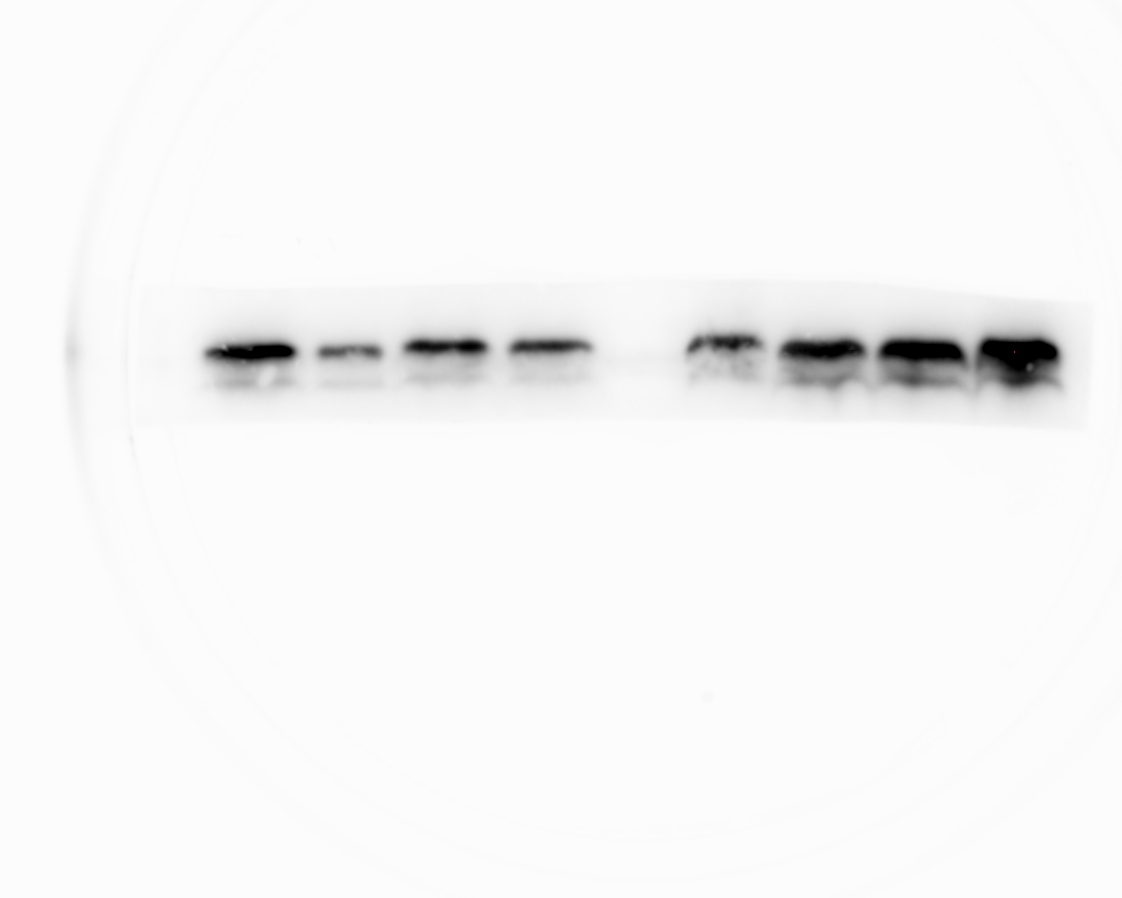

Supplement: Supplementary file 2 [file DataSheet3.ZIP › pictures of WB/2020-08-27 lc3(Chemiluminescence).jpg]

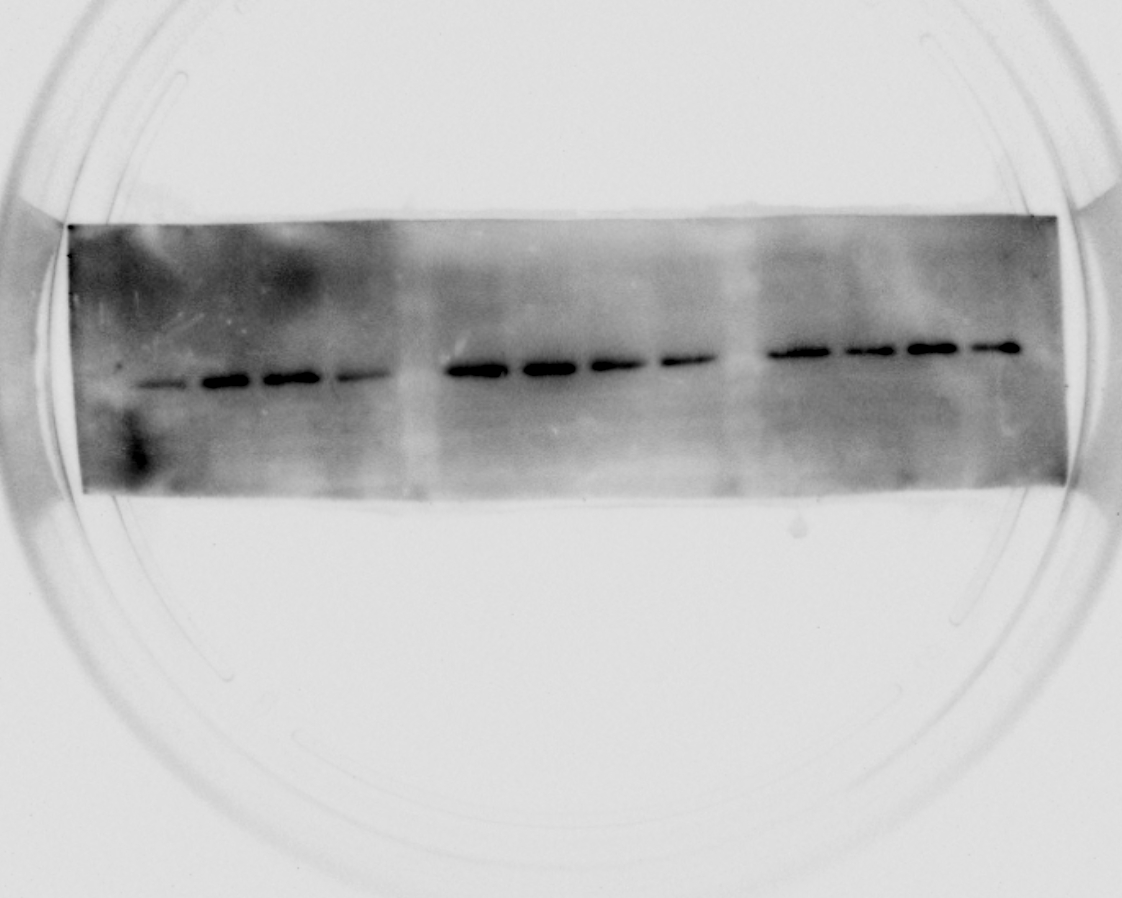

Supplement: Supplementary file 2 [file DataSheet3.ZIP › pictures of WB/2020-08-27 Rab8(Chemiluminescence).jpg]

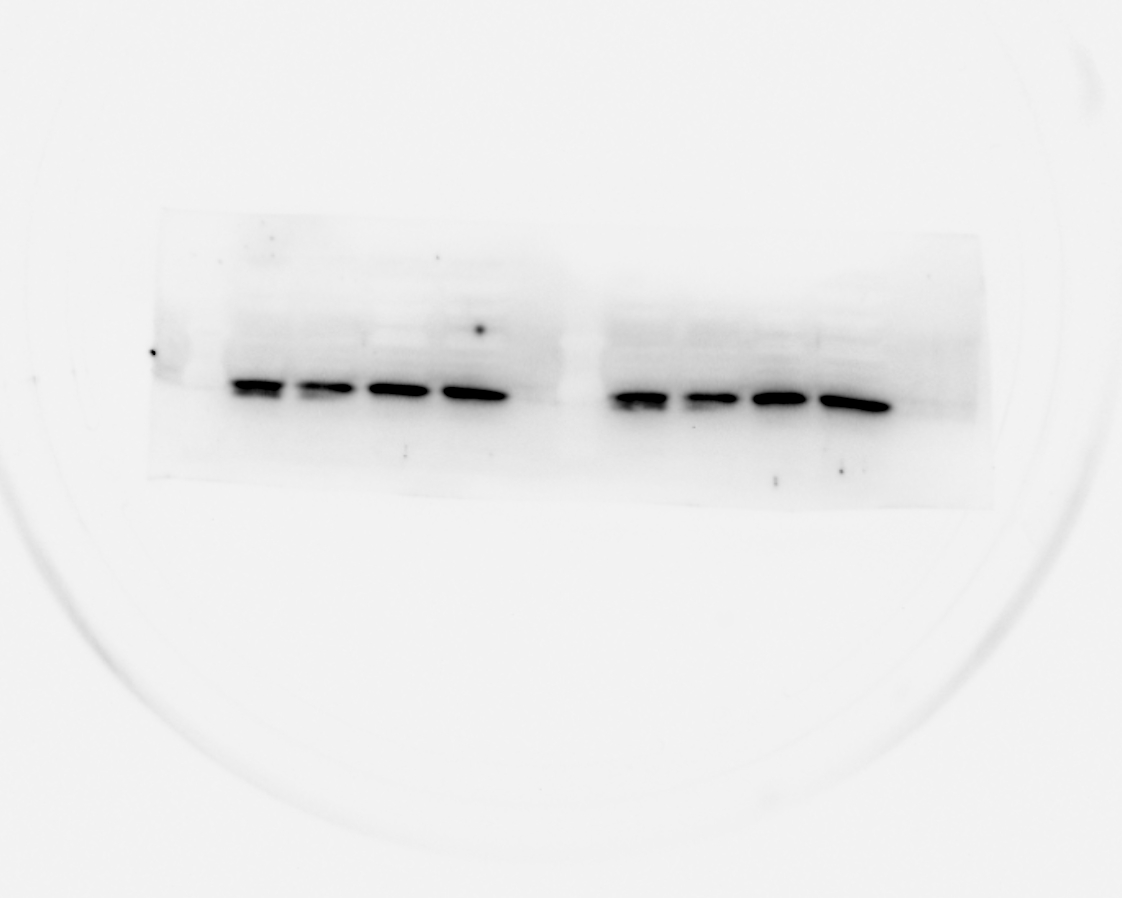

Supplement: Supplementary file 2 [file DataSheet3.ZIP › pictures of WB/2020-09-04 Rab8(Chemiluminescence).jpg]

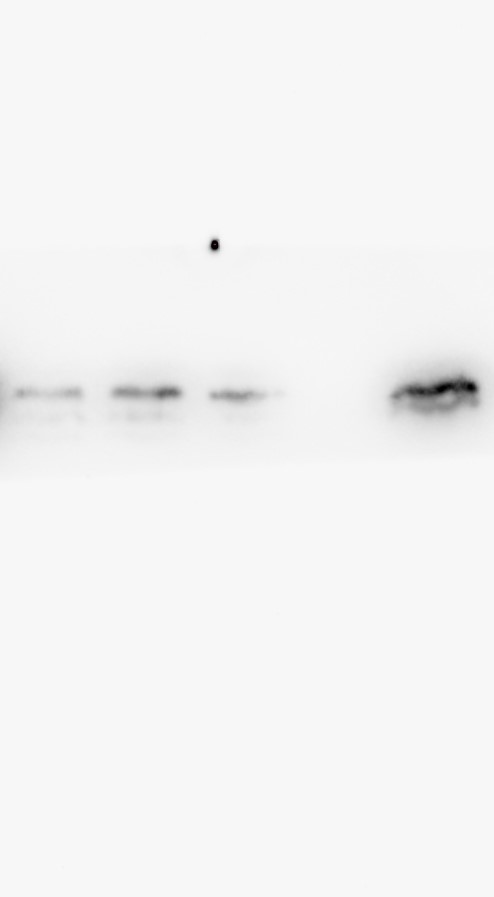

Supplement: Supplementary file 2 [file DataSheet3.ZIP › pictures of WB/2020-09-18 lc3 (Chemiluminescence).jpg]

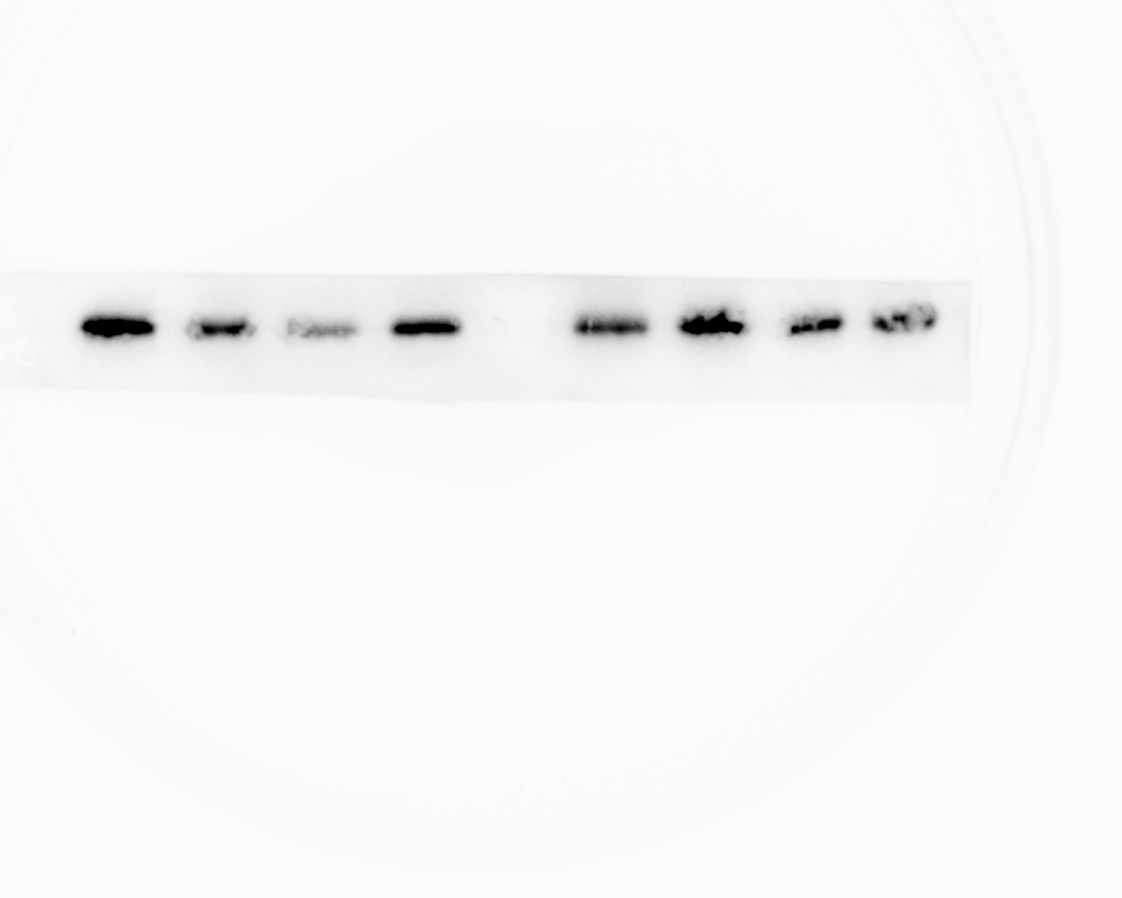

Supplement: Supplementary file 2 [file DataSheet3.ZIP › pictures of WB/2020-10-30 Rab10 2(Chemiluminescence).jpg]

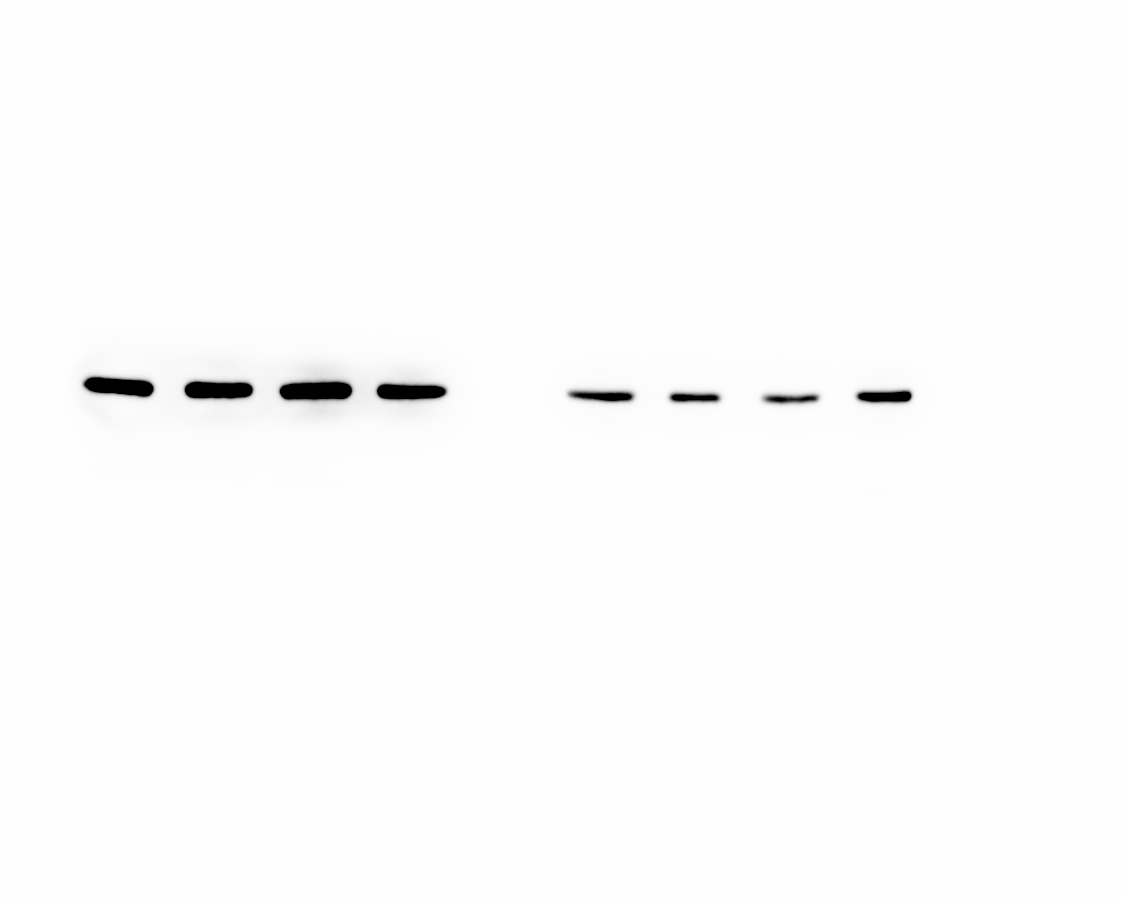

Supplement: Supplementary file 2 [file DataSheet3.ZIP › pictures of WB/2020-10-31 GAPDH 2(Chemiluminescence).jpg]

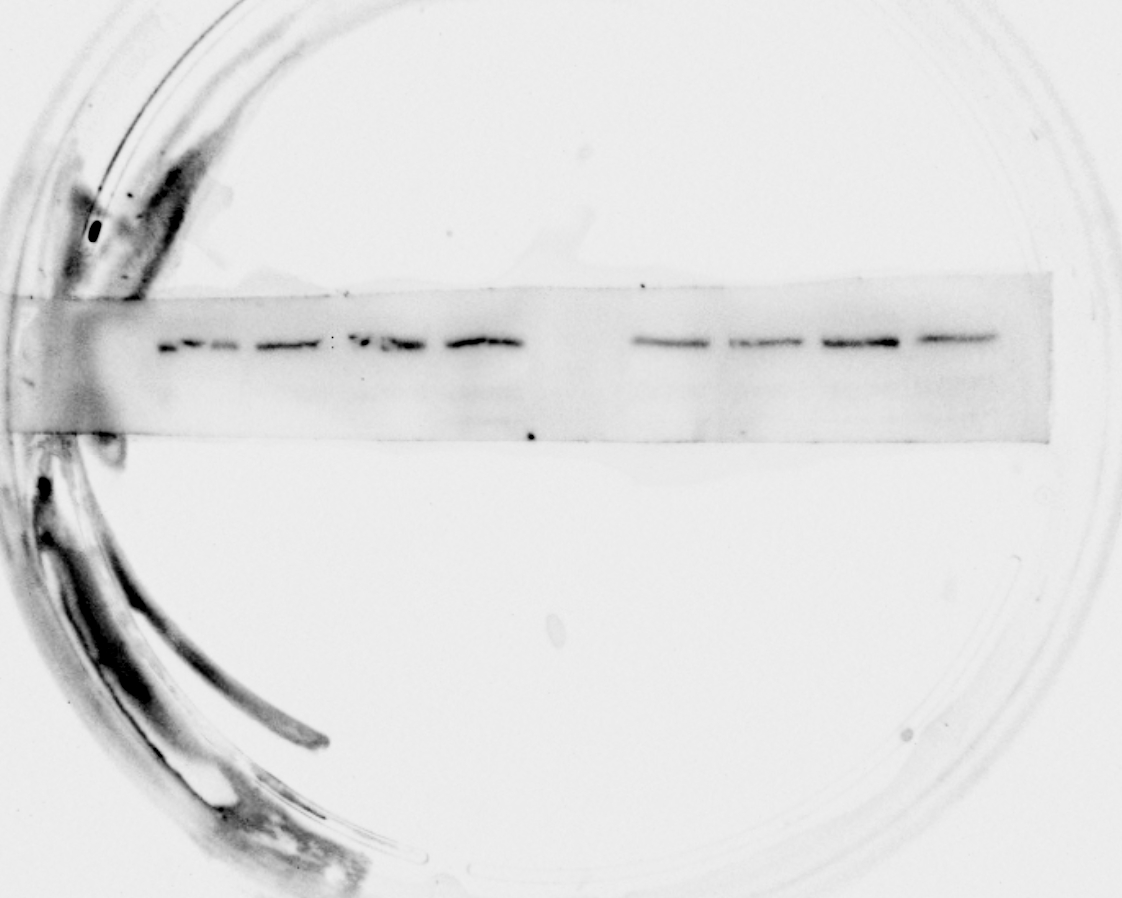

Supplement: Supplementary file 2 [file DataSheet3.ZIP › pictures of WB/2020-11-03 P62 2(Chemiluminescence).jpg]

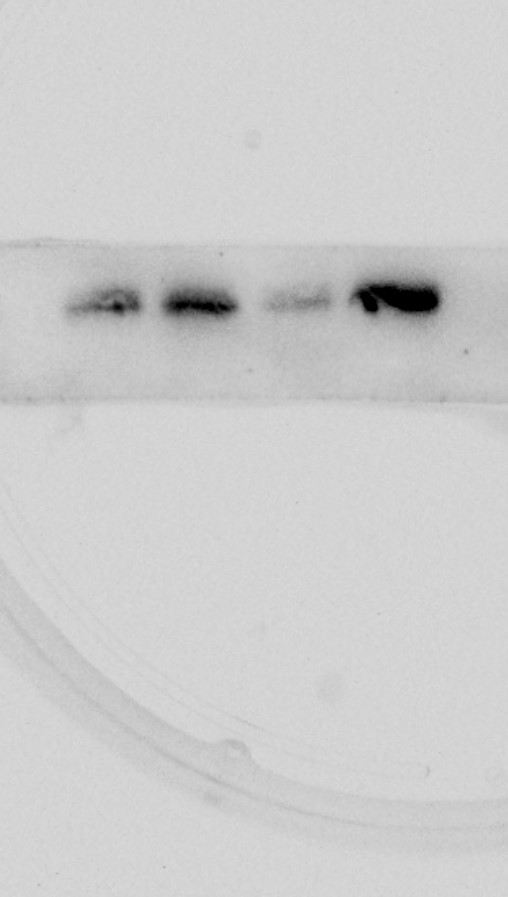

Supplement: Supplementary file 2 [file DataSheet3.ZIP › pictures of WB/2020-11-03 Rab10 2(Chemiluminescence).jpg]

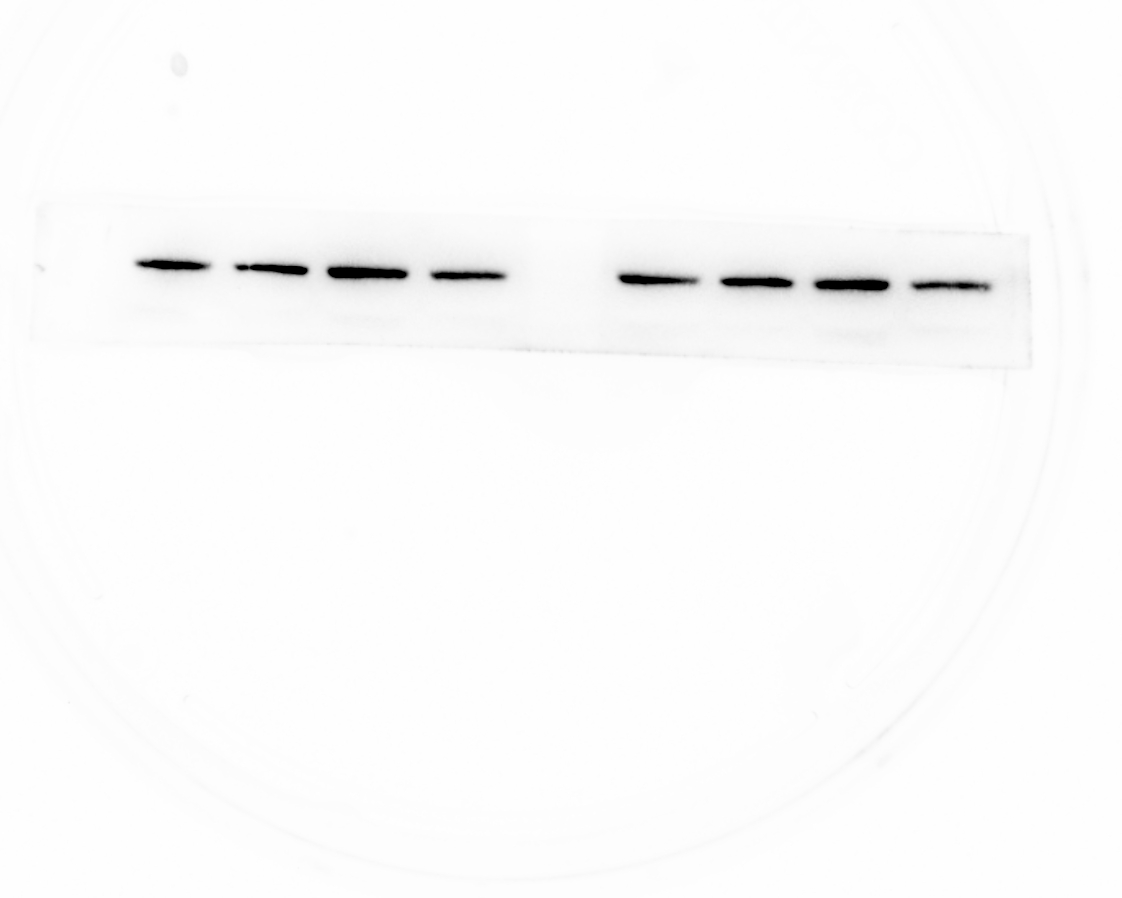

Supplement: Supplementary file 2 [file DataSheet3.ZIP › pictures of WB/2020-11-04 P62 2(Chemiluminescence).jpg]

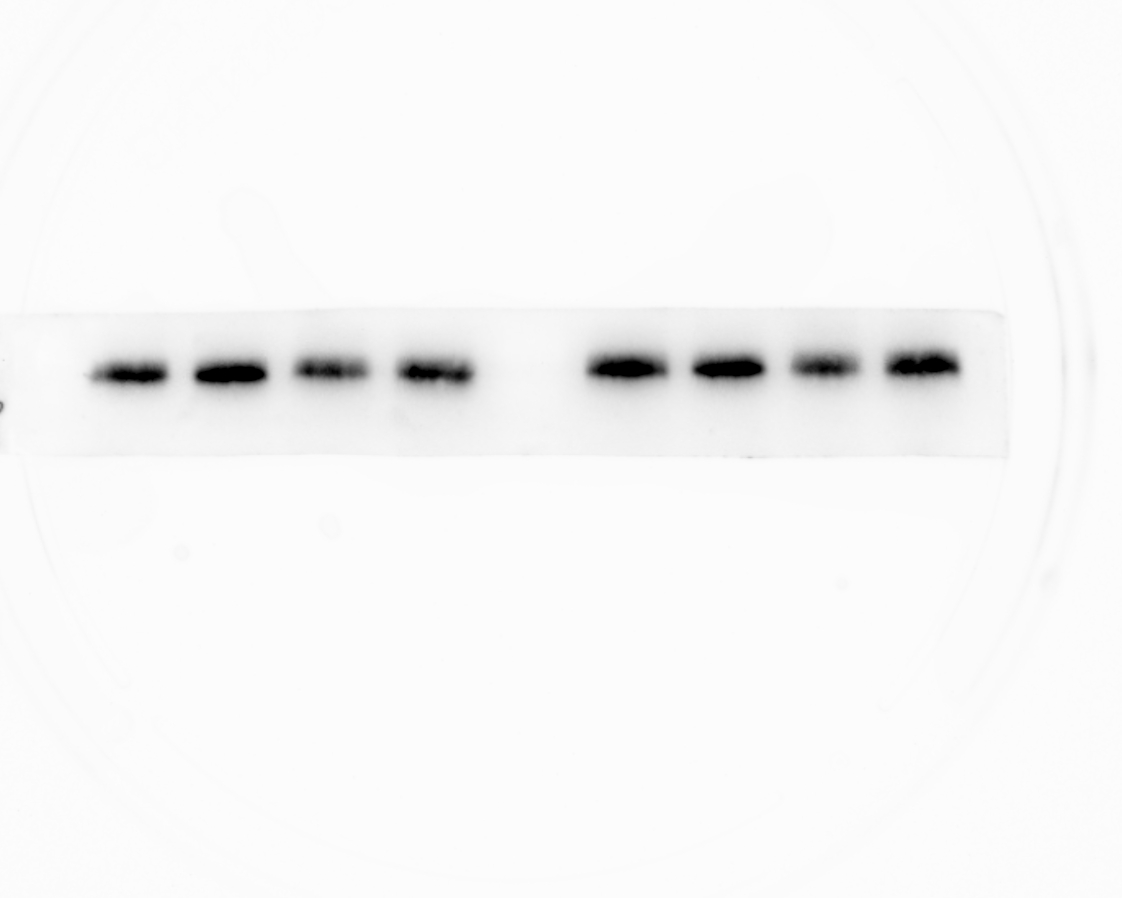

Supplement: Supplementary file 2 [file DataSheet3.ZIP › pictures of WB/2020-11-04 Rab10 2(Chemiluminescence).jpg]

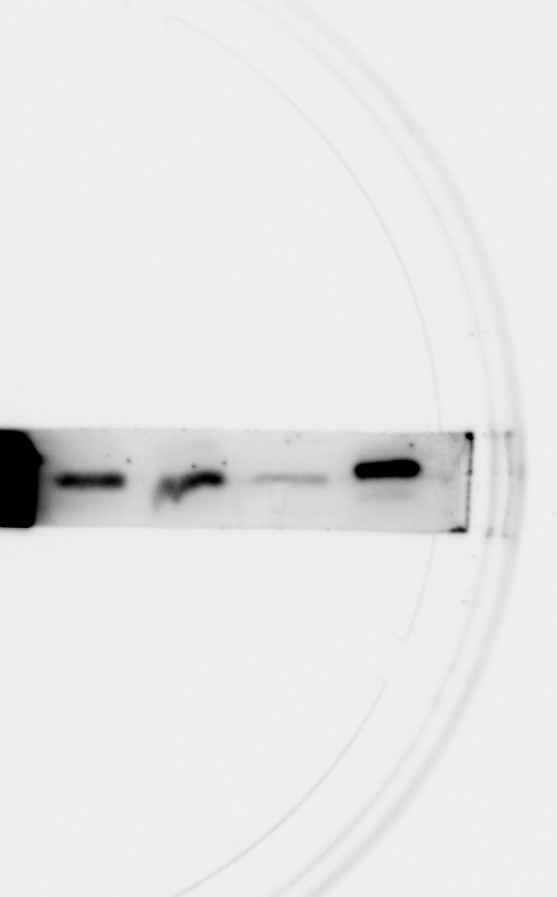

Supplement: Supplementary file 2 [file DataSheet3.ZIP › pictures of WB/2020-11-20 Rab8 2(Chemiluminescence).jpg]

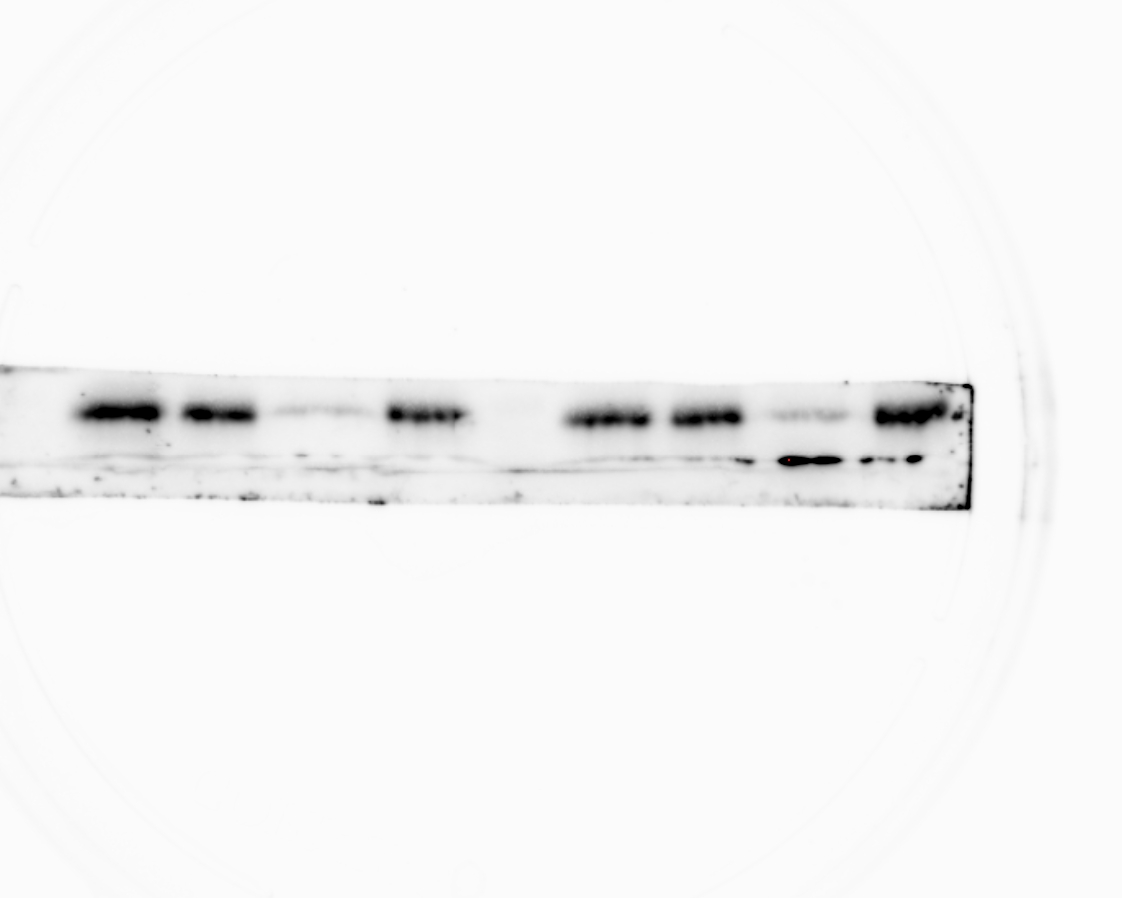

Supplement: Supplementary file 2 [file DataSheet3.ZIP › pictures of WB/2020-11-20 Rab8 2(Chemiluminescence).jpg]

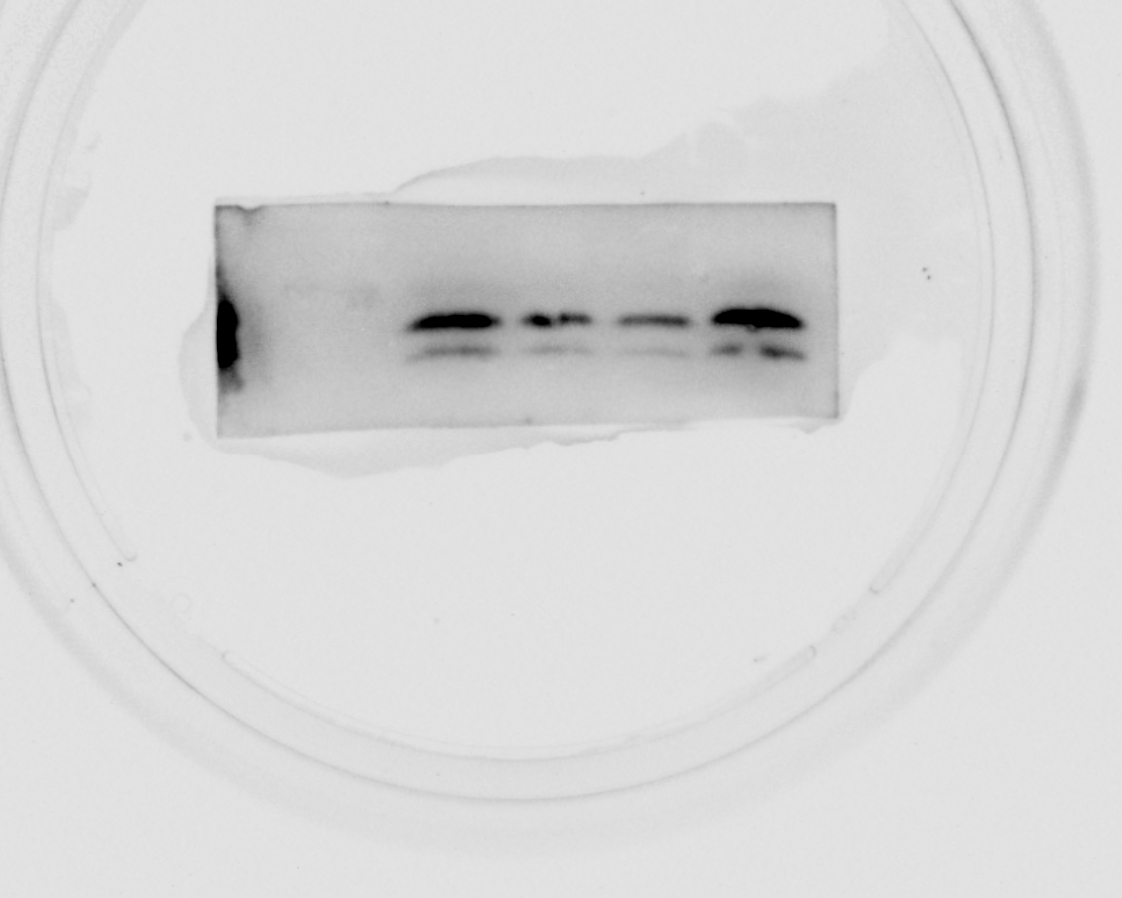

Supplement: Supplementary file 2 [file DataSheet3.ZIP › pictures of WB/2020-11-23 LC3 2(Chemiluminescence).jpg]

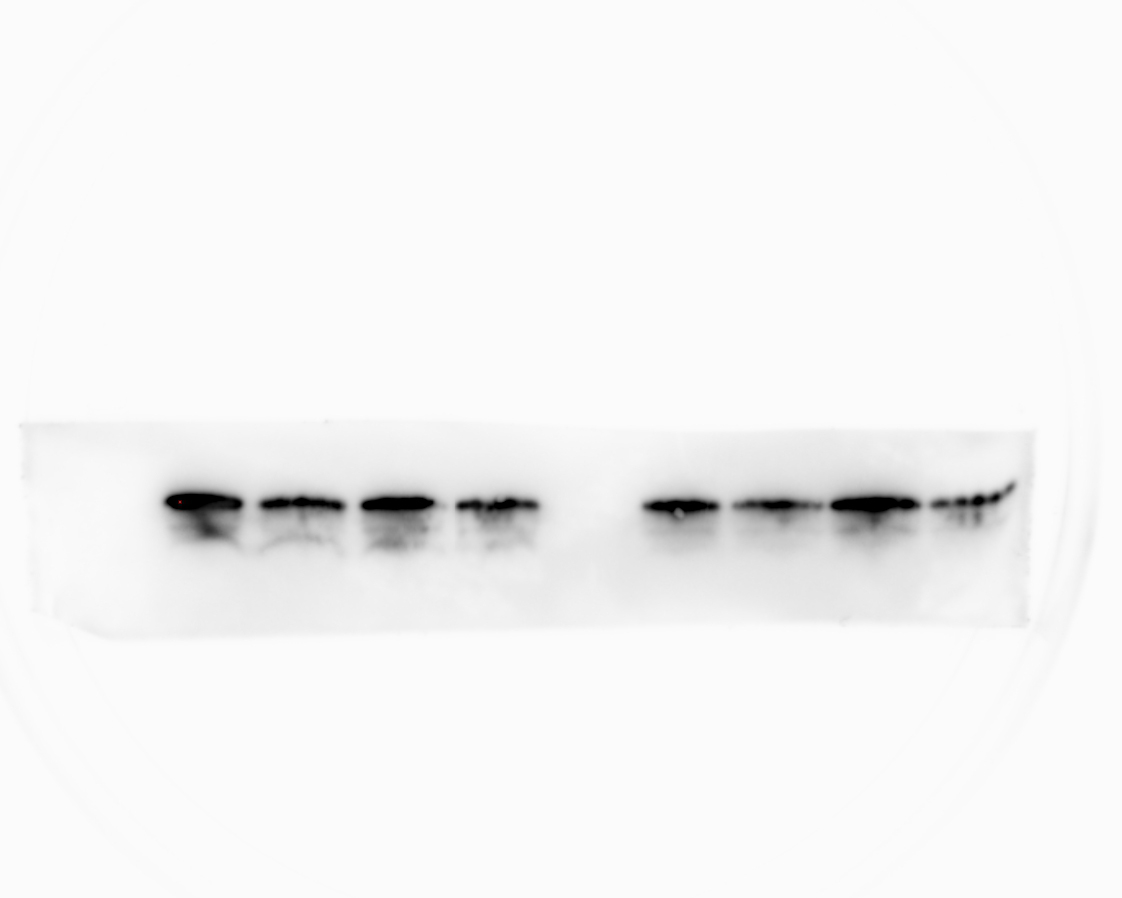

Supplement: Supplementary file 2 [file DataSheet3.ZIP › pictures of WB/2020-11-25 LC3(Chemiluminescence).jpg]

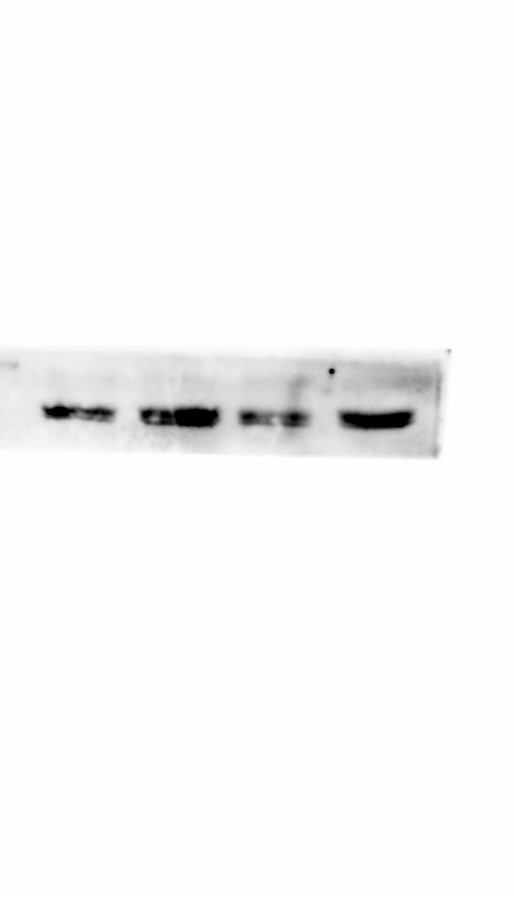

Supplement: Supplementary file 2 [file DataSheet3.ZIP › pictures of WB/2020-12-23 GLUT4 2(Chemiluminescence).jpg]

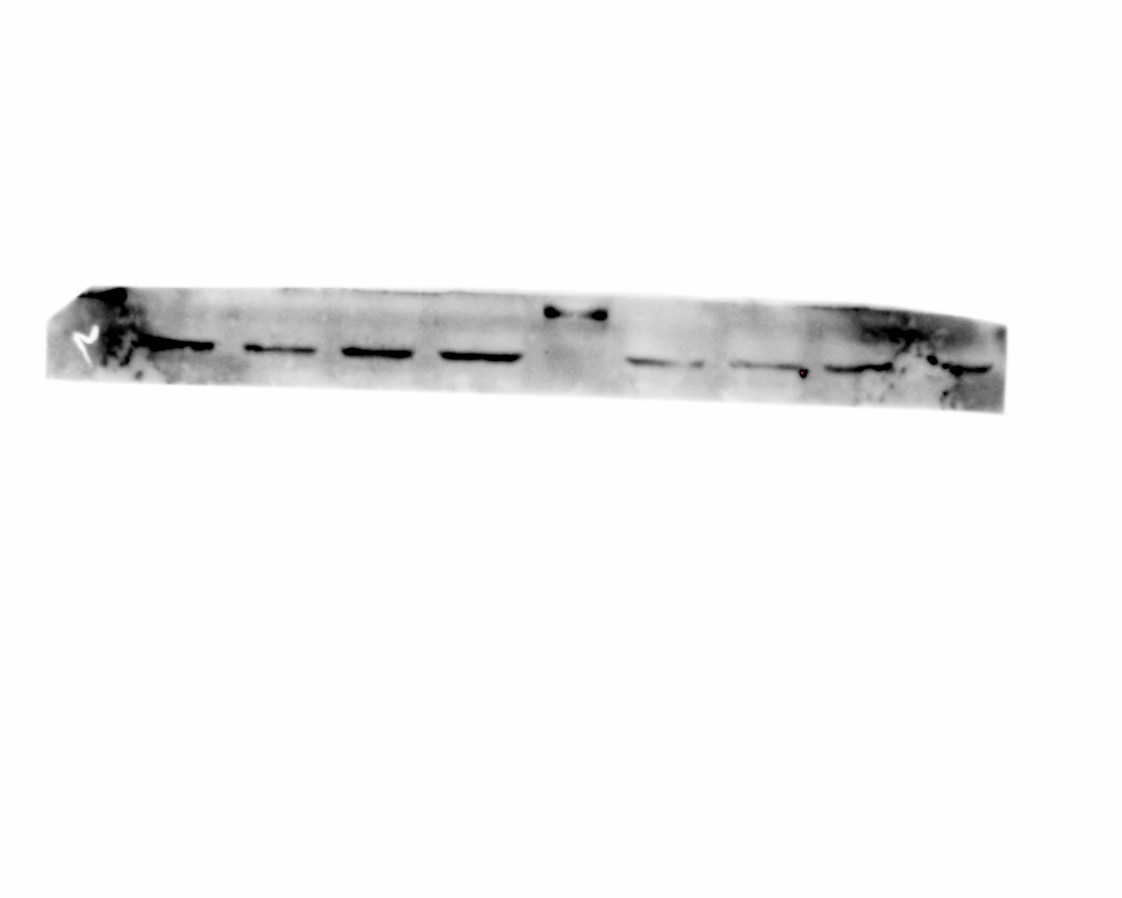

Supplement: Supplementary file 2 [file DataSheet3.ZIP › pictures of WB/2020-12-23 GLUT4 (Chemiluminescence).jpg]

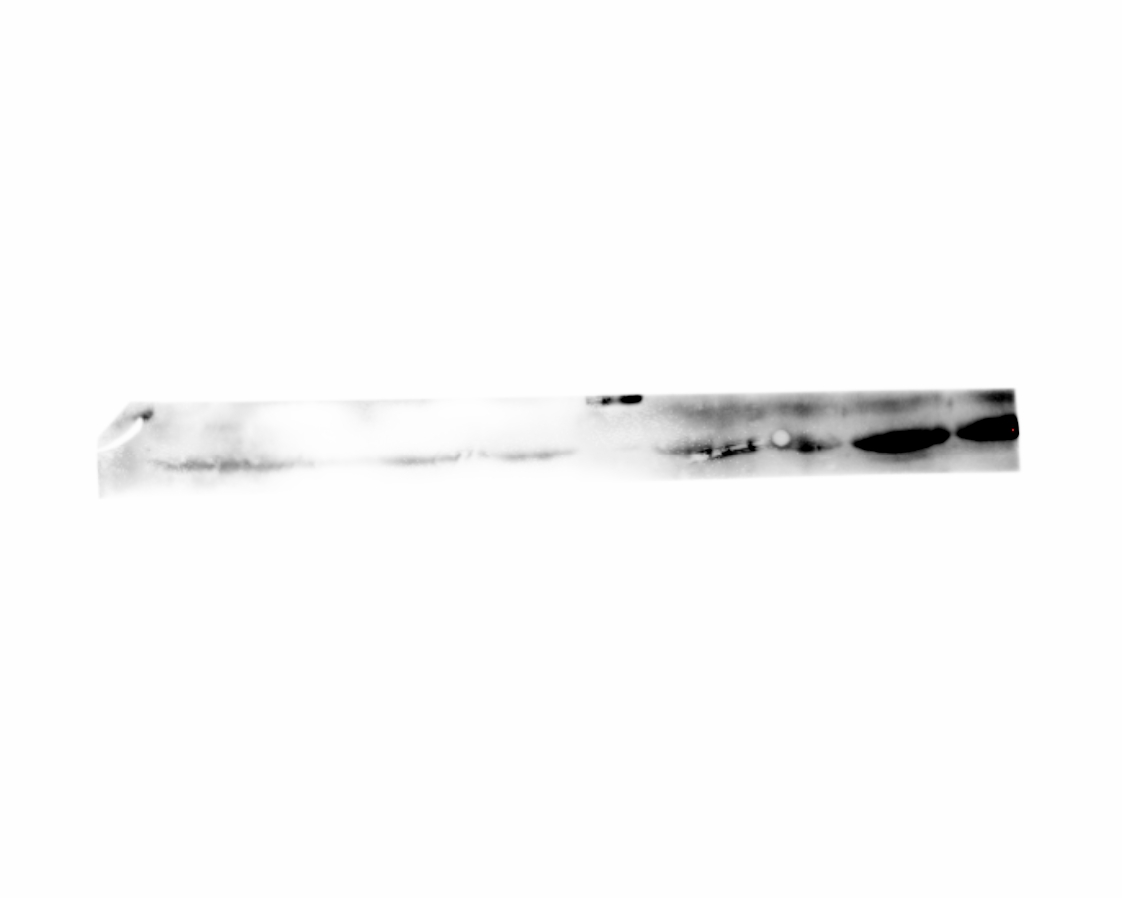

Supplement: Supplementary file 2 [file DataSheet3.ZIP › pictures of WB/2020-12-23 GLUT4(Chemiluminescence).jpg]

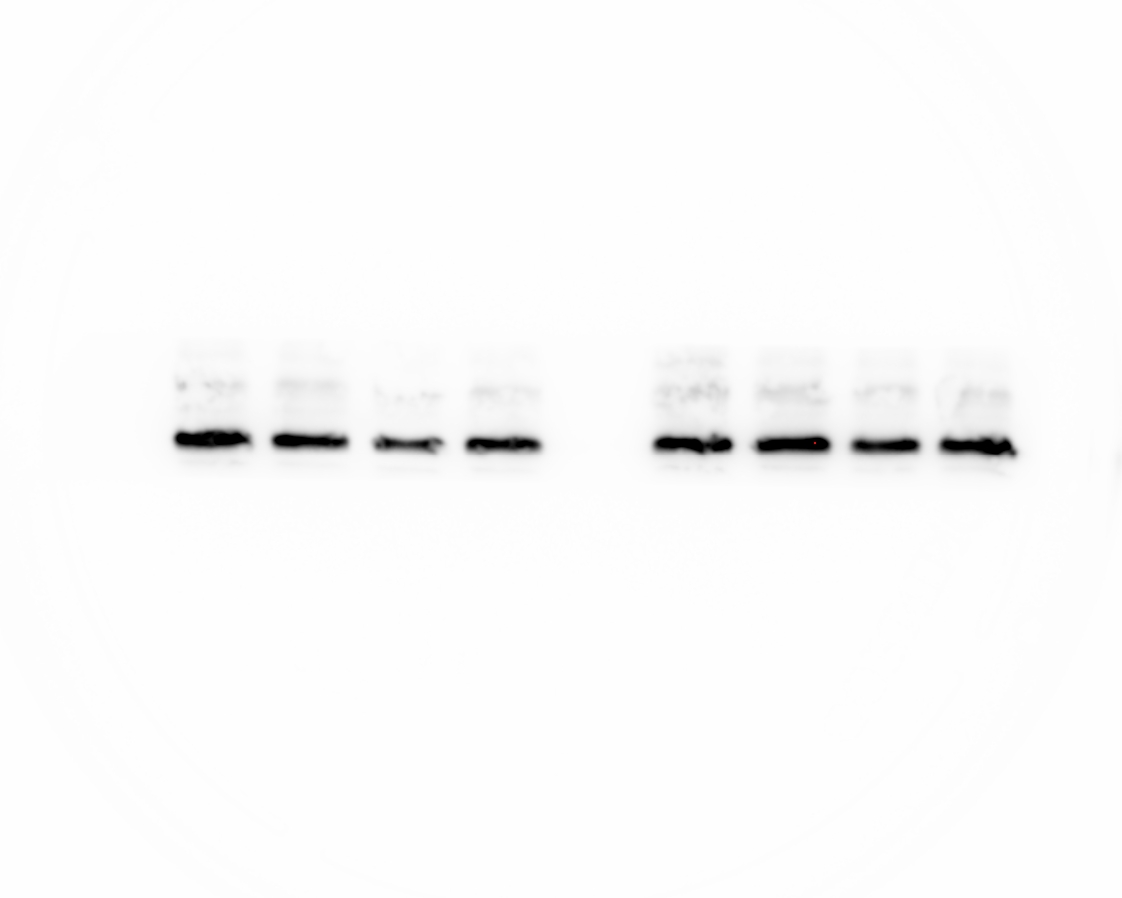

Supplement: Supplementary file 2 [file DataSheet3.ZIP › pictures of WB/2020-12-24 GLUT4 2(Chemiluminescence).jpg]

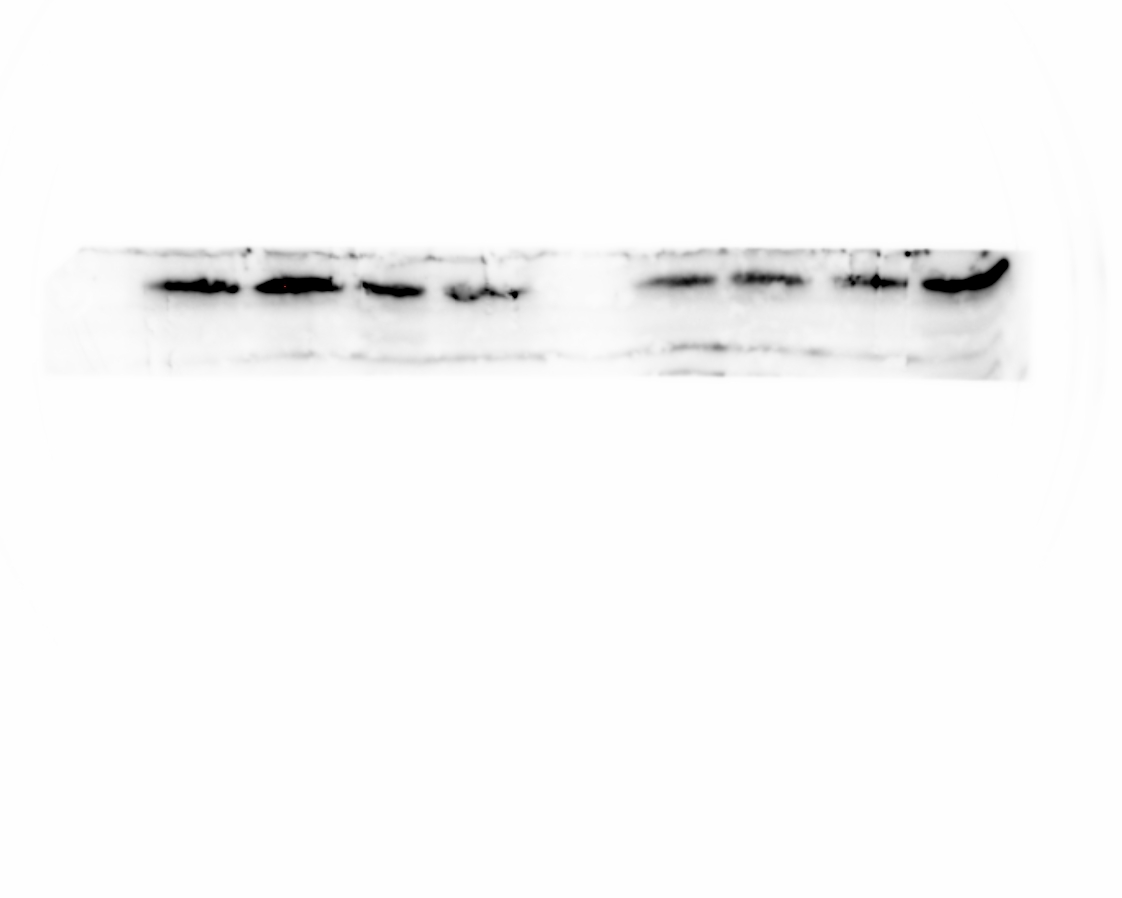

Supplement: Supplementary file 2 [file DataSheet3.ZIP › pictures of WB/2021-01-05 GLUT4 2(Chemiluminescence).jpg]

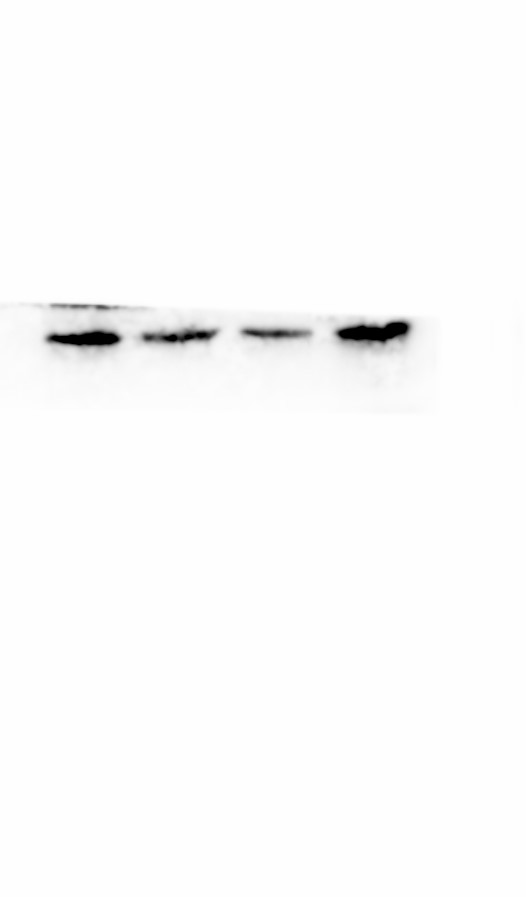

Supplement: Supplementary file 2 [file DataSheet3.ZIP › pictures of WB/2021-01-06 GLUT4 2(Chemiluminescence).jpg]

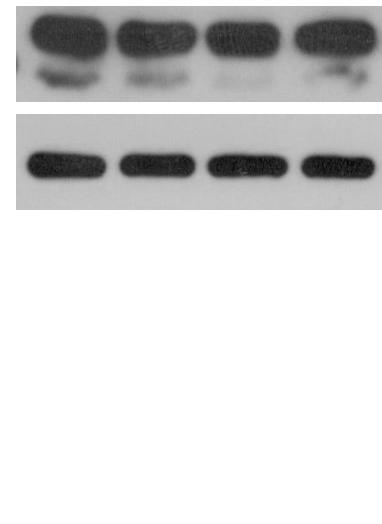

Supplement: Supplementary file 2 [file DataSheet3.ZIP › pictures of WB/2021-01-23 LC3 2(Chemiluminescence).jpg]

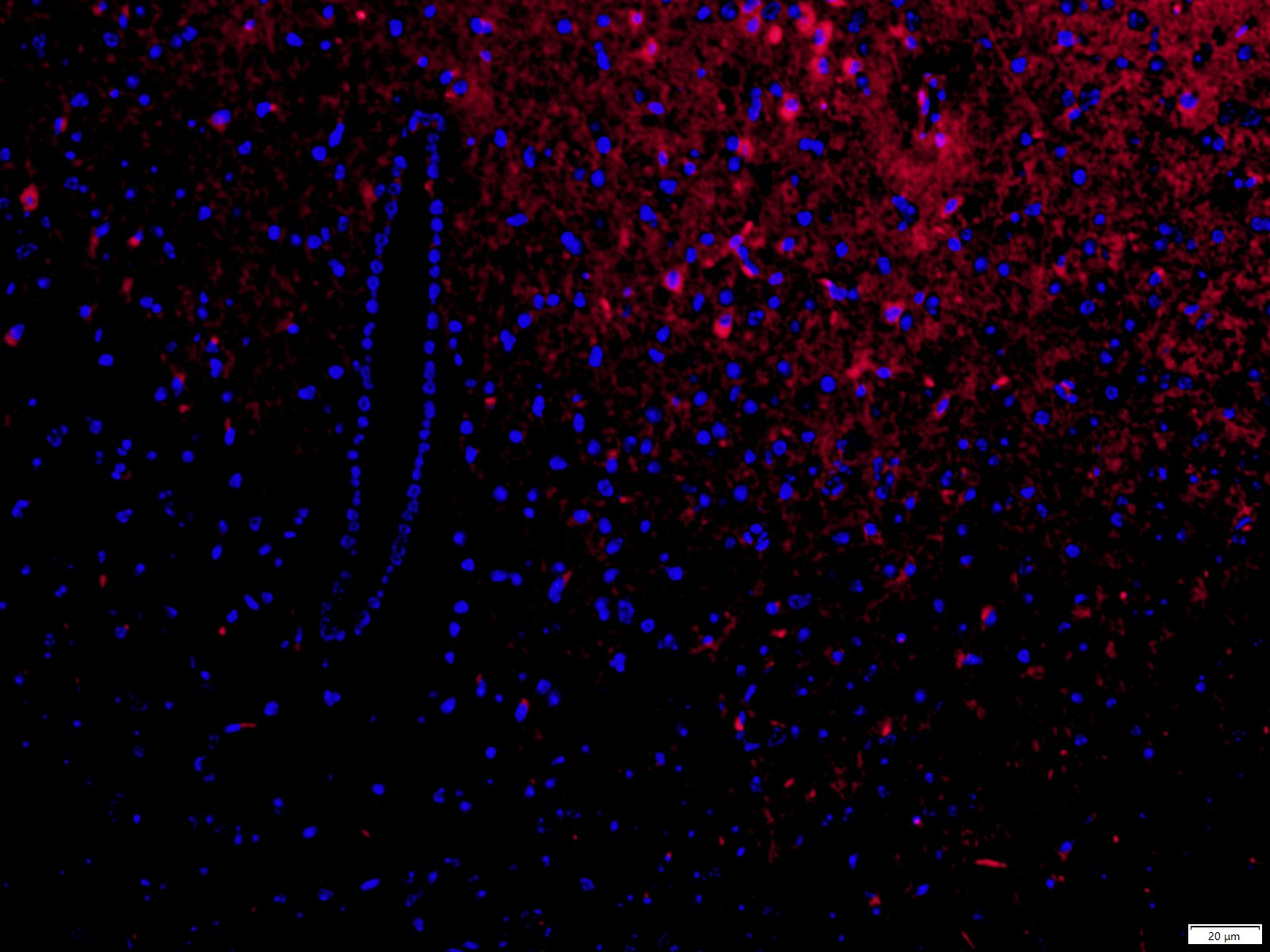

Supplement: Supplementary file 6 [file DataSheet2.ZIP › immunofluorescence of GLUT4/part 1 experiment/Control/C.jpg]

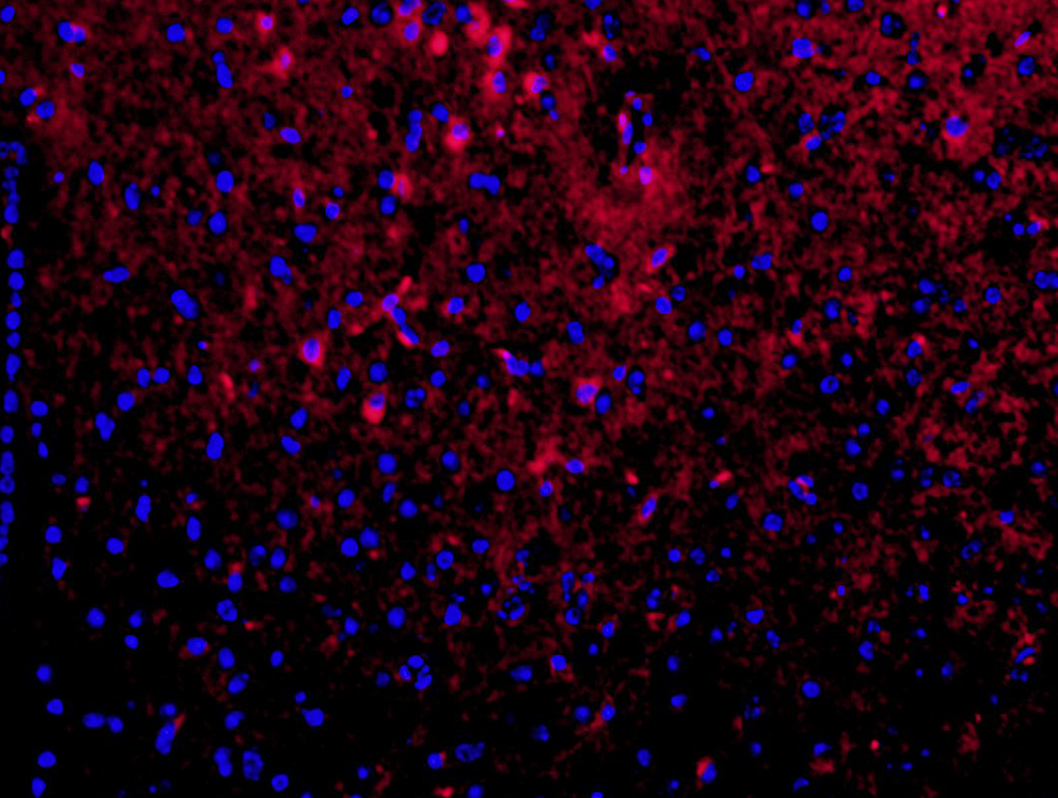

Supplement: Supplementary file 6 [file DataSheet2.ZIP › immunofluorescence of GLUT4/part 1 experiment/Control/C-1.jpg]

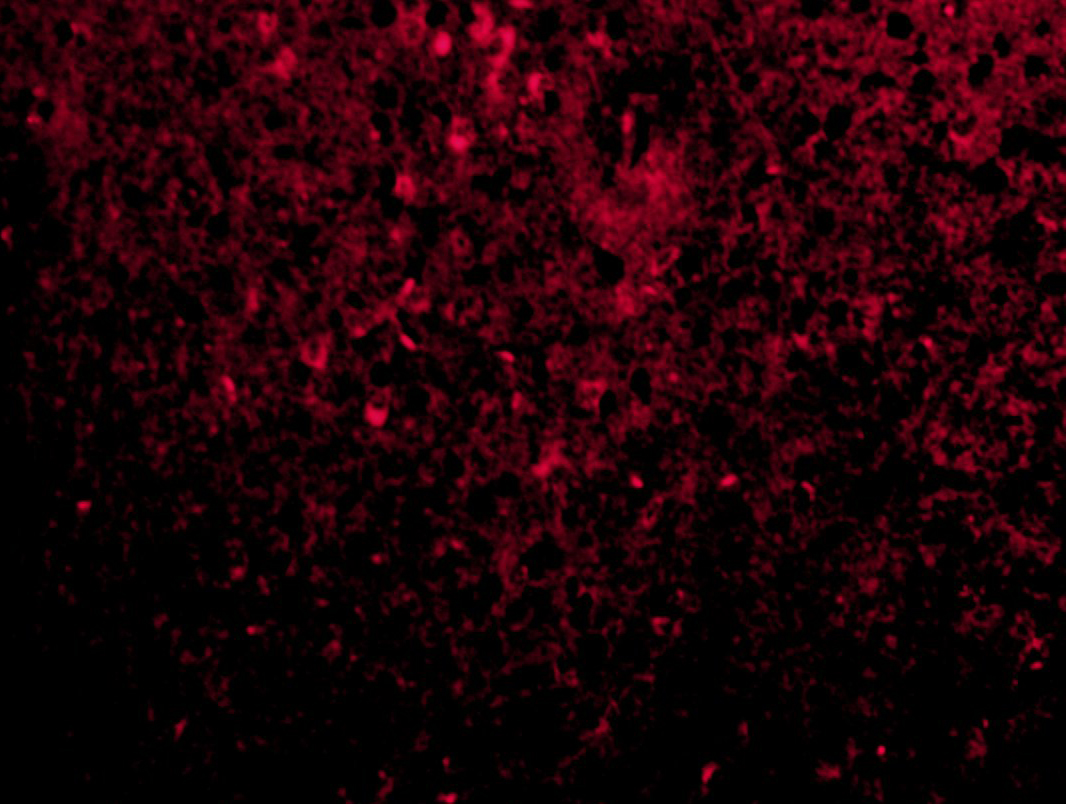

Supplement: Supplementary file 6 [file DataSheet2.ZIP › immunofluorescence of GLUT4/part 1 experiment/Control/C-2.jpg]

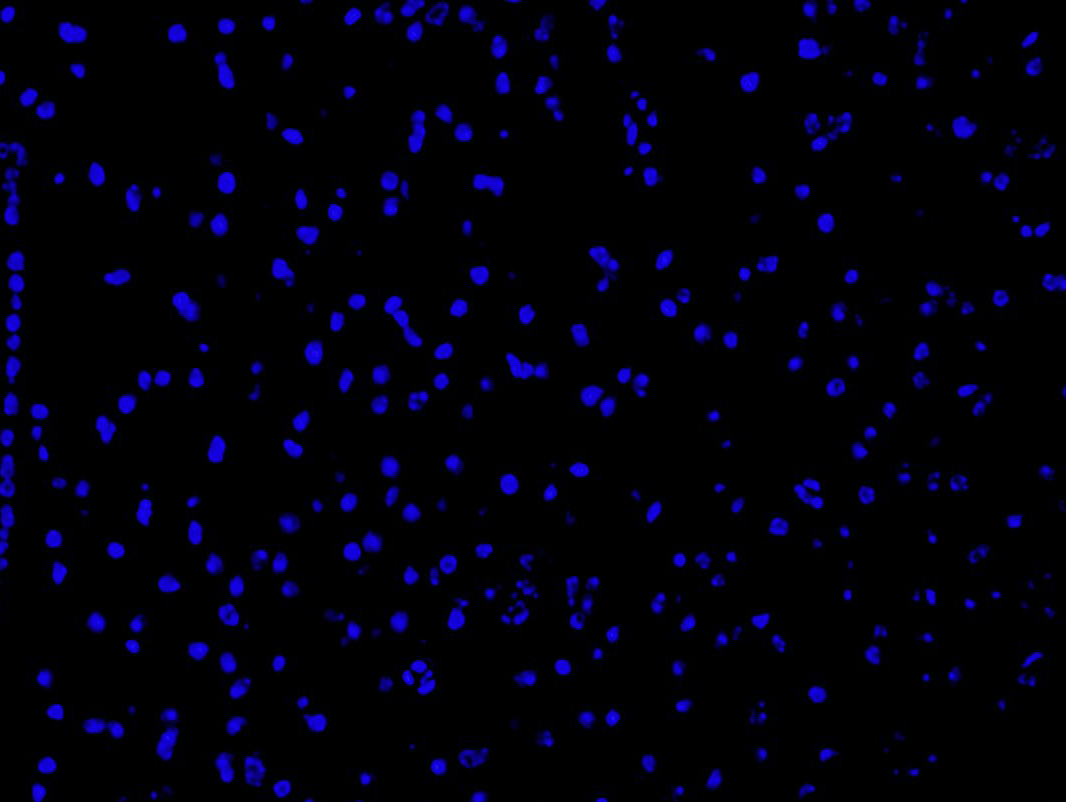

Supplement: Supplementary file 6 [file DataSheet2.ZIP › immunofluorescence of GLUT4/part 1 experiment/Control/C-3.jpg]

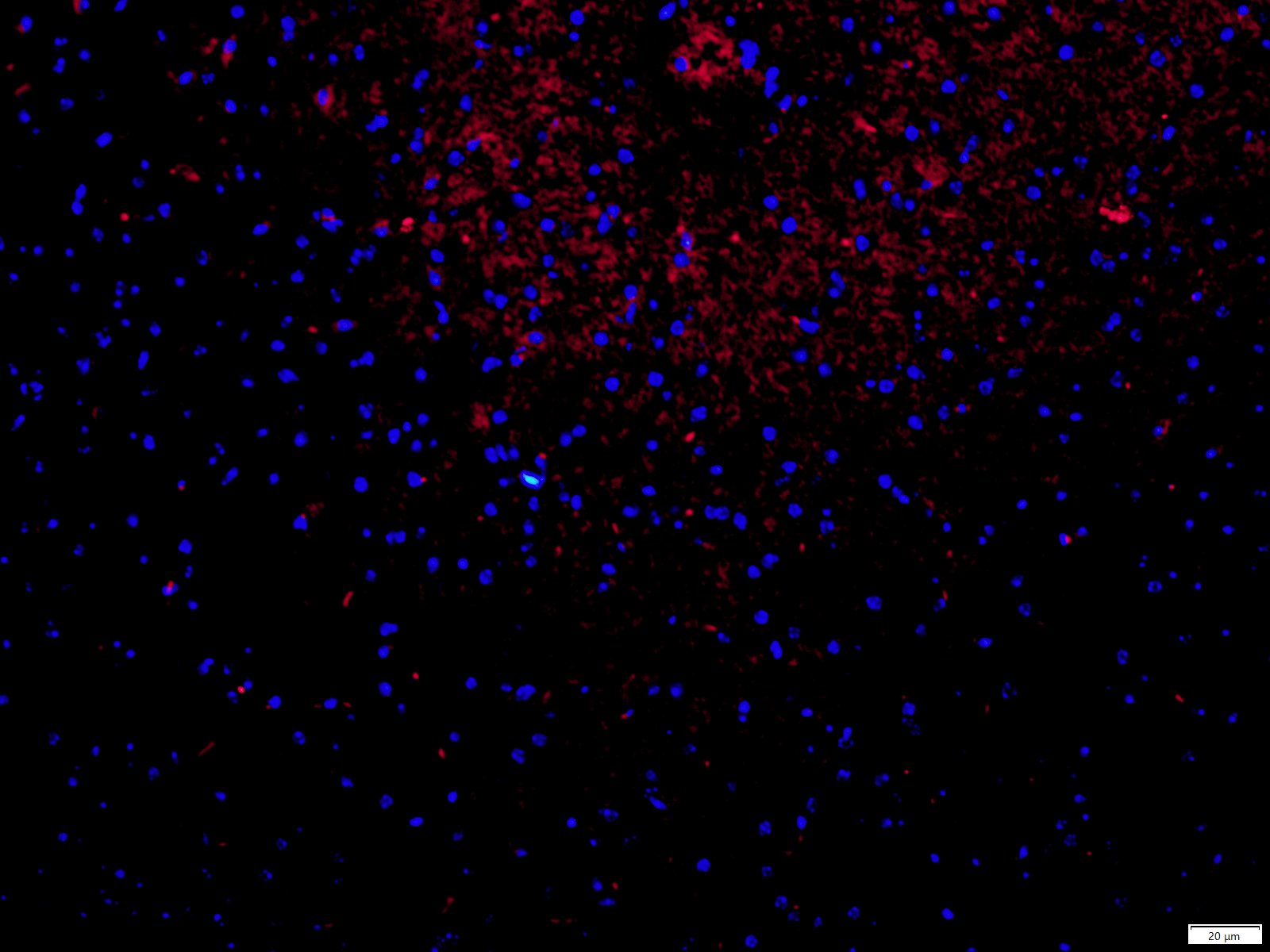

Supplement: Supplementary file 6 [file DataSheet2.ZIP › immunofluorescence of GLUT4/part 1 experiment/CUMS/M.jpg]

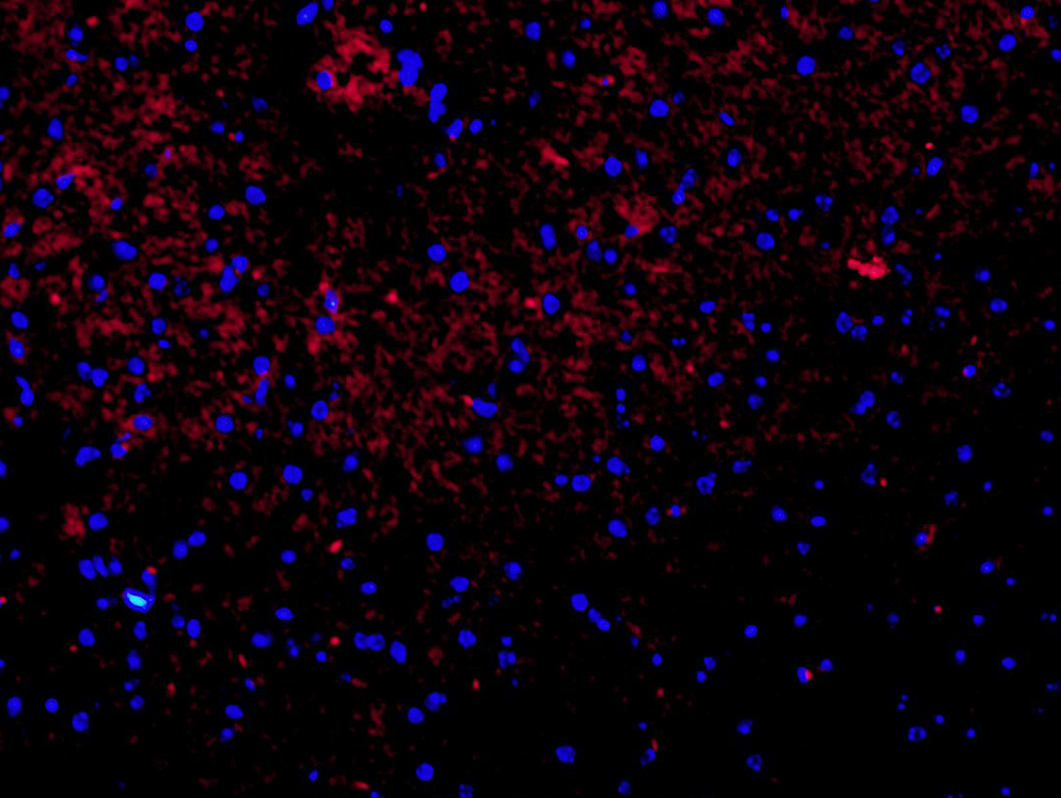

Supplement: Supplementary file 6 [file DataSheet2.ZIP › immunofluorescence of GLUT4/part 1 experiment/CUMS/M-1.jpg]

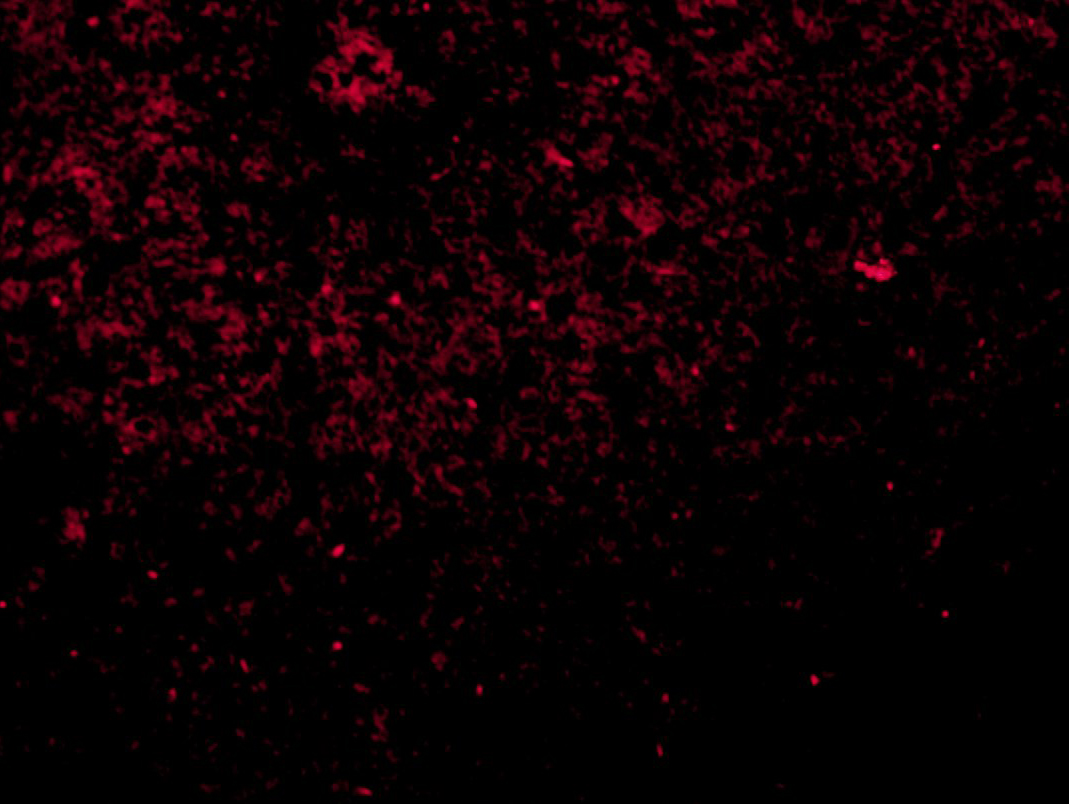

Supplement: Supplementary file 6 [file DataSheet2.ZIP › immunofluorescence of GLUT4/part 1 experiment/CUMS/M-2.jpg]

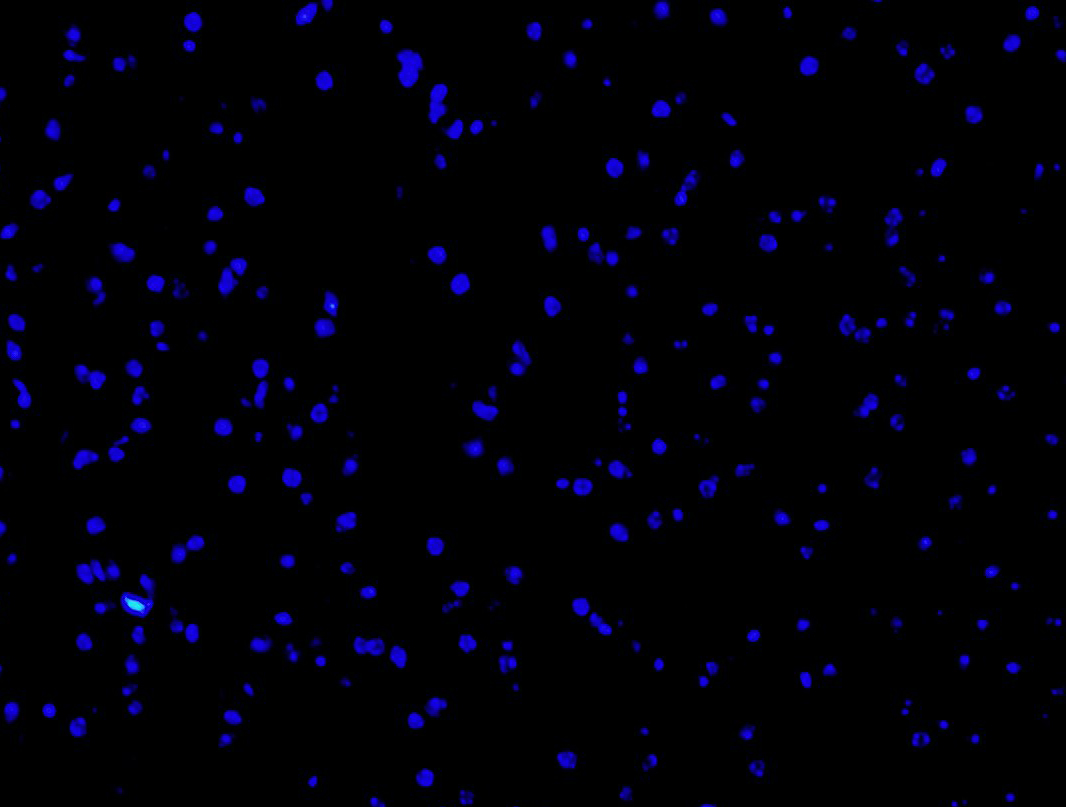

Supplement: Supplementary file 6 [file DataSheet2.ZIP › immunofluorescence of GLUT4/part 1 experiment/CUMS/M-3.jpg]

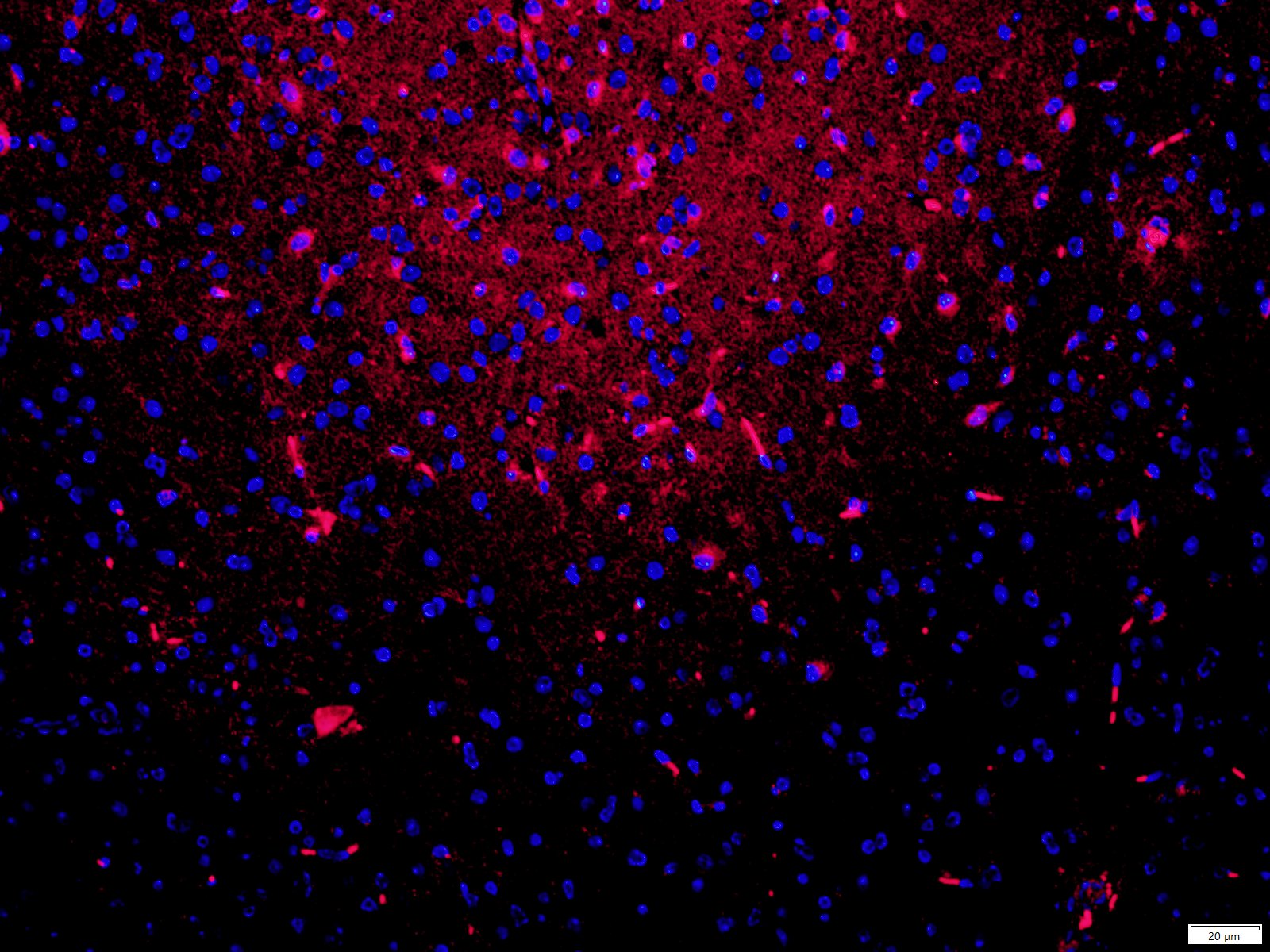

Supplement: Supplementary file 6 [file DataSheet2.ZIP › immunofluorescence of GLUT4/part 1 experiment/Flex/F.jpg]

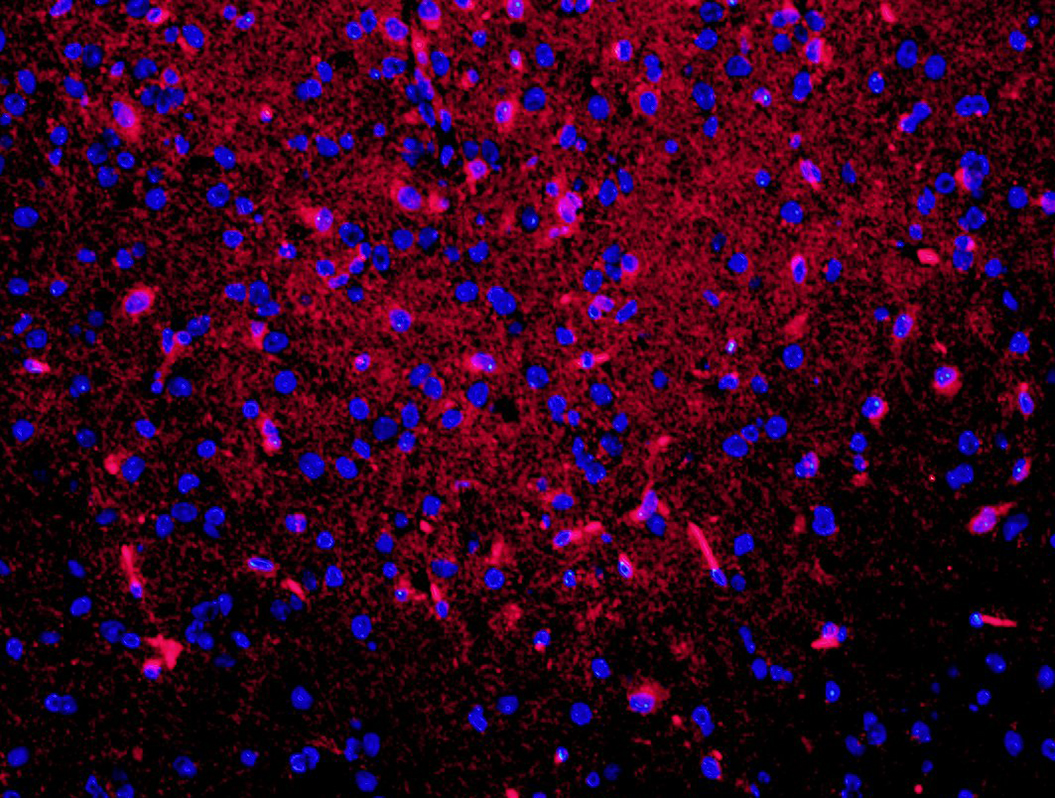

Supplement: Supplementary file 6 [file DataSheet2.ZIP › immunofluorescence of GLUT4/part 1 experiment/Flex/F-1.jpg]

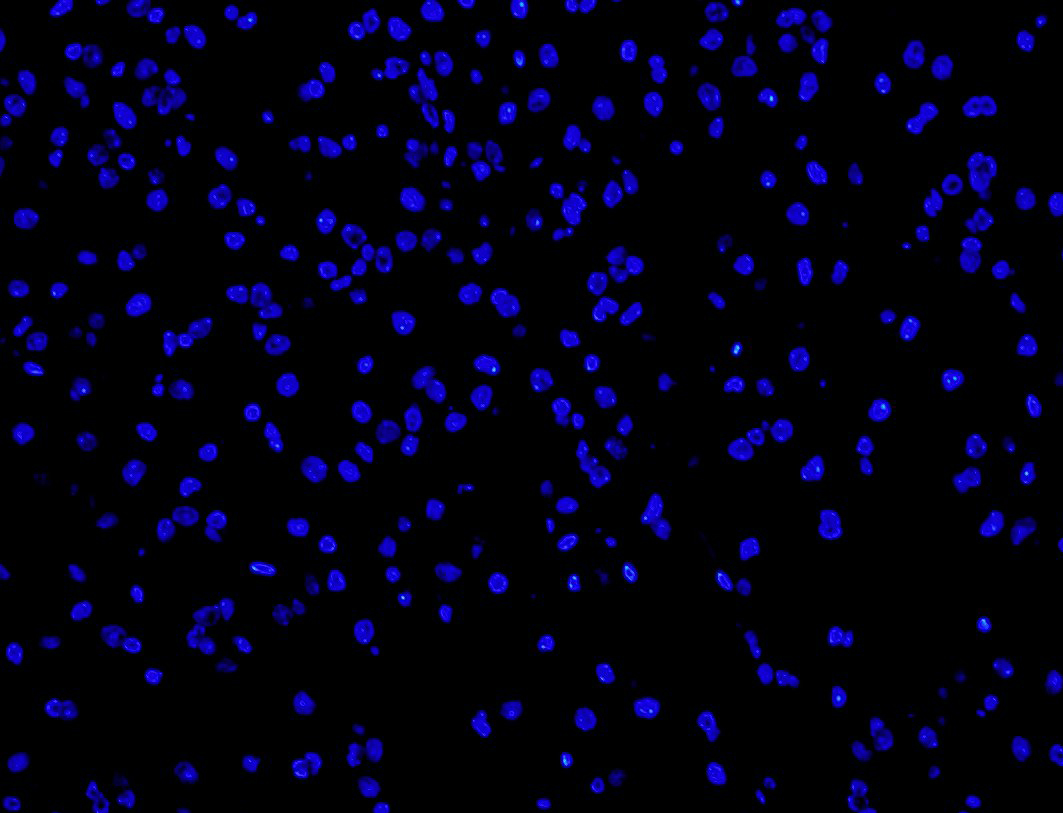

Supplement: Supplementary file 6 [file DataSheet2.ZIP › immunofluorescence of GLUT4/part 1 experiment/Flex/F-2.jpg]

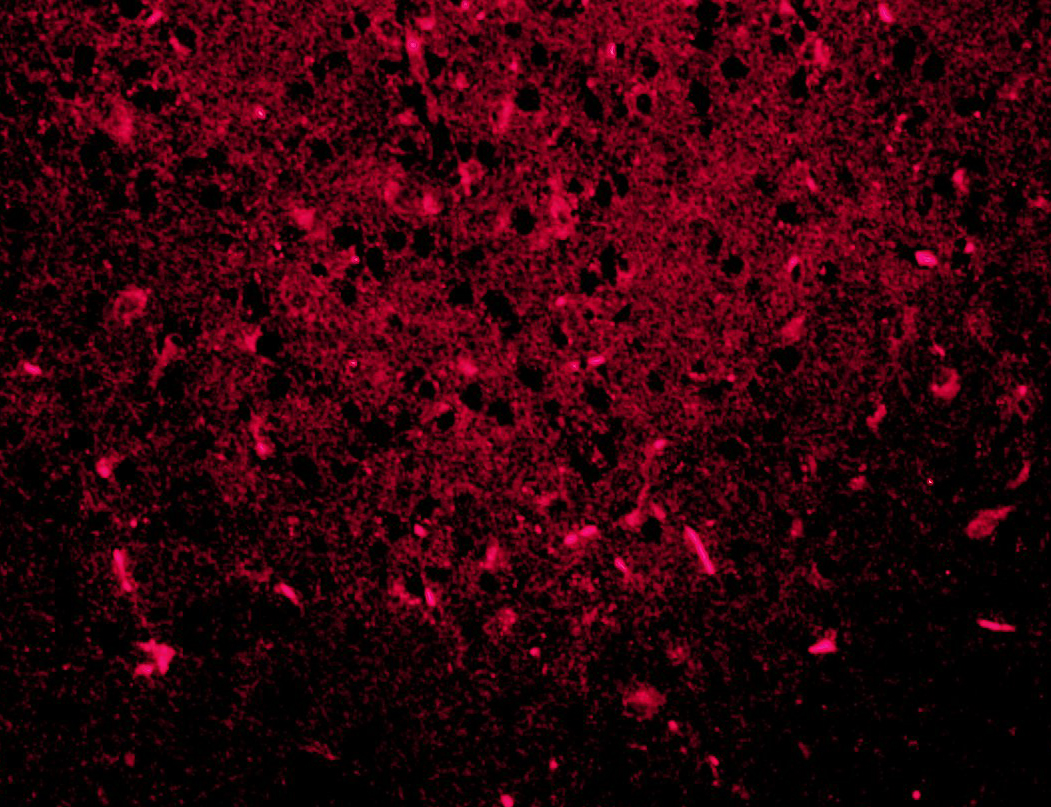

Supplement: Supplementary file 6 [file DataSheet2.ZIP › immunofluorescence of GLUT4/part 1 experiment/Flex/F-3.jpg]

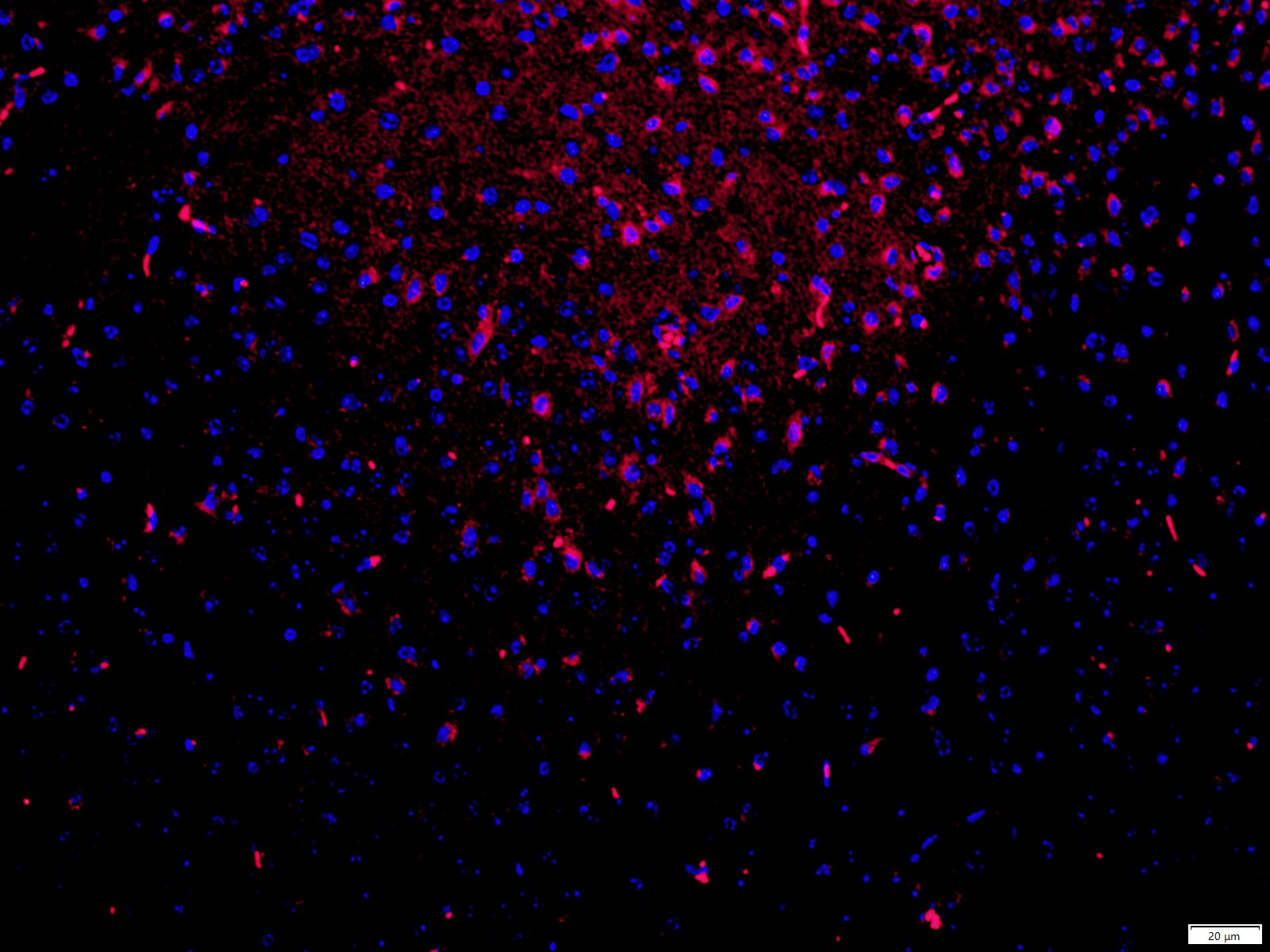

Supplement: Supplementary file 6 [file DataSheet2.ZIP › immunofluorescence of GLUT4/part 1 experiment/XYS/X.jpg]

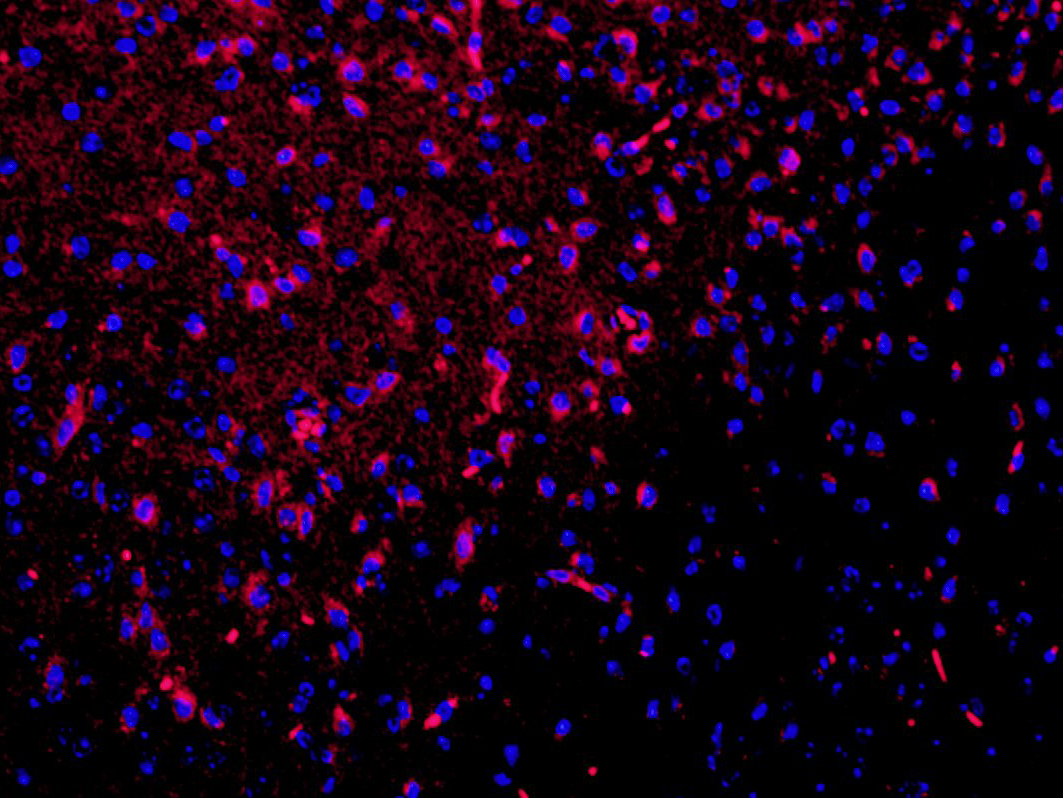

Supplement: Supplementary file 6 [file DataSheet2.ZIP › immunofluorescence of GLUT4/part 1 experiment/XYS/X-1.jpg]

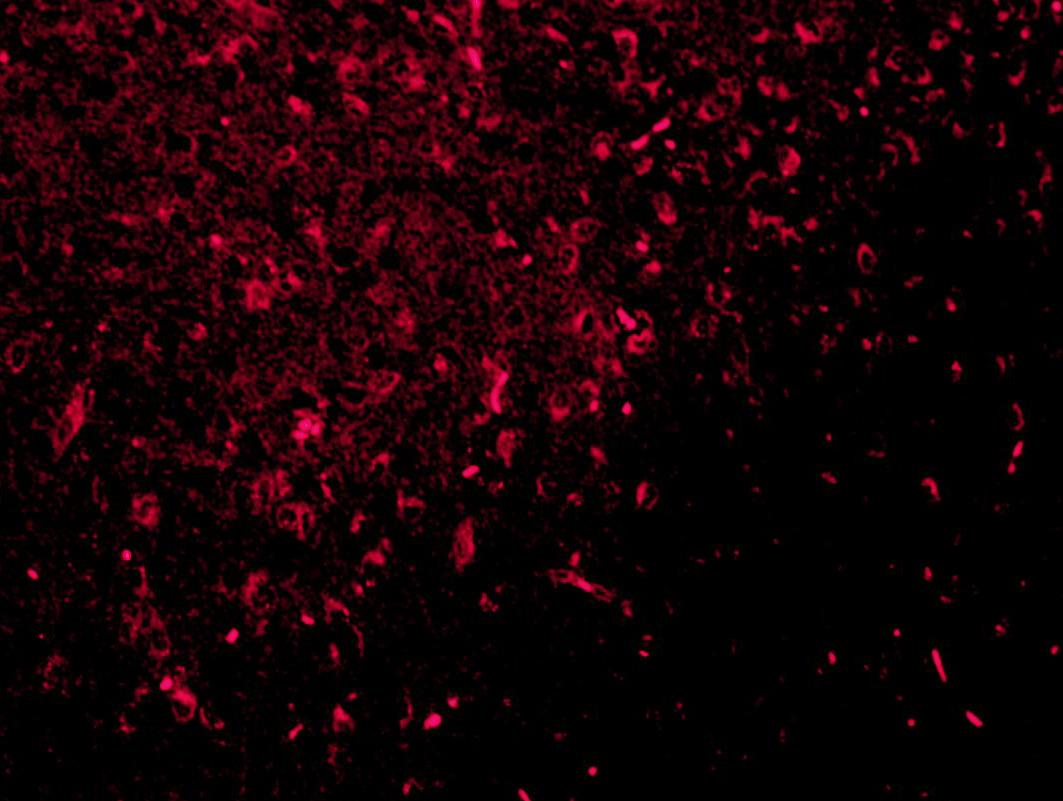

Supplement: Supplementary file 6 [file DataSheet2.ZIP › immunofluorescence of GLUT4/part 1 experiment/XYS/X-2.jpg]

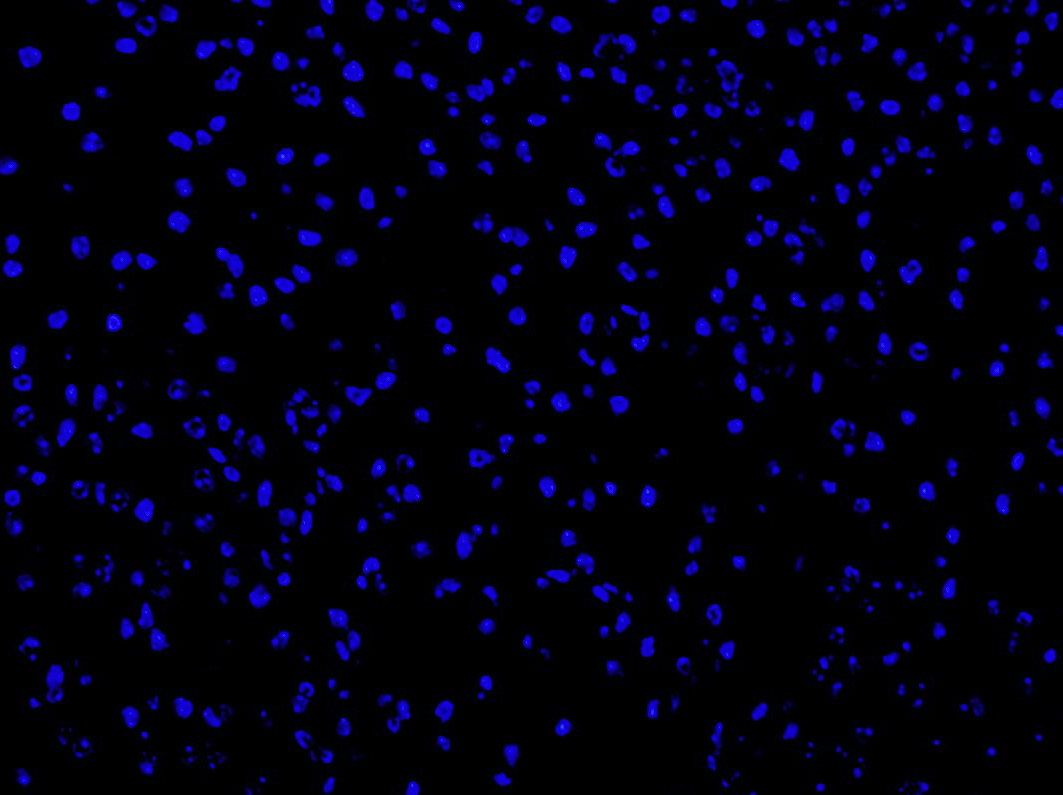

Supplement: Supplementary file 6 [file DataSheet2.ZIP › immunofluorescence of GLUT4/part 1 experiment/XYS/X-3.jpg]

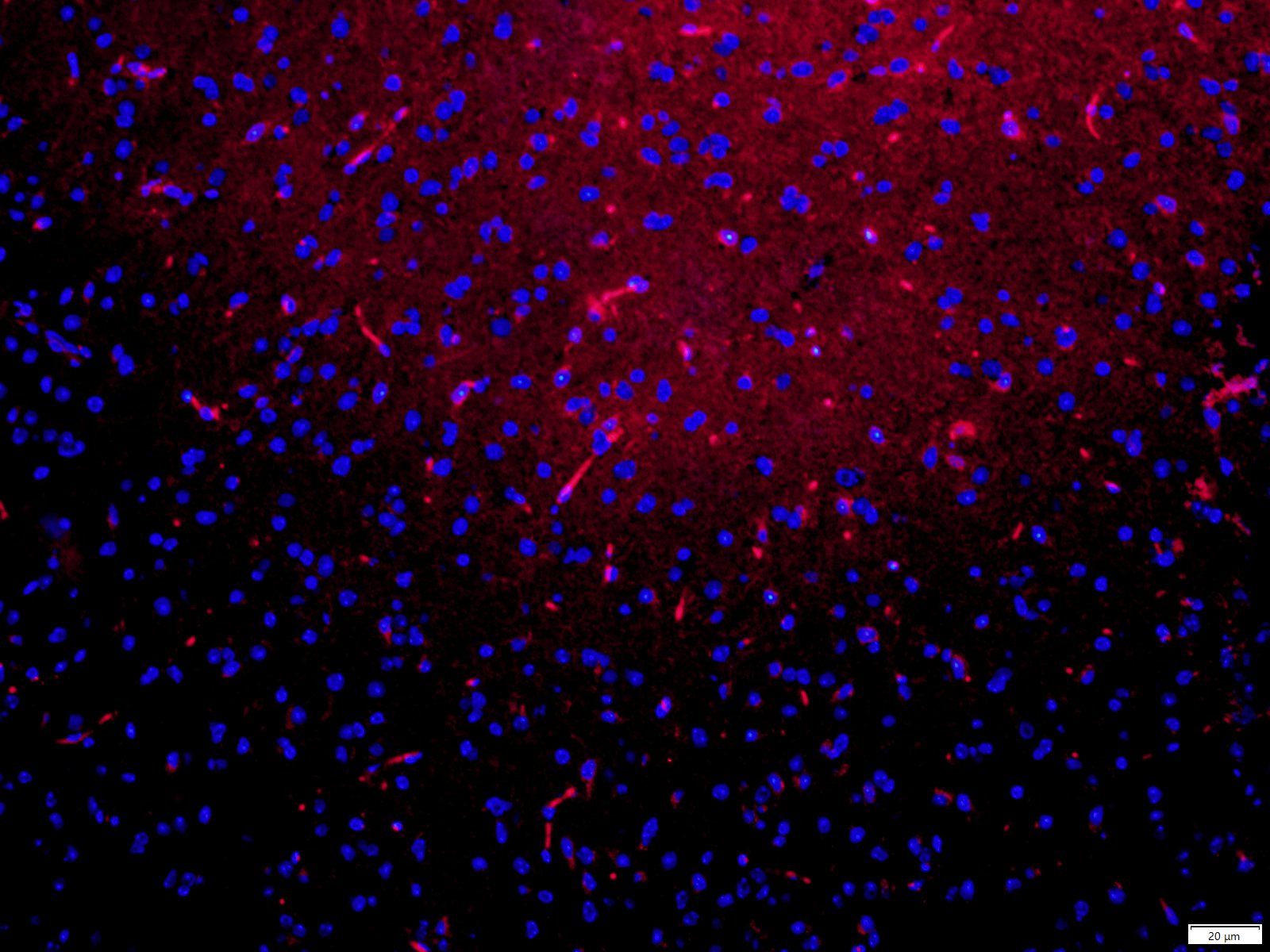

Supplement: Supplementary file 6 [file DataSheet2.ZIP › immunofluorescence of GLUT4/part 1 experiment/XYS/═╝╧±_01.jpg]

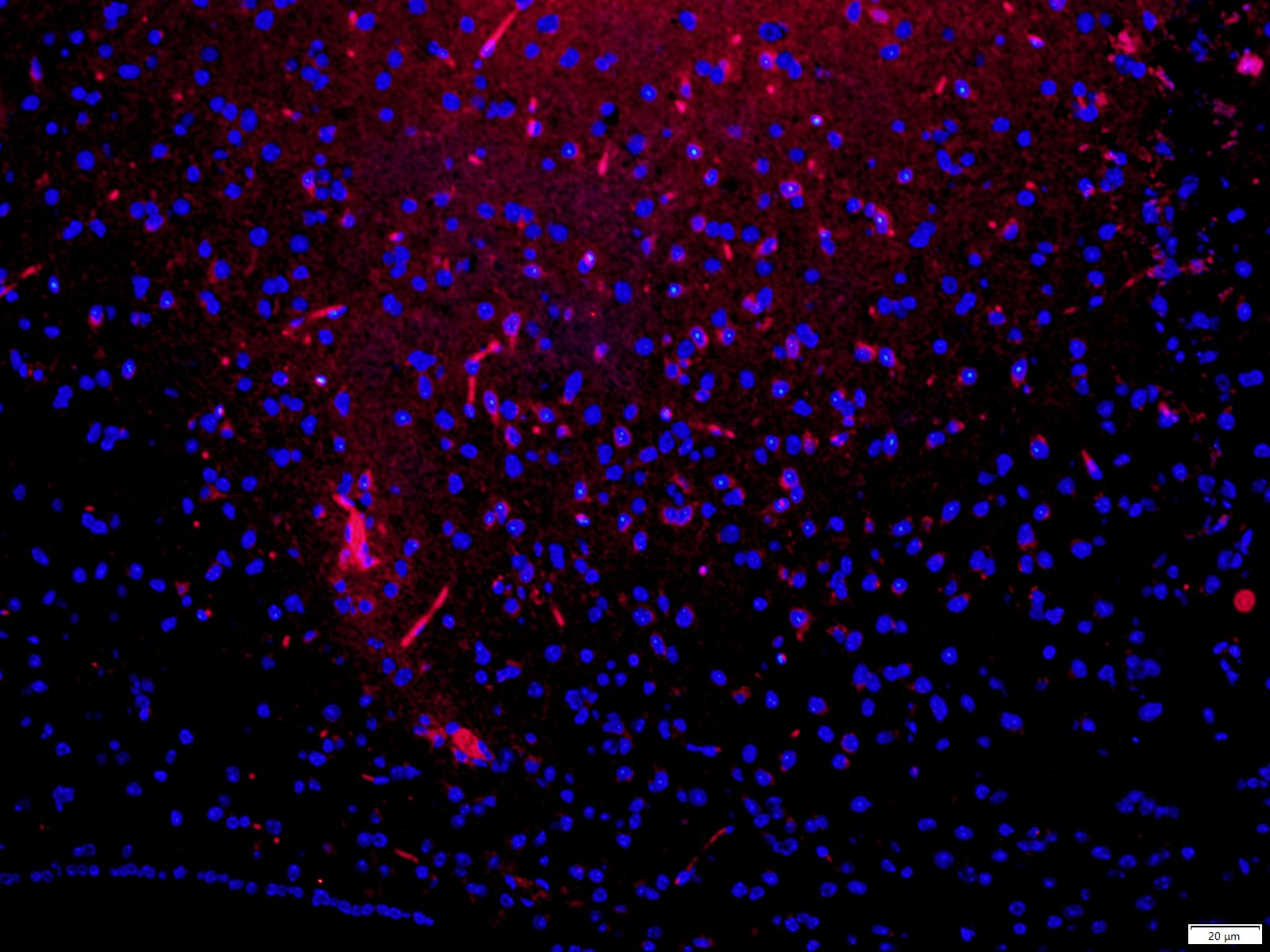

Supplement: Supplementary file 6 [file DataSheet2.ZIP › immunofluorescence of GLUT4/part 1 experiment/XYS/═╝╧±_02.jpg]

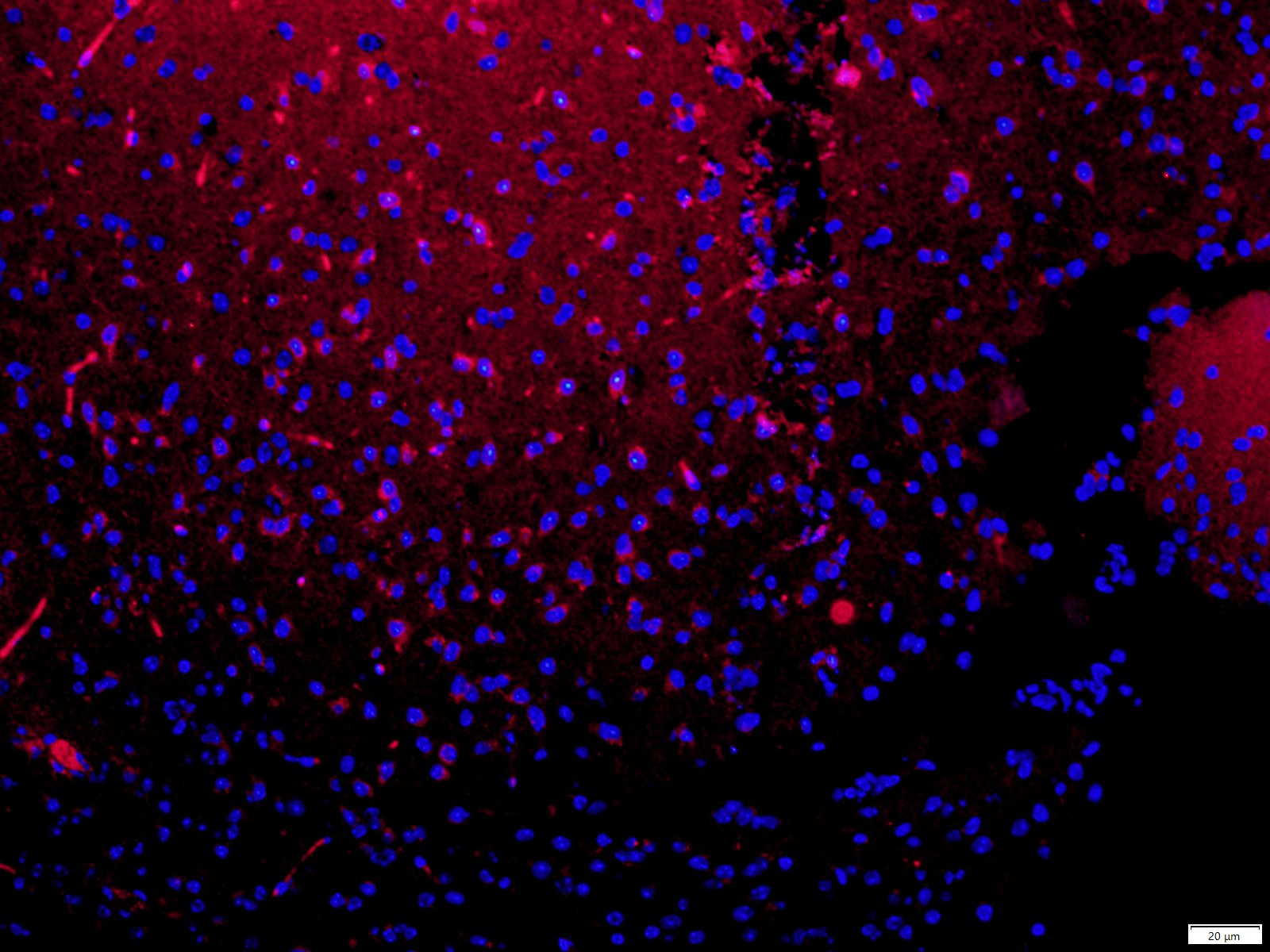

Supplement: Supplementary file 6 [file DataSheet2.ZIP › immunofluorescence of GLUT4/part 1 experiment/XYS/═╝╧±_03.jpg]

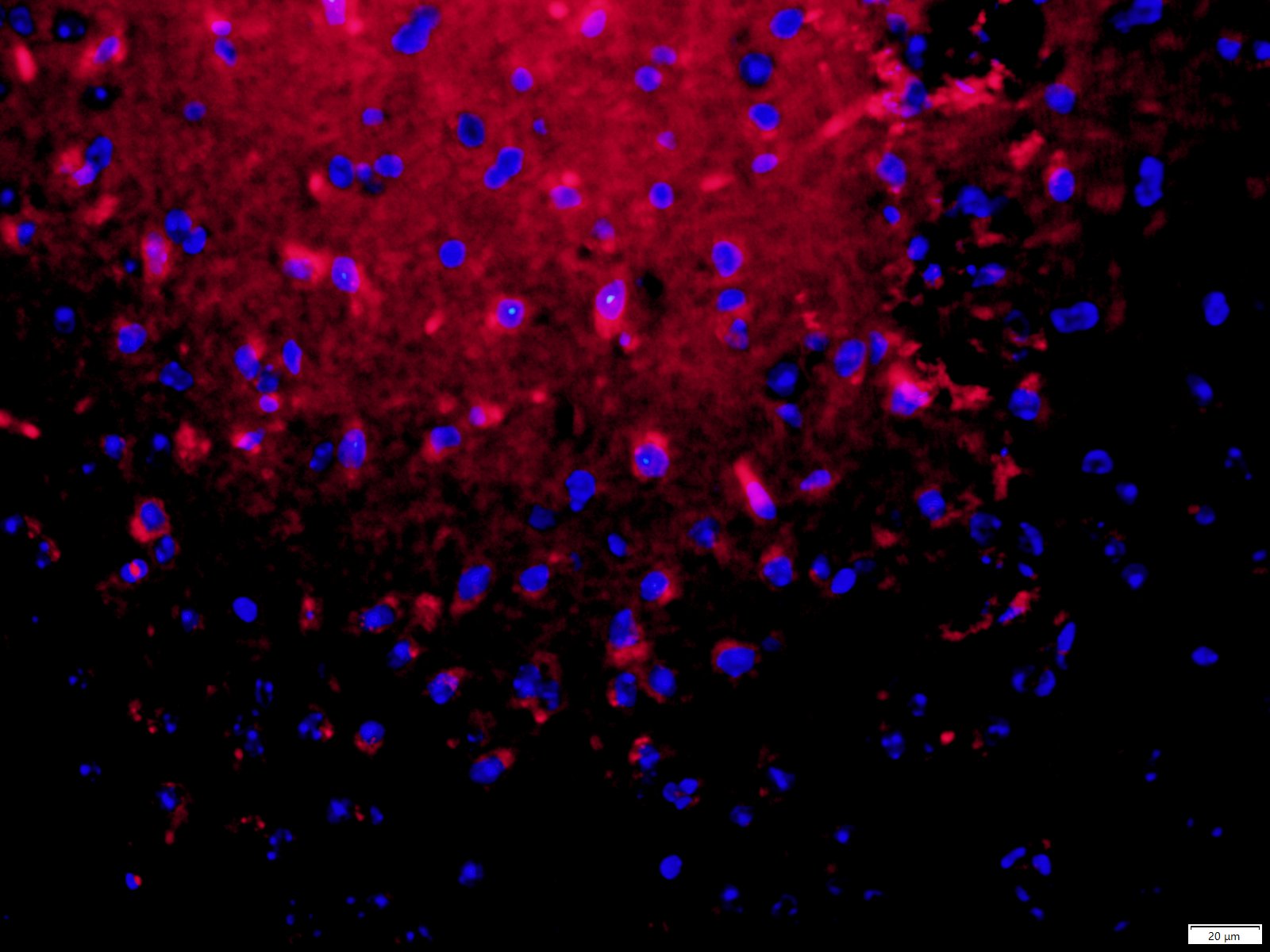

Supplement: Supplementary file 6 [file DataSheet2.ZIP › immunofluorescence of GLUT4/part 1 experiment/XYS/═╝╧±_04.jpg]

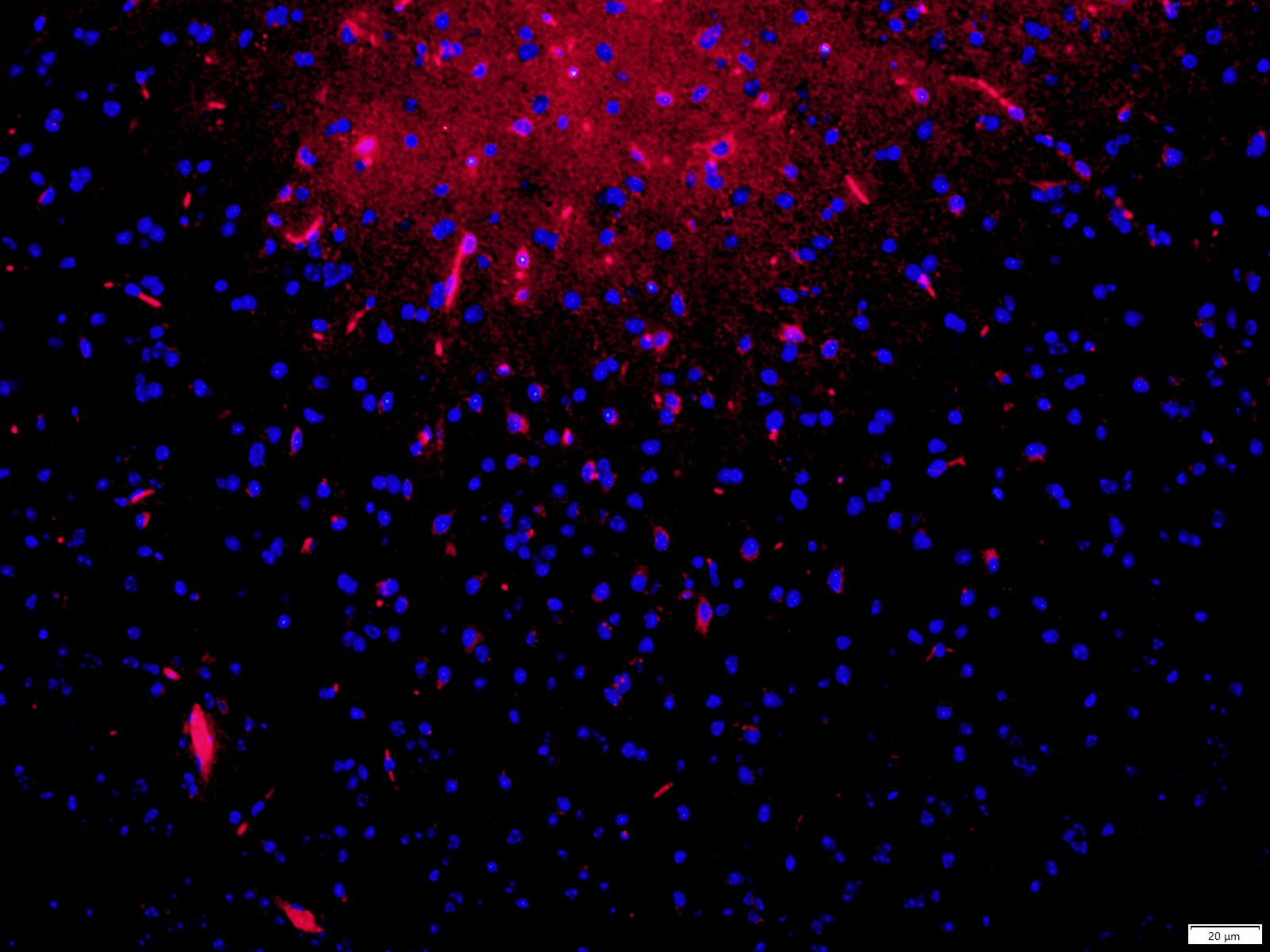

Supplement: Supplementary file 6 [file DataSheet2.ZIP › immunofluorescence of GLUT4/part 1 experiment/XYS/═╝╧±_05.jpg]

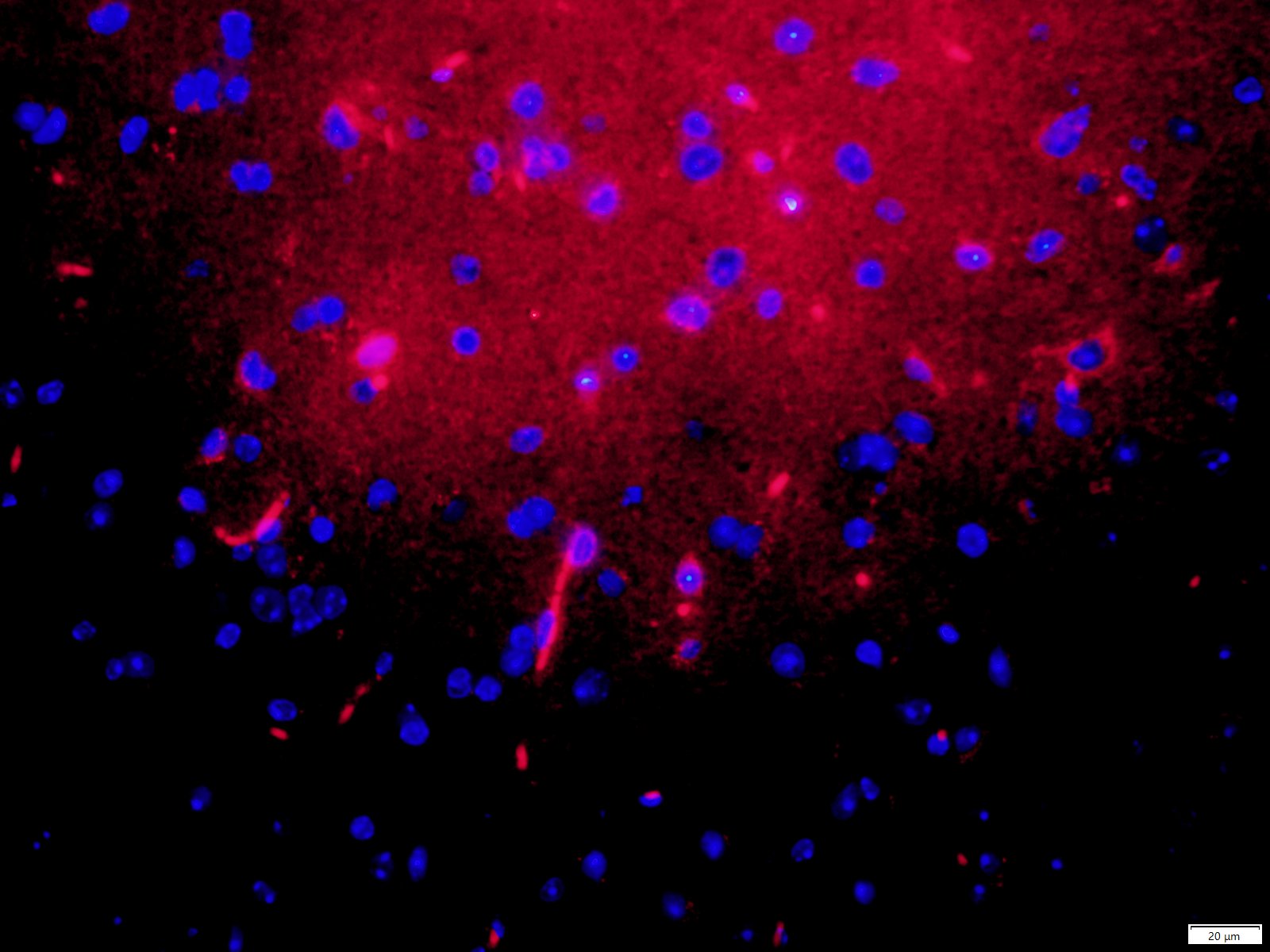

Supplement: Supplementary file 6 [file DataSheet2.ZIP › immunofluorescence of GLUT4/part 1 experiment/XYS/═╝╧±_06.jpg]

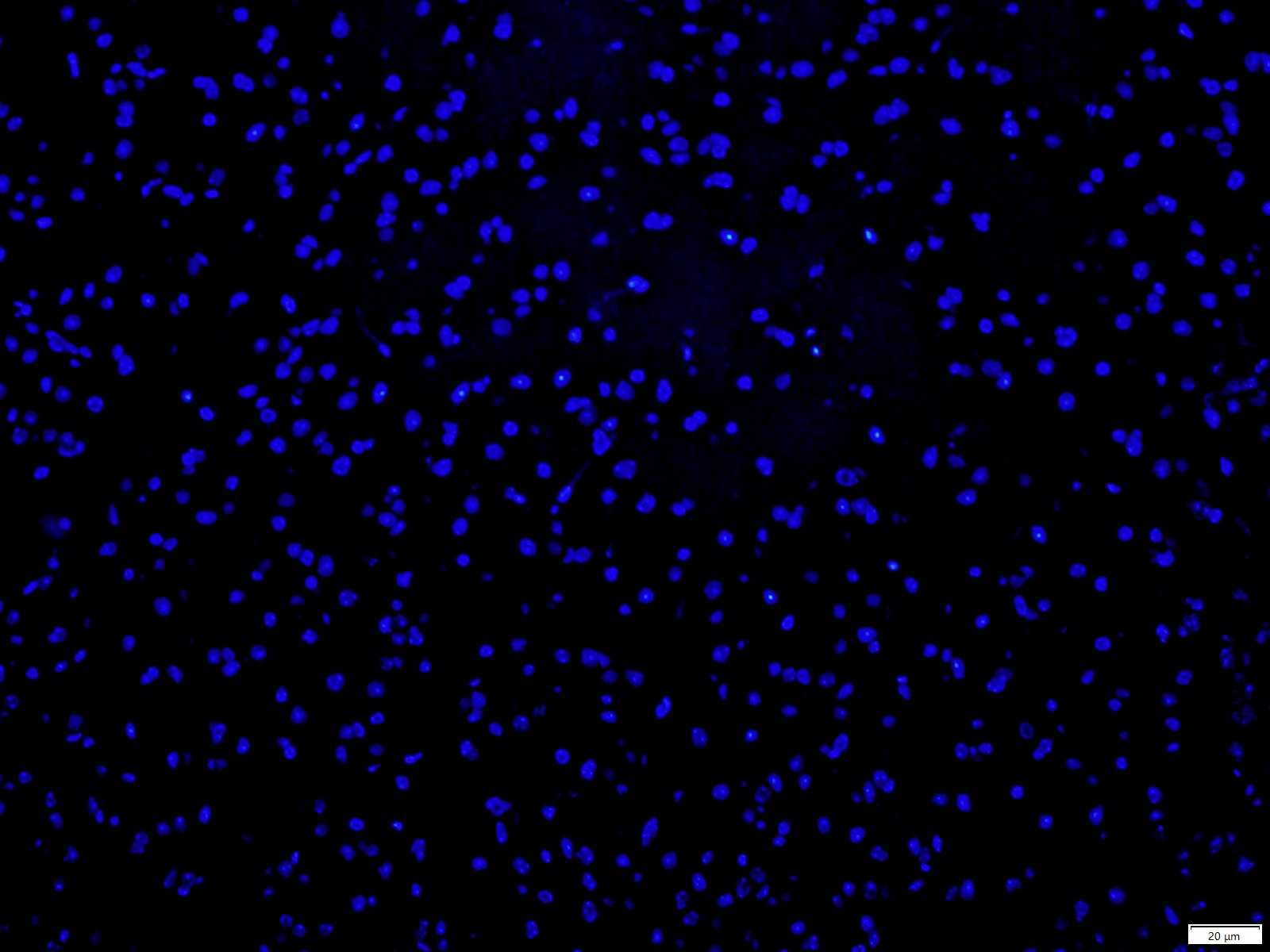

Supplement: Supplementary file 6 [file DataSheet2.ZIP › immunofluorescence of GLUT4/part 1 experiment/XYS/═╝╧±_3756.jpg]

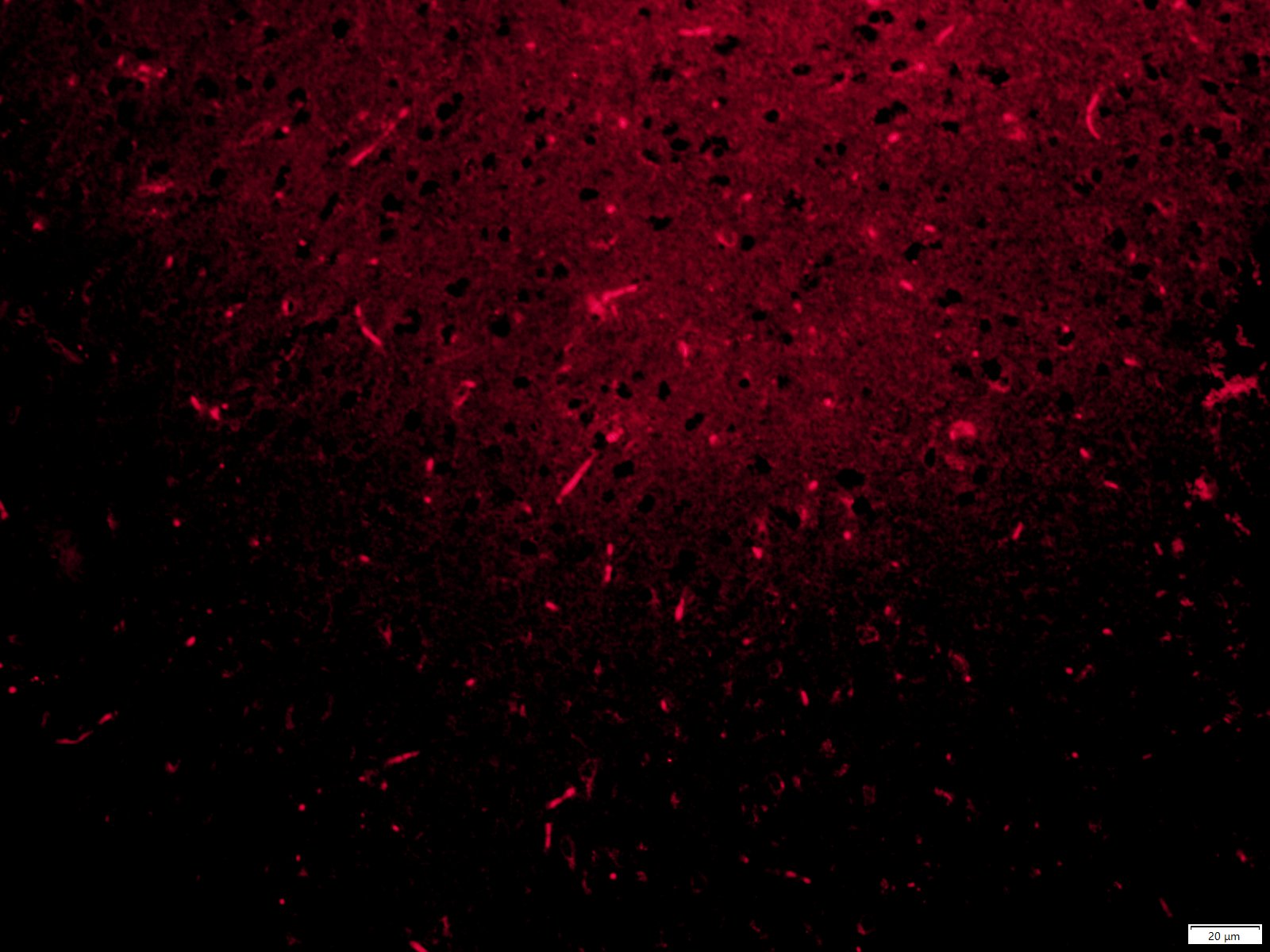

Supplement: Supplementary file 6 [file DataSheet2.ZIP › immunofluorescence of GLUT4/part 1 experiment/XYS/═╝╧±_3757.jpg]

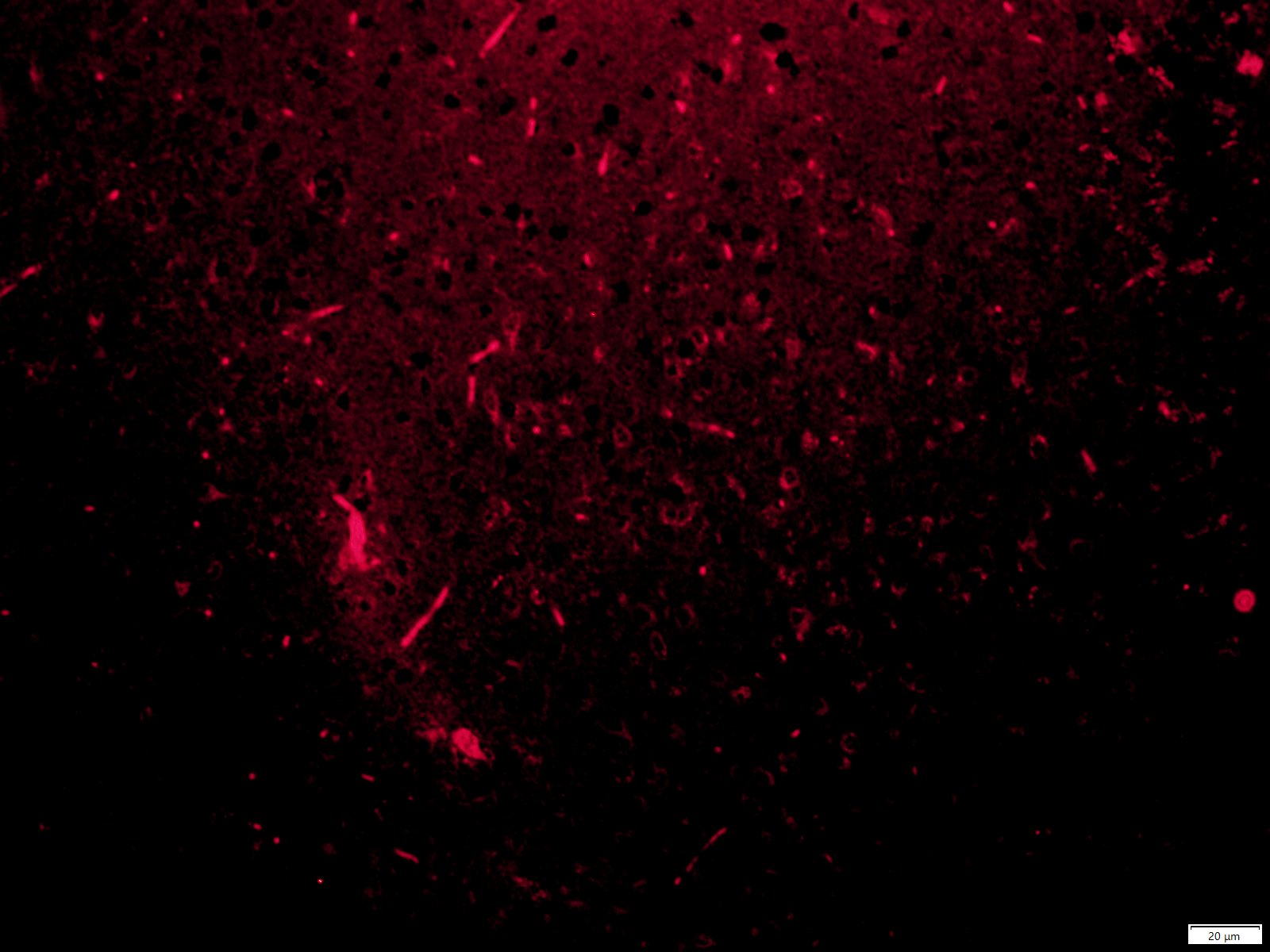

Supplement: Supplementary file 6 [file DataSheet2.ZIP › immunofluorescence of GLUT4/part 1 experiment/XYS/═╝╧±_3758.jpg]

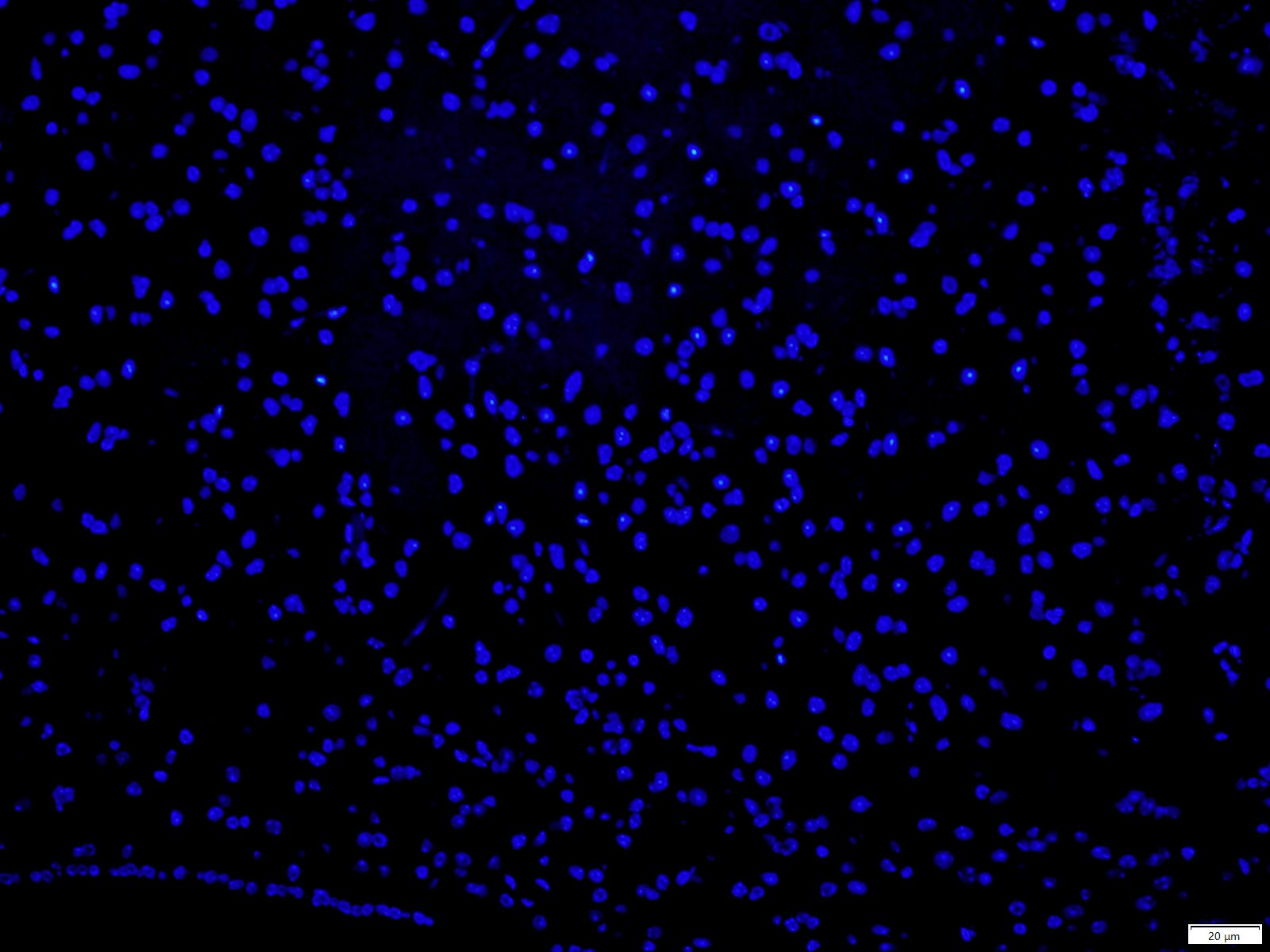

Supplement: Supplementary file 6 [file DataSheet2.ZIP › immunofluorescence of GLUT4/part 1 experiment/XYS/═╝╧±_3759.jpg]

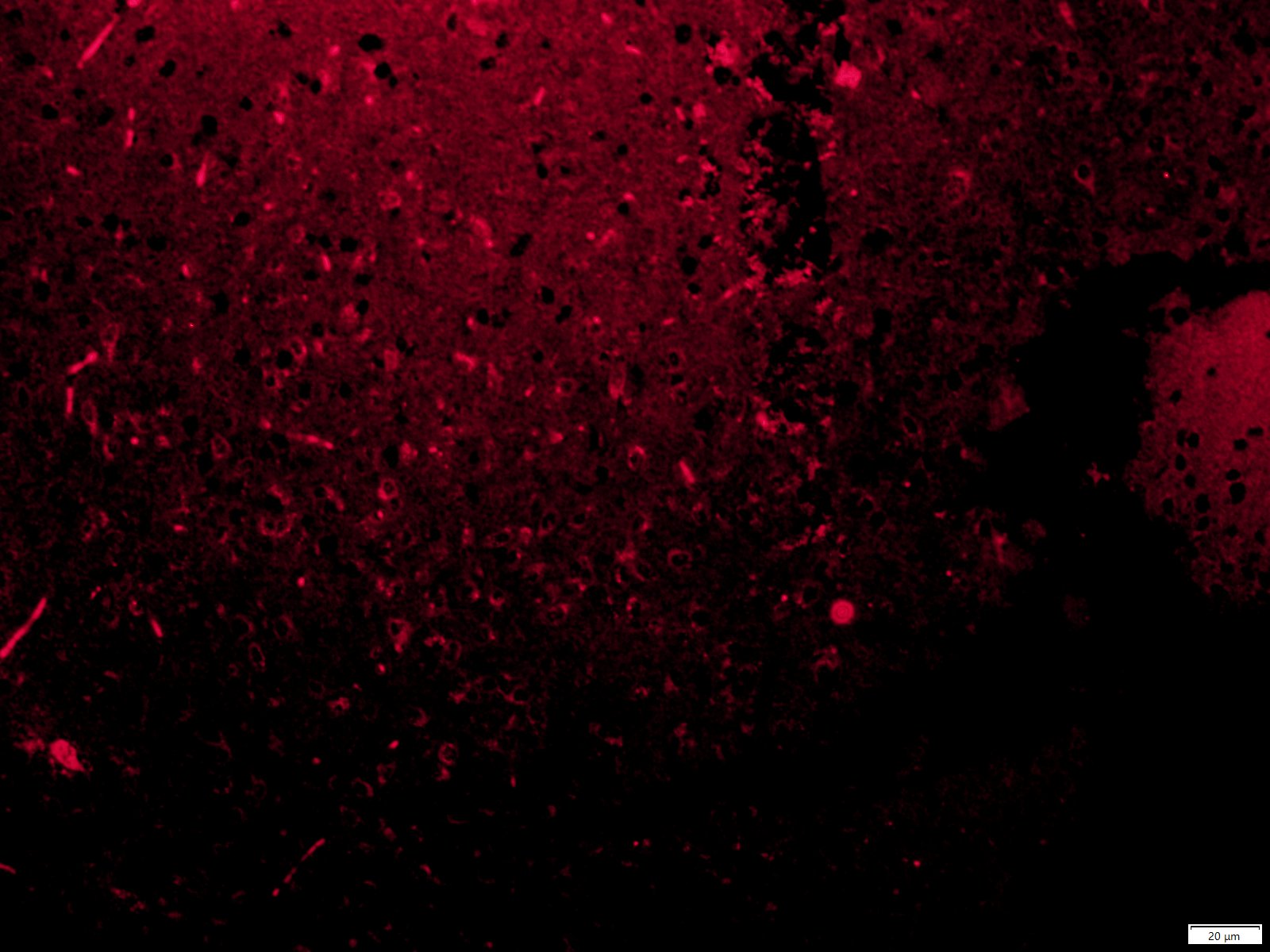

Supplement: Supplementary file 6 [file DataSheet2.ZIP › immunofluorescence of GLUT4/part 1 experiment/XYS/═╝╧±_3760.jpg]

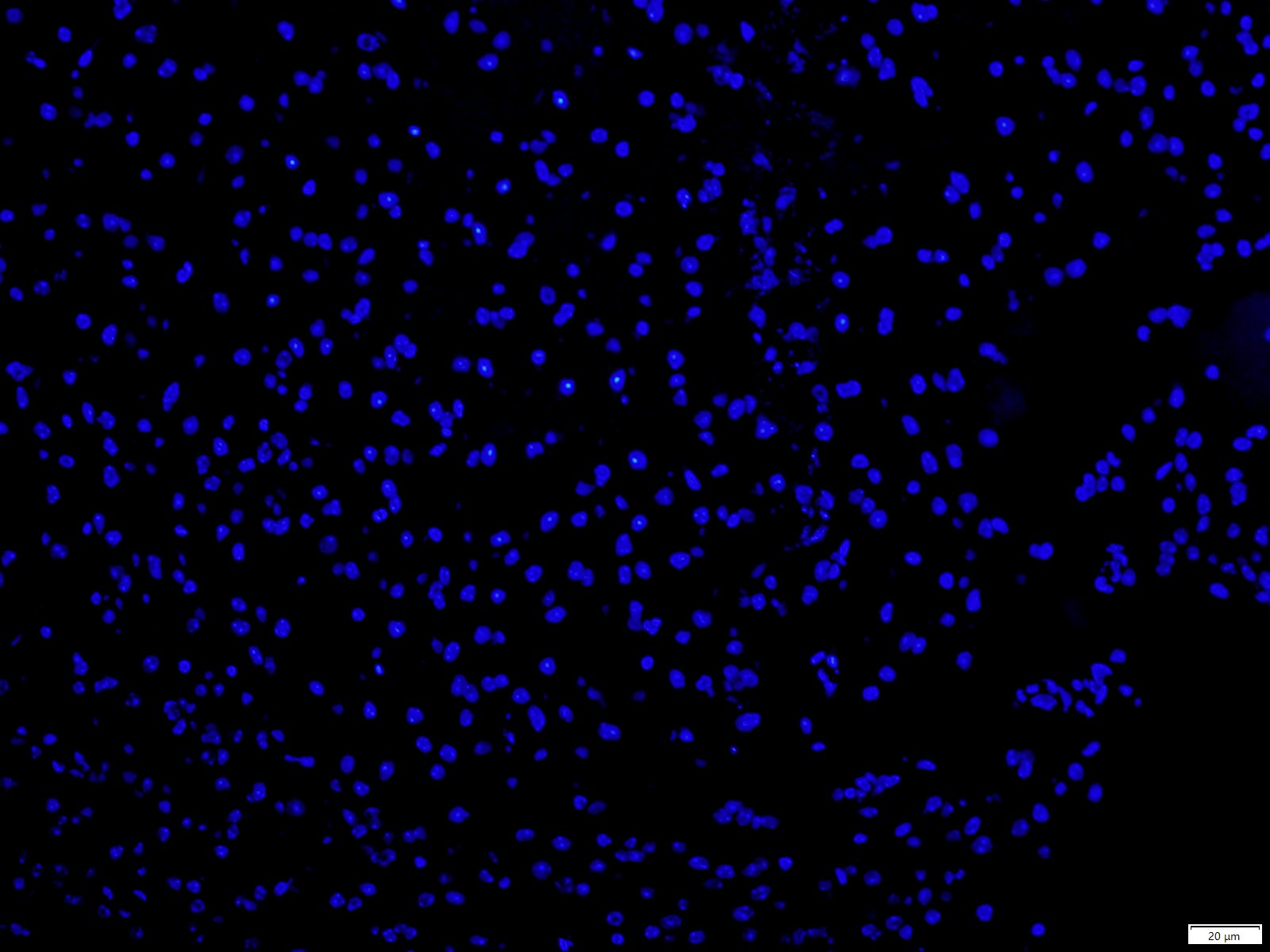

Supplement: Supplementary file 6 [file DataSheet2.ZIP › immunofluorescence of GLUT4/part 1 experiment/XYS/═╝╧±_3761.jpg]

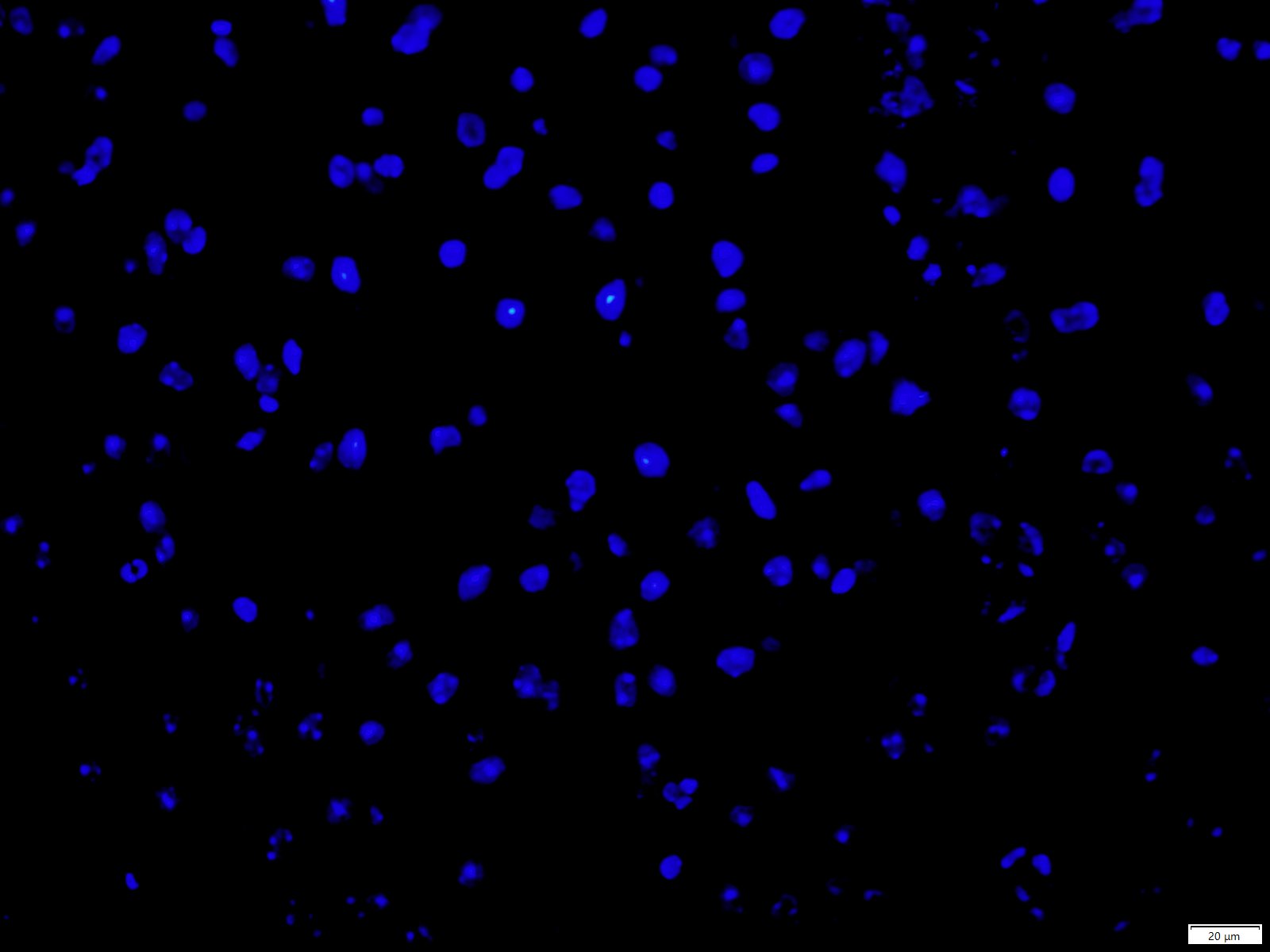

Supplement: Supplementary file 6 [file DataSheet2.ZIP › immunofluorescence of GLUT4/part 1 experiment/XYS/═╝╧±_3762.jpg]

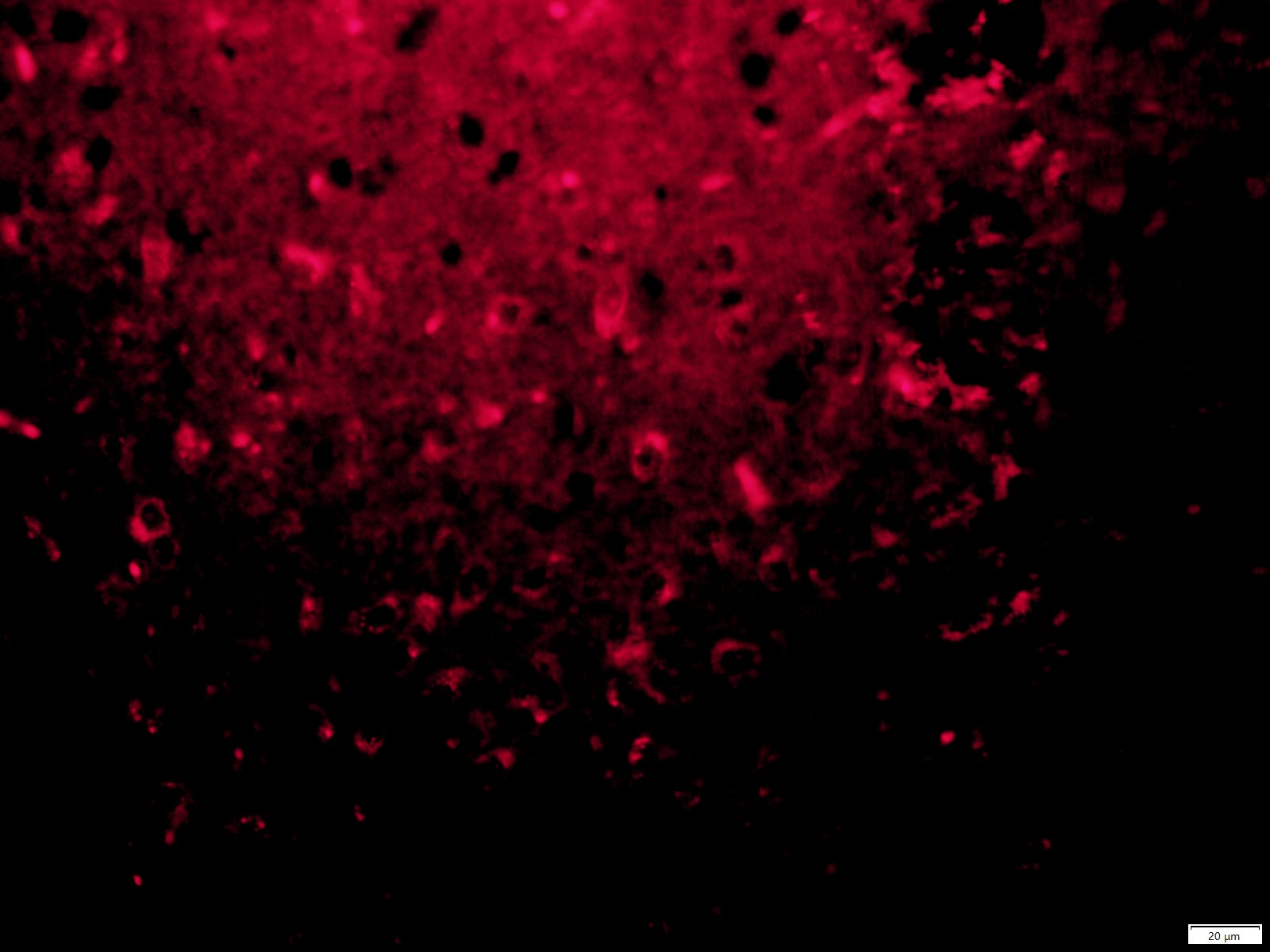

Supplement: Supplementary file 6 [file DataSheet2.ZIP › immunofluorescence of GLUT4/part 1 experiment/XYS/═╝╧±_3763.jpg]

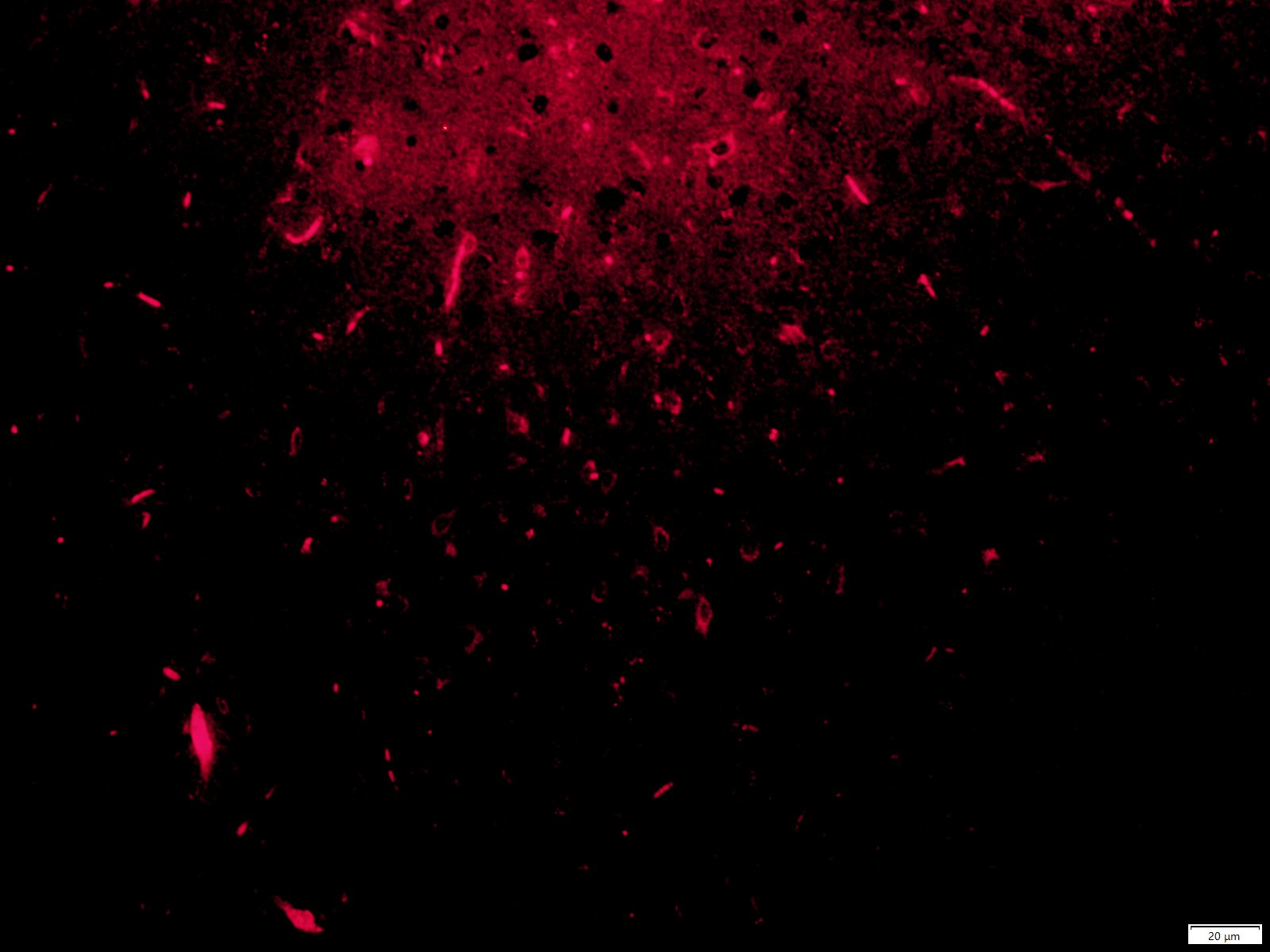

Supplement: Supplementary file 6 [file DataSheet2.ZIP › immunofluorescence of GLUT4/part 1 experiment/XYS/═╝╧±_3765.jpg]

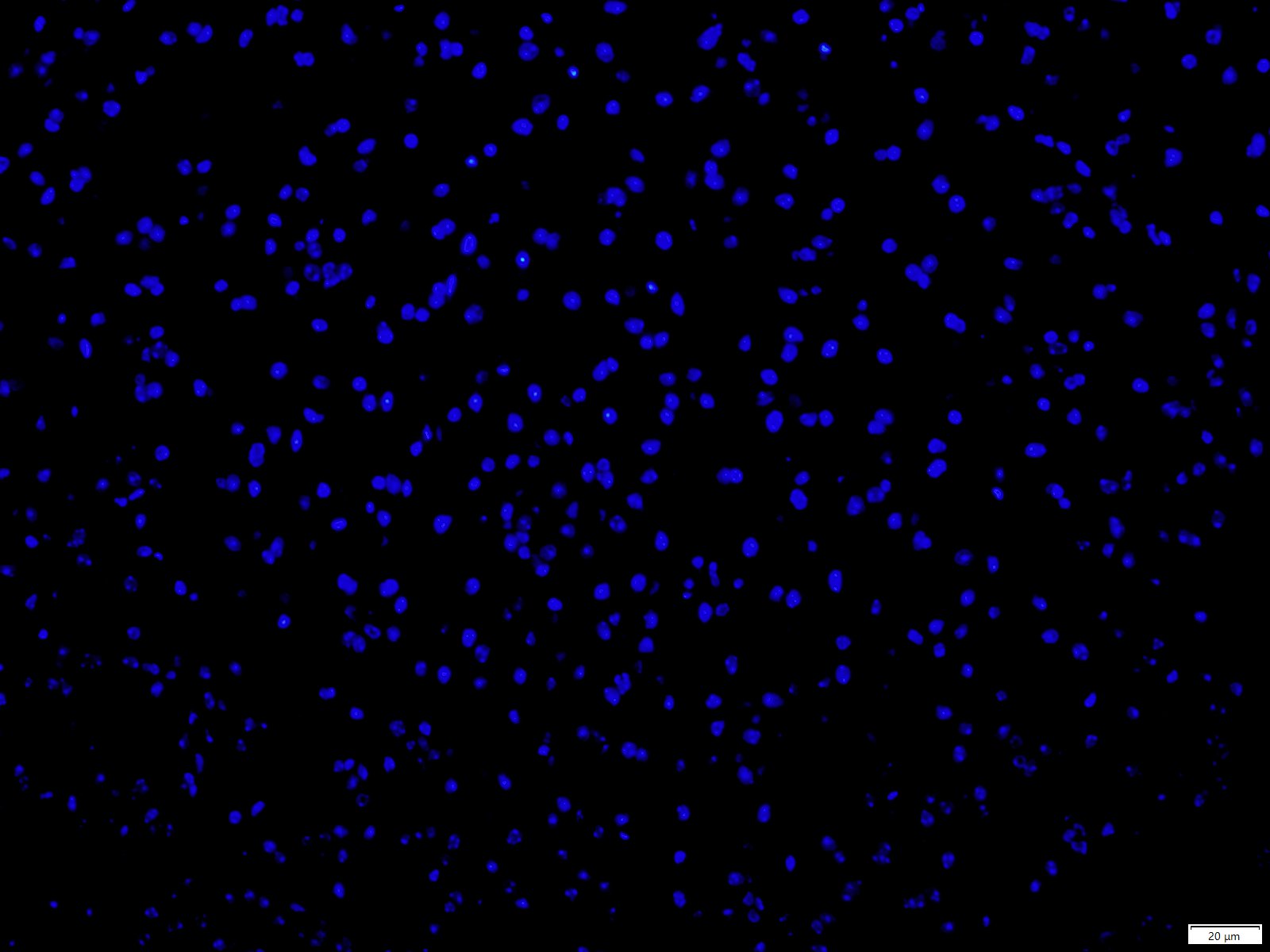

Supplement: Supplementary file 6 [file DataSheet2.ZIP › immunofluorescence of GLUT4/part 1 experiment/XYS/═╝╧±_3766.jpg]

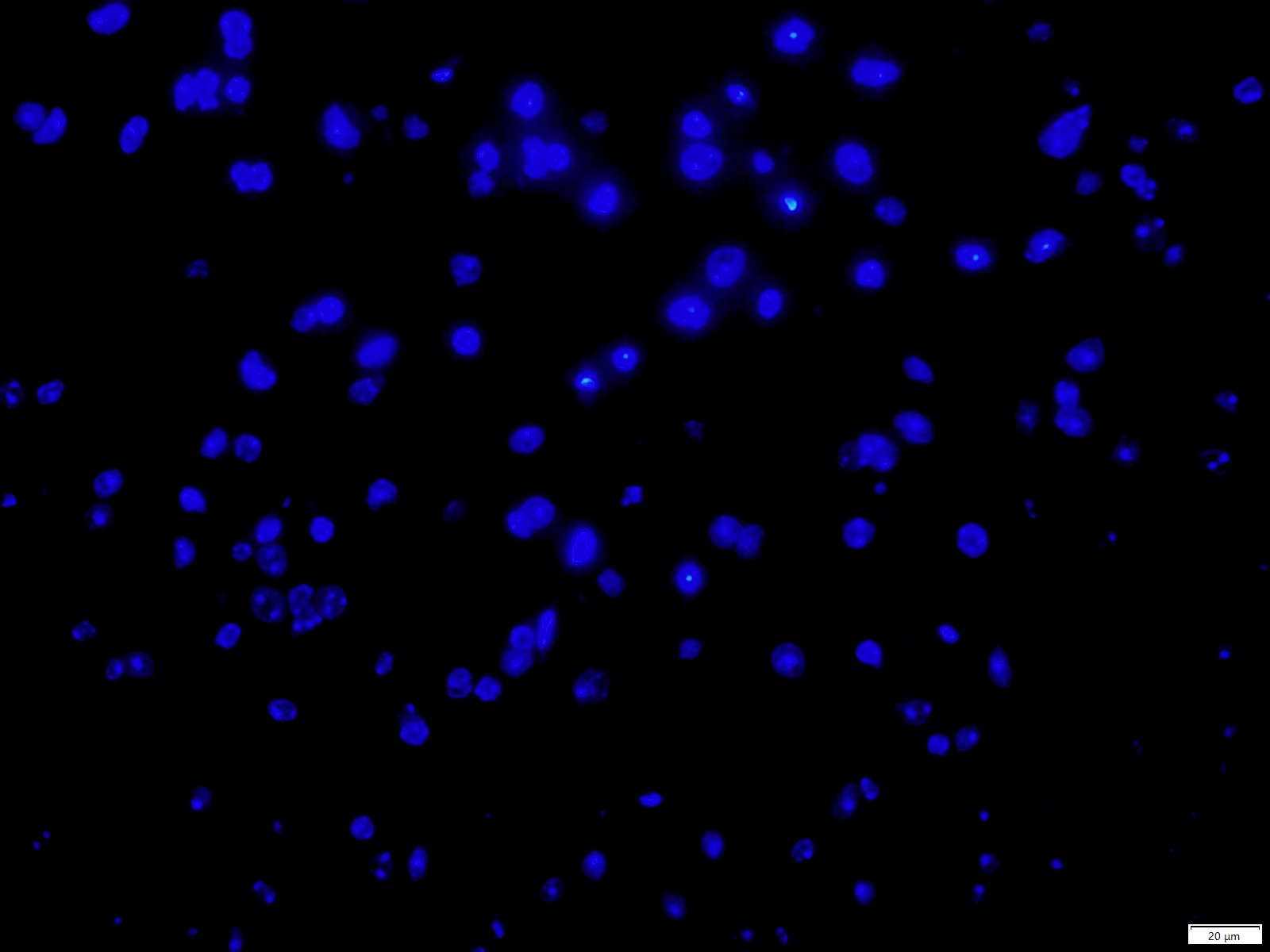

Supplement: Supplementary file 6 [file DataSheet2.ZIP › immunofluorescence of GLUT4/part 1 experiment/XYS/═╝╧±_3767.jpg]

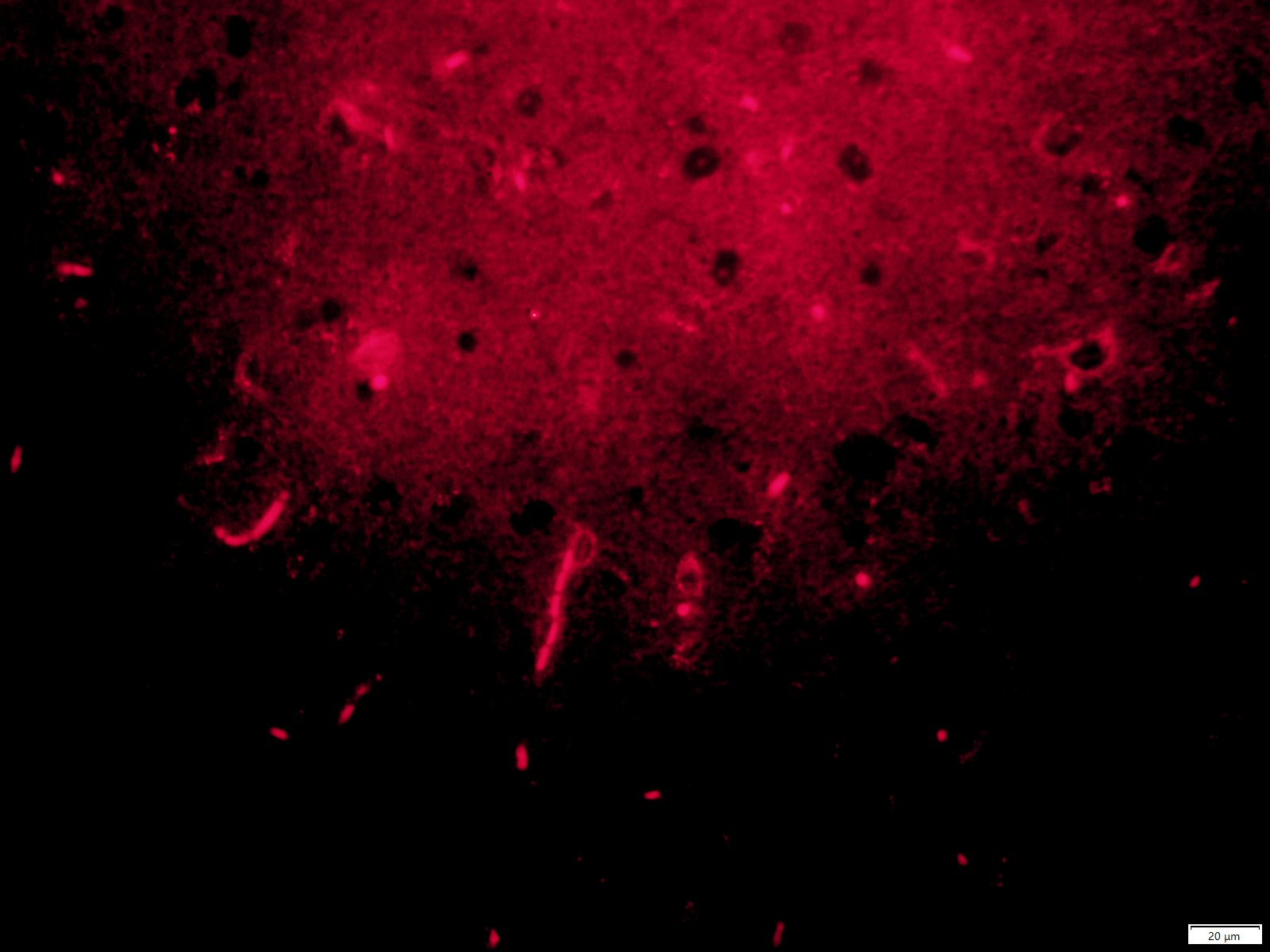

Supplement: Supplementary file 6 [file DataSheet2.ZIP › immunofluorescence of GLUT4/part 1 experiment/XYS/═╝╧±_3768.jpg]

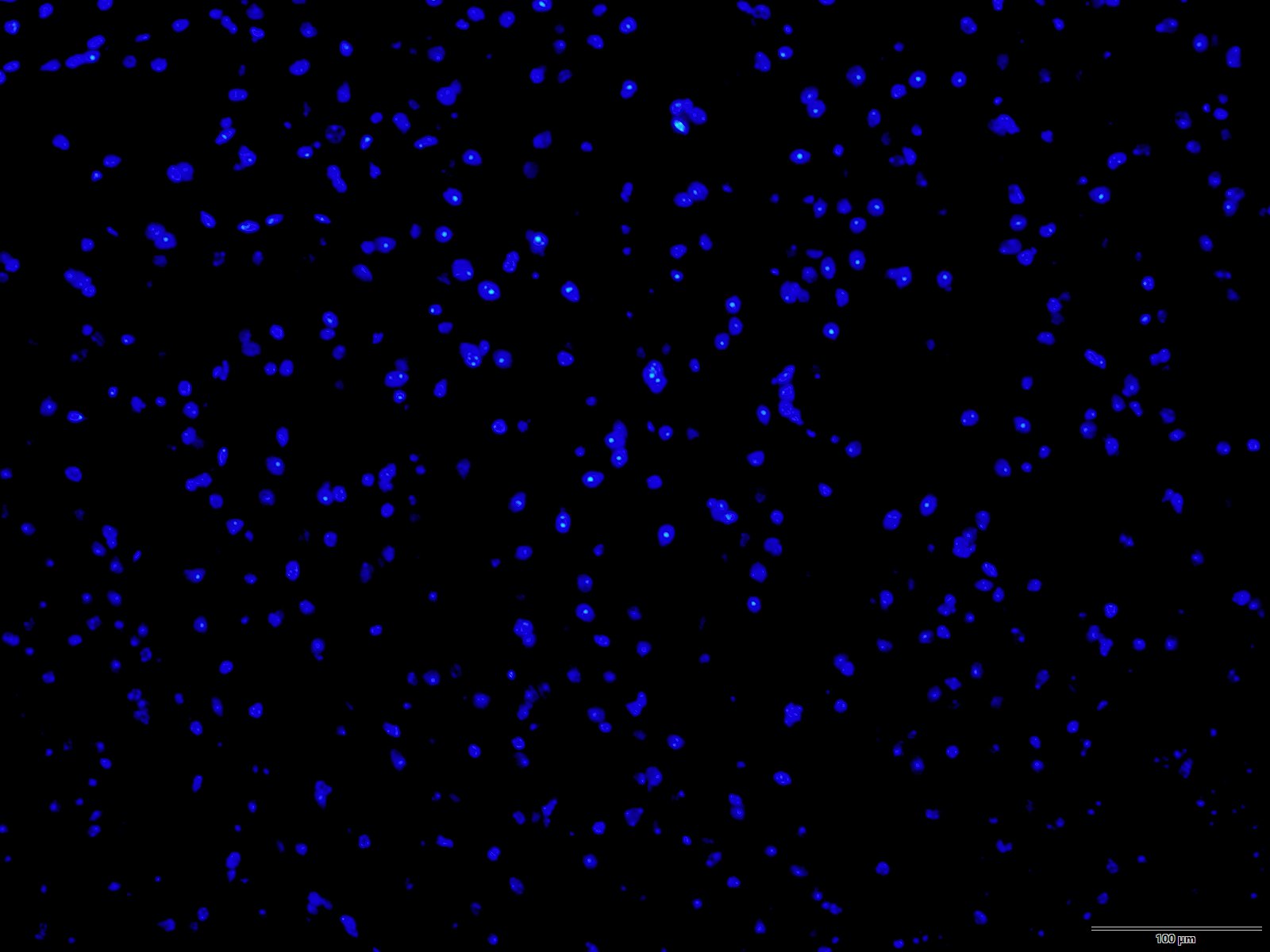

Supplement: Supplementary file 6 [file DataSheet2.ZIP › immunofluorescence of GLUT4/part 2 experiment/3-MA/1-1.jpg]

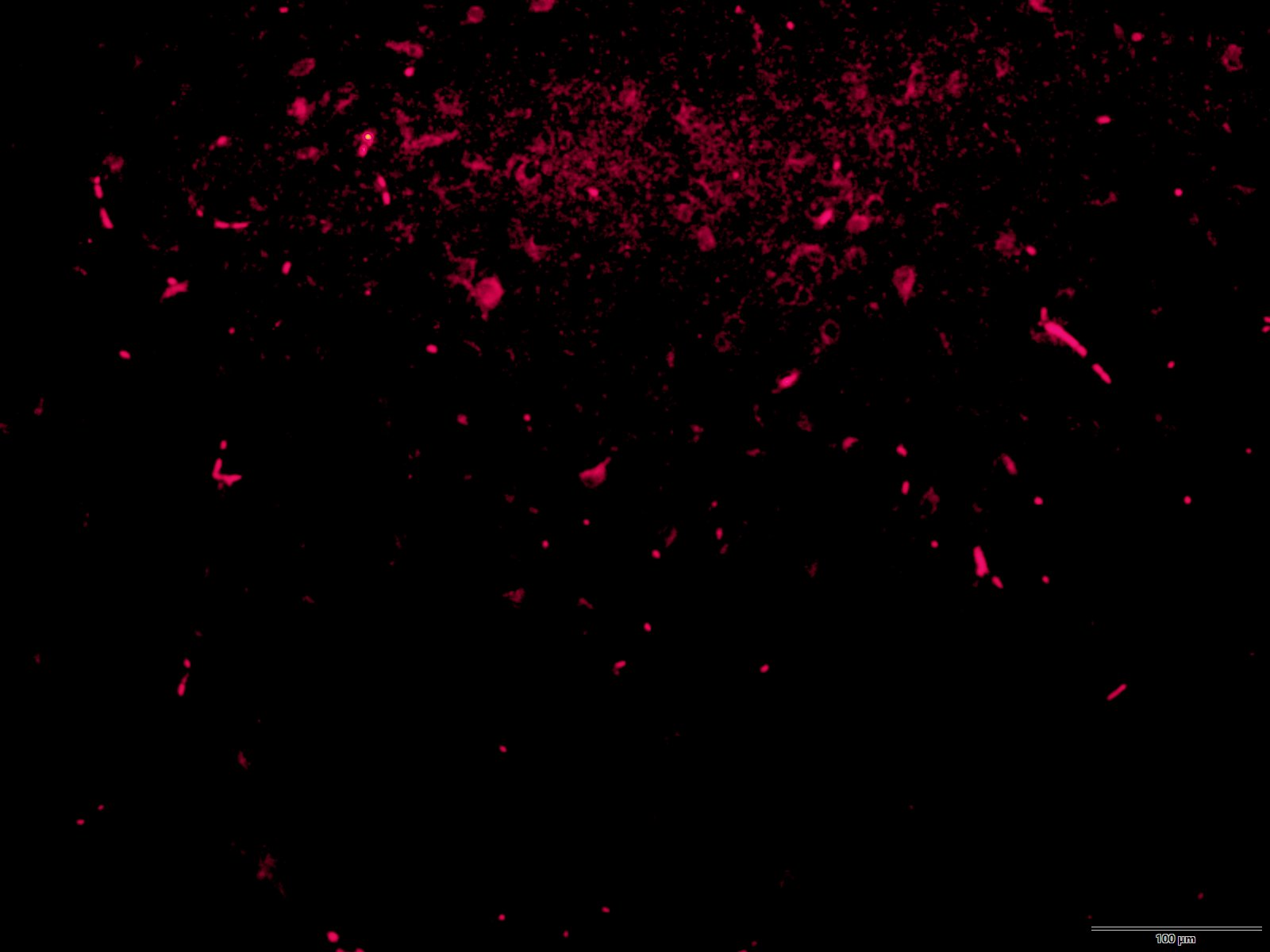

Supplement: Supplementary file 6 [file DataSheet2.ZIP › immunofluorescence of GLUT4/part 2 experiment/3-MA/1-2.jpg]

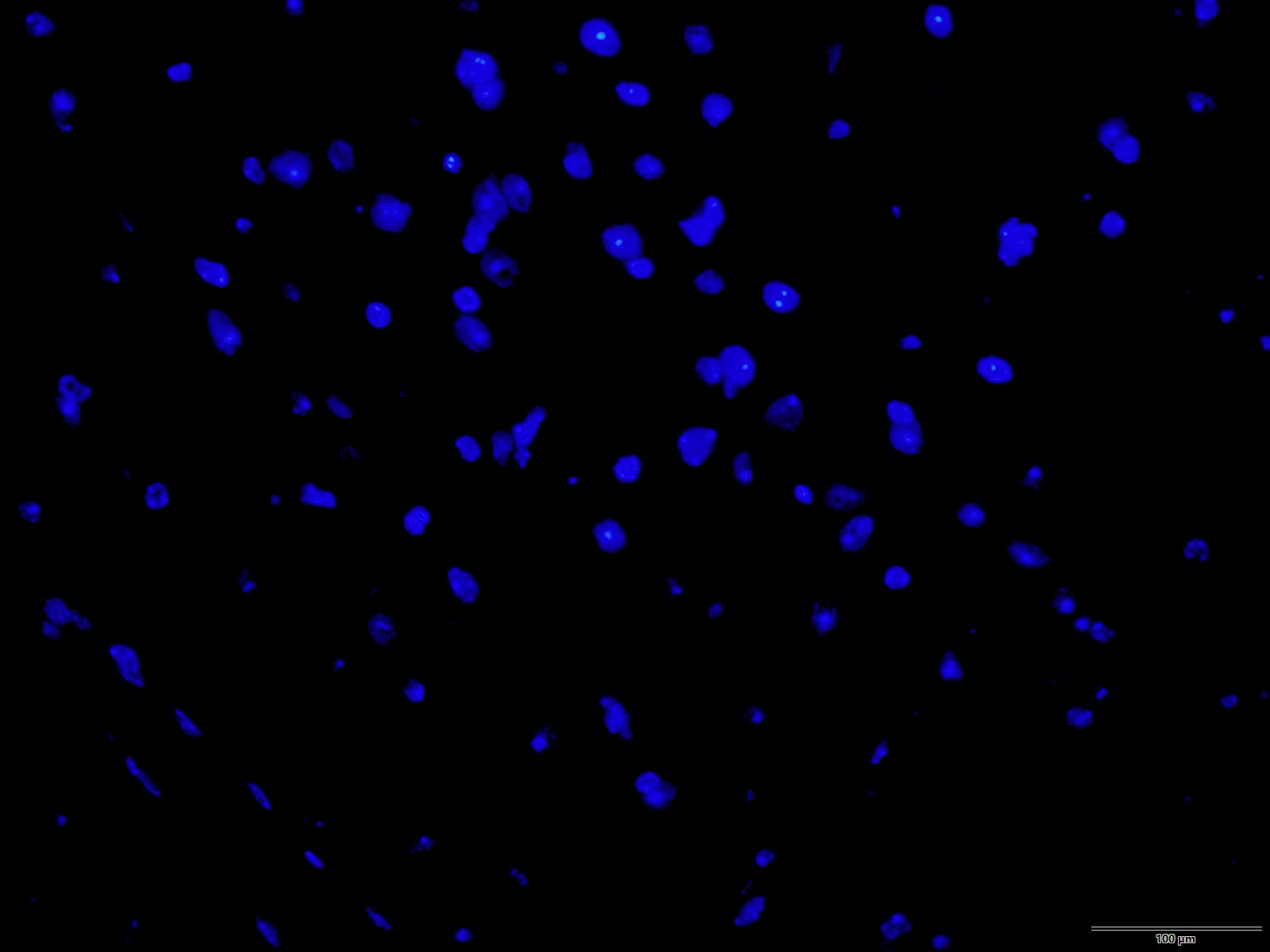

Supplement: Supplementary file 6 [file DataSheet2.ZIP › immunofluorescence of GLUT4/part 2 experiment/3-MA/2-1.jpg]

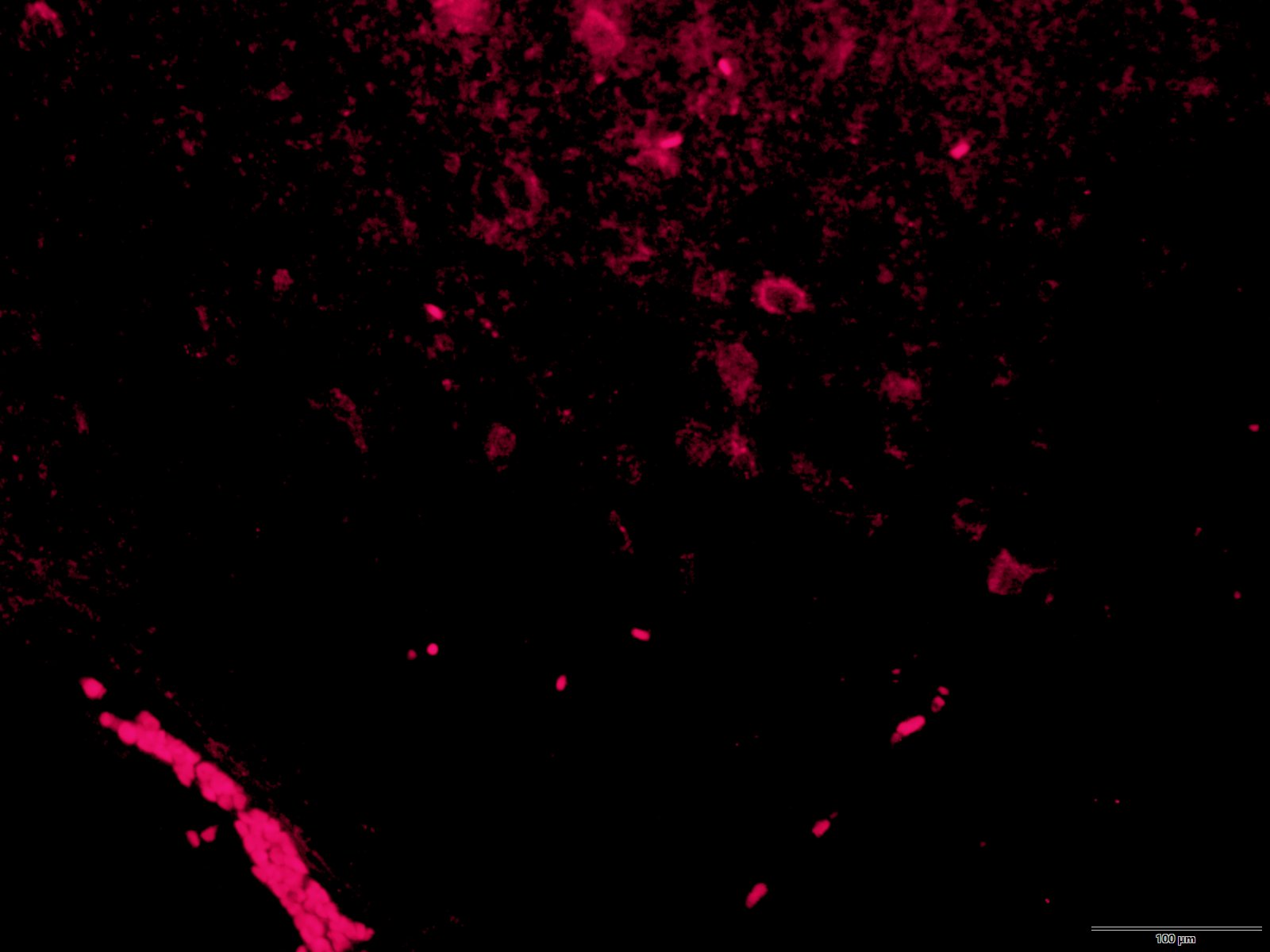

Supplement: Supplementary file 6 [file DataSheet2.ZIP › immunofluorescence of GLUT4/part 2 experiment/3-MA/2-2.jpg]

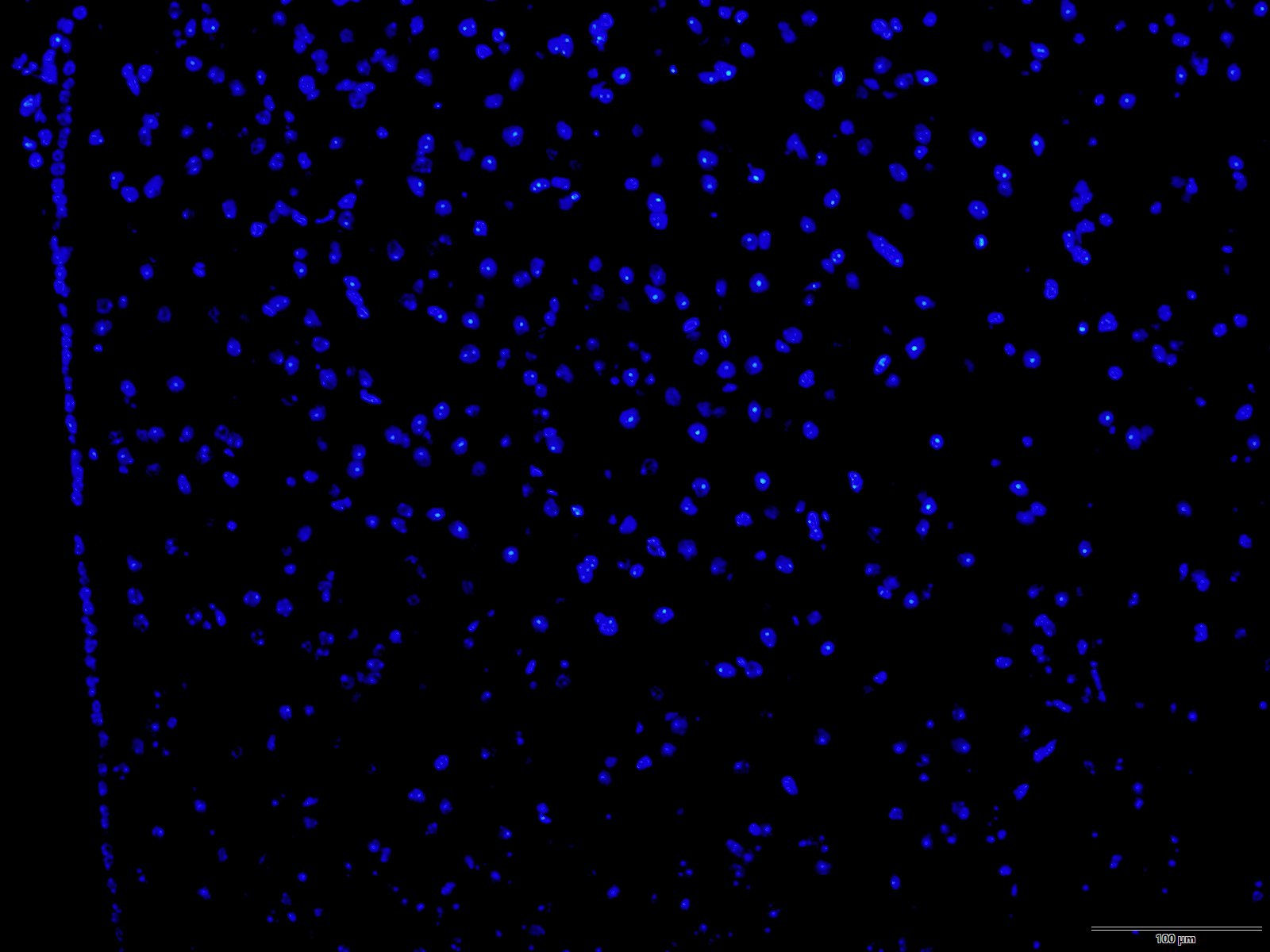

Supplement: Supplementary file 6 [file DataSheet2.ZIP › immunofluorescence of GLUT4/part 2 experiment/3-MA/3-1.jpg]

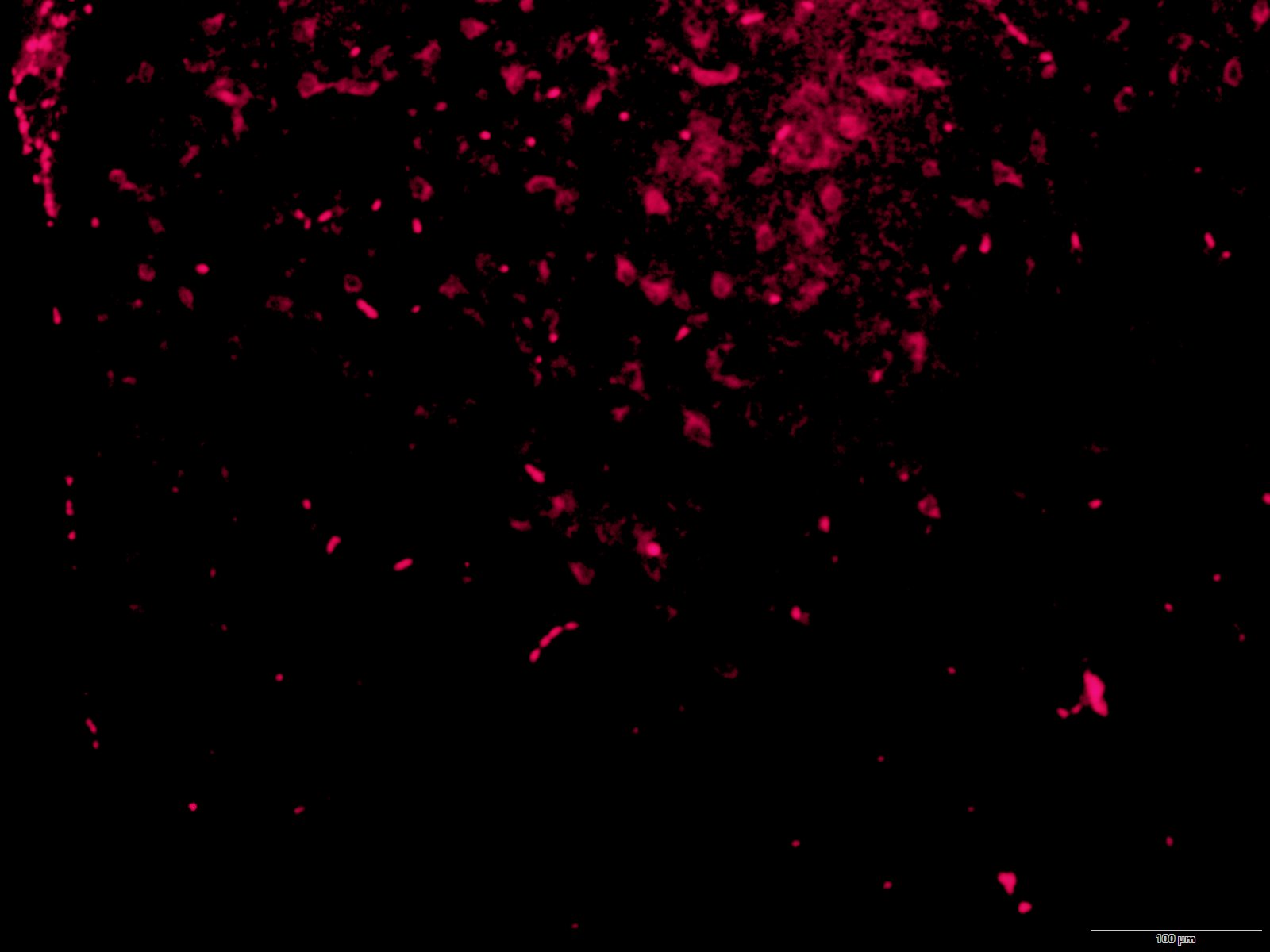

Supplement: Supplementary file 6 [file DataSheet2.ZIP › immunofluorescence of GLUT4/part 2 experiment/3-MA/3-2.jpg]

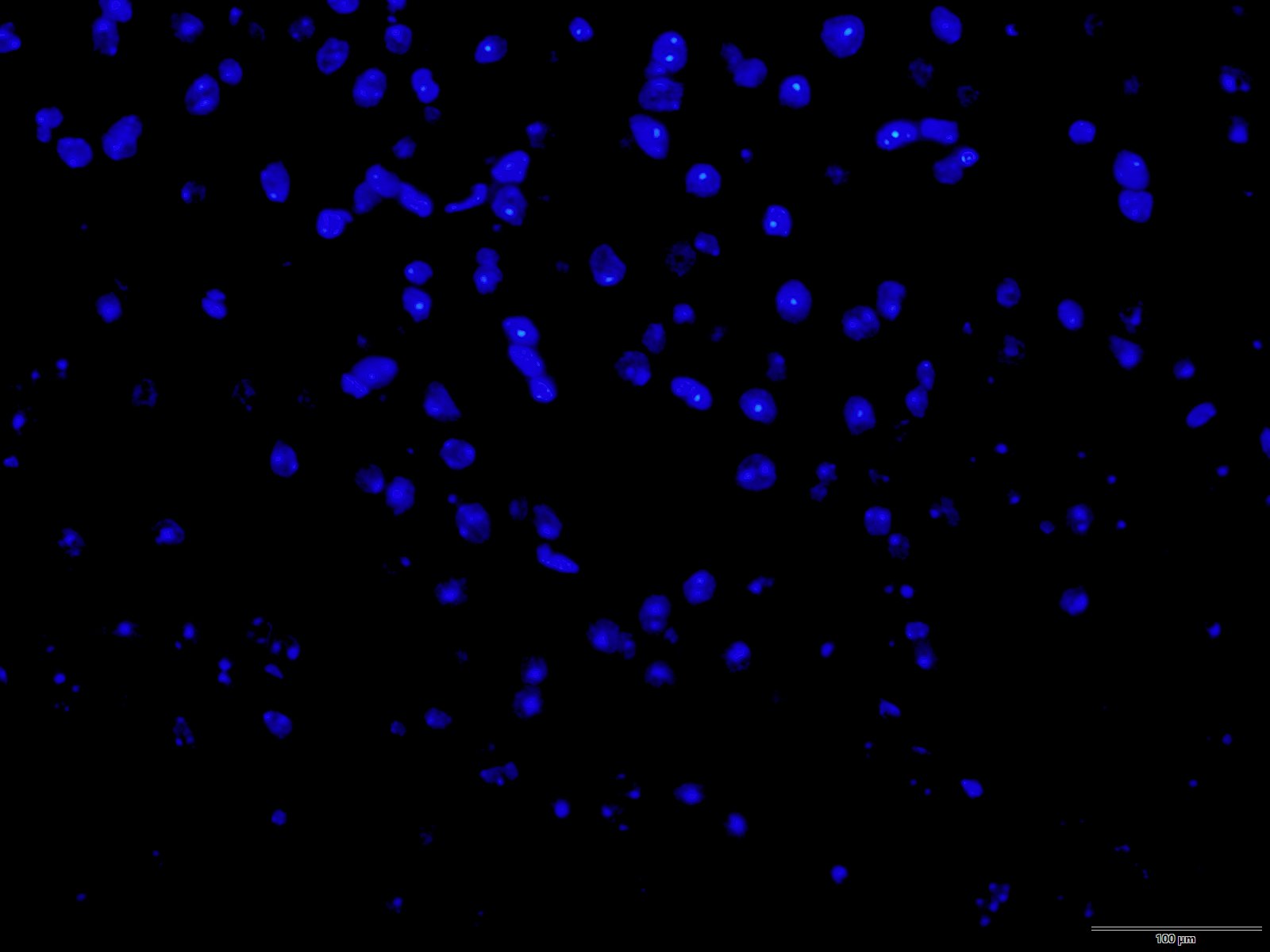

Supplement: Supplementary file 6 [file DataSheet2.ZIP › immunofluorescence of GLUT4/part 2 experiment/3-MA/4-1.jpg]

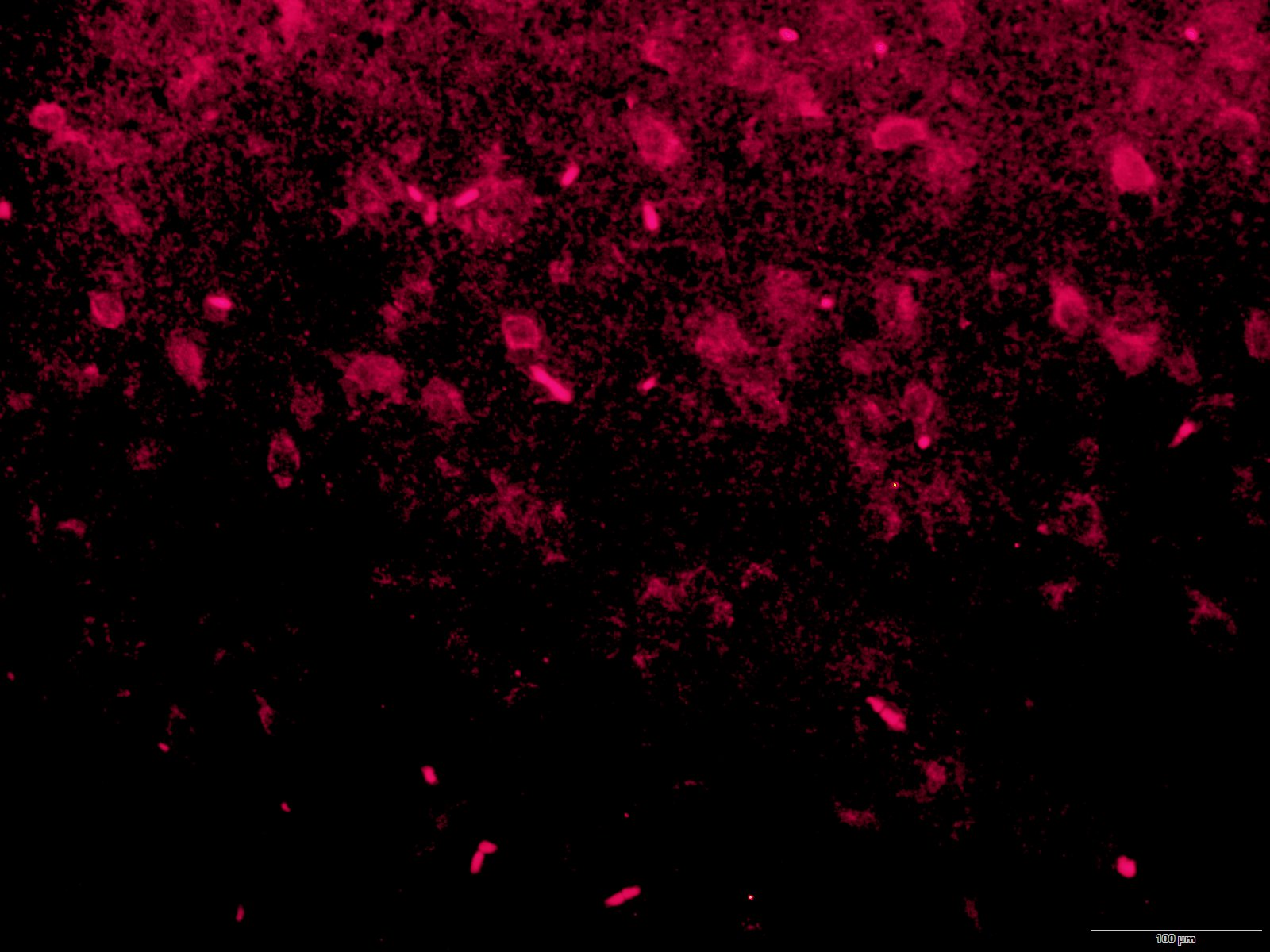

Supplement: Supplementary file 6 [file DataSheet2.ZIP › immunofluorescence of GLUT4/part 2 experiment/3-MA/4-2.jpg]

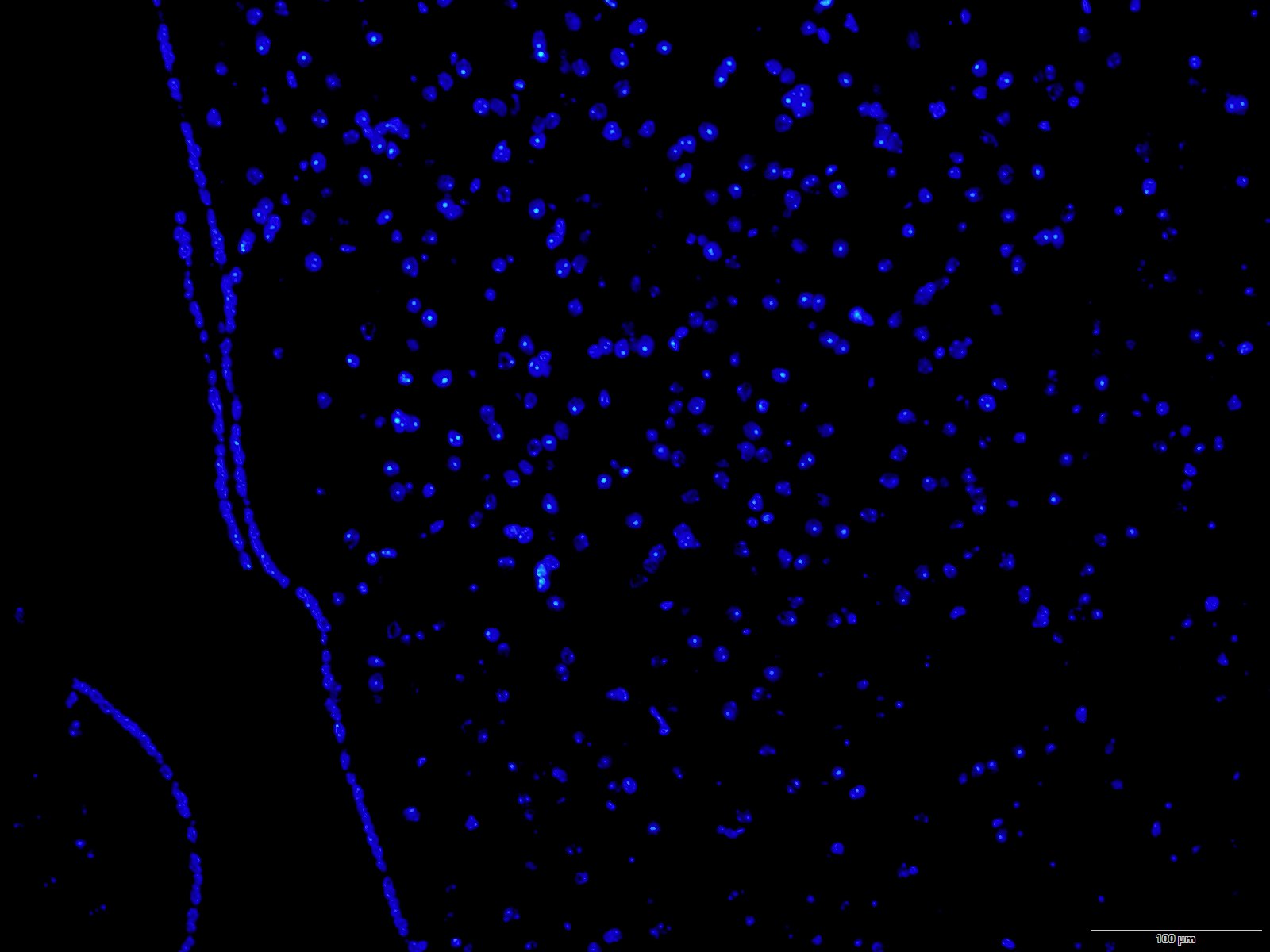

Supplement: Supplementary file 6 [file DataSheet2.ZIP › immunofluorescence of GLUT4/part 2 experiment/3-MA/6-1.jpg]

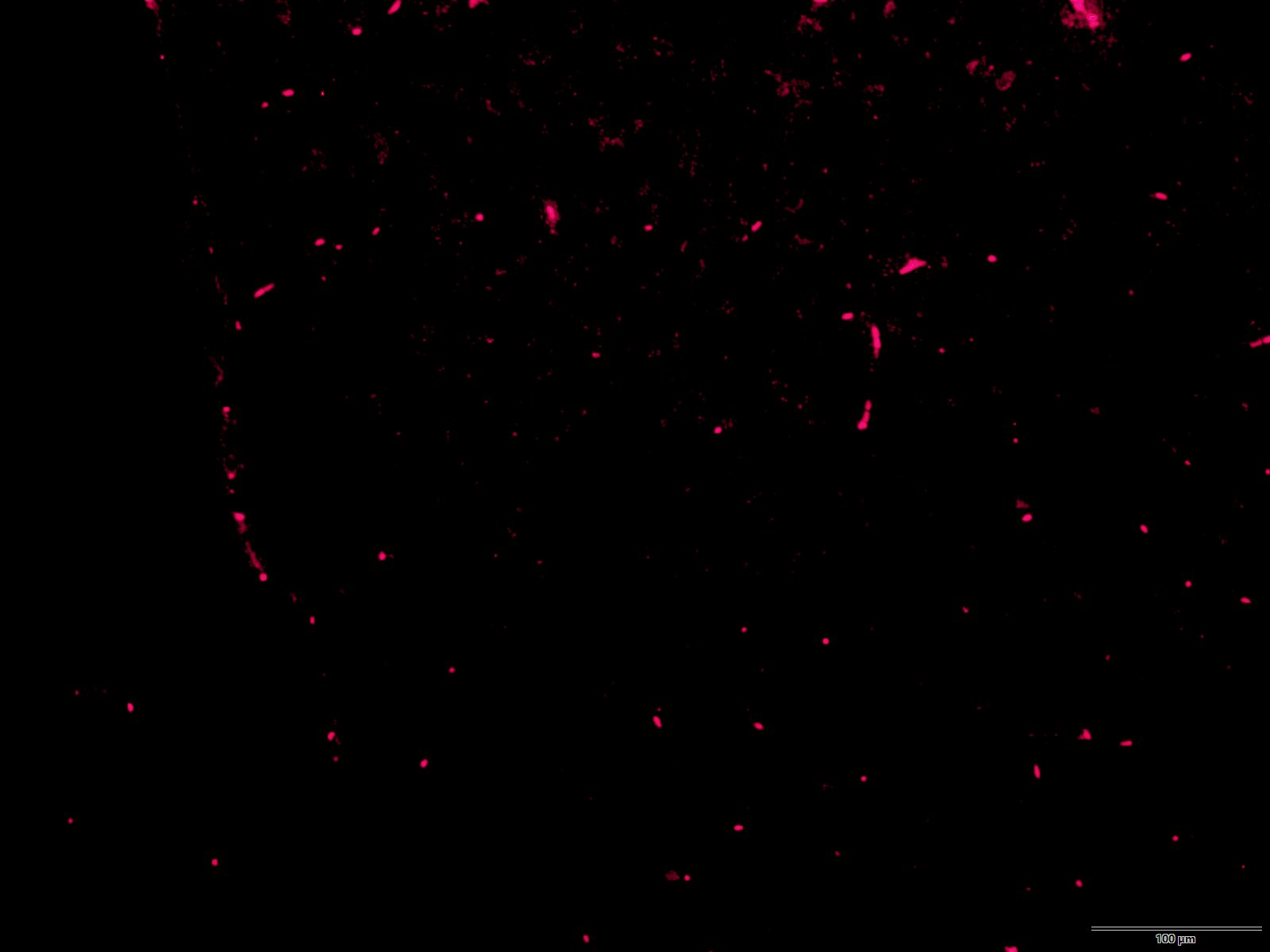

Supplement: Supplementary file 6 [file DataSheet2.ZIP › immunofluorescence of GLUT4/part 2 experiment/3-MA/6-2.jpg]

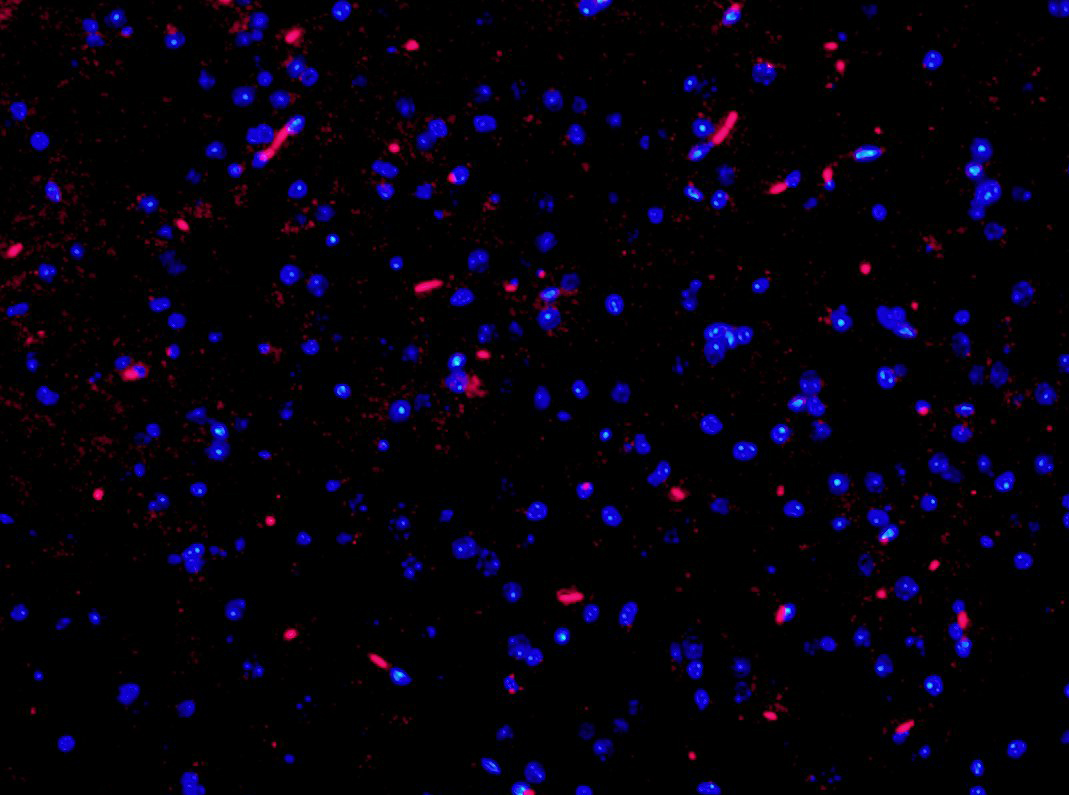

Supplement: Supplementary file 6 [file DataSheet2.ZIP › immunofluorescence of GLUT4/part 2 experiment/3-MA/M2 1.jpg]

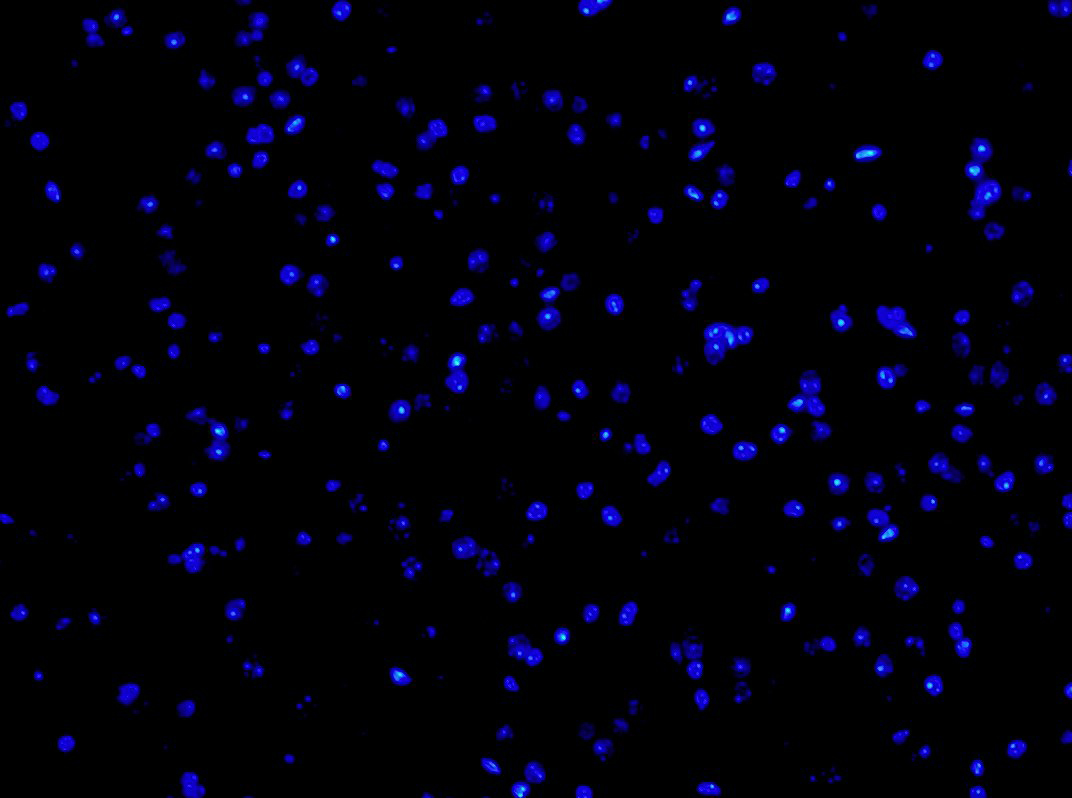

Supplement: Supplementary file 6 [file DataSheet2.ZIP › immunofluorescence of GLUT4/part 2 experiment/3-MA/M2 2.jpg]

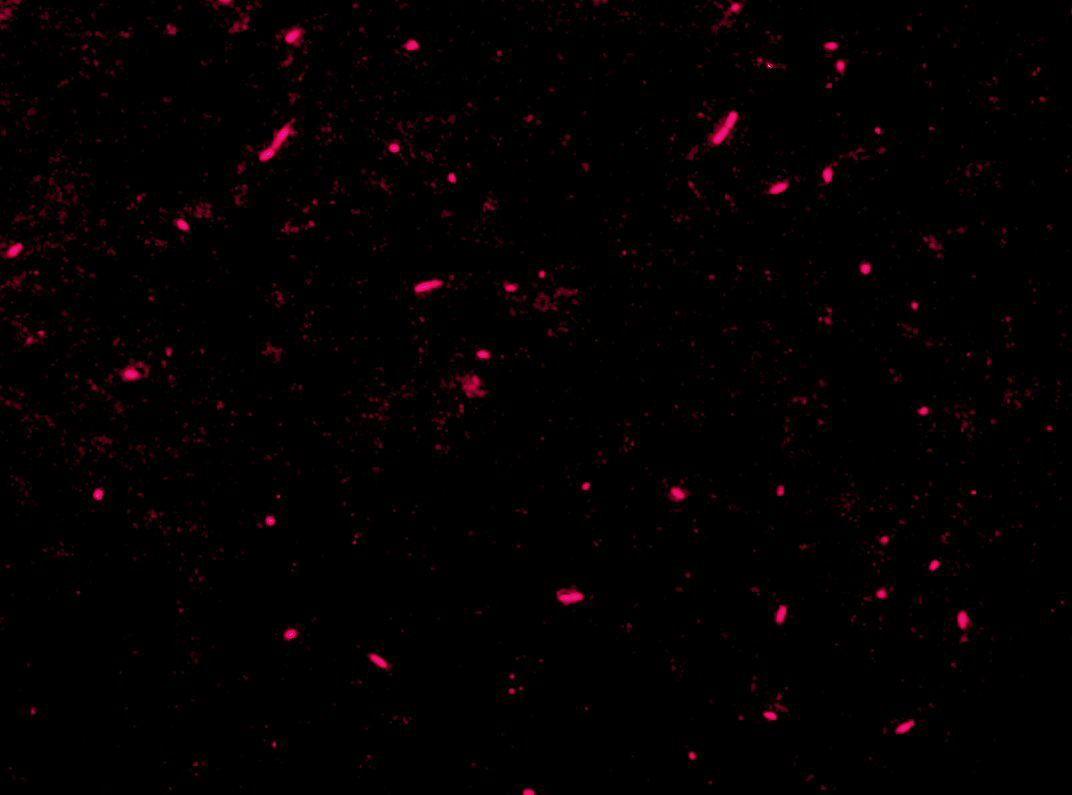

Supplement: Supplementary file 6 [file DataSheet2.ZIP › immunofluorescence of GLUT4/part 2 experiment/3-MA/M2 3.jpg]

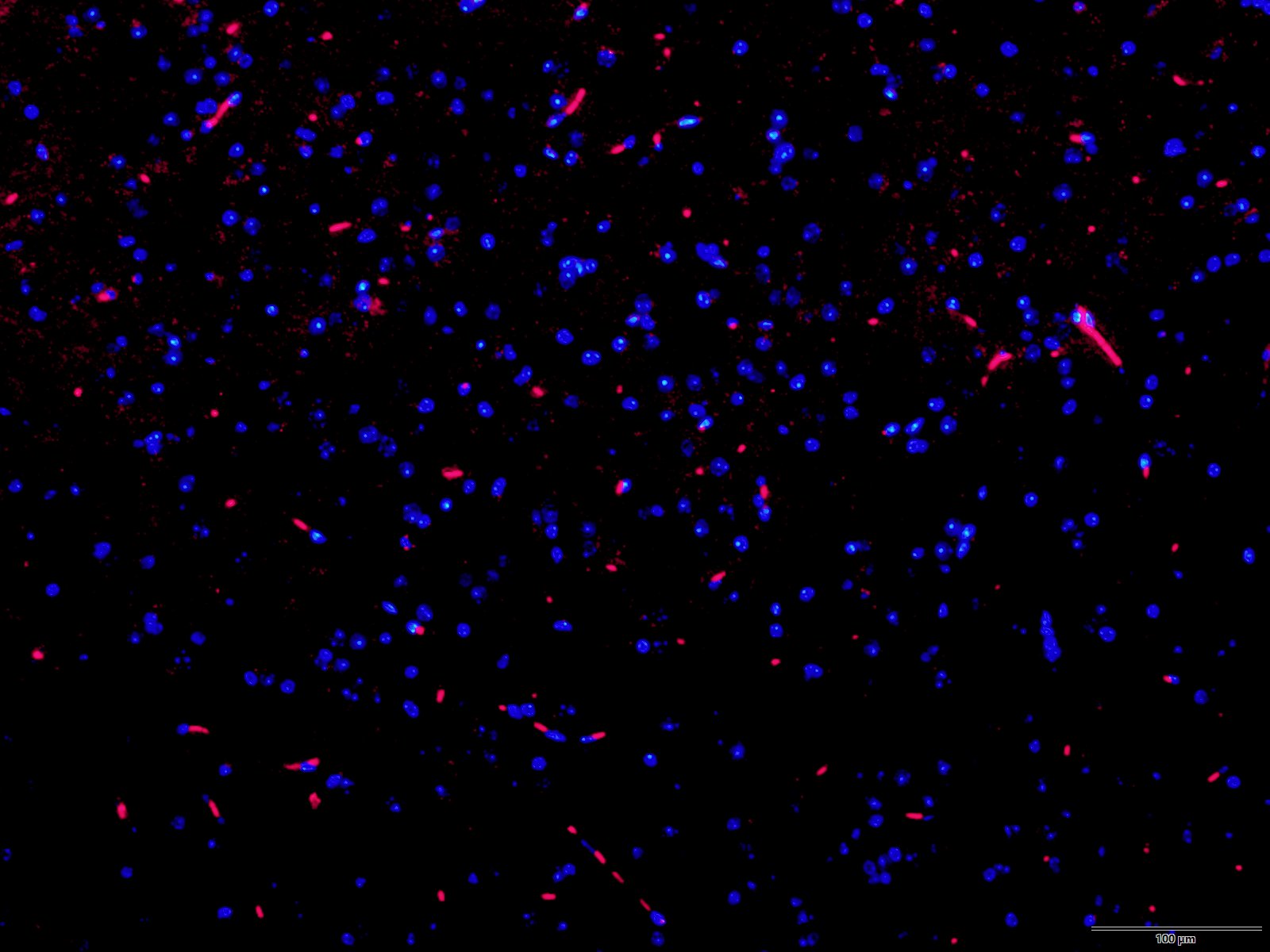

Supplement: Supplementary file 6 [file DataSheet2.ZIP › immunofluorescence of GLUT4/part 2 experiment/3-MA/M2.jpg]

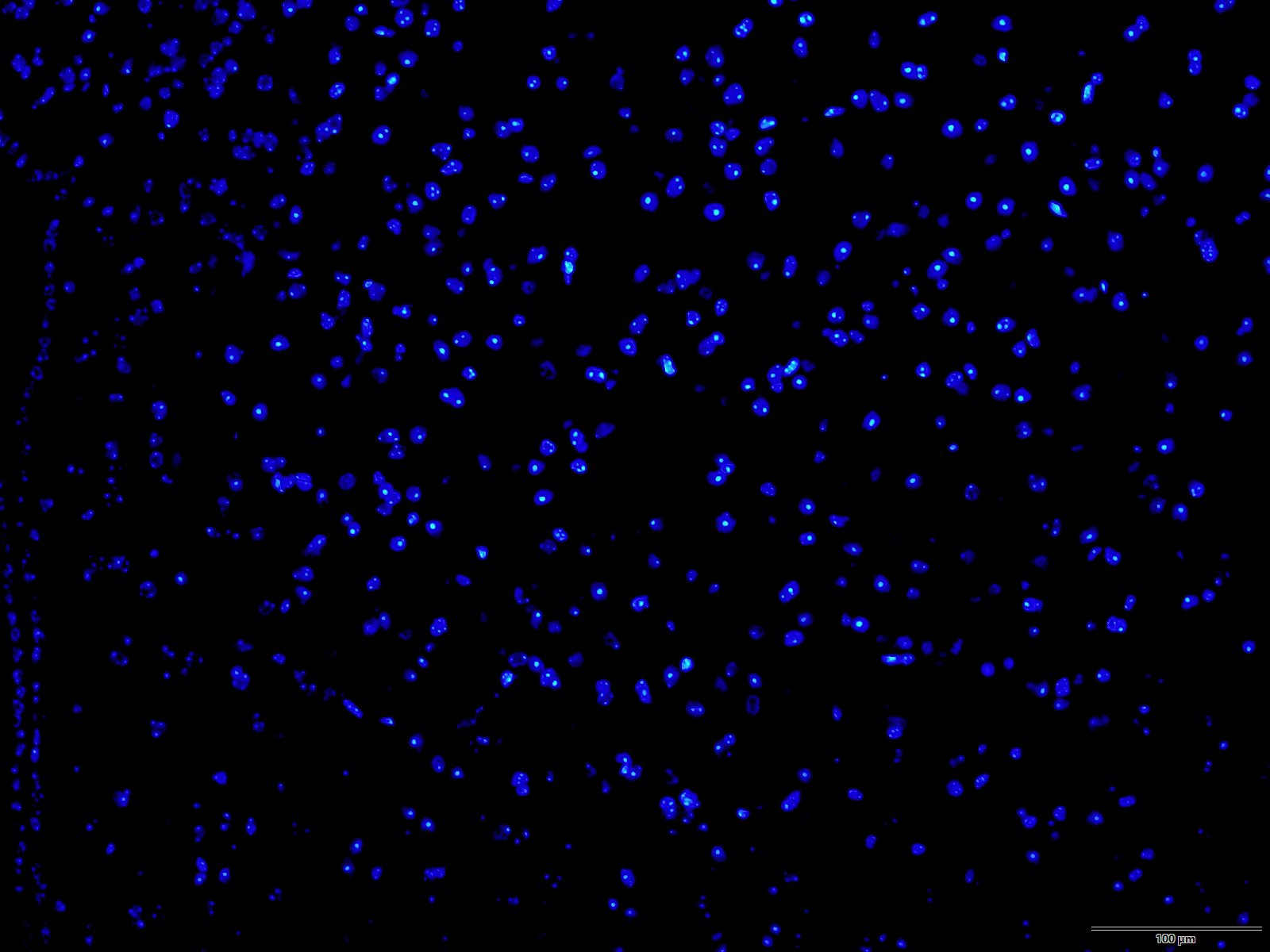

Supplement: Supplementary file 6 [file DataSheet2.ZIP › immunofluorescence of GLUT4/part 2 experiment/Control/1-1.jpg]

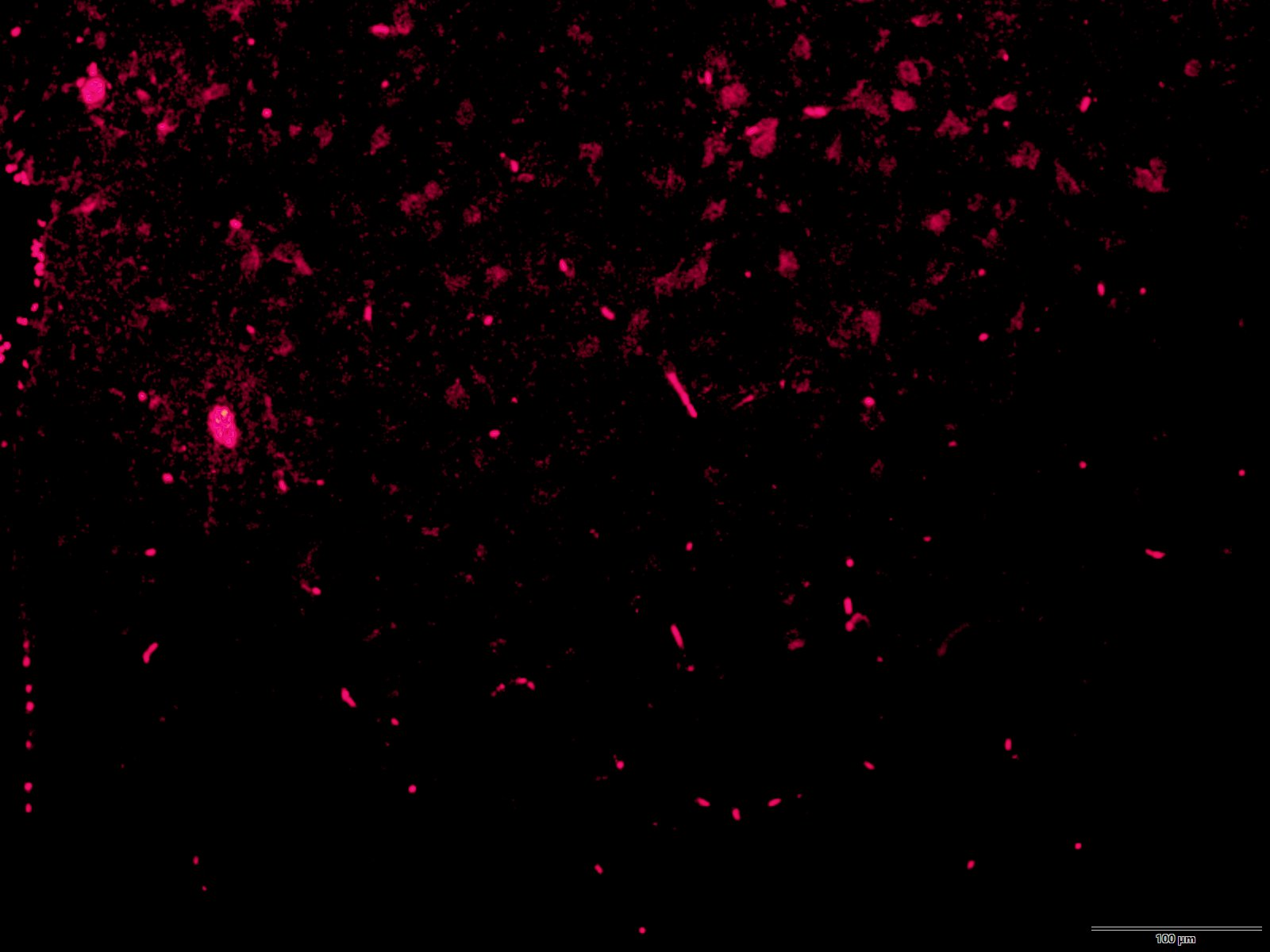

Supplement: Supplementary file 6 [file DataSheet2.ZIP › immunofluorescence of GLUT4/part 2 experiment/Control/1-2.jpg]

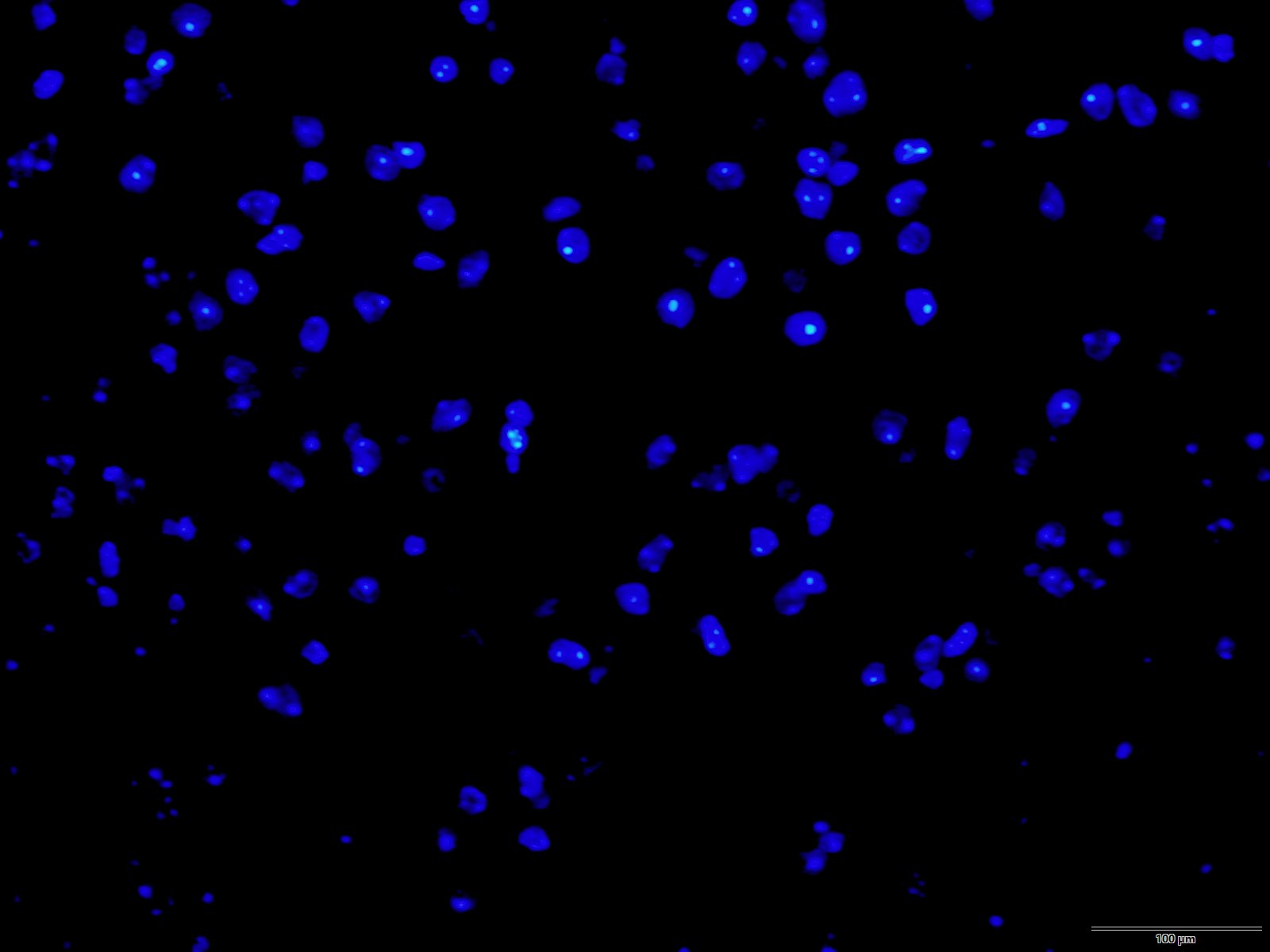

Supplement: Supplementary file 6 [file DataSheet2.ZIP › immunofluorescence of GLUT4/part 2 experiment/Control/2-1.jpg]

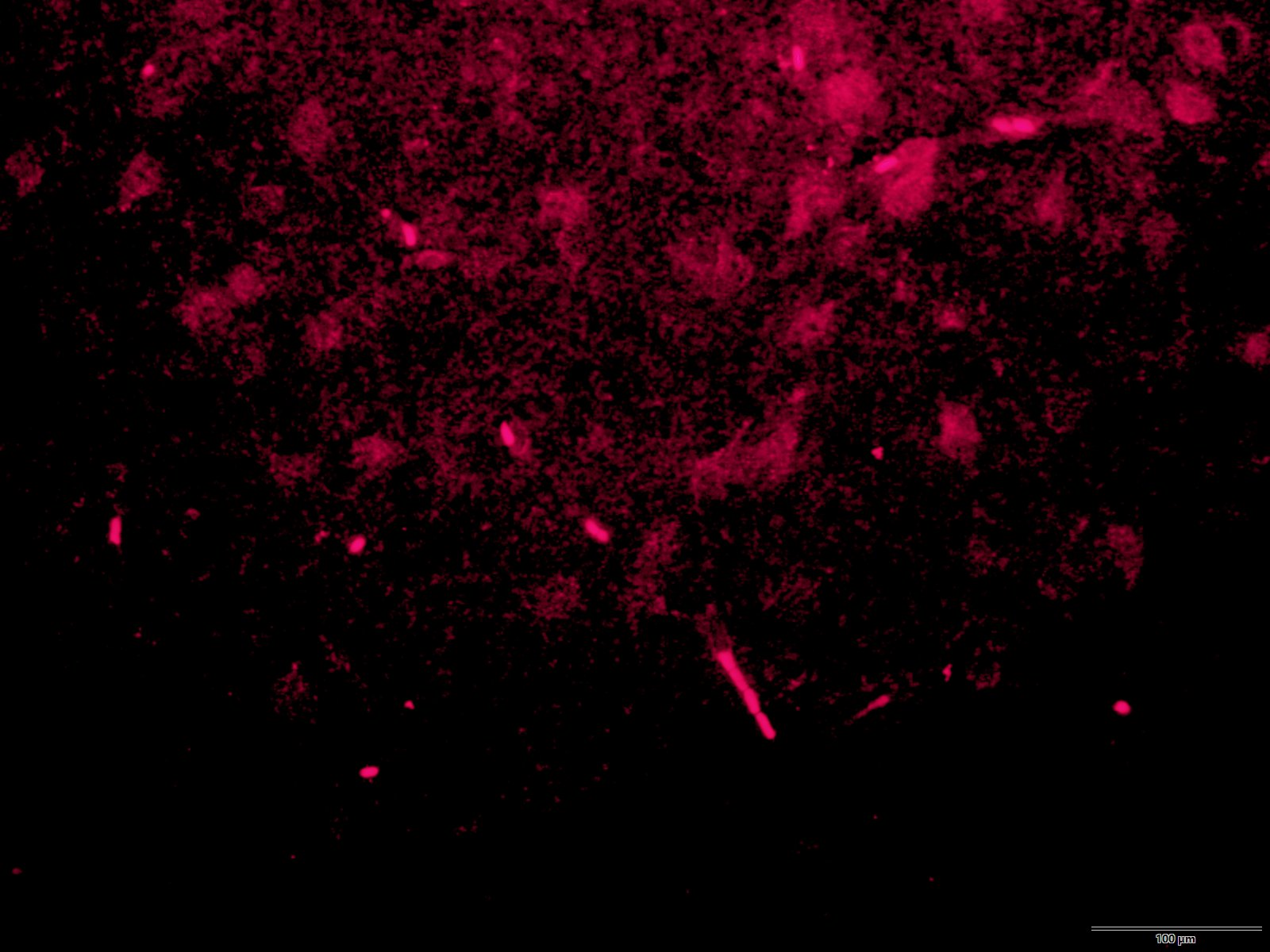

Supplement: Supplementary file 6 [file DataSheet2.ZIP › immunofluorescence of GLUT4/part 2 experiment/Control/2-2.jpg]

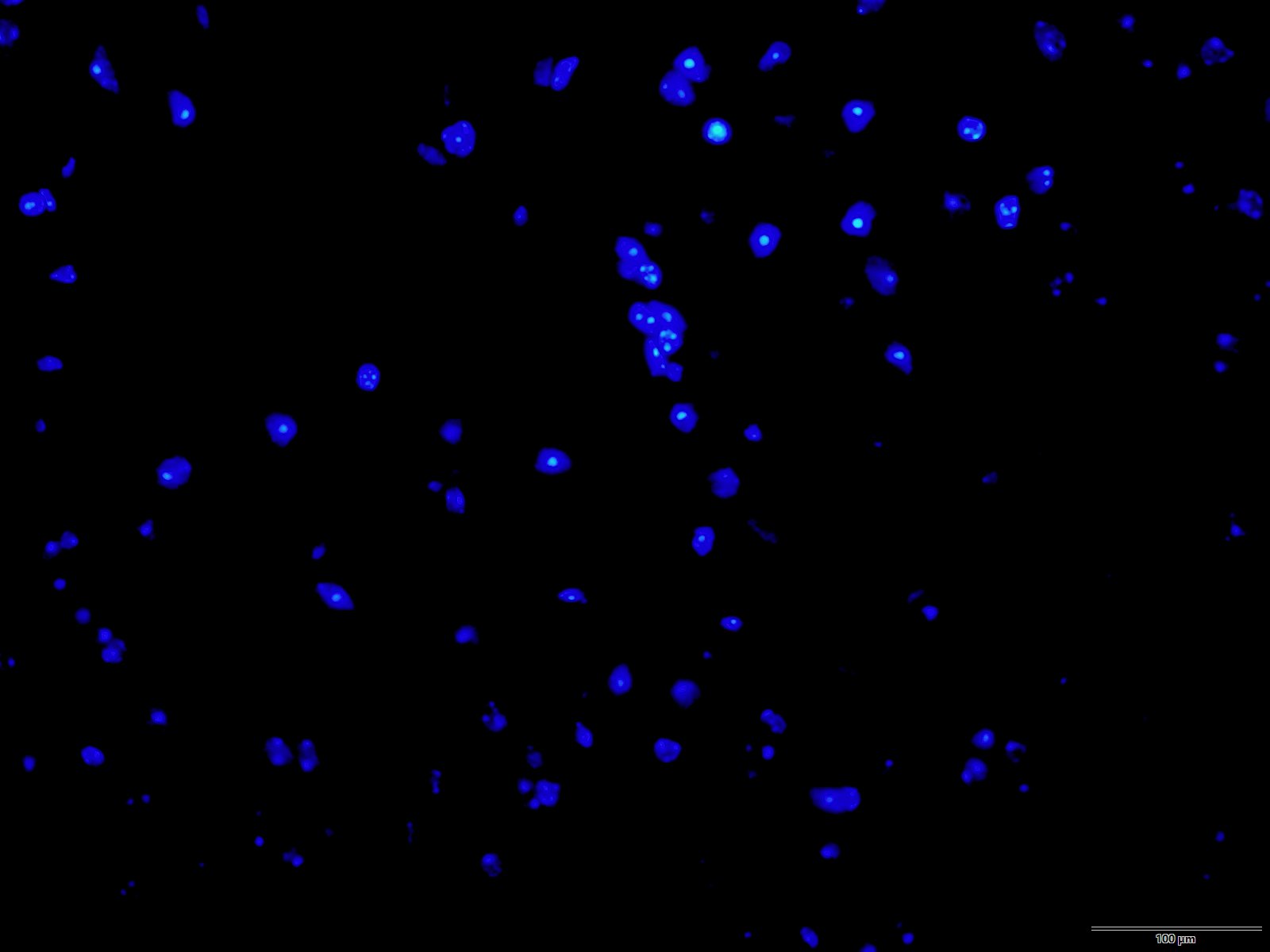

Supplement: Supplementary file 6 [file DataSheet2.ZIP › immunofluorescence of GLUT4/part 2 experiment/Control/4-1.jpg]

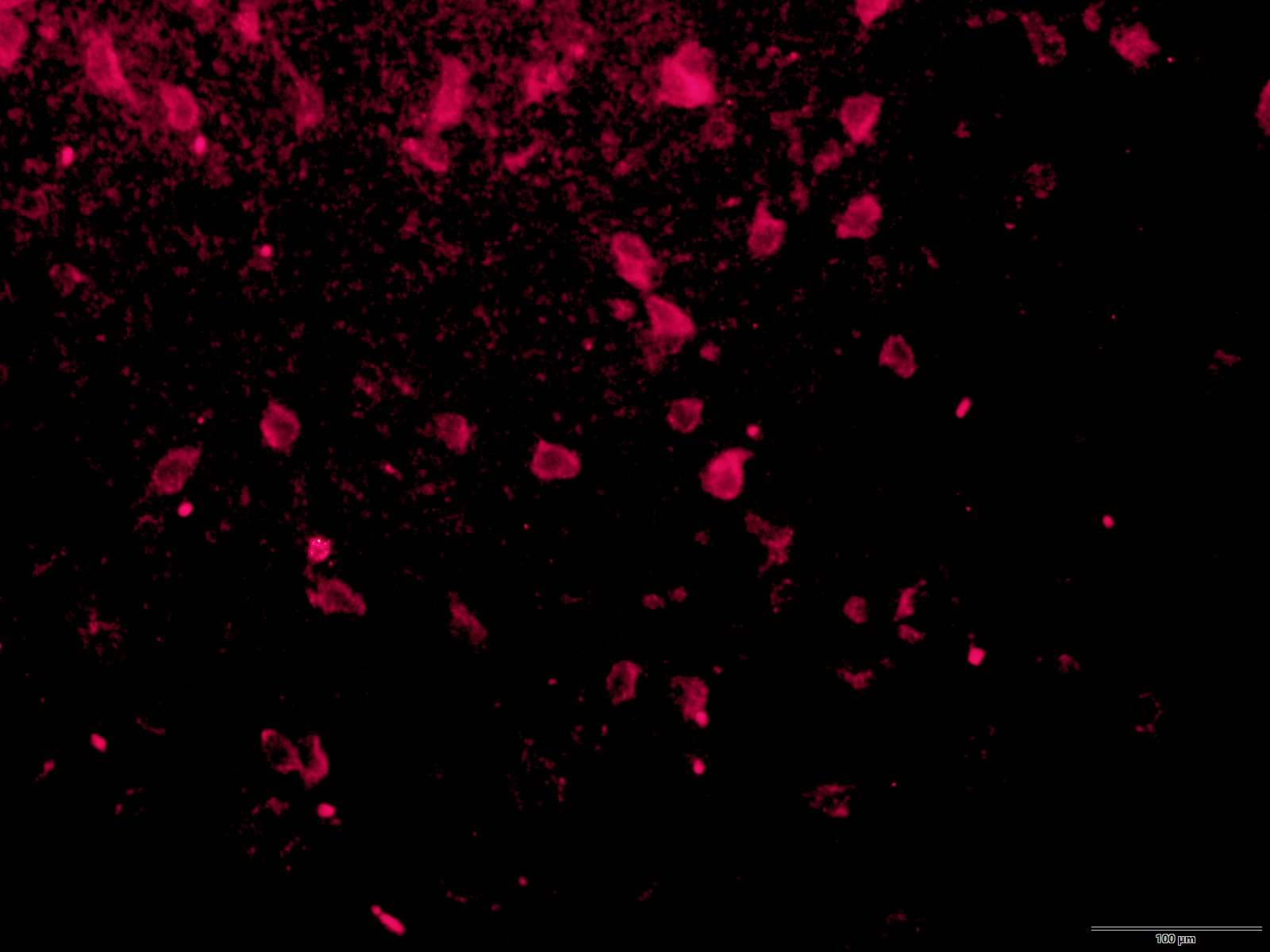

Supplement: Supplementary file 6 [file DataSheet2.ZIP › immunofluorescence of GLUT4/part 2 experiment/Control/4-2.jpg]

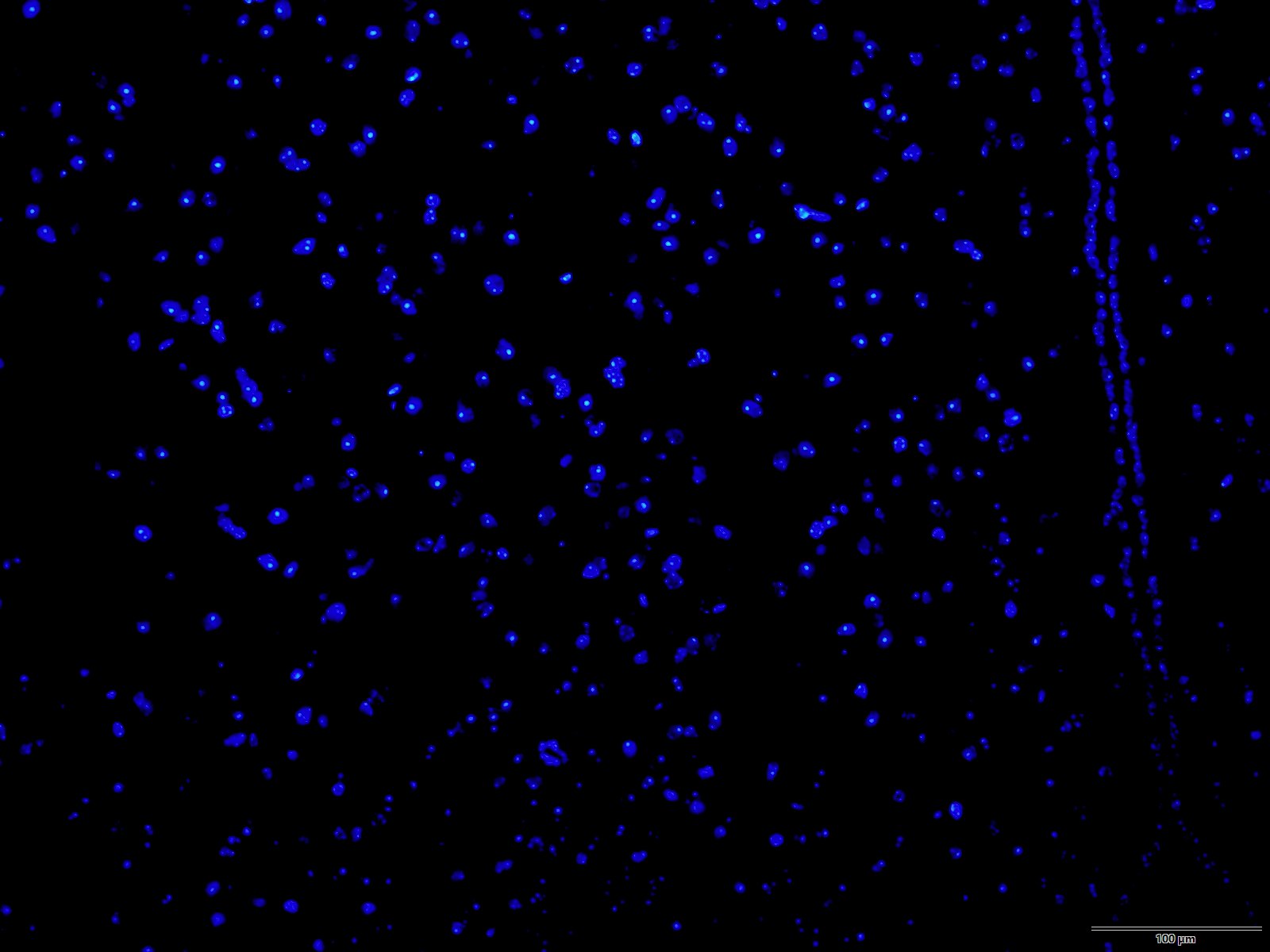

Supplement: Supplementary file 6 [file DataSheet2.ZIP › immunofluorescence of GLUT4/part 2 experiment/Control/5-1.jpg]

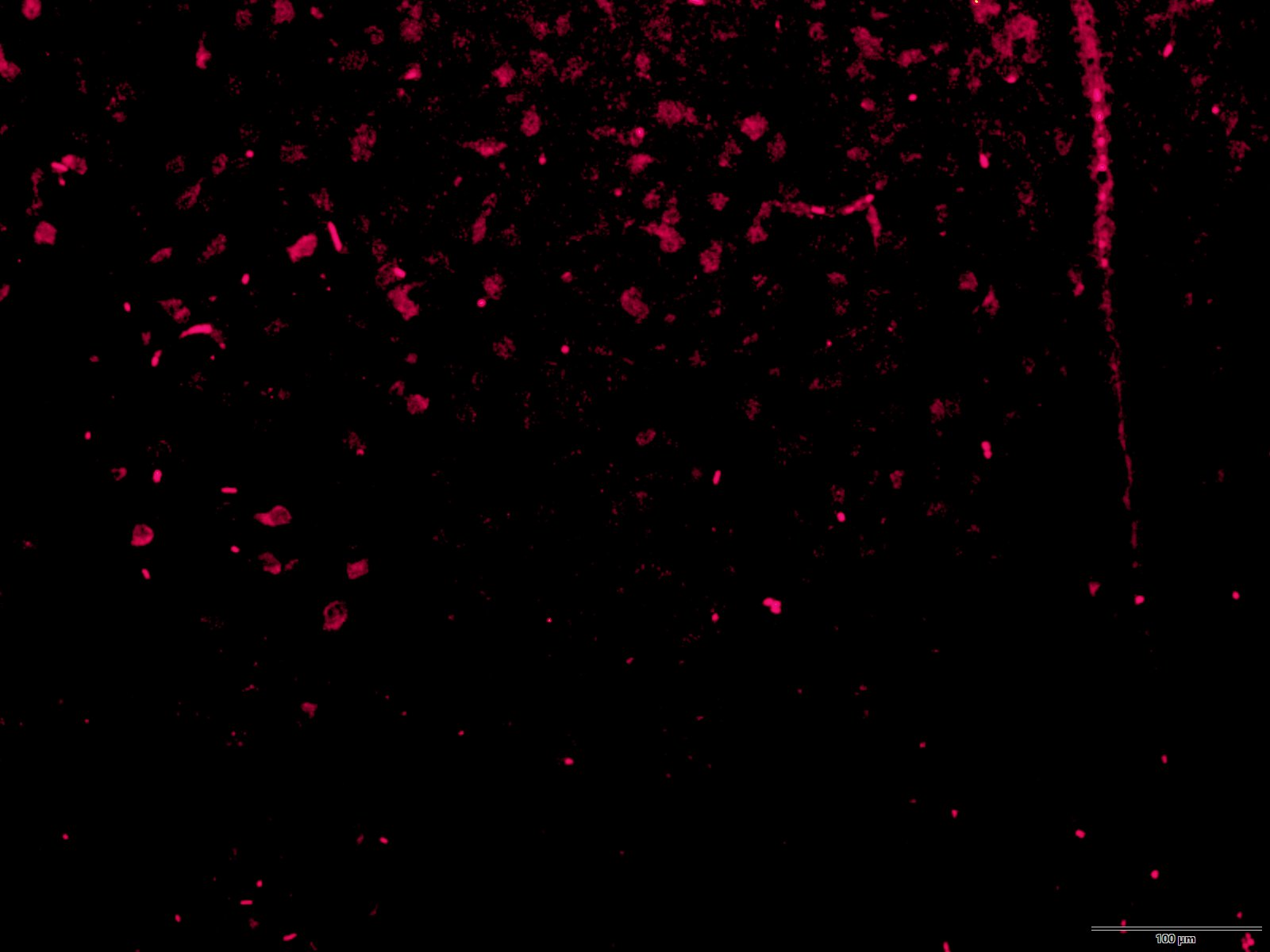

Supplement: Supplementary file 6 [file DataSheet2.ZIP › immunofluorescence of GLUT4/part 2 experiment/Control/5-2.jpg]

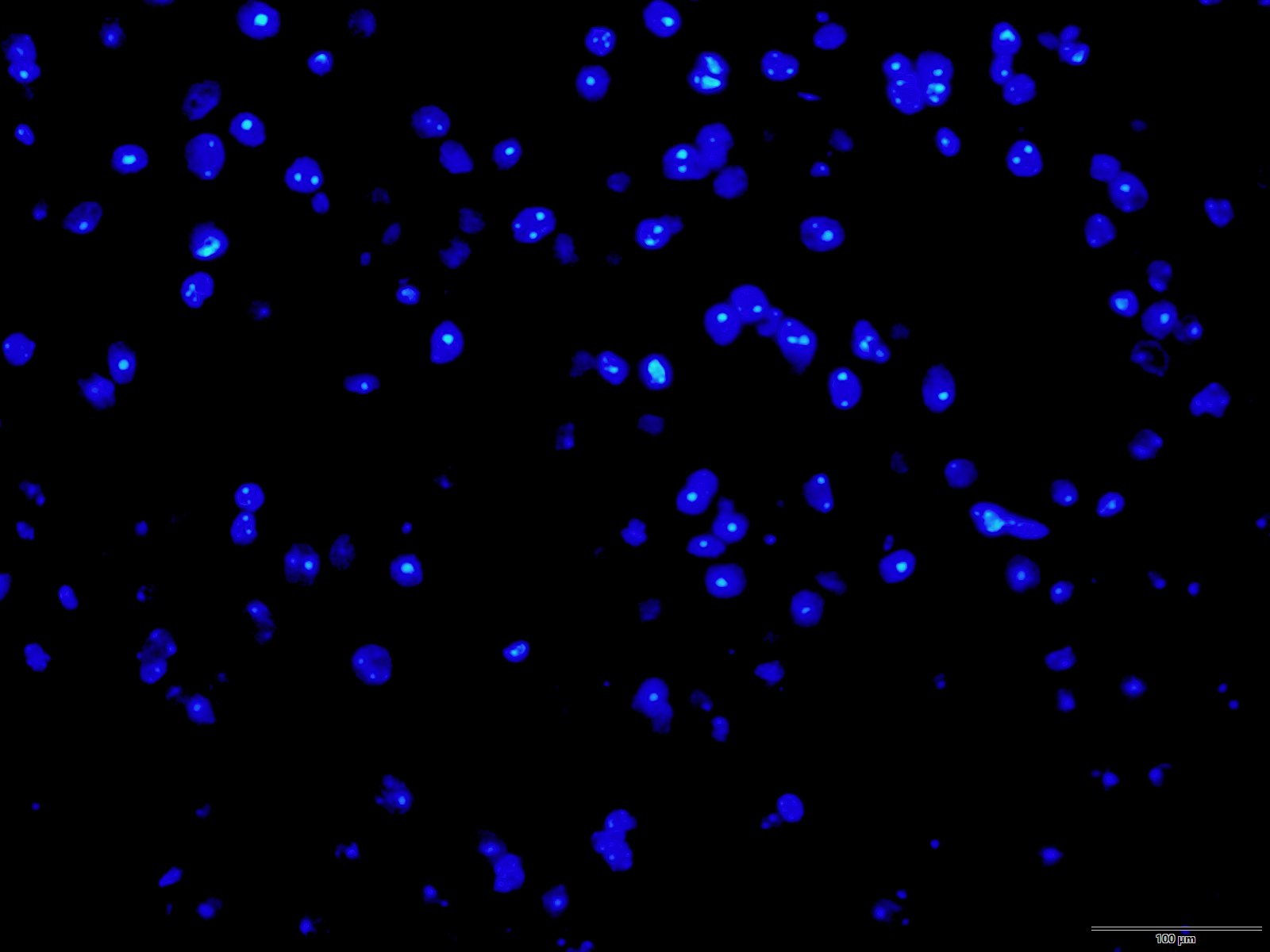

Supplement: Supplementary file 6 [file DataSheet2.ZIP › immunofluorescence of GLUT4/part 2 experiment/Control/6-1.jpg]

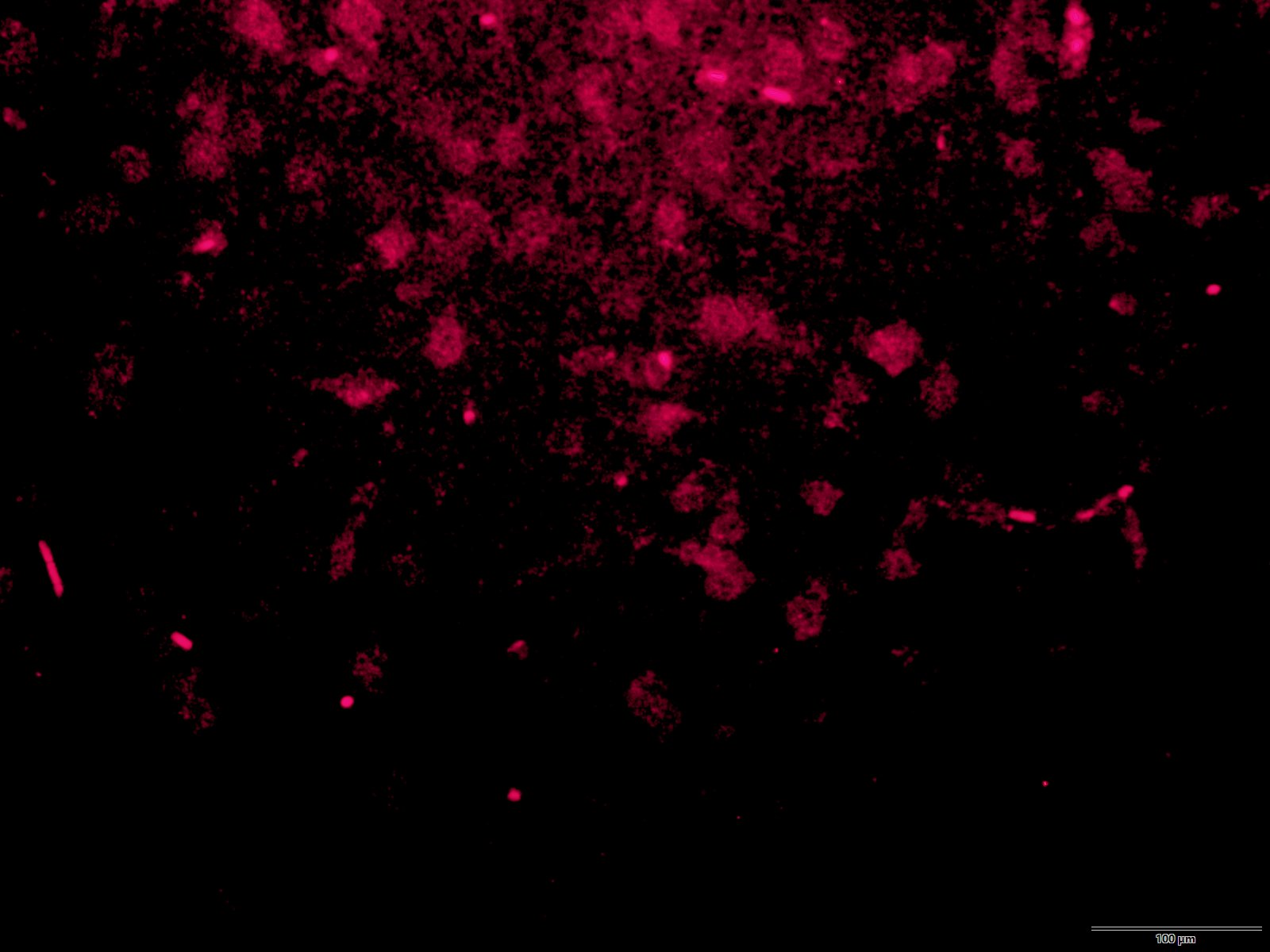

Supplement: Supplementary file 6 [file DataSheet2.ZIP › immunofluorescence of GLUT4/part 2 experiment/Control/6-2.jpg]

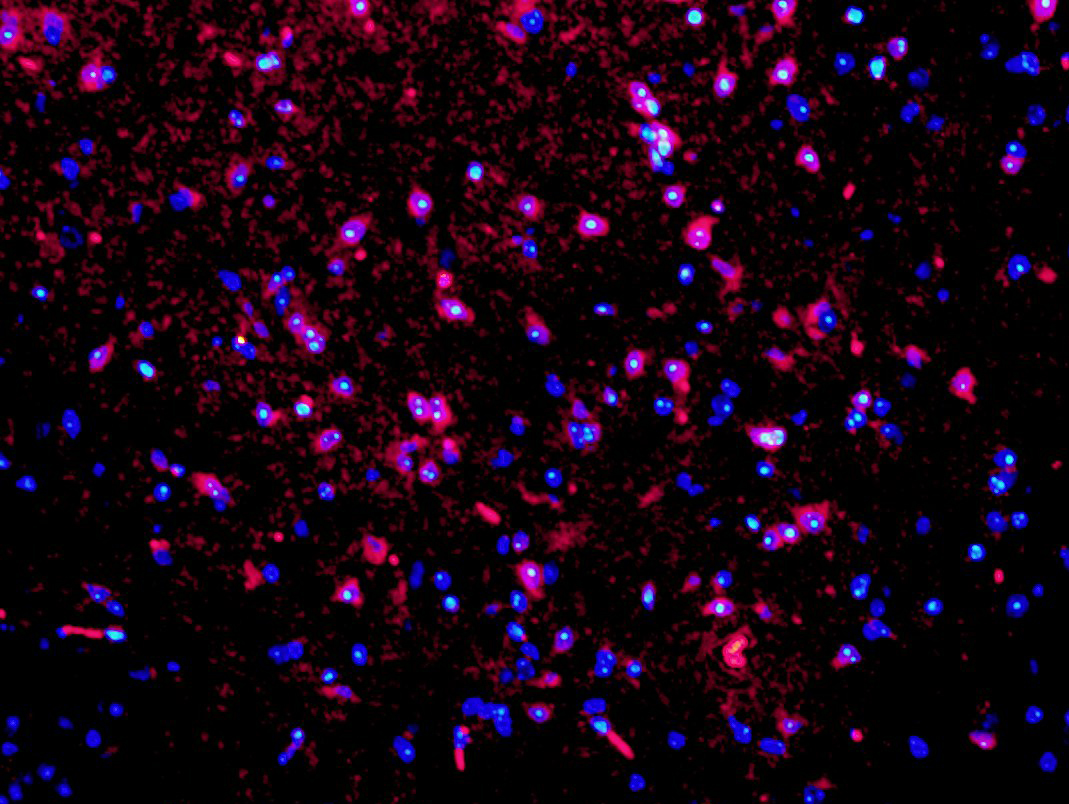

Supplement: Supplementary file 6 [file DataSheet2.ZIP › immunofluorescence of GLUT4/part 2 experiment/Control/C2 1.jpg]

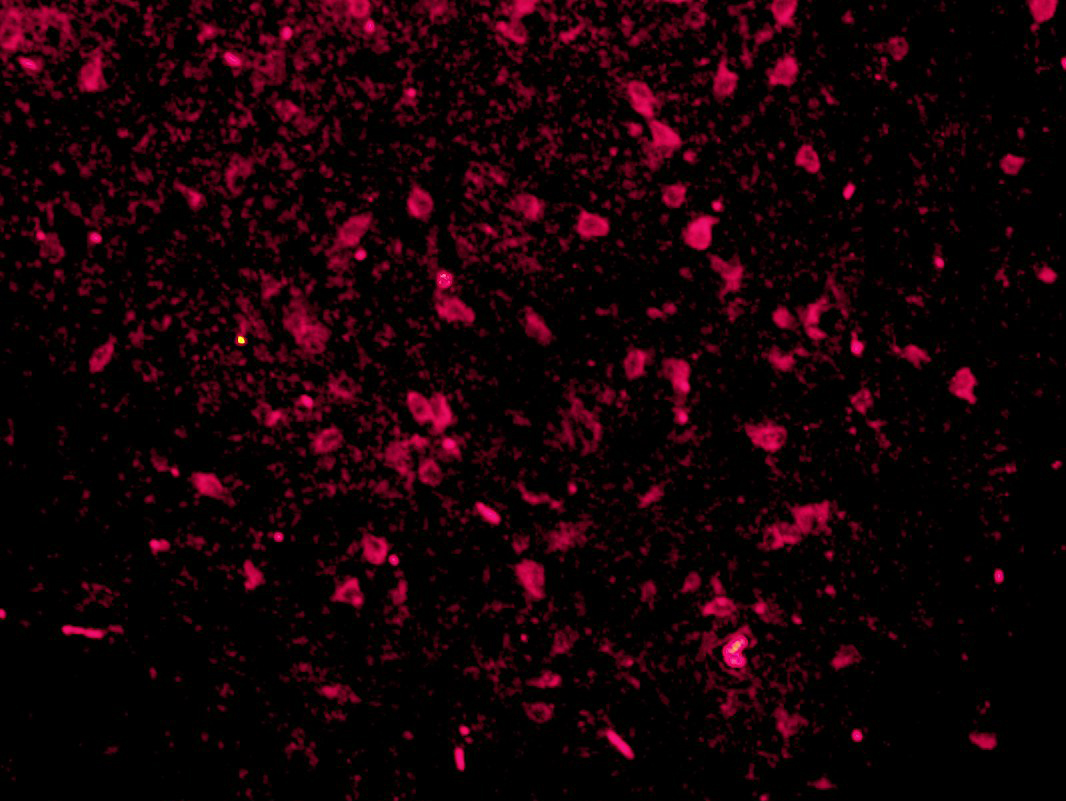

Supplement: Supplementary file 6 [file DataSheet2.ZIP › immunofluorescence of GLUT4/part 2 experiment/Control/C2 2.jpg]

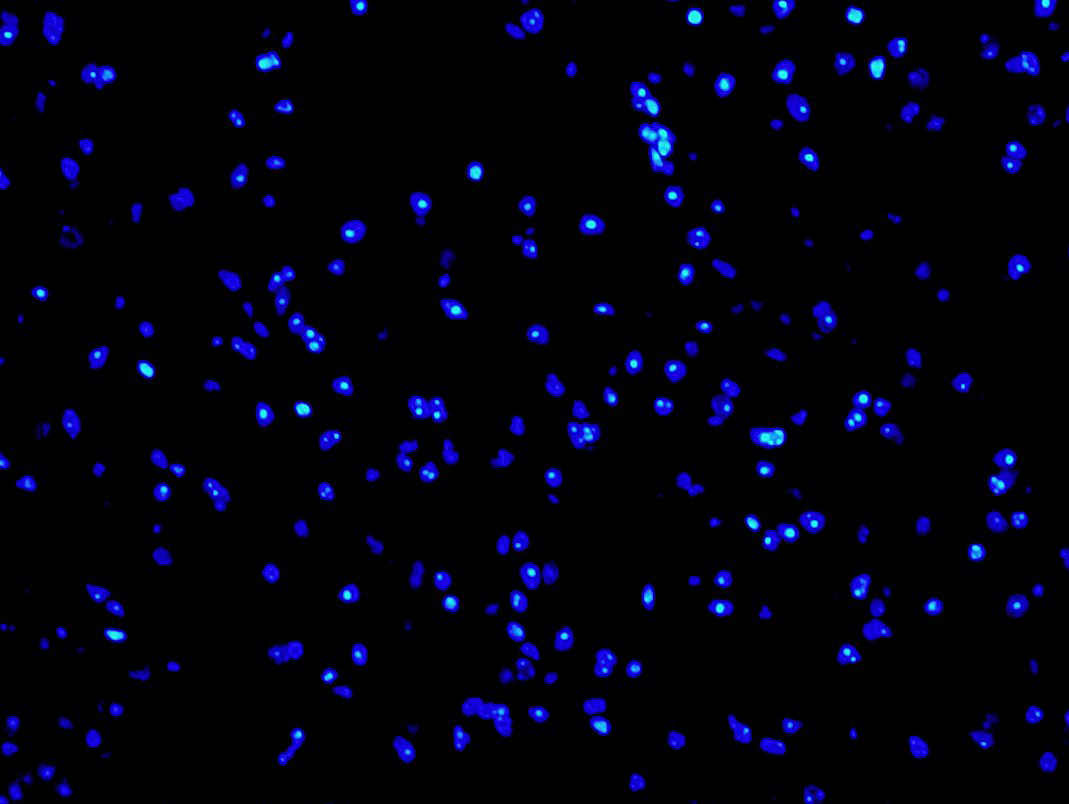

Supplement: Supplementary file 6 [file DataSheet2.ZIP › immunofluorescence of GLUT4/part 2 experiment/Control/C2 3.jpg]

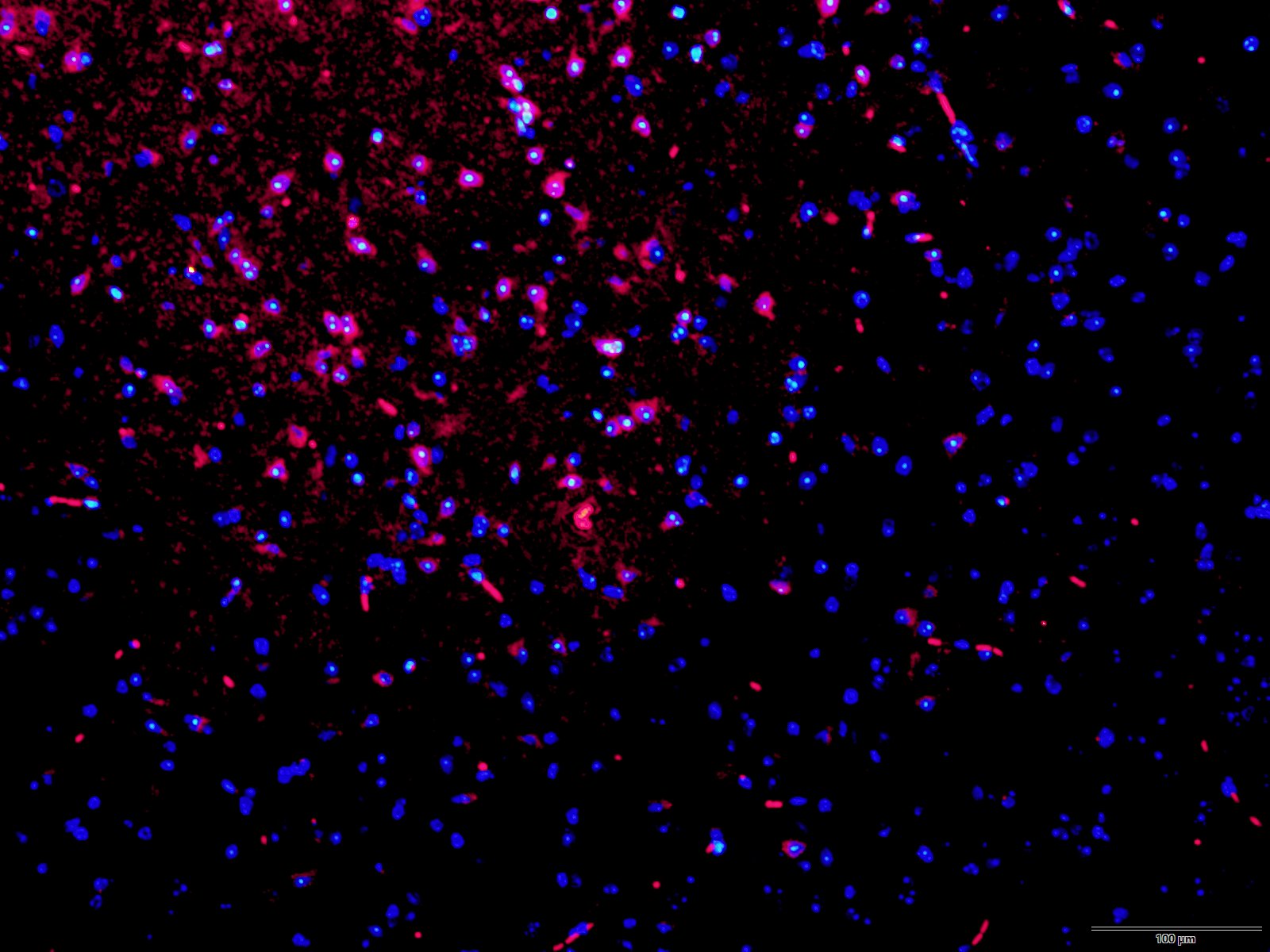

Supplement: Supplementary file 6 [file DataSheet2.ZIP › immunofluorescence of GLUT4/part 2 experiment/Control/C2.jpg]

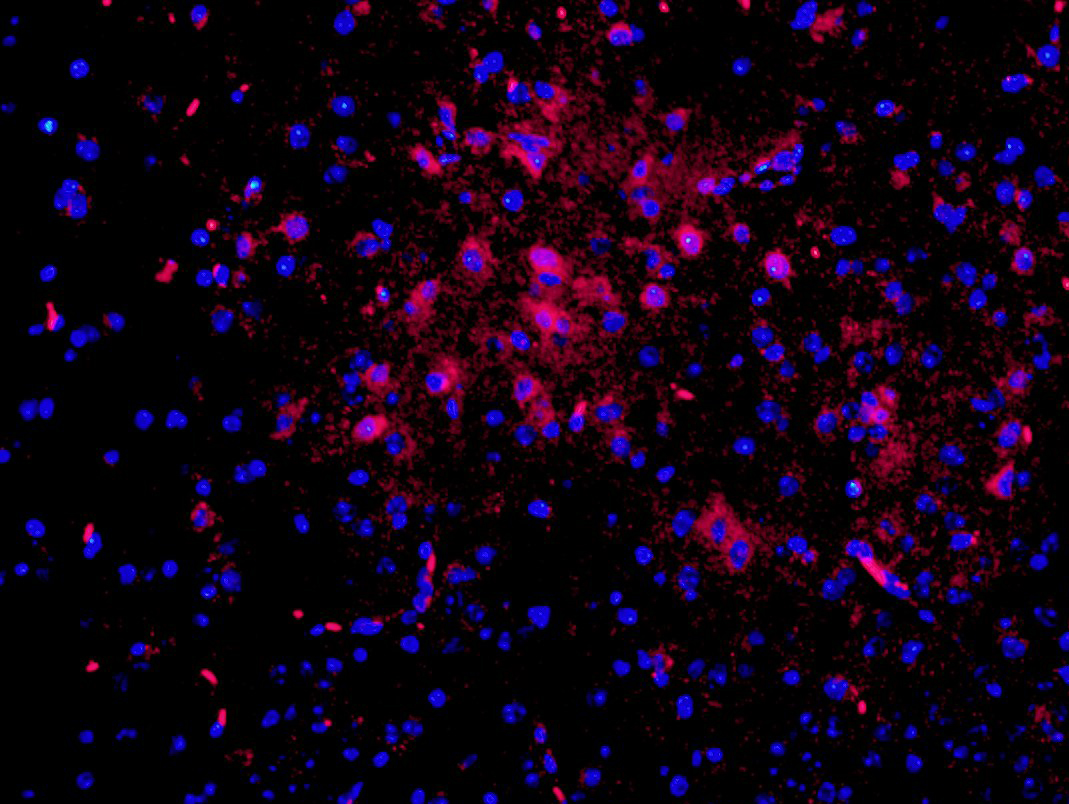

Supplement: Supplementary file 6 [file DataSheet2.ZIP › immunofluorescence of GLUT4/part 2 experiment/Sham/S2 1.jpg]

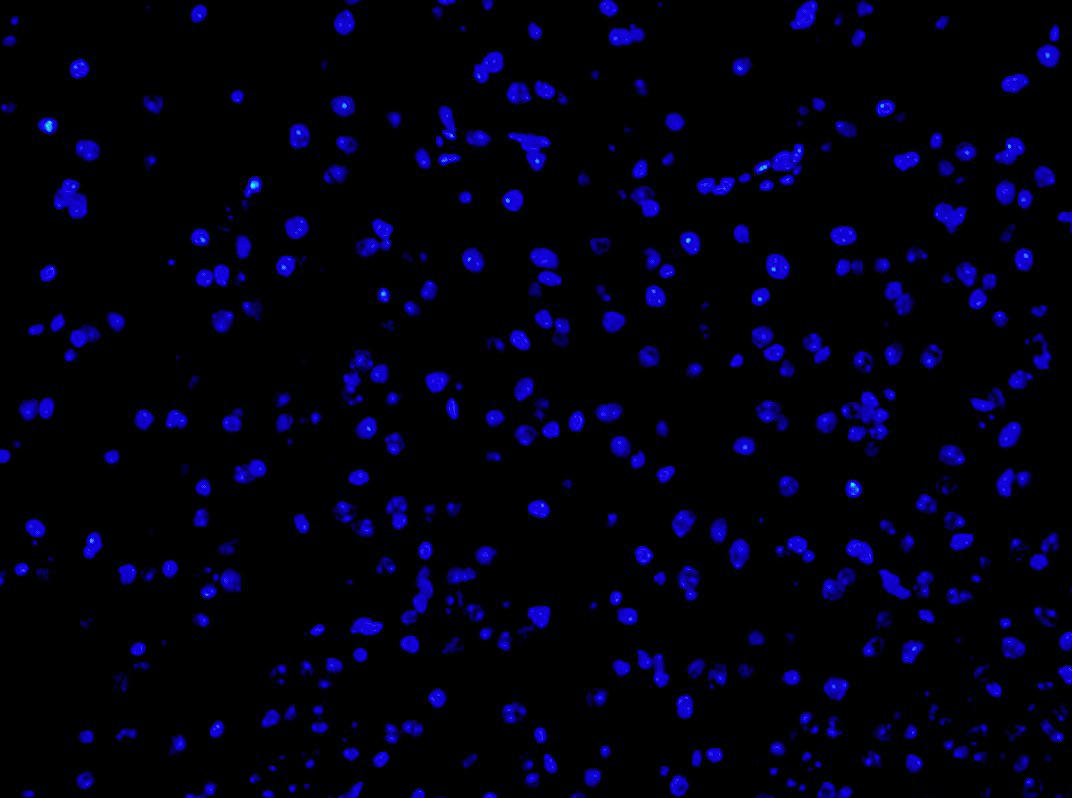

Supplement: Supplementary file 6 [file DataSheet2.ZIP › immunofluorescence of GLUT4/part 2 experiment/Sham/S2 2.jpg]

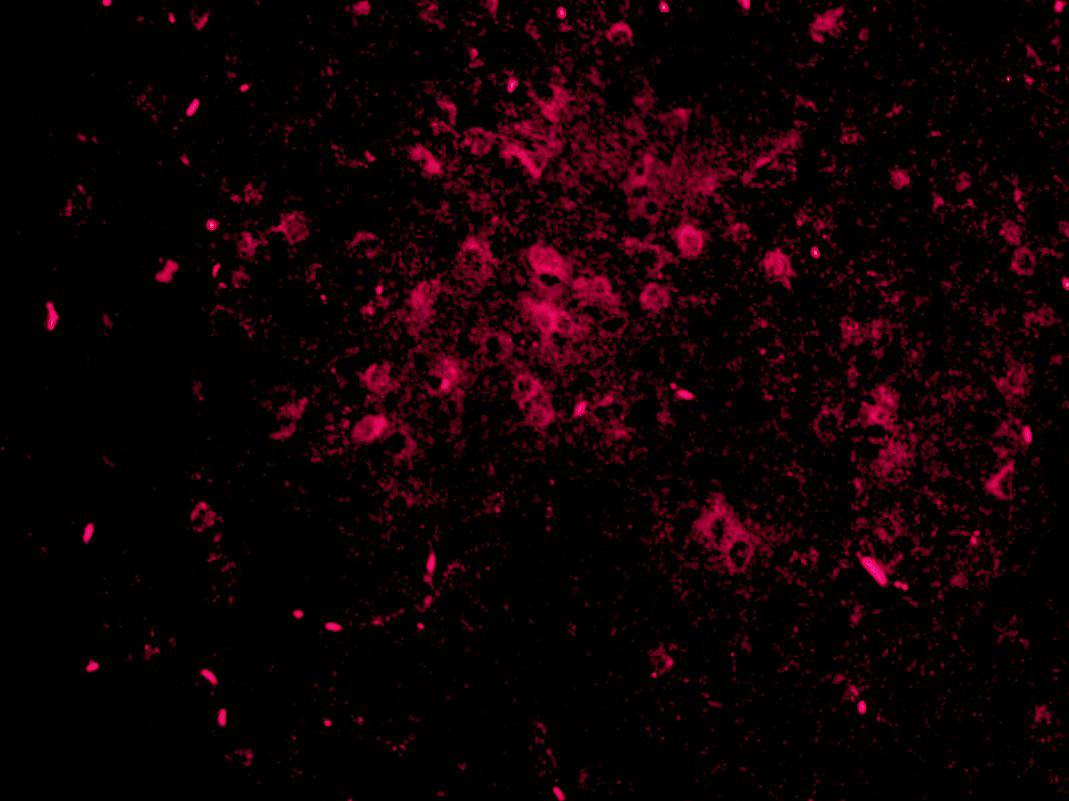

Supplement: Supplementary file 6 [file DataSheet2.ZIP › immunofluorescence of GLUT4/part 2 experiment/Sham/S2 3.jpg]

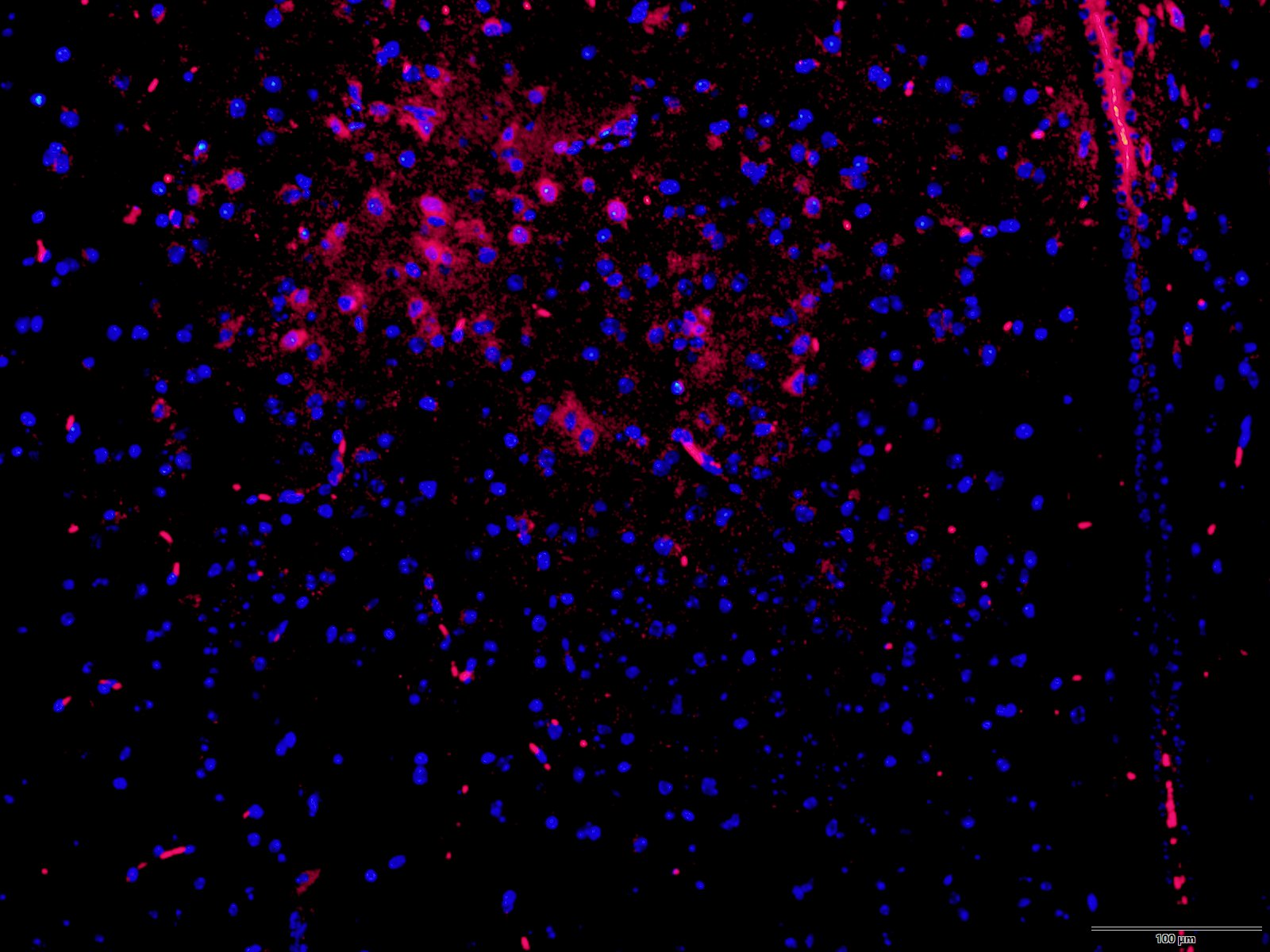

Supplement: Supplementary file 6 [file DataSheet2.ZIP › immunofluorescence of GLUT4/part 2 experiment/Sham/S2.jpg]

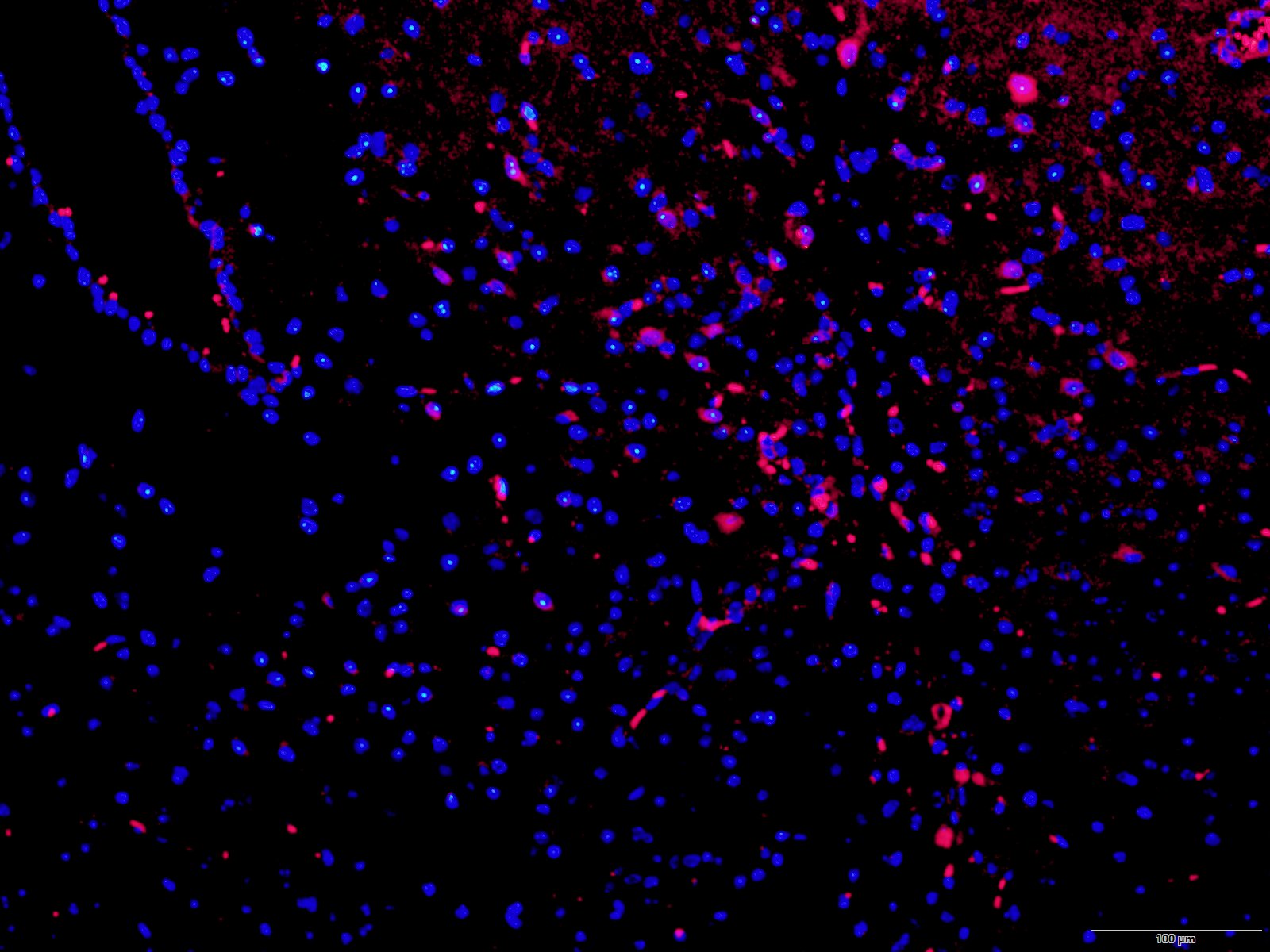

Supplement: Supplementary file 6 [file DataSheet2.ZIP › immunofluorescence of GLUT4/part 2 experiment/Sham/═╝╧±_01.jpg]

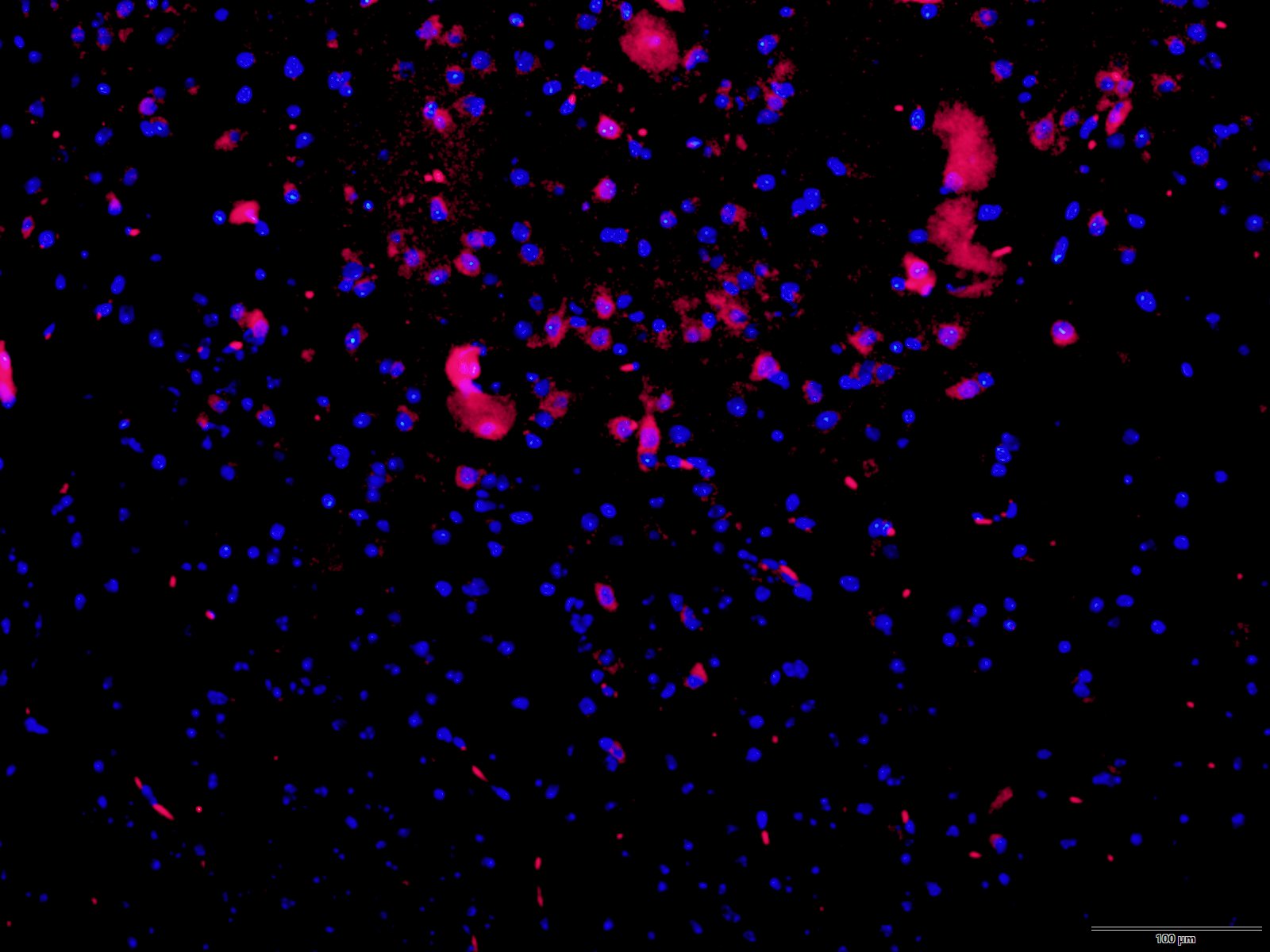

Supplement: Supplementary file 6 [file DataSheet2.ZIP › immunofluorescence of GLUT4/part 2 experiment/Sham/═╝╧±_02.jpg]

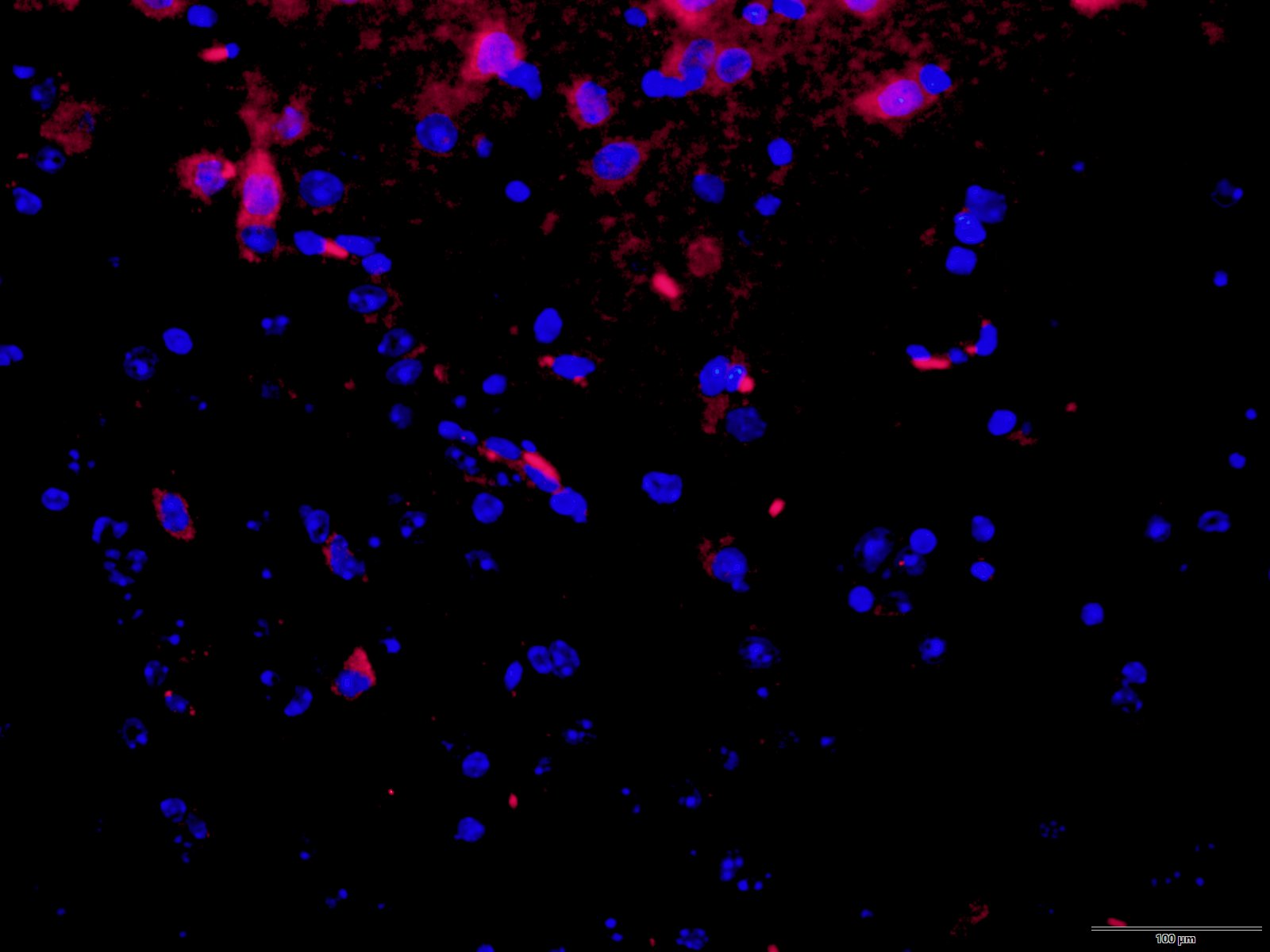

Supplement: Supplementary file 6 [file DataSheet2.ZIP › immunofluorescence of GLUT4/part 2 experiment/Sham/═╝╧±_03.jpg]

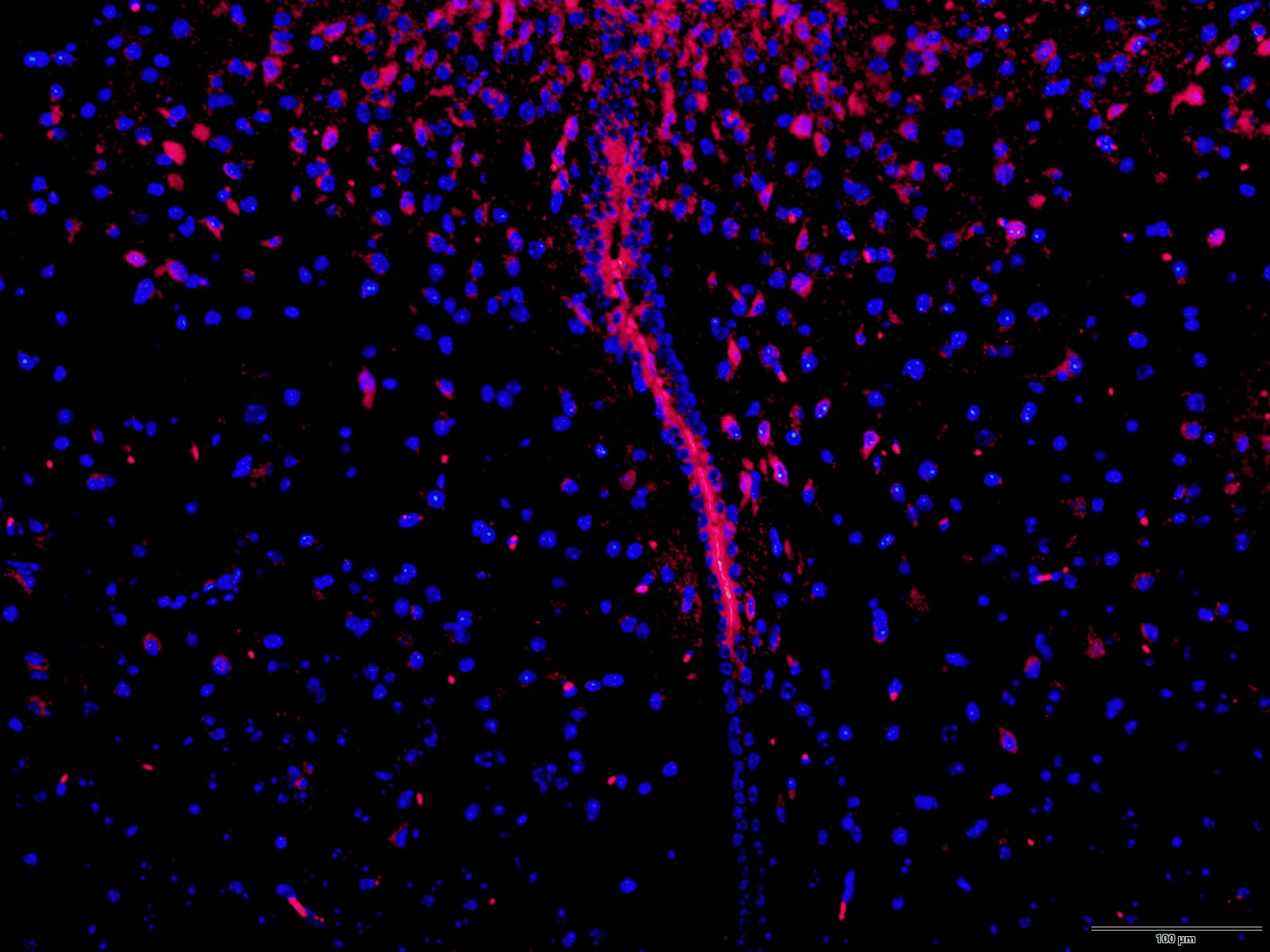

Supplement: Supplementary file 6 [file DataSheet2.ZIP › immunofluorescence of GLUT4/part 2 experiment/Sham/═╝╧±_04.jpg]

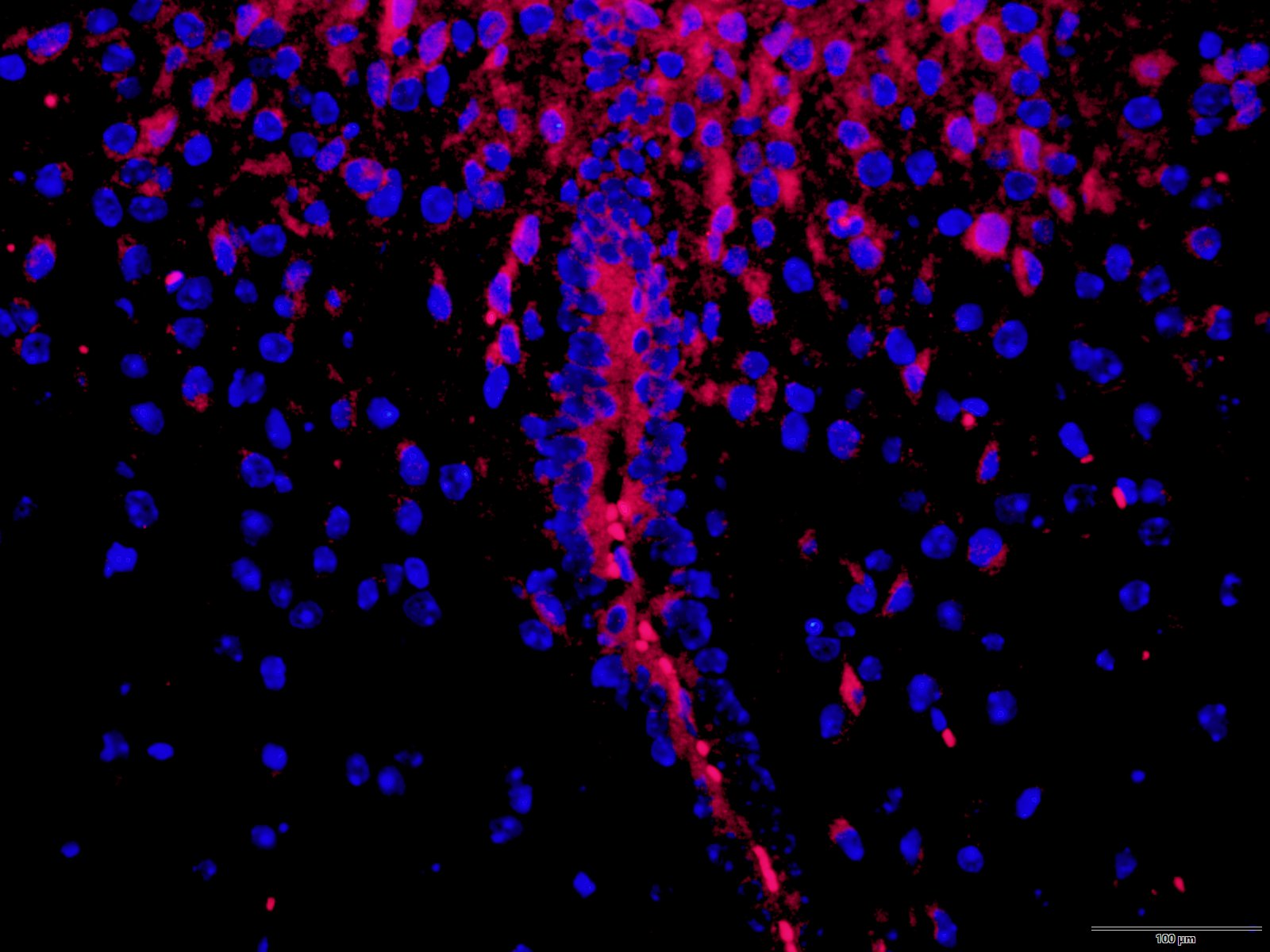

Supplement: Supplementary file 6 [file DataSheet2.ZIP › immunofluorescence of GLUT4/part 2 experiment/Sham/═╝╧±_05.jpg]

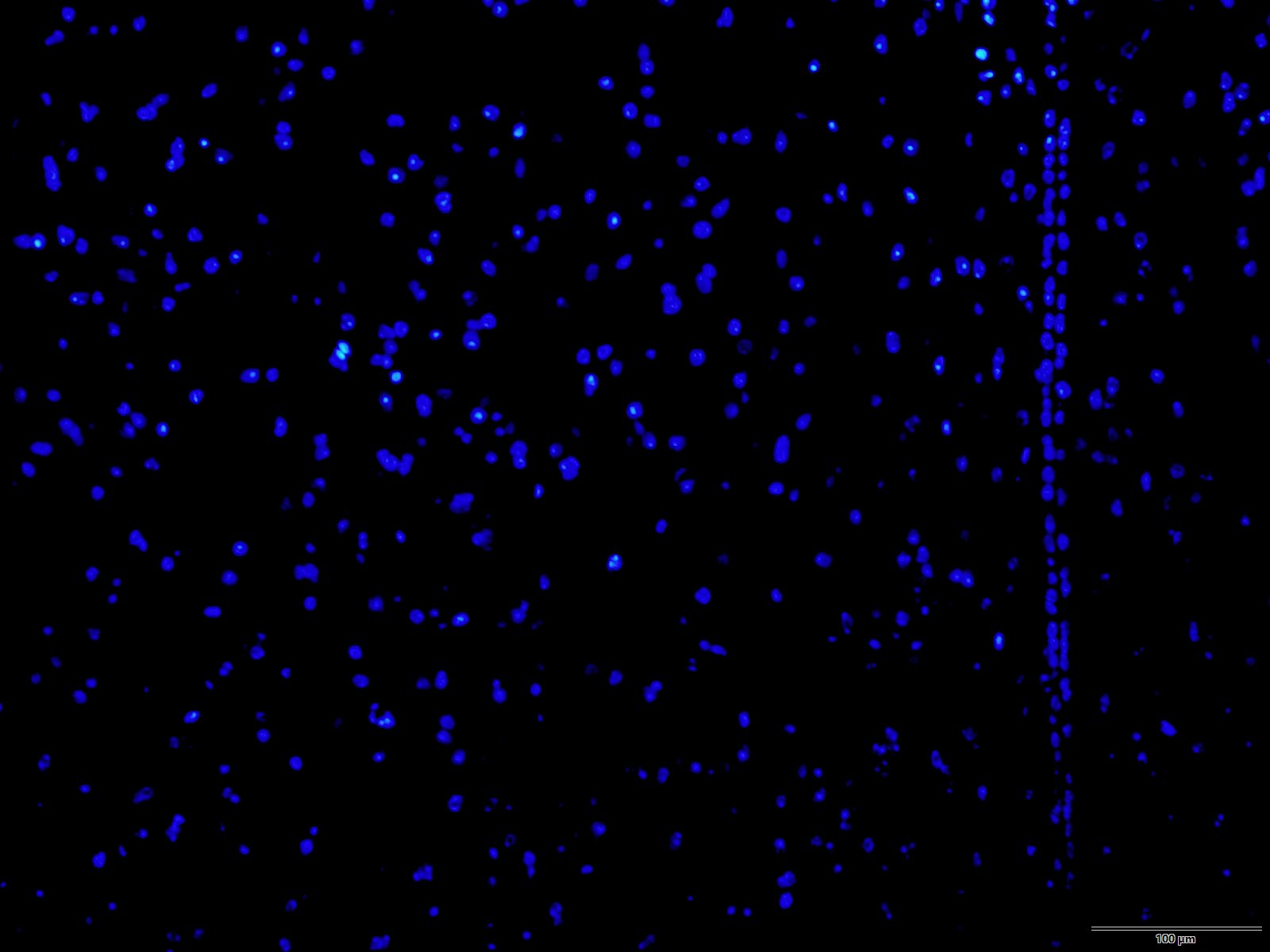

Supplement: Supplementary file 6 [file DataSheet2.ZIP › immunofluorescence of GLUT4/part 2 experiment/XYS/1-1.jpg]
