# Supplementary figures and images for: Xiaoyaosan Exerts Antidepressant-Like Effect by Regulating Autophagy Involves the Expression of GLUT4 in the Mice Hypothalamic Neurons (part 2 of 2)
Source: Front Pharmacol. 2022 Jun 16;13:873646. doi: 10.3389/fphar.2022.873646 (PMC9243304; doi:10.3389/fphar.2022.873646)

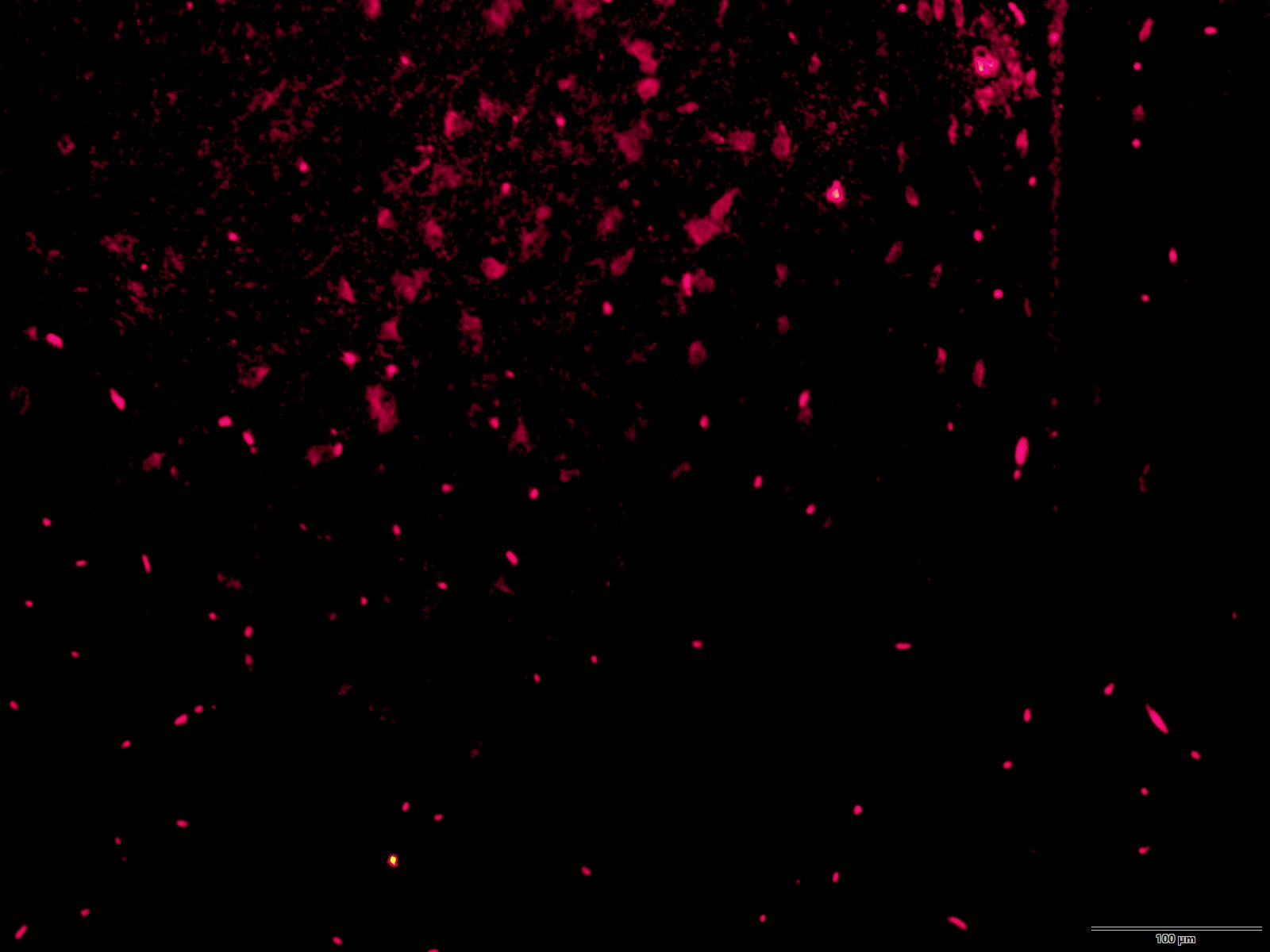

Supplement: Supplementary file 6 [file DataSheet2.ZIP › immunofluorescence of GLUT4/part 2 experiment/XYS/1-2.jpg]

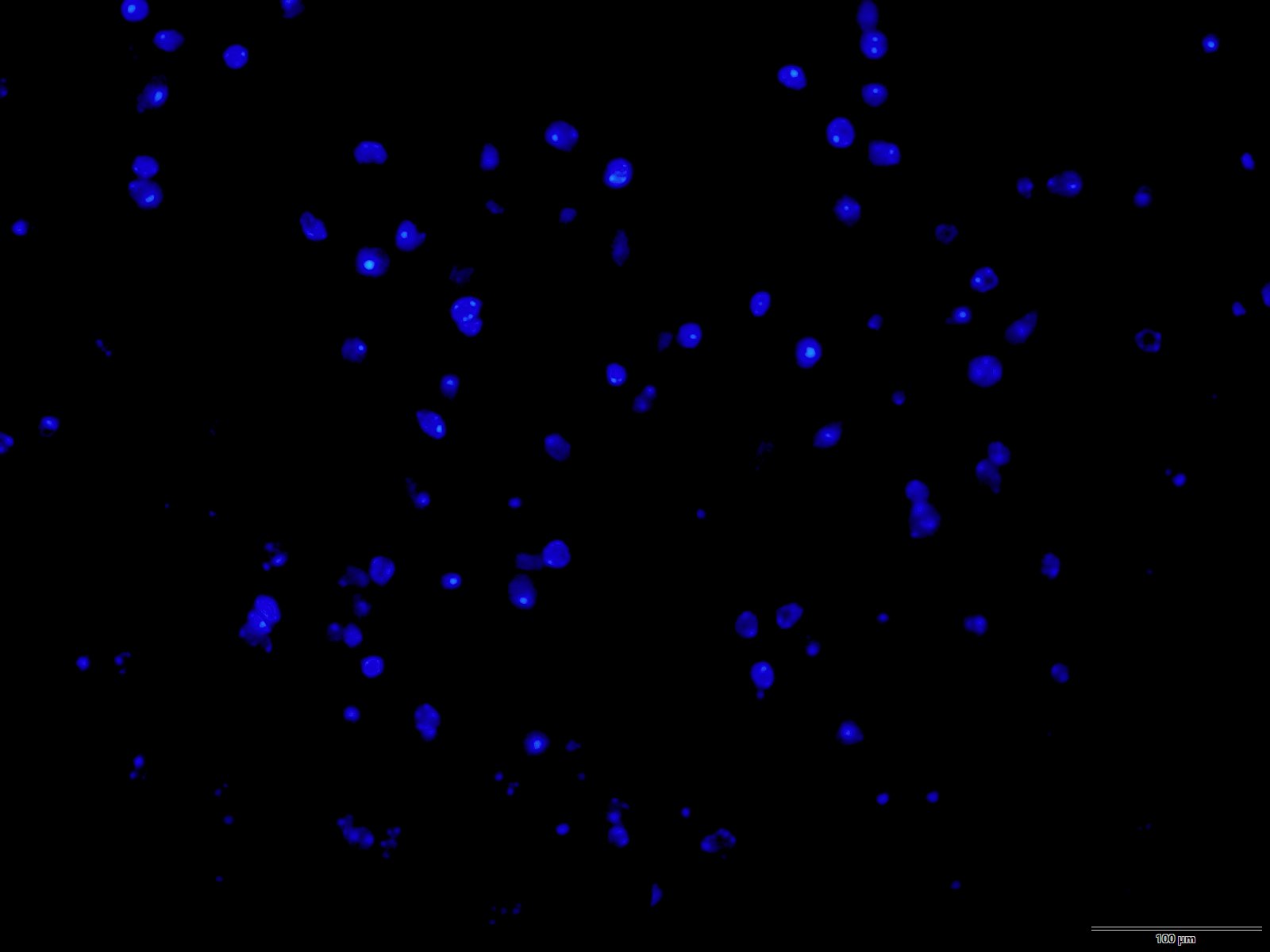

Supplement: Supplementary file 6 [file DataSheet2.ZIP › immunofluorescence of GLUT4/part 2 experiment/XYS/2-1.jpg]

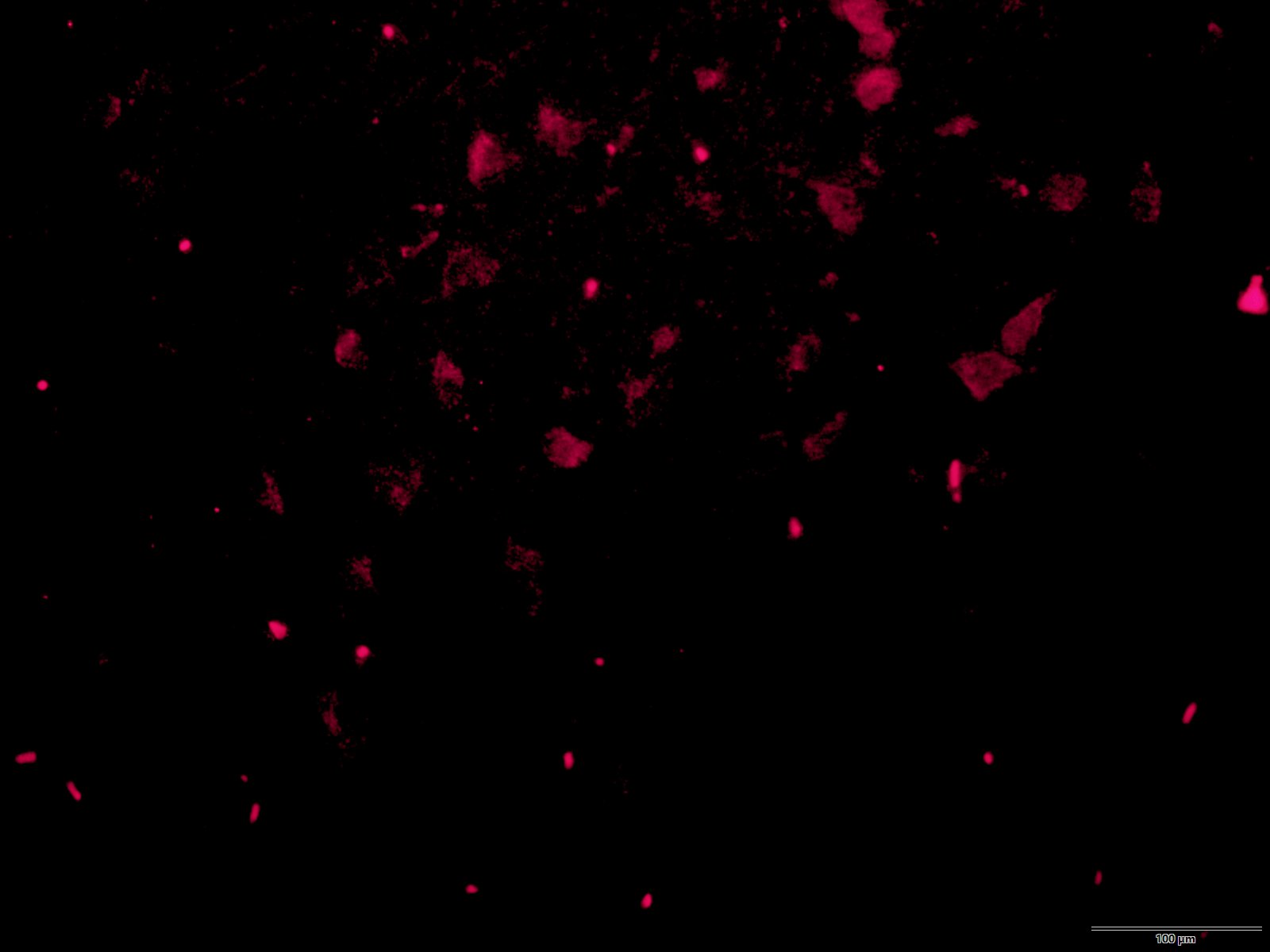

Supplement: Supplementary file 6 [file DataSheet2.ZIP › immunofluorescence of GLUT4/part 2 experiment/XYS/2-2.jpg]

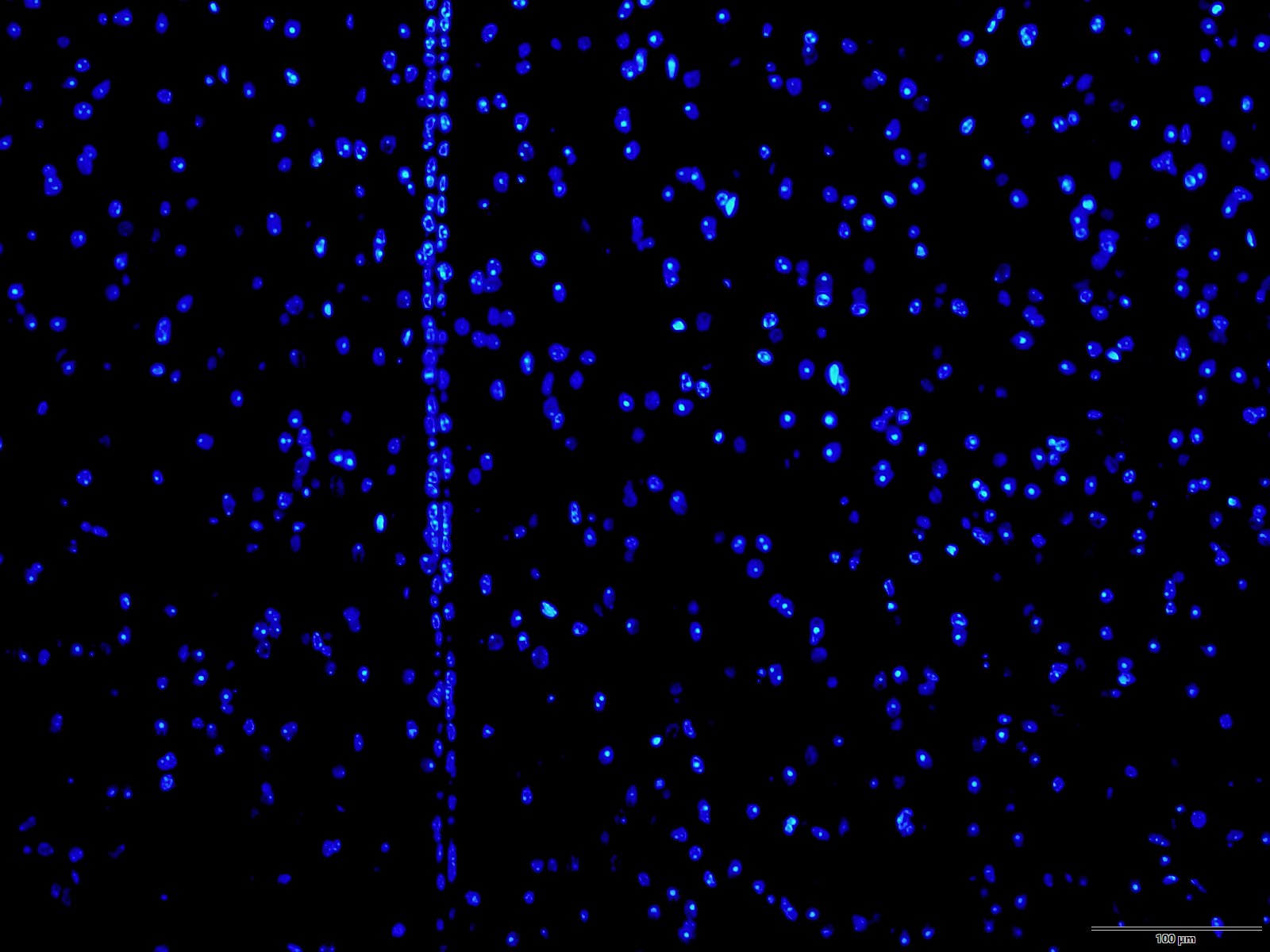

Supplement: Supplementary file 6 [file DataSheet2.ZIP › immunofluorescence of GLUT4/part 2 experiment/XYS/3-1.jpg]

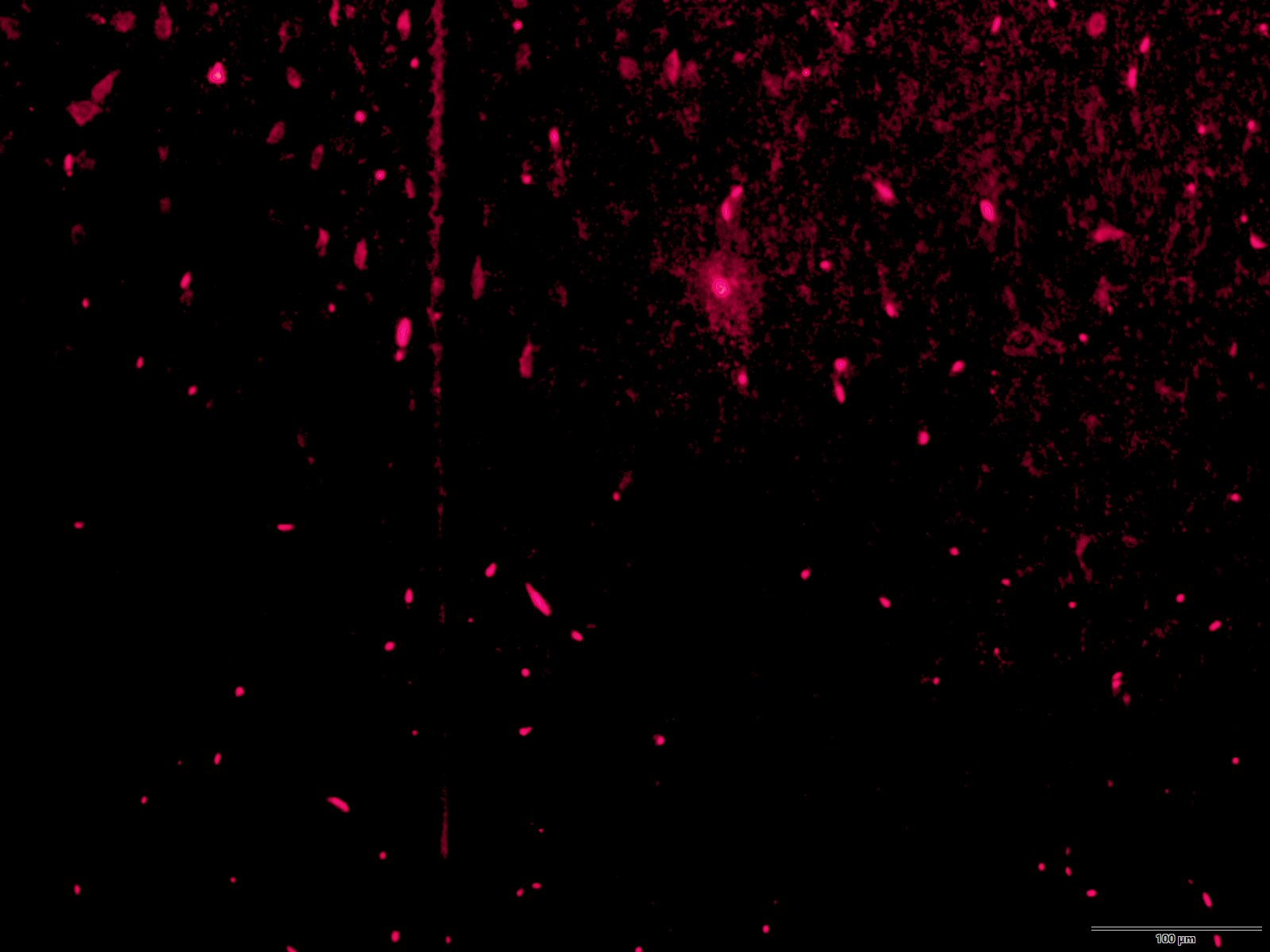

Supplement: Supplementary file 6 [file DataSheet2.ZIP › immunofluorescence of GLUT4/part 2 experiment/XYS/3-2.jpg]

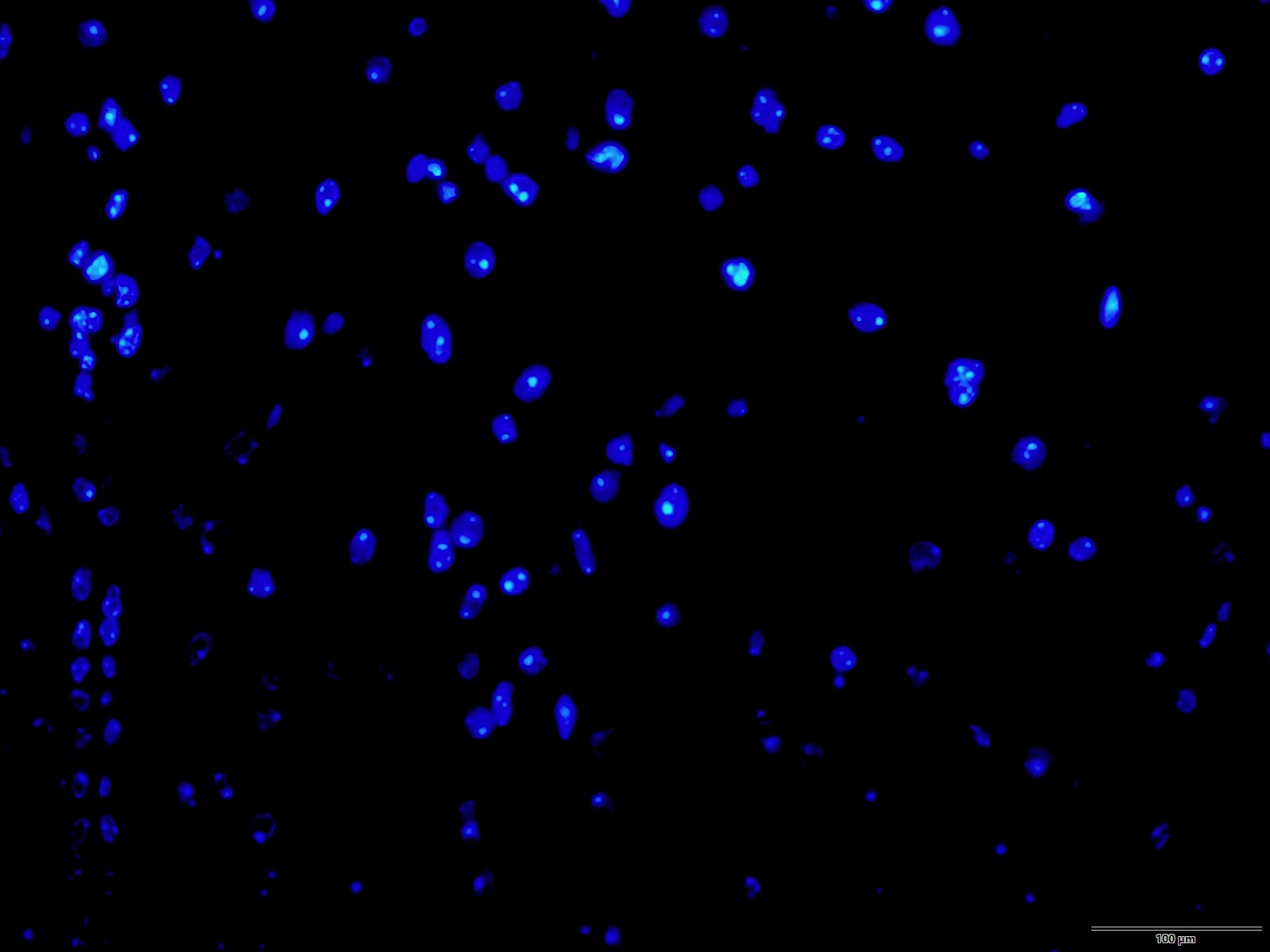

Supplement: Supplementary file 6 [file DataSheet2.ZIP › immunofluorescence of GLUT4/part 2 experiment/XYS/4-1.jpg]

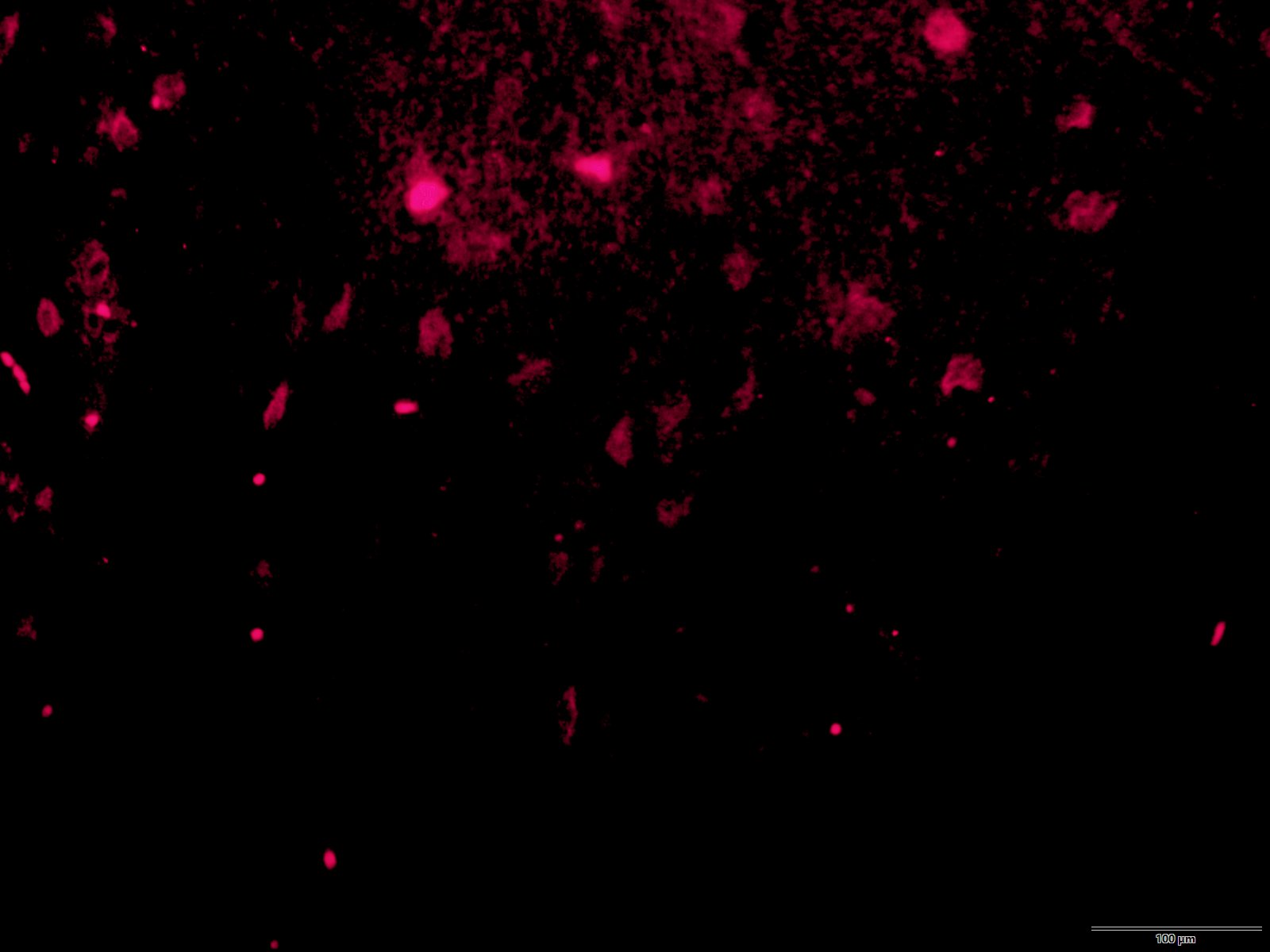

Supplement: Supplementary file 6 [file DataSheet2.ZIP › immunofluorescence of GLUT4/part 2 experiment/XYS/4-2.jpg]

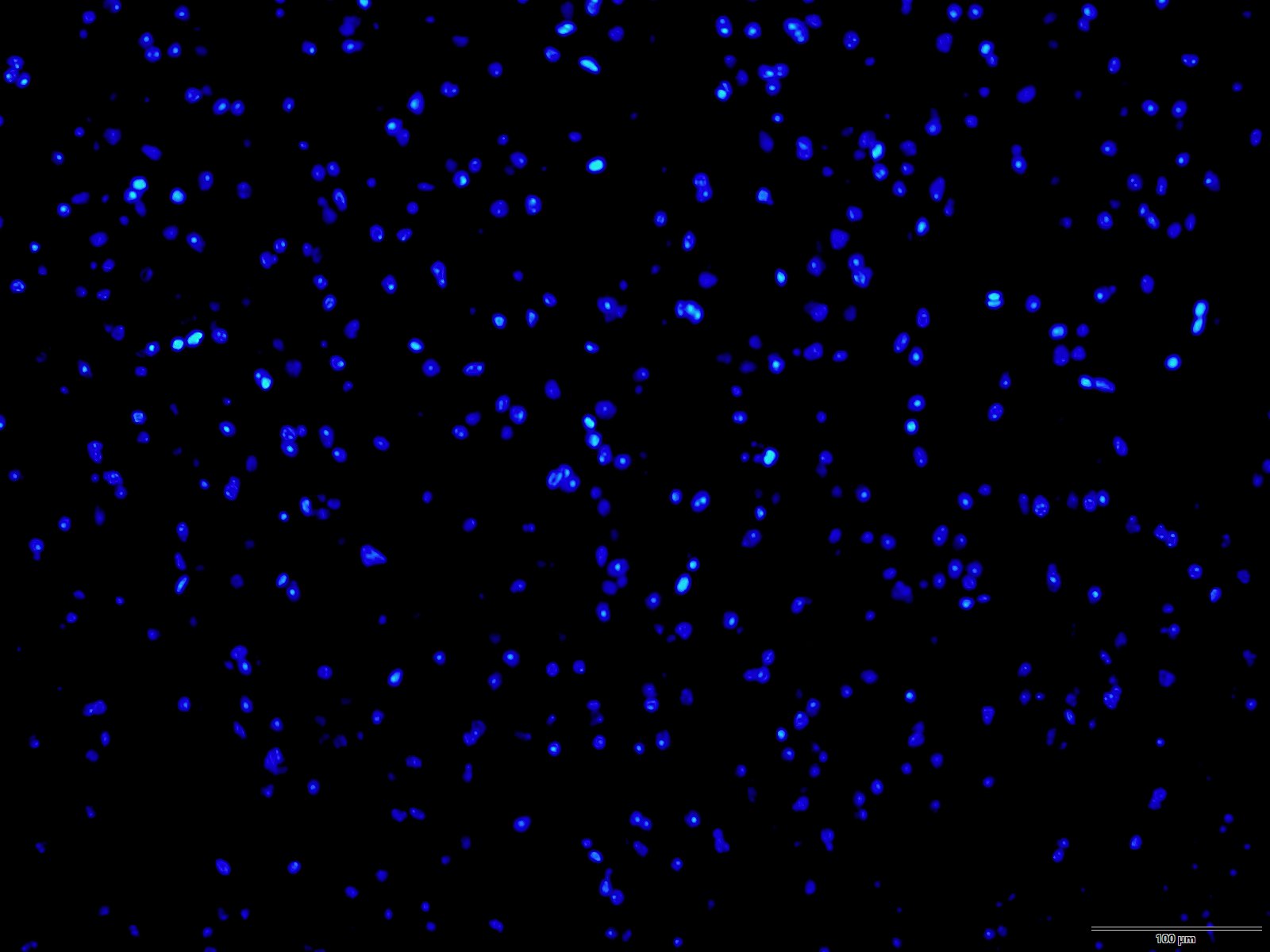

Supplement: Supplementary file 6 [file DataSheet2.ZIP › immunofluorescence of GLUT4/part 2 experiment/XYS/5-1.jpg]

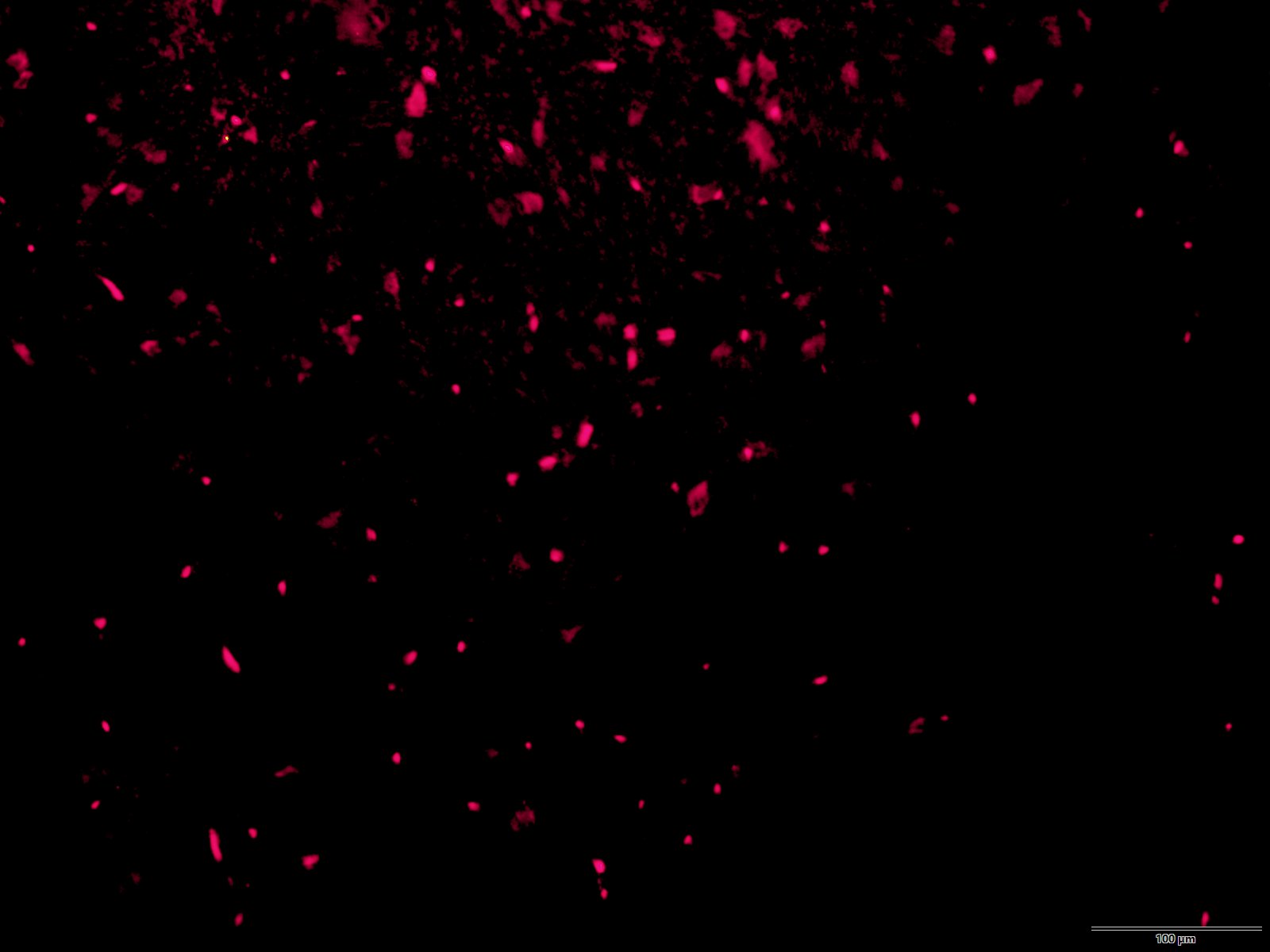

Supplement: Supplementary file 6 [file DataSheet2.ZIP › immunofluorescence of GLUT4/part 2 experiment/XYS/5-2.jpg]

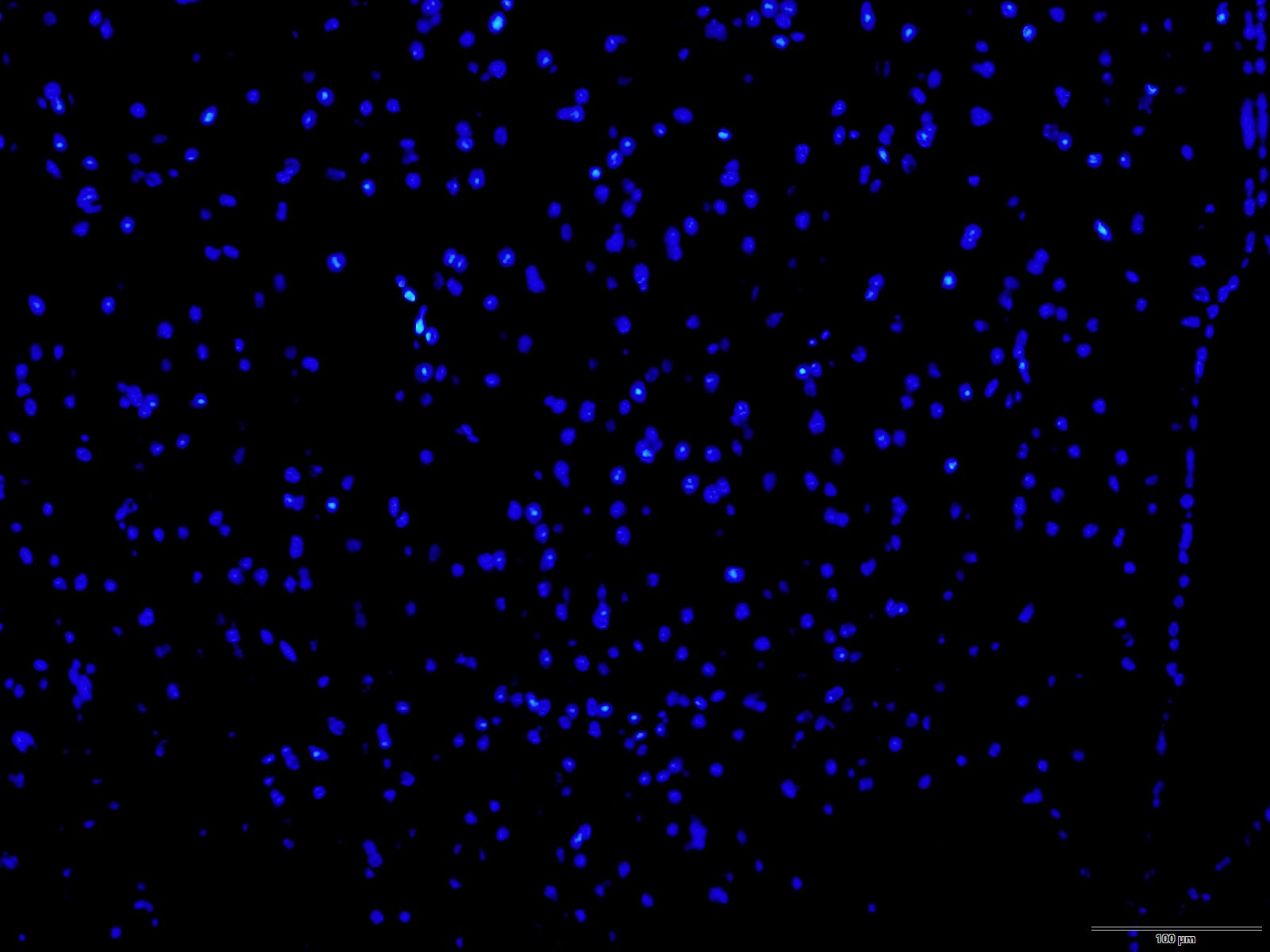

Supplement: Supplementary file 6 [file DataSheet2.ZIP › immunofluorescence of GLUT4/part 2 experiment/XYS/6-1.jpg]

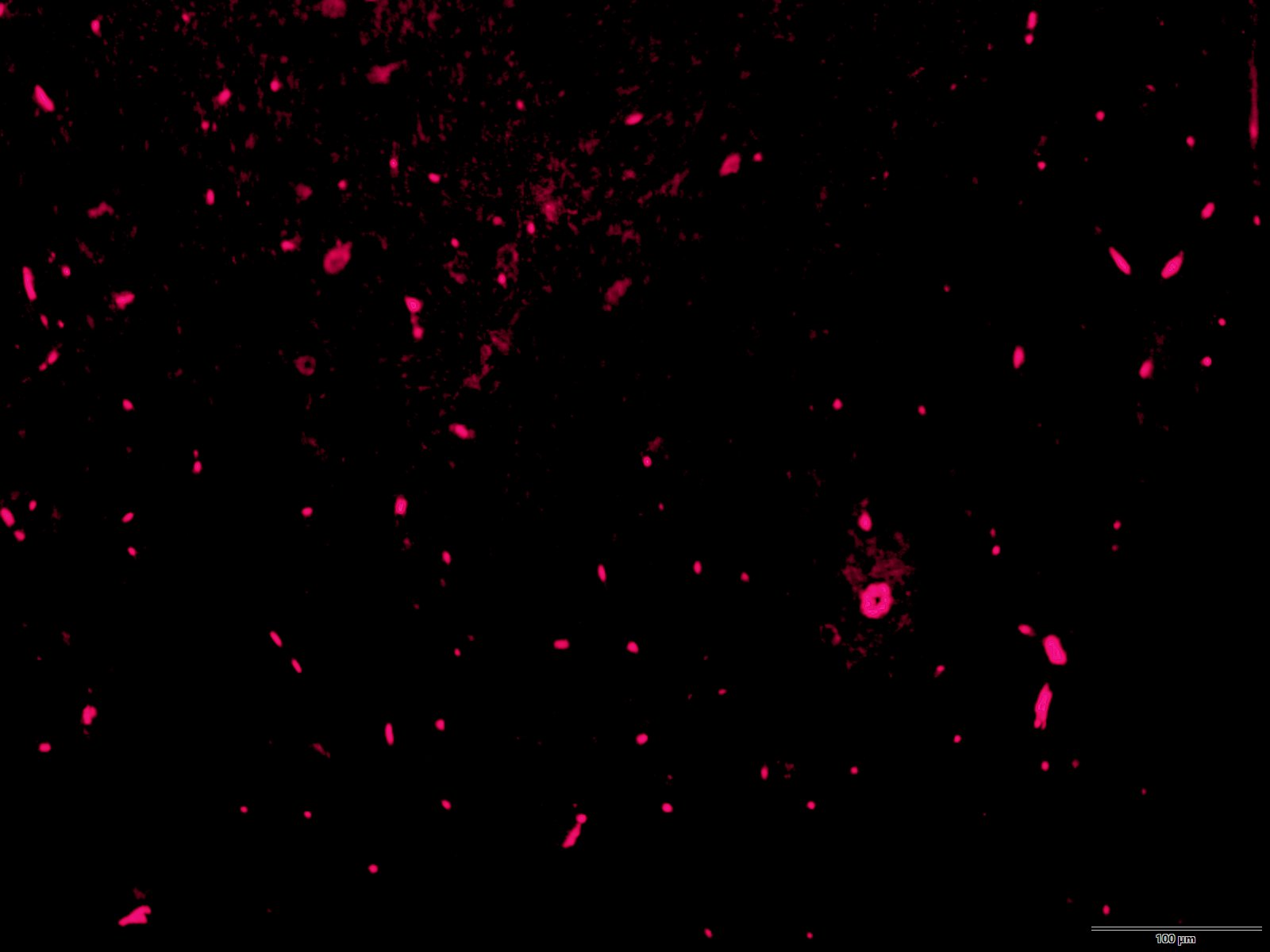

Supplement: Supplementary file 6 [file DataSheet2.ZIP › immunofluorescence of GLUT4/part 2 experiment/XYS/6-2.jpg]

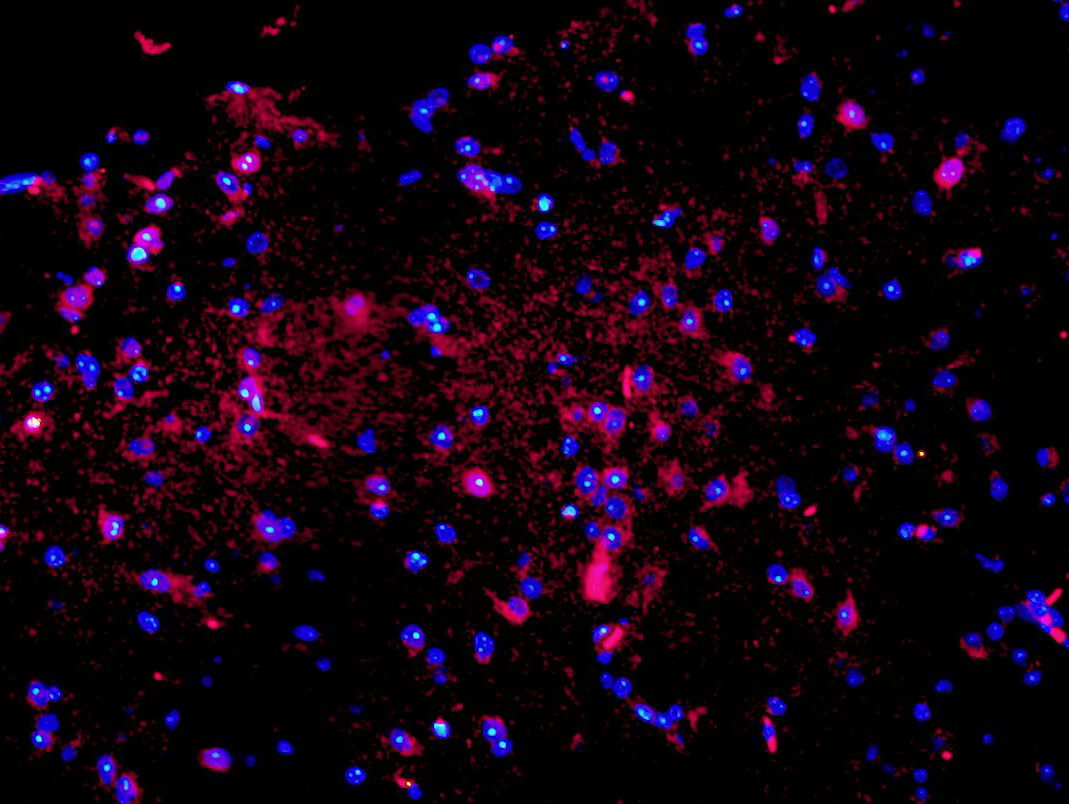

Supplement: Supplementary file 6 [file DataSheet2.ZIP › immunofluorescence of GLUT4/part 2 experiment/XYS/X2 1.jpg]

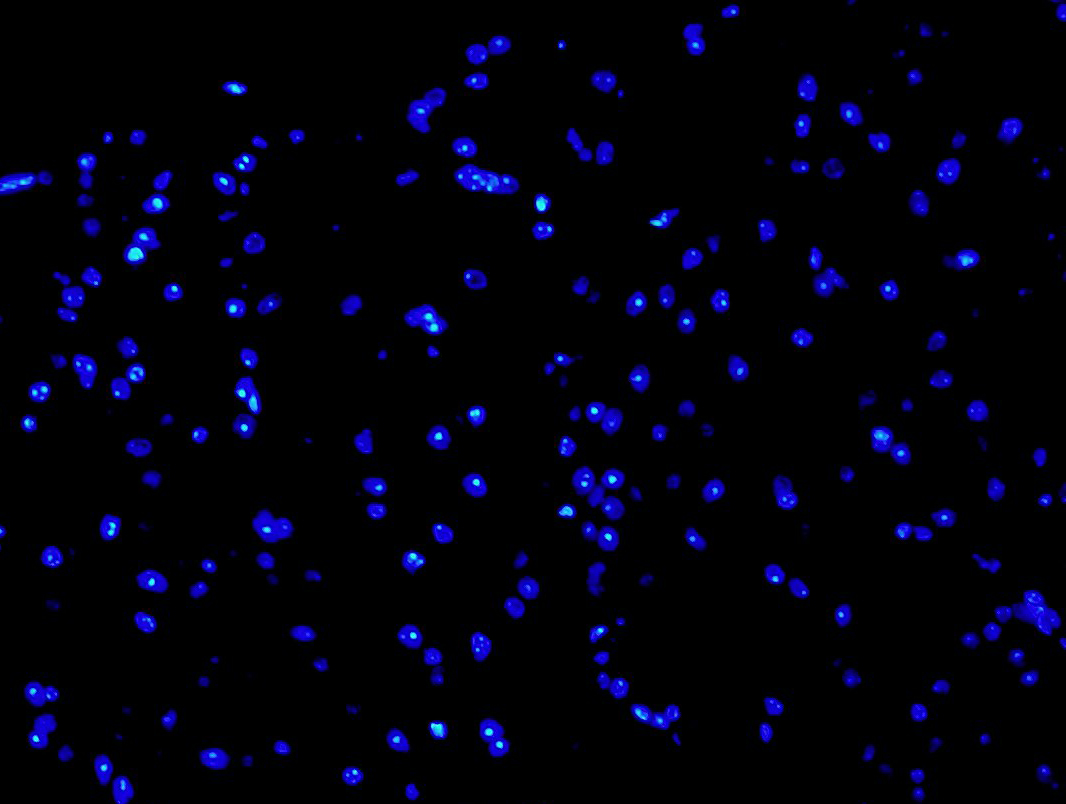

Supplement: Supplementary file 6 [file DataSheet2.ZIP › immunofluorescence of GLUT4/part 2 experiment/XYS/X2 2.jpg]

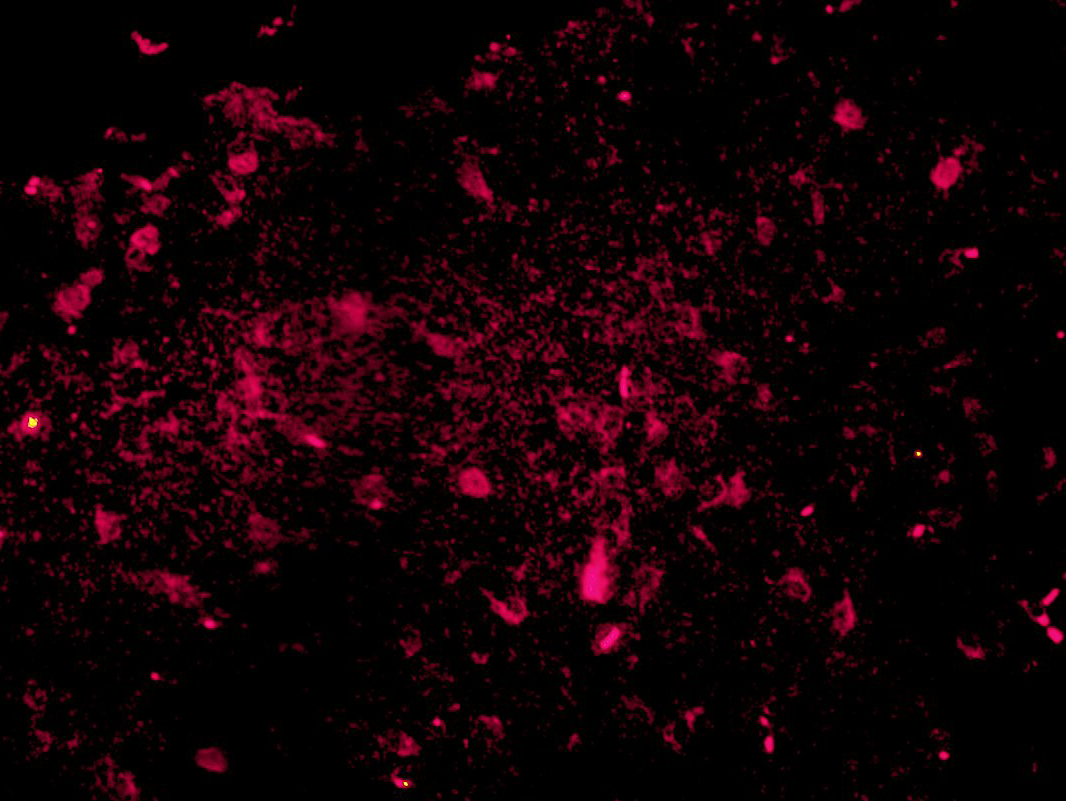

Supplement: Supplementary file 6 [file DataSheet2.ZIP › immunofluorescence of GLUT4/part 2 experiment/XYS/X2 3.jpg]

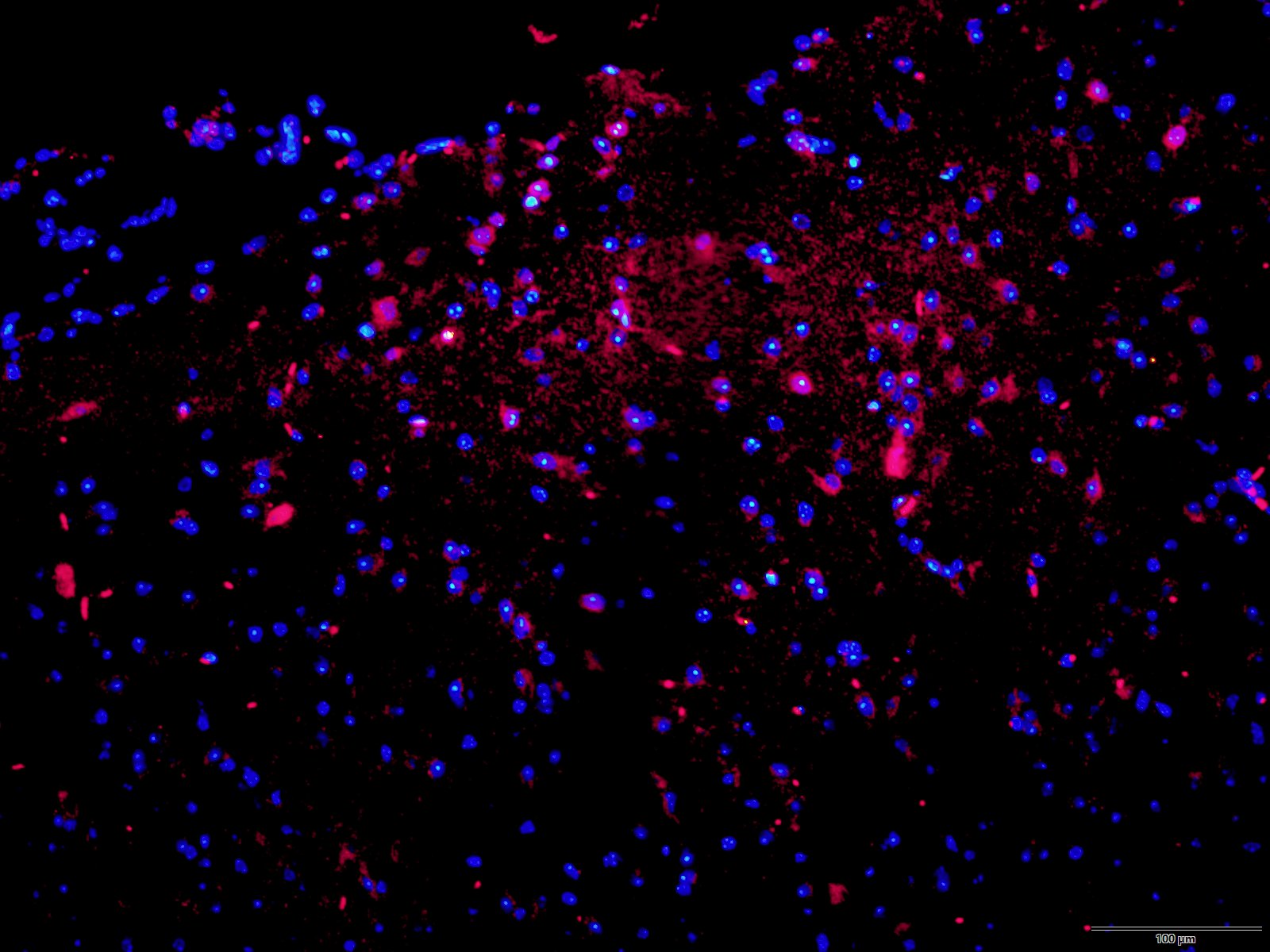

Supplement: Supplementary file 6 [file DataSheet2.ZIP › immunofluorescence of GLUT4/part 2 experiment/XYS/X2.jpg]
